# Signalling pathway impact analysis based on the strength of interaction between genes

**DOI:** 10.1049/iet-syb.2015.0089

**Published:** 2016-08-01

**Authors:** Zhenshen Bao, Xianbin Li, Xiangzhen Zan, Liangzhong Shen, Runnian Ma, Wenbin Liu

**Affiliations:** ^1^ Department of Physics and Electronic information engineering Wenzhou University Wenzhou Zhejiang People's Republic of China; ^2^ College of Information engineering Wenzhou University Wenzhou Zhejiang People's Republic of China; ^3^ Telecommunication Engineering Institute, Air Force Engineering University Xi'an People's Republic of China

**Keywords:** genetics, cancer, biology computing, perturbation theory, biological organs, data analysis, gene interaction strength, cancer‐related pathways, differentially expressed genes, biological experiments, signal perturbation propagation, signalling pathway impact analysis methods, Pearson correlation coefficient, mutual information, colorectal cancer dataset analysis, pancreatic cancer dataset, PSPIA, MSPIA

## Abstract

Signalling pathway analysis is a popular approach that is used to identify significant cancer‐related pathways based on differentially expressed genes (DEGs) from biological experiments. The main advantage of signalling pathway analysis lies in the fact that it assesses both the number of DEGs and the propagation of signal perturbation in signalling pathways. However, this method simplifies the interactions between genes by categorising them only as activation (+1) and suppression (−1), which does not encompass the range of interactions in real pathways, where interaction strength between genes may vary. In this study, the authors used newly developed signalling pathway impact analysis (SPIA) methods, SPIA based on Pearson correlation coefficient (PSPIA), and mutual information (MSPIA), to measure the interaction strength between pairs of genes. In analyses of a colorectal cancer dataset, a lung cancer dataset, and a pancreatic cancer dataset, PSPIA and MSPIA identified more candidate cancer‐related pathways than were identified by SPIA. Generally, MSPIA performed better than PSPIA.

## 1 Introduction

With the establishment of pathway databases, including the KEGG, BioCata, and Reactome databases, analysis of differentially expressed genes (DEGs) has become a dominant analytical method in systemic biological research. In such analysis, the first step is to list DEGs according to gene expression profiles. Next, pathways with significant number of DEGs are identified, after which gene ontology functional enrichment analysis is performed on sets of DEGs in affected pathways, allowing researchers and clinicians to better understand interactions between diseases and genes. Early methods of pathway analysis mainly included techniques based on overexpression analysis (ORA) and functional class scoring (FCS) [1, 2, 3]. ORA methods, such as Onto‐Express [4, 5] and GOEASE [6], determine differentially expressed pathways mainly according to the number of DEGs (NDE). FCS methods, such as gene set enrichment analysis (GSEA) [7], take into consideration coordinated variation of DEGs in each pathway. The main disadvantage of ORA and FCS methods is that they do not consider the position and interaction (activation or inhibition) of genes in signalling pathways. In order to overcome this disadvantage, Tarca *et al.* [8] introduced signalling pathway impact analysis (SPIA), which was the first signalling pathway analysis method to consider the impact of DEGs on pathway perturbation. Voichita *et al.* [9, 10] proposed a gene weighting method to avoid human participation in significance determination during DEG screening, after which they applied SPIA to determine the importance of individual genes in signalling pathways. Ullah [11] improved the SPIA method by substituting the fold‐change used in SPIA with the *t* ‐value produced by the limma software package to increase the accuracy of SPIA. Korucuoglu *et al.* [12] depicted gene relationships within signalling pathways as directed acyclic graphs (Bayesian network) and proposed Bayesian pathway analysis (BPA). Li *et al.* [13] integrated SPIA with subpathway recognition, proposing a subpathway recognition method based on a minimum spanning tree. Additionally, Zhao *et al.* integrated information related to protein–protein interactions and microRNAs to identify disease‐associated pathways [14, 15, 16].

In a signalling pathway, the effect of upstream gene perturbation on downstream genes is closely related to the intensity of the interaction between such pairs of genes. In order to simplify model parameters, SPIA only considers the relationships of activation and inhibition; it does not consider the intensity of interactions between genes. However, interaction intensity can be measured by Pearson correlation analysis or mutual information computation for gene expression profiles. Therefore, we developed modified SPIA methods based on Pearson's correlation coefficient (PSPIA) and mutual information (MSPIA). Through the application of PSPIA and MSPIA to colon cancer, lung cancer, and pancreatic cancer datasets, we found that, in comparison with SPIA, GSEA, and BPA, proposed PSPIA and MSPIA recognise more pathways related to diseases and have more stable performance.

## 2 Materials and methods

### 2.1 Data sets

In this paper, we use the following three cancer data sets: a colon cancer dataset, a lung cancer dataset, and a pancreatic cancer dataset. The colon cancer dataset included 12 colon cancer samples and 10 normal samples (the identification of the datasets in Gene Expression Omnibus dataset (ID) = GSE4107) [17]. The lung cancer dataset obtained by Li‐Jen *et al.* (ID = GSE27262) [18, 19] included 25 lung cancer samples and 25 normal samples. The pancreatic cancer dataset obtained by Pei *et al.* [20] (ID = GSE16515) included 36 pancreatic cancer samples and 16 normal samples.

### 2.2 Signal pathway conversion

In the KEGG database (http://www.kegg.jp/kegg/xml/), every pathway corresponds to an XML document stored in KGML format. There are two main types of nodes in the KGML format: gene product and compound. To compare with SPIA method, we analyse the 137 signalling pathways listed in the SPIA R package. First, we downloaded the signalling pathways from the KEGG database one by one. Next, the graphite package [21] was used to reconstruct each signalling pathway into a gene network. Finally, the DEGs identified using the limma package for R were mapped onto each gene network.

### 2.3 Significance analysis of signalling pathways [8]

Tarca *et al.* argued that the results of expression profile differences in one signalling pathway mainly include the overrepresentation of DEGs and the abnormal perturbation in a given subpathway. SPIA defines a new significance evaluation index, *P*_*G*_, which is calculated by the following formula

(1) $$P_{G}=c-c\ln(c)$$

where *c* = *P*_NDE_ × *P*_PERT_, and *P*_NDE_ and *P*_PERT_ represent the significance of the DEG number and differential signal‐induced perturbation, respectively, in a given pathway.

The first probability *P*_NDE_ = *P* (*X* ≥ *N*_de_ |*H*_0_) captures the significance of a given subpathway *P*_*i*_ by an over‐representation analysis of the NDE contained in the pathway. *H*_0_ represents the null hypothesis, in which random DEGs appear in a given subpathway. The probability *P*_NDE_ is obtained by assuming that the NDE follows a hypergeometric distribution. If the whole genome has a total of *m* genes, of which *t* are involved in the pathway under investigation, while the set of genes submitted for analysis has a total of *n* genes, of which *r* are involved in the same pathway, then the *p* ‐value can be calculated to evaluate the significance of the enrichment of a group of DEGs for that pathway as follows

(2) $$P_{\text{NDE}}(x>r)=1-\sum_{x=0}^{r-1}\left(\left(\begin{array}{c} t\\ x \end{array}\right)\left(\begin{array}{c} m-t\\ h-x \end{array}\right)/\left(\begin{array}{c} m\\ h \end{array}\right)\right)$$

The second probability *P*_PERT_ is calculated based on the amount of perturbation measured in each pathway. The gene perturbation factor is defined as

(3) $$\text{PF}(g_{i})=\Delta E(g_{i})+\sum_{\;j=1}^{k}\beta_{ij}\cdot \frac{\text{PF}(g_{j})}{N_{\text{ds}}(g_{j})}$$

where the term Δ*E* (*g*_*i*_) represents the signed, normalised, measured expression change of gene *g*_*i*_ (log fold‐change if two conditions are compared). The second term in (3) is the sum of the perturbation factors of the gene *g*_*j*_ directly upstream of target gene *g*_*i*_, normalised by the number of downstream genes of each such gene *N*_ds_ (*g*_*j*_). *β*_*ij*_ quantifies the interaction between genes *g*_*i*_ and *g*_*j*_ (activation or inhibition). SPIA uses all |*β* | = 1 in order to minimise the number of model parameters. Detailed information can be found in [8].

To consider the influence of the interaction strength between two adjacent genes with different intensities on perturbation, formula (3) was modified as follows

(4) $$\text{PF}(g_{i})=\Delta E(g_{i})+\sum_{\;j=1}^{s}\beta_{ij}\cdot w_{ij}\frac{\text{PF}(g_{j})}{N_{\text{ds}}(g_{j})}$$

where *w*_*ij*_ represents the intensity of the interaction between two genes. For PSPIA, the interaction intensity is represented by Pearson's correlation coefficient and *w*_*ij*_ is calculated as follows

(5) $$w_{ij}=\left|\frac{1}{n-1}\sum_{k=1}^{n}\left(\frac{E_{ik}-\bar{E_{i}}}{s_{E_{i}}}\right)\left(\frac{E_{\;jk}-\bar{E_{j}}}{s_{E_{j}}}\right)\right|$$

where *n* represents the sample size of the expression profiles, *E*_*ik*_ represents the expression value of gene *g*_*i*_ in the *k* th sample; $\bar{E_{i}}$ represents the average expression value of gene *g*_*i*_; and $s_{E_{i}}$ represents the standard deviation of the expression values of gene *g*_*i*_.

For MSPIA, mutual information is used to describe the non‐linear interaction strength of two genes and *w*_*ij*_ is calculated as follows:

(6) $$w_{ij}=\sum_{y\in Y}\sum_{x\in X}\;p(x,y)\mathrm{lg}\frac{\;p(x,y)}{\;p(x)p(y)}$$

To calculate mutual information, we binarised the normalised microarray data as 1 (overexpression) and 0 (underexpression). *X* and *Y* represent the binarised expression value space of genes *g*_*i*_ and *g*_*j*_, respectively, such as *X* = {1, 1, 1, 1, 1, 1, 0, 1, 0, 0, 0, 0, 0}. Then, the probability space of *X* is as follows

(7) $$\left[\begin{array}{c} X\\ P \end{array}\right]=\left[\begin{array}{cc} 0 & 1\\ \;p(x=0) & \;p(x=1) \end{array}\right]$$

Moreover, the probability space of *Y* is as follows

(8) $$\left[\begin{array}{c} Y\\ P \end{array}\right]=\left[\begin{array}{cc} 0 & 1\\ \;p(y=0) & \;p(y=1) \end{array}\right]$$

The joint probability space of *X* and *Y* is as follows

(9) $$\begin{array}{rl} & \left[\begin{array}{c} X,\;Y\\ P \end{array}\right]\\ & =\left[\begin{array}{cccc} 0,0 & 0,1 & 1,0 & 1,1\\ \;p(x=0,y=0) & \;p(x=0,y=1) & \;p(x=1,y=0) & \;p(x=1,y=1) \end{array}\right] \end{array}$$

Finally, the mutual information between genes *g*_*i*_ and *g*_*j*_ can be caculated as formula (6).

## 3 Results and discussion

In this section, we compared the proposed PSPIA and MSPIA methods with the SPIA method by analysing three cancer data sets. Additionally, we also presented the results of the GSEA method and the recently proposed BPA method. The SPIA R package developed by Tarca *et al.* was applied directly to perform SPIA. GESA was performed using the enrichment analysis software developed by Subramanian. BPA was performed with software developed by Korucuoglu *et al.* We used the limma software package to identify DEGs as genes with a *p* ‐value <0.05.

Fair comparisons of methods are difficult because of the unavailability of gold standard cancer‐pathway association data. In addition to the differences in the techniques used in the compared methods, the calculations of the significance of the identified pathways are also performed differently. Therefore, one common method of comparison is to evaluate them using a specific *p* ‐value threshold for significance. In the PSPIA, MSPIA, SPIA, and BPA results, signalling pathways with *p* ‐values (false discovery rate (FDR)) <0.01 were considered as significant pathways. In the GSEA results, signalling pathways with *q* ‐values <0.01 were considered significant.

Tables 1, 2, 3 list the significant pathways possibly related to cancer that were identified via PSPIA, MSPIA, and SPIA, respectively, in the colon cancer, lung cancer, and pancreatic cancer datasets, as well as the *P*_*G*_ (FDR) of each pathway. In addition, pathways identified via GSEA and BPA are also indicated. Table 4 lists the number of significant pathways identified in each cancer dataset using the five tested methods.

**Table 1:** Significant pathways identified by PSPIA, MSPIA, and SPIA in the colon cancer dataset

No	Pathway name	PSPIA	MSPIA	SPIA	GSEA	BPA	Ref
1	Parkinson's disease	1.48 × 10^−6^	1.23 × 10^−9^	3.02 × 10^−9^			
2	MAPK signalling pathway	1.48 × 10^−6^	5.18 × 10^−4^	7.14 × 10^−4^			[46]
3	Alzheimer's disease	2.43 × 10^−5^	1.43 × 10^−9^	3.57 × 10^−9^			
4	focal adhesion	2.34 × 10^−4^	1.23 × 10^−9^	1.72 × 10^−9^			[39, 40]
5	Huntington's disease	2.48 × 10^−4^	2.02 × 10^−5^	1.85 × 10^−5^			
6	pathways in cancer	2.48 × 10^−4^	9.98 × 10^−5^	1.9 × 10^−4^			
7	axon guidance	0.0082	2.04 × 10^−5^	1.44 × 10^−4^			[51]
8	protein processing in endoplasmic reticulum	5.37 × 10^−4^	0.51	0.51			
9	dilated cardiomyopathy	9.71 × 10^−4^	1.12 × 10^−4^	0.22			
10	transcriptional misregulation in cancer	0.0023	0.035	0.063			
11	colorectal cancer	0.0037	0.10	0.15			
12	bacterial invasion of epithelial cells	0.0066	0.10	0.47			
13	calcium signalling pathway	0.0082	0.21	0.37			[23]
14	salmonella infection	0.0087	0.024	0.11			[24]
15	ECM‐receptor interaction	0.042	5.03 × 10^−6^	5.00 × 10^−6^			[37]
16	PPAR signalling pathway	0.088	1.23 × 10^−5^	3.18 × 10^−5^			[38]

*p ‐value <0.01.

**Table 2:** Significant signalling pathways identified by PSPIA, MSPIA, and SPIA in the lung cancer dataset

No	Pathway name	PSPIA	MSPIA	SPIA	GSEA	BPA	Ref
1	pathways in cancer	1.82 × 10^−11^	8.93 × 10^−9^	8.86 × 10^−9^			
2	protein processing in endoplasmic reticulum	1.07 × 10^−6^	1.06 × 10^−6^	3.19 × 10^−6^			
3	fanconi anemia pathway	5.75 × 10^−6^	2.48 × 10^−6^	3.16 × 10^−6^			[52]
4	focal adhesion	1.29 × 10^−5^	7.60 × 10^−7^	7.54 × 10^−7^		yes	[41, 42]
5	chemokine signalling pathway	2.56 × 10^−5^	0.0010	1.60 × 10^−5^			[53]
6	cell cycle	2.65 × 10^−5^	1.29 × 10^−5^	7.36 × 10^−5^		yes	[54]
7	small cell lung cancer	3.50 × 10^−5^	5.86 × 10^−6^	3.19 × 10^−6^			
8	HTLV‐I infection	6.92 × 10^−5^	1.37 × 10^−4^	3.78 × 10^−4^			
9	Wnt signalling pathway	1.20 × 10^−4^	5.12 × 10^−5^	3.39 × 10^−4^			[49]
10	osteoclast differentiation	3.68 × 10^−4^	0.0010	3.83 × 10^−4^			[55]
11	vascular smooth muscle contraction	5.31 × 10^−4^	3.32 × 10^−4^	3.56 × 10^−6^	yes		[56]
12	ECM‐receptor interaction	6.77 × 10^−4^	7.61 × 10^−4^	0.0041		yes	
13	bacterial invasion of epithelial cells	7.01 × 10^−4^	9.73 × 10^−4^	4.64 × 10^−4^			
14	transcriptional misregulation in cancer	8.59 × 10^−4^	0.0010	6.73 × 10^−4^			
15	MAPK signalling pathway	8.65 × 10^−4^	7.61 × 10^−4^	3.19 × 10^−6^			[47]
16	RNA transport	0.0024	0.0013	0.0023			
17	pancreatic cancer	0.0029	9.70 × 10^−4^	0.0044		yes	
18	TGF‐beta signalling pathway	0.0038	0.0067	0.0019		yes	[57]
19	colorectal cancer	0.0092	0.0091	0.0066		yes	
20	melanogenesis	6.92 × 10^−5^	7.84 × 10^−5^	0.072		yes	
21	natural killer cell mediated cytotoxicity	2.70 × 10^−4^	0.14	0.13			[25]
22	amoebiasis	0.0014	0.0012	0.018			
23	melanoma	0.0029	0.0012	0.012			
24	tight junction	0.0057	0.0010	0.028		yes	[26]
25	Fc gamma R‐mediated phagocytosis	0.0057	0.035	0.017			
26	chagas disease	0.0092	0.0023	0.022			
27	salmonella infection	0.0098	0.0049	0.032			[27]
28	calcium signalling pathway	0.018	4.12 × 10^−4^	3.78 × 10^−4^			[58]
29	cytokine–cytokine receptor interaction	0.011	2.66 × 10^−4^	0.030			
30	chronic myeloid leukemia	0.012	0.0046	0.049			
31	glioma	0.023	0.0068	0.046			
32	Parkinson's disease	0.010	0.0097	0.013			
33	salivary secretion	0.012	0.012	7.36 × 10^−5^			

*p ‐value <0.01

**Table 3:** Significant signalling pathways identified by PSPIA, MSPIA, and SPIA in the pancreatic cancer dataset

No	Pathway Name	PSPIA	MSPIA	SPIA	GSEA	BPA	Ref
1	focal adhesion	8.07 × 10^−9^	6.74 × 10^−7^	1.01 × 10^−6^			[43]
2	ECM‐receptor interaction	4.36 × 10^−8^	8.84 × 10^−8^	8.71 × 10^−8^			
3	pathways in cancer	7.27 × 10^−8^	6.74 × 10^−7^	5.37 × 10^−7^			
4	small cell lung cancer	4.03 × 10^−7^	6.74 × 10^−7^	5.37 × 10^−7^			
5	regulation of actin cytoskeleton	9.33 × 10^−5^	1.50 × 10^−4^	2.74 × 10^−5^			
6	arrhythmogenic right ventricular cardiomyopathy	1.68 × 10^−4^	2.70 × 10^−4^	2.50 × 10^−4^			
7	endocrine and other factor‐regulated calcium reabsorption	0.0023	0.0078	1.01 10^−6^			
8	pancreatic secretion	0.0050	0.0076	0.0089			
9	bacterial invasion of epithelial cells	1.54 × 10^−6^	1.56 × 10^−6^	0.020			
10	gastric acid secretion	8.80 × 10^−5^	0.0097	0.020			[28]
11	salmonella infection	8.80 × 10^−5^	0.0076	0.057			[29]
12	cell cycle	0.0016	0.0014	0.020			[30, 31]
13	pathogenic *Escherichia coli* infection	0.0045	0.0078	0.028			[32]
14	p53 signalling pathway	0.042	0.0076	0.056			[33]
15	Wnt signalling pathway	0.012	0.0078	0.014			[34, 35, 36]
16	mineral absorption	0.011	0.0087	0.014			

*P ‐value < 0.01

**Table 4:** Number of significant signalling pathways identified by all five methods

No	dataset	GEO	control samples	test samples	PSPIA	MSPIA	SPIA	GSEA	BPA
1	colon cancer	GSE4107	10	12	17	10	9	0	0
2	lung cancer	GSE27262	25	25	24	31	21	2	29
3	pancreatic cancer	GSE16515	16	36	11	15	8	0	0

GEO: gene expression omnibus

### 3.1 Comparison of PSPIA, MSPIA, and SPIA results

First, the number of significant pathways identified via PSPIA and MSPIA was much greater than that identified via SPIA. This result suggests that consideration of the interaction intensity of genes improves the sensitivity of SPIA, i.e. the proposed methods can identify more potential disease‐related pathways.

Second, some pathways identified via PSPIA and MSPIA, but not via SPIA, were related to particular type of cancer (see the last column of Tables 1, 2, 3). For example, among the seven pathways identified through PSPIA, but not through SPIA, in the colon cancer dataset, four pathways were related to colon cancer: *colorectal cancer pathway* [22]*, calcium signalling pathway* [23], *transcriptional misregulation in cancer*, and *salmonella infection* [24]. The *colorectal cancer pathway* is directly related to colon cancer [22]. The *calcium signalling pathway* plays an important role in proliferation and migration of colon cancer cells [23]. Salmonella infection can reduce the risk of cancer migration, including that of colon cancer [24]. One pathway was identified via MPSIA in the colon cancer dataset that was not identified by SPIA.

Among the eight pathways identified through PSPIA, but not through SPIA, in the lung cancer dataset, three pathways are related to lung cancer: *the natural killer cell‐mediated cytotoxicity* [25], *tight junction* [26], and *salmonella infection* [27]. *Natural killer cell‐mediated cytotoxicity* pathway activation can aggravate human lung cancer. Expression of tight junction proteins, such as claudins, are up‐regulated or down‐regulated in lung cancer. Among the five pathways identified through MPSIA, but not through SPIA, in the lung cancer dataset, *tight junction pathway* [26] and *salmonella infection pathway* [27] are related to lung cancer.

Among the five pathways identified through PSPIA, but not through SPIA, in the pancreatic cancer dataset, four pathways are associated with pancreatic cancer: the *gastric acid secretion pathway* [28], *salmonella infection pathway* [29], *cell cycle pathway* [30, 31], and *pathogenic Escherichia coli infection pathway* [32]. The pathological characteristics of pancreatic cancer indicate that hyperchlorhydria is directly related to pancreatic cancer. Activation of the cell cycle pathway can inhibit proliferation of pancreatic cancer cells. Some experiments suggest that A1‐R, a Salmonella species, has a significant inhibitory effect on low‐passage pancreatic cancer cells. Among the eight pathways identified through MPSIA, but not through SPIA, in the pancreatic cancer dataset, seven pathways are associated with this cancer: *gastric acid secretion pathway* [28], *salmonella infection pathway* [29]*, cell cycle pathway* [30, 31], *pathogenic Escherichia coli infection pathway* [32], *p53 signalling pathway* [33], and *Wnt signalling pathway* [34, 35, 36].

In comparison with PSPIA, the number of significant pathways identified through MSPIA was larger in the lung cancer and pancreatic cancer datasets, but smaller in the colon cancer dataset. Apart from the quality of the data itself, one possible reason for this difference is that PSPIA was conducted based on the intensity of the linear correlations of genes, while MSPIA was based on the intensity of the more common non‐linear relationship (including linear correlations). When the sample size is sufficiently large, MSPIA can capture more non‐linear interactions between genes than can PSPIA. Conversely, in the case of a small sample size, the linear correlation intensity of genes reflected by PSPIA is more reliable than that provided by MSPIA.

Third, all pathways identified in the three cancer data sets via SPIA were also identified by MSPIA. In the colon cancer dataset, only the *ECM* *‐receptor interaction pathwa* y [37] and *PPAR signalling pathway* [38], which have been reported to be related to colon cancer, were not identified via PSPIA. In the lung cancer dataset, only the *calcium signalling pathway* and *salivary secretion* were not identified via PSPIA. The *calcium signalling pathway* has been reported to be related to lung cancer. In the pancreatic cancer dataset, PSPIA identified all pathways identified via SPIA. The *pancreatic cancer pathway* is obviously a significant pathway associated with pancreatic cancer, but it could not be identified by PSPIA, MSPIA, and SPIA with *p* ‐value <0.01. The *p* ‐values of this pathway were 0.058, 0.059, and 0.13 by PSPIA, MSPIA, and SPIA respectively. Therefore, the PSPIA and MSPIA methods identified this pathway more significantly than the SPIA method. This indicates that the two proposed methods are more efficient than the SPIA methods.

Finally, some pathways were identified by PSPIA, MSPIA, and SPIA that might have no association with the corresponding cancer. For example, the *Parkinson's disease pathway, Alzheimer's disease pathway,* and *Huntington's disease pathway* were identified in the colon cancer dataset by PSPIA, MSPIA, and SPIA. These anomalous results might have been caused by the quality of the microarray data set or by the inherent limitations of SPIA‐based methods. The anomalous pathways were easily eliminated by the subpathway method [13].

### 3.2 Comparison of the capabilities of PSPIA, MSPIA, and SPIA to identify common cancer pathways

Several pathways have been reliably associated with cancer, including the *focal adhesion pathway* [39, 40, 41, 42, 43], *regulation of the actin cytoskeleton pathway* [44]*, MAPK signalling pathway* [45, 46, 47], *ECM‐receptor interaction pathway*, *Wnt signalling pathway* [34, 48, 49], and *p53 signalling pathway* [33, 50]. Table 5 lists the significance of these pathways in the PSPIA, MSPIA, and SPIA results for the three tested datasets. The PSPIA, MSPIA, and SPIA methods identified most of the pathways listed above with *p* ‐values smaller than 0.01, providing indirect evidence for the validity of the SPIA and SPIA‐based methods.

**Table 5:** *p* ‐values of seven common signalling pathways related to cancer that were identified using PSPIA, MSPIA, and SPIA

	Colorectal cancer dataset			Lung cancer dataset			Pancreatic cancer dataset		
Pathway name	PSPIA	MSPIA	SPIA	PSPIA	MSPIA	SPIA	PSPIA	MSPIA	SPIA
focal adhesion	2.3 × 10^−4^	1.2 × 10^−9^	1.7 × 10^−9^	1.3 × 10^−5^	1.5 × 10^−6^	1.5 × 10^−6^	8.1 × 10^−9^	6.7 × 10^−7^	1.0 × 10^−6^
pathways in cancer	2.4 × 10^−4^	9.9 × 10^−5^	1.9 × 10^−4^	1.8 × 10^−11^	8.9 × 10^−9^	8.9 × 10^−9^	7.3 × 10^−8^	6.7 × 10^−7^	5.4 × 10^−7^
regulation of actin cytoskeleton	0.069	0.060	0.034	0.078	0.063	0.062	9.3 × 10^−5^	1.6 × 10^−4^	2.7 × 10^−5^
MAPK signalling pathway	1.4 × 10^−6^	5.2 × 10^−4^	7.1 × 10^−4^	8.7 × 10^−4^	7.6 × 10^−4^	1.9 × 10^−5^	0.10	0.051	0.11
ECM‐receptor interaction	0.042	5.1 × 10^−6^	5.0 × 10^−6^	6.8 × 10^−4^	7.6 × 10^−6^	0.0041	4.4 × 10^−8^	8.8 × 10^−8^	8.7 × 10^−8^
Wnt signalling pathway	0.059	0.02	0.017	1.2 × 10^−4^	5.1 × 10^−5^	0.0037	0.011	0.0078	0.014
p53 signalling pathway	0.33	0.65	0.59	0.17	0.14	0.12	0.04	0.01	0.056

In the three data sets, all pathways had *p* ‐values below 0.1, with the exception of the *p53 signalling pathway*, which had relatively larger *p* ‐values in the significance analysis by each of the three methods for the colon cancer and lung cancer datasets. In the PSPIA results, the *p* ‐values for *regulation of the actin cytoskeleton* (colon cancer dataset), *ECM‐receptor interaction* (colon cancer dataset), *regulation of the actin cytoskeleton* (lung cancer dataset), *MAPK signalling pathway* (pancreatic cancer dataset), and *p53 signalling pathway* (pancreatic cancer dataset) were 0.068, 0.042, 0.078, 0.1 and 0.042, respectively. In the MSPIA results, the *p* ‐values for *regulation of actin cytoskeleton* (colon cancer dataset), *regulation of actin cytoskeleton* (lung cancer dataset), and *MAPK signalling pathway* (pancreatic cancer dataset) were 0.063, 0.06, and 0.05, respectively. In the SPIA results, the *p* ‐values for *regulation of actin cytoskeleton* (colon cancer dataset), *regulation of actin cytoskeleton* (lung cancer dataset), *MAPK signalling pathway* (pancreatic cancer dataset), and *p53 signalling pathway* (pancreatic cancer dataset), were 0.034, 0.062, 0.11, and 0.056, respectively. The number of pathways identified and corresponding *p* ‐values show that MSPIA had the best performance among the three methods, whereas SPIA performed slightly better than PSPIA.

### 3.3 Comparison with other methods

Classical GSEA and BPA methods were also selected for comparison. As shown in Table 4, when the *p* ‐value or *q* ‐value threshold was 0.01, the performance of the two methods was not stable. In the colon cancer and pancreatic cancer datasets, the two methods failed to identify any relevant pathway. In the lung cancer dataset, GSEA identified only two significant pathways, while BPA identified 29 significant pathways. In general, PSPIA, MSPIA, and SPIA were all better than GSEA and BPA. The main disadvantage of GSEA is that it only considers the total NDE in a pathway, but neglects the perturbation induced by differential signals. The major disadvantage of BPA is the loss of some pathway information caused by deleting some relationships according to the correlation of genes during the transformation of the signal pathway into a directed acyclic graph. In addition, in the case of a small sample size, the correlation may be distorted.

## 4 Conclusion

Recognition of pathways related to cancer or other diseases is of great importance for understanding their pathogenetic mechanisms and developing effective therapies. In recent years, researchers have proposed many pathway analysis methods. The advantage of SPIA and major reason for its popularity is the combination of information regarding DEGs and their perturbation within pathways. In real biological pathways, the transmission of a perturbation signal is closely related to the intensity of the interaction between genes. For this reason, in this article based on the SPIA, we adopted PSPIA and MSPIA to obtain the interaction intensity, which was integrated into the transmission process of the perturbation signal. Thus, two signalling pathway analysis methods based on Pearson's correlation coefficient and mutual information were proposed: PSPIA and MSPIA.

With the comparison of results from the colon cancer, lung cancer, and pancreatic cancer datasets, we found that the modified PSPIA and MSPIA methods recognised more signalling pathways possibly related to cancer than did the original SPIA method. In addition, among the significant pathways identified through PSPIA and MSPIA, some pathways were not identified through SPIA, but have been reported as related to some cancers in previous articles. Among the three data sets, MSPIA identified more significant pathways in lung cancer and pancreatic cancer datasets than did PSPIA, but identified fewer significant pathways in the colon cancer dataset. We believe that this difference may be related to the sample size. In the case of a large sample size, mutual information can better interpret the interaction between genes than can Pearson's correlation coefficient. In addition, through comparison of the capacity of the three methods to identify seven common cancer pathways, we found that MSPIA had the best performance, whereas SPIA was slightly better than PSPIA. Therefore, it can be concluded that consideration of the intensity of the interaction between genes can further improve the capacity of SPIA to recognise cancer‐related pathways. In general, the recognition capacity of MSPIA was better than that of PSPIA.

We also investigated the pathway recognition capacities of the GSEA and BPA methods in the three cancer datasets. The results of the analyses indicate that the recognition capacity and stability of GSEA and BPA are inferior to those of SPIA and SPIA‐based methods.

## Supporting information

Supplementary Data

Supplementary Data

Supplementary Data
