# Supplementary material for: Signalling pathway impact analysis based on the strength of interaction between genes
Source: IET Syst Biol. 2016 Aug 1;10(4):147–52. doi: 10.1049/iet-syb.2015.0089 (PMC8687233; doi:10.1049/iet-syb.2015.0089)
Supplement: Supplementary file 1 — Supplementary Data [file SYB2-10-147-s003.docx]

Result obtained by the PSPIA methods in the colon cancer dataset

| No | Name | ID | pSize | NDE | pNDE | tA | pPERT | pG | pGFdr | pGFWER |
| --- | --- | --- | --- | --- | --- | --- | --- | --- | --- | --- |
| 1 | Parkinson's disease | 5012 | 116 | 67 | 1.57E-09 | 0.11423 | 0.338 | 1.18E-08 | 1.48E-06 | 1.62E-06 |
| 2 | MAPK signaling pathway | 4010 | 259 | 121 | 4.73E-08 | 0.765242 | 0.021 | 2.16E-08 | 1.48E-06 | 2.96E-06 |
| 3 | Alzheimer's disease | 5010 | 159 | 82 | 3.52E-08 | 0.027488 | 0.822 | 5.32E-07 | 2.43E-05 | 7.28E-05 |
| 4 | Focal adhesion | 4510 | 199 | 93 | 1.58E-06 | -0.49281 | 0.276 | 6.83E-06 | 0.000234 | 0.000936 |
| 5 | Huntington's disease | 5016 | 171 | 83 | 8.39E-07 | -0.01413 | 0.809 | 1.03E-05 | 0.000248 | 0.001414 |
| 6 | Pathways in cancer | 5200 | 321 | 134 | 1.89E-05 | -1.66163 | 0.038 | 1.09E-05 | 0.000248 | 0.001491 |
| 7 | Protein processing in endoplasmic reticulum | 4141 | 162 | 52 | 0.387687 | 1.803829 | 5.00E-06 | 2.74E-05 | 0.000537 | 0.003759 |
| 8 | Dilated cardiomyopathy | 5414 | 90 | 43 | 0.00053 | 0.30636 | 0.008 | 5.67E-05 | 0.000971 | 0.007768 |
| 9 | Transcriptional misregulation in cancer | 5202 | 158 | 74 | 1.60E-05 | -0.01982 | 0.755 | 0.000149 | 0.002266 | 0.020395 |
| 10 | Colorectal cancer | 5210 | 62 | 27 | 0.022874 | -1.92189 | 0.001 | 0.000267 | 0.003662 | 0.036619 |
| 11 | Bacterial invasion of epithelial cells | 5100 | 70 | 35 | 0.000598 | 0.284637 | 0.081 | 0.00053 | 0.006598 | 0.072573 |
| 12 | Calcium signaling pathway | 4020 | 180 | 69 | 0.018351 | 0.502267 | 0.004 | 0.000772 | 0.008164 | 0.105786 |
| 13 | Axon guidance | 4360 | 128 | 59 | 0.00019 | -0.22781 | 0.387 | 0.000775 | 0.008164 | 0.106137 |
| 14 | Salmonella infection | 5132 | 80 | 40 | 0.000252 | -0.20445 | 0.336 | 0.000877 | 0.008587 | 0.120216 |
| 15 | HTLV-I infection | 5166 | 256 | 105 | 0.000314 | 0.205582 | 0.428 | 0.001334 | 0.012103 | 0.182695 |
| 16 | Tuberculosis | 5152 | 172 | 69 | 0.005741 | 0.59611 | 0.025 | 0.001414 | 0.012103 | 0.193655 |
| 17 | Toxoplasmosis | 5145 | 120 | 56 | 0.000184 | 0.018905 | 0.903 | 0.001608 | 0.01296 | 0.22032 |
| 18 | Toll-like receptor signaling pathway | 4620 | 98 | 40 | 0.0223 | -1.46183 | 0.01 | 0.002098 | 0.015969 | 0.287436 |
| 19 | Pertussis | 5133 | 69 | 32 | 0.004634 | 0.688155 | 0.067 | 0.002818 | 0.02032 | 0.386077 |
| 20 | Osteoclast differentiation | 4380 | 129 | 58 | 0.000477 | 0.064773 | 0.793 | 0.003357 | 0.022999 | 0.459972 |
| 21 | Leukocyte transendothelial migration | 4670 | 113 | 51 | 0.000912 | -0.1318 | 0.701 | 0.005339 | 0.034542 | 0.731446 |
| 22 | Influenza A | 5164 | 160 | 66 | 0.003163 | 0.206923 | 0.211 | 0.005547 | 0.034542 | 0.759933 |
| 23 | Leishmaniasis | 5140 | 66 | 33 | 0.000847 | 0.010811 | 0.925 | 0.006388 | 0.038053 | 0.875223 |
| 24 | Arrhythmogenic right ventricular cardiomyopathy (ARVC) | 5412 | 74 | 36 | 0.000964 | -0.00116 | 0.977 | 0.007502 | 0.0423 | 1 |
| 25 | ECM-receptor interaction | 4512 | 83 | 39 | 0.0014 | 0.069384 | 0.695 | 0.007719 | 0.0423 | 1 |
| 26 | RIG-I-like receptor signaling pathway | 4622 | 70 | 22 | 0.499197 | 0.548203 | 0.003 | 0.011238 | 0.058926 | 1 |
| 27 | Wnt signaling pathway | 4310 | 149 | 61 | 0.005382 | 0.161835 | 0.289 | 0.011613 | 0.058926 | 1 |
| 28 | NOD-like receptor signaling pathway | 4621 | 57 | 28 | 0.002841 | -0.04019 | 0.625 | 0.013021 | 0.061834 | 1 |
| 29 | Progesterone-mediated oocyte maturation | 4914 | 84 | 39 | 0.001845 | -0.00436 | 0.968 | 0.013089 | 0.061834 | 1 |
| 30 | NF-kappa B signaling pathway | 4064 | 88 | 39 | 0.00507 | -0.22202 | 0.383 | 0.014066 | 0.064236 | 1 |
| 31 | Endocrine and other factor-regulated calcium reabsorption | 4961 | 49 | 25 | 0.00244 | -0.00858 | 0.934 | 0.016145 | 0.068648 | 1 |
| 32 | Pancreatic cancer | 5212 | 69 | 31 | 0.009246 | 0.166779 | 0.259 | 0.016846 | 0.068648 | 1 |
| 33 | Epithelial cell signaling in Helicobacter pylori infection | 5120 | 67 | 30 | 0.01094 | -0.18803 | 0.219 | 0.016852 | 0.068648 | 1 |
| 34 | Regulation of actin cytoskeleton | 4810 | 212 | 82 | 0.008514 | -0.37022 | 0.285 | 0.017037 | 0.068648 | 1 |
| 35 | Chagas disease (American trypanosomiasis) | 5142 | 101 | 44 | 0.004489 | 0.110272 | 0.56 | 0.01756 | 0.068734 | 1 |
| 36 | Pancreatic secretion | 4972 | 91 | 36 | 0.046593 | 0.089236 | 0.058 | 0.018683 | 0.0711 | 1 |
| 37 | PPAR signaling pathway | 3320 | 69 | 32 | 0.004634 | 0.010883 | 0.785 | 0.024066 | 0.088097 | 1 |
| 38 | Salivary secretion | 4970 | 82 | 30 | 0.154299 | 0.1511 | 0.024 | 0.024436 | 0.088097 | 1 |
| 39 | Rheumatoid arthritis | 5323 | 83 | 35 | 0.018427 | 0.090321 | 0.208 | 0.025159 | 0.088378 | 1 |
| 40 | Vascular smooth muscle contraction | 4270 | 110 | 46 | 0.009209 | 0.153786 | 0.469 | 0.027835 | 0.094374 | 1 |
| 41 | Herpes simplex infection | 5168 | 171 | 68 | 0.007669 | -0.0978 | 0.573 | 0.028243 | 0.094374 | 1 |
| 42 | Viral myocarditis | 5416 | 66 | 29 | 0.016423 | -0.12276 | 0.28 | 0.029348 | 0.09573 | 1 |
| 43 | Complement and coagulation cascades | 4610 | 67 | 26 | 0.100083 | 1.503499 | 0.048 | 0.030449 | 0.096659 | 1 |
| 44 | B cell receptor signaling pathway | 4662 | 75 | 33 | 0.010709 | -0.14263 | 0.459 | 0.031044 | 0.096659 | 1 |
| 45 | Malaria | 5144 | 47 | 23 | 0.006921 | -0.00754 | 0.766 | 0.03308 | 0.100568 | 1 |
| 46 | Legionellosis | 5134 | 51 | 23 | 0.021907 | -0.13115 | 0.248 | 0.033767 | 0.100568 | 1 |
| 47 | Cholinergic synapse | 4725 | 109 | 42 | 0.051416 | -0.41328 | 0.118 | 0.037039 | 0.105764 | 1 |
| 48 | Staphylococcus aureus infection | 5150 | 48 | 22 | 0.020082 | -0.74385 | 0.306 | 0.037436 | 0.105764 | 1 |
| 49 | Cocaine addiction | 5030 | 50 | 16 | 0.479612 | -0.71291 | 0.013 | 0.037893 | 0.105764 | 1 |
| 50 | Amyotrophic lateral sclerosis (ALS) | 5014 | 52 | 23 | 0.028081 | -0.14563 | 0.227 | 0.0386 | 0.105764 | 1 |
| 51 | Small cell lung cancer | 5222 | 83 | 32 | 0.080367 | 0.238139 | 0.085 | 0.040893 | 0.106341 | 1 |
| 52 | Hedgehog signaling pathway | 4340 | 54 | 19 | 0.286106 | 0.460936 | 0.024 | 0.041069 | 0.106341 | 1 |
| 53 | Apoptosis | 4210 | 86 | 35 | 0.032456 | 0.228017 | 0.212 | 0.041139 | 0.106341 | 1 |
| 54 | Glutamatergic synapse | 4724 | 119 | 48 | 0.016856 | -0.06601 | 0.616 | 0.057809 | 0.146663 | 1 |
| 55 | Gap junction | 4540 | 85 | 36 | 0.015778 | 0.057156 | 0.735 | 0.063283 | 0.157633 | 1 |
| 56 | Melanogenesis | 4916 | 99 | 40 | 0.026534 | 0.16866 | 0.455 | 0.065396 | 0.159986 | 1 |
| 57 | Prion diseases | 5020 | 34 | 15 | 0.069987 | -0.13882 | 0.179 | 0.067396 | 0.161987 | 1 |
| 58 | T cell receptor signaling pathway | 4660 | 108 | 43 | 0.028624 | -0.1187 | 0.458 | 0.069933 | 0.163924 | 1 |
| 59 | Basal cell carcinoma | 5217 | 54 | 21 | 0.127526 | 0.169586 | 0.104 | 0.070595 | 0.163924 | 1 |
| 60 | Shigellosis | 5131 | 60 | 26 | 0.026988 | 0.06485 | 0.512 | 0.072983 | 0.166645 | 1 |
| 61 | GnRH signaling pathway | 4912 | 94 | 39 | 0.017941 | -0.06399 | 0.792 | 0.074654 | 0.167664 | 1 |
| 62 | Serotonergic synapse | 4726 | 114 | 40 | 0.184965 | 0.230438 | 0.096 | 0.089334 | 0.197398 | 1 |
| 63 | Vasopressin-regulated water reabsorption | 4962 | 44 | 19 | 0.055678 | -0.08647 | 0.343 | 0.094689 | 0.205912 | 1 |
| 64 | Mineral absorption | 4978 | 49 | 13 | 0.786605 | -0.0242 | 0.025 | 0.096928 | 0.207486 | 1 |
| 65 | Gastric acid secretion | 4971 | 73 | 29 | 0.065375 | -0.16691 | 0.319 | 0.101566 | 0.214069 | 1 |
| 66 | Oocyte meiosis | 4114 | 109 | 41 | 0.076314 | 0.19835 | 0.298 | 0.108786 | 0.21712 | 1 |
| 67 | Natural killer cell mediated cytotoxicity | 4650 | 127 | 46 | 0.109945 | -0.896 | 0.207 | 0.10885 | 0.21712 | 1 |
| 68 | Dopaminergic synapse | 4728 | 128 | 47 | 0.088278 | 0.158531 | 0.258 | 0.108915 | 0.21712 | 1 |
| 69 | GABAergic synapse | 4727 | 87 | 32 | 0.137075 | -0.09006 | 0.167 | 0.109352 | 0.21712 | 1 |
| 70 | Amoebiasis | 5146 | 106 | 41 | 0.050733 | 0.065719 | 0.503 | 0.119129 | 0.229404 | 1 |
| 71 | Morphine addiction | 5032 | 89 | 36 | 0.033426 | 0.029624 | 0.78 | 0.121155 | 0.229404 | 1 |
| 72 | Pathogenic Escherichia coli infection | 5130 | 51 | 22 | 0.041976 | -0.06991 | 0.627 | 0.122054 | 0.229404 | 1 |
| 73 | Lysosome | 4142 | 117 | 39 | 0.306623 | 0.033266 | 0.086 | 0.122237 | 0.229404 | 1 |
| 74 | SNARE interactions in vesicular transport | 4130 | 35 | 9 | 0.795906 | 0.085098 | 0.039 | 0.138827 | 0.252556 | 1 |
| 75 | Thyroid cancer | 5216 | 29 | 14 | 0.036282 | -0.01023 | 0.863 | 0.139766 | 0.252556 | 1 |
| 76 | Amphetamine addiction | 5031 | 69 | 20 | 0.671091 | 0.300025 | 0.047 | 0.140562 | 0.252556 | 1 |
| 77 | Tight junction | 4530 | 131 | 50 | 0.042876 | 0.027575 | 0.745 | 0.141947 | 0.252556 | 1 |
| 78 | Renal cell carcinoma | 5211 | 69 | 28 | 0.053647 | -0.02464 | 0.808 | 0.17939 | 0.31162 | 1 |
| 79 | Retrograde endocannabinoid signaling | 4723 | 100 | 38 | 0.074516 | 0.028416 | 0.583 | 0.179693 | 0.31162 | 1 |
| 80 | Long-term depression | 4730 | 65 | 22 | 0.339724 | 0.21811 | 0.132 | 0.184064 | 0.315209 | 1 |
| 81 | Intestinal immune network for IgA production | 4672 | 44 | 19 | 0.055678 | 0.012985 | 0.87 | 0.195088 | 0.32721 | 1 |
| 82 | p53 signaling pathway | 4115 | 67 | 24 | 0.221535 | 0.08586 | 0.222 | 0.197326 | 0.32721 | 1 |
| 83 | Vibrio cholerae infection | 5110 | 53 | 20 | 0.171223 | -0.09973 | 0.289 | 0.198237 | 0.32721 | 1 |
| 84 | Chemokine signaling pathway | 4062 | 179 | 65 | 0.065133 | 0.100675 | 0.819 | 0.209695 | 0.342003 | 1 |
| 85 | ErbB signaling pathway | 4012 | 87 | 34 | 0.061347 | -0.02414 | 0.888 | 0.213001 | 0.343307 | 1 |
| 86 | Bile secretion | 4976 | 71 | 22 | 0.530821 | -0.17801 | 0.107 | 0.219709 | 0.350001 | 1 |
| 87 | Jak-STAT signaling pathway | 4630 | 153 | 47 | 0.538729 | 0.15486 | 0.119 | 0.240227 | 0.377495 | 1 |
| 88 | VEGF signaling pathway | 4370 | 71 | 25 | 0.245948 | 0.150936 | 0.264 | 0.242479 | 0.377495 | 1 |
| 89 | Fc gamma R-mediated phagocytosis | 4666 | 91 | 34 | 0.107537 | 0.076357 | 0.683 | 0.265233 | 0.408281 | 1 |
| 90 | Chronic myeloid leukemia | 5220 | 72 | 27 | 0.134802 | 0.072666 | 0.575 | 0.275733 | 0.412502 | 1 |
| 91 | Prostate cancer | 5215 | 89 | 32 | 0.172681 | -0.1856 | 0.45 | 0.276232 | 0.412502 | 1 |
| 92 | Dorso-ventral axis formation | 4320 | 23 | 10 | 0.138052 | 0.013508 | 0.586 | 0.284322 | 0.412502 | 1 |
| 93 | Neurotrophin signaling pathway | 4722 | 119 | 44 | 0.087598 | 0.02342 | 0.927 | 0.285089 | 0.412502 | 1 |
| 94 | Insulin signaling pathway | 4910 | 136 | 48 | 0.147679 | 0.141662 | 0.551 | 0.28551 | 0.412502 | 1 |
| 95 | Alcoholism | 5034 | 128 | 37 | 0.70942 | 0.242735 | 0.115 | 0.286042 | 0.412502 | 1 |
| 96 | Fc epsilon RI signaling pathway | 4664 | 73 | 28 | 0.102642 | -0.05487 | 0.826 | 0.293997 | 0.419558 | 1 |
| 97 | Epstein-Barr virus infection | 5169 | 189 | 61 | 0.354849 | 0.140791 | 0.28 | 0.328777 | 0.464356 | 1 |
| 98 | Long-term potentiation | 4720 | 69 | 24 | 0.274702 | -0.1664 | 0.375 | 0.337151 | 0.471324 | 1 |
| 99 | Notch signaling pathway | 4330 | 47 | 17 | 0.257063 | -0.12734 | 0.416 | 0.345999 | 0.478806 | 1 |
| 100 | Antigen processing and presentation | 4612 | 63 | 18 | 0.69361 | 0.082071 | 0.167 | 0.365523 | 0.498047 | 1 |
| 101 | Measles | 5162 | 128 | 44 | 0.215128 | -0.11108 | 0.542 | 0.367173 | 0.498047 | 1 |
| 102 | Regulation of autophagy | 4140 | 33 | 11 | 0.439944 | 0.099755 | 0.272 | 0.37372 | 0.501958 | 1 |
| 103 | mTOR signaling pathway | 4150 | 62 | 23 | 0.173326 | 0.022891 | 0.782 | 0.406417 | 0.538657 | 1 |
| 104 | Endometrial cancer | 5213 | 52 | 20 | 0.147403 | 0.011719 | 0.928 | 0.408907 | 0.538657 | 1 |
| 105 | Systemic lupus erythematosus | 5322 | 81 | 23 | 0.71865 | -0.28038 | 0.203 | 0.426706 | 0.55675 | 1 |
| 106 | RNA degradation | 3018 | 69 | 23 | 0.366372 | -0.02136 | 0.409 | 0.434276 | 0.561281 | 1 |
| 107 | TGF-beta signaling pathway | 4350 | 81 | 24 | 0.630906 | 0.175409 | 0.245 | 0.443172 | 0.567426 | 1 |
| 108 | Type I diabetes mellitus | 4940 | 39 | 14 | 0.297047 | 0.044015 | 0.544 | 0.456126 | 0.574796 | 1 |
| 109 | Circadian rhythm | 4710 | 21 | 7 | 0.481452 | 0.071641 | 0.337 | 0.457319 | 0.574796 | 1 |
| 110 | Melanoma | 5218 | 71 | 25 | 0.245948 | -0.07424 | 0.7 | 0.475053 | 0.591657 | 1 |
| 111 | Glioma | 5214 | 64 | 23 | 0.222388 | -0.01352 | 0.932 | 0.533451 | 0.658403 | 1 |
| 112 | Bladder cancer | 5219 | 41 | 15 | 0.258815 | 0.017842 | 0.827 | 0.544003 | 0.665432 | 1 |
| 113 | Olfactory transduction | 4740 | 112 | 14 | 0.999999 | -0.29211 | 0.224 | 0.559128 | 0.677881 | 1 |
| 114 | Graft-versus-host disease | 5332 | 34 | 11 | 0.485994 | 0.048017 | 0.478 | 0.571402 | 0.686685 | 1 |
| 115 | Non-small cell lung cancer | 5223 | 54 | 16 | 0.623938 | 0.07641 | 0.382 | 0.58014 | 0.691123 | 1 |
| 116 | Adipocytokine signaling pathway | 4920 | 68 | 23 | 0.335815 | -0.02236 | 0.821 | 0.630929 | 0.745149 | 1 |
| 117 | Acute myeloid leukemia | 5221 | 56 | 19 | 0.352203 | -0.02118 | 0.823 | 0.648814 | 0.754581 | 1 |
| 118 | Hepatitis C | 5160 | 128 | 37 | 0.70942 | -0.1535 | 0.411 | 0.650925 | 0.754581 | 1 |
| 119 | Cell cycle | 4110 | 122 | 37 | 0.578595 | 0.098044 | 0.512 | 0.656642 | 0.754581 | 1 |
| 120 | Type II diabetes mellitus | 4930 | 47 | 16 | 0.366052 | 0.018077 | 0.819 | 0.660947 | 0.754581 | 1 |
| 121 | Cytosolic DNA-sensing pathway | 4623 | 59 | 15 | 0.85039 | 0.03841 | 0.381 | 0.68915 | 0.774742 | 1 |
| 122 | Allograft rejection | 5330 | 33 | 11 | 0.439944 | 0.024544 | 0.738 | 0.689916 | 0.774742 | 1 |
| 123 | RNA transport | 3013 | 145 | 46 | 0.434835 | -0.0063 | 0.848 | 0.736619 | 0.812741 | 1 |
| 124 | African trypanosomiasis | 5143 | 32 | 11 | 0.393569 | 0.002406 | 0.948 | 0.740946 | 0.812741 | 1 |
| 125 | Autoimmune thyroid disease | 5320 | 47 | 14 | 0.61366 | 0.037701 | 0.609 | 0.741552 | 0.812741 | 1 |
| 126 | Cytokine-cytokine receptor interaction | 4060 | 251 | 79 | 0.430891 | 0.018883 | 0.924 | 0.764811 | 0.83158 | 1 |
| 127 | Asthma | 5310 | 26 | 9 | 0.406957 | 0 | 1 | 0.772831 | 0.833683 | 1 |
| 128 | Taste transduction | 4742 | 45 | 8 | 0.984176 | 0.040048 | 0.491 | 0.834665 | 0.893353 | 1 |
| 129 | Carbohydrate digestion and absorption | 4973 | 38 | 10 | 0.777759 | 0.016844 | 0.65 | 0.850385 | 0.899126 | 1 |
| 130 | Sulfur relay system | 4122 | 9 | 3 | 0.558239 | -0.0018 | 0.913 | 0.853185 | 0.899126 | 1 |
| 131 | Phosphatidylinositol signaling system | 4070 | 79 | 23 | 0.66763 | -0.00397 | 0.909 | 0.909969 | 0.951647 | 1 |
| 132 | Viral carcinogenesis | 5203 | 182 | 47 | 0.9401 | -0.01067 | 0.667 | 0.919711 | 0.954548 | 1 |
| 133 | Aldosterone-regulated sodium reabsorption | 4960 | 39 | 11 | 0.693892 | 0.00161 | 0.95 | 0.933906 | 0.961994 | 1 |
| 134 | Maturity onset diabetes of the young | 4950 | 23 | 6 | 0.757769 | -0.00272 | 0.921 | 0.948922 | 0.970167 | 1 |
| 135 | Fanconi anemia pathway | 3460 | 48 | 7 | 0.997171 | 0.008925 | 0.73 | 0.959086 | 0.973295 | 1 |
| 136 | Phototransduction | 4744 | 28 | 6 | 0.903457 | 0.00011 | 0.998 | 0.994997 | 0.996519 | 1 |
| 137 | Neuroactive ligand-receptor interaction | 4080 | 264 | 57 | 0.9997 | -0.00442 | 0.918 | 0.996519 | 0.996519 | 1 |

Result obtained by the MSPIA methods in the colon cancer dataset

| No | Name | ID | pSize | NDE | pNDE | tA | pPERT | pG | pGFdr | pGFWER |
| --- | --- | --- | --- | --- | --- | --- | --- | --- | --- | --- |
| 1 | Focal adhesion | 4510 | 199 | 68 | 8.54E-08 | 4.967822 | 5.00E-06 | 1.26E-11 | 1.23E-09 | 1.74E-09 |
| 2 | Parkinson's disease | 5012 | 116 | 53 | 1.53E-11 | -1.11272 | 0.04 | 1.79E-11 | 1.23E-09 | 2.47E-09 |
| 3 | Alzheimer's disease | 5010 | 159 | 66 | 1.15E-11 | -0.35711 | 0.095 | 3.11E-11 | 1.43E-09 | 4.30E-09 |
| 4 | ECM-receptor interaction | 4512 | 83 | 27 | 0.00148 | 2.294276 | 5.00E-06 | 1.46E-07 | 5.03E-06 | 2.01E-05 |
| 5 | PPAR signaling pathway | 3320 | 69 | 30 | 1.41E-06 | -0.39052 | 0.017 | 4.45E-07 | 1.23E-05 | 6.14E-05 |
| 6 | Huntington's disease | 5016 | 171 | 60 | 1.69E-07 | -0.08797 | 0.291 | 8.76E-07 | 2.02E-05 | 0.000121 |
| 7 | Axon guidance | 4360 | 128 | 46 | 2.10E-06 | 1.058822 | 0.028 | 1.04E-06 | 2.04E-05 | 0.000143 |
| 8 | Pathways in cancer | 5200 | 321 | 84 | 0.000366 | 3.085654 | 0.001 | 5.79E-06 | 9.98E-05 | 0.000799 |
| 9 | Dilated cardiomyopathy | 5414 | 90 | 22 | 0.094476 | -0.80551 | 5.00E-06 | 7.35E-06 | 0.000113 | 0.001015 |
| 10 | MAPK signaling pathway | 4010 | 259 | 76 | 1.23E-05 | 0.563522 | 0.222 | 3.76E-05 | 0.000519 | 0.005187 |
| 11 | Wnt signaling pathway | 4310 | 149 | 44 | 0.000662 | -0.38474 | 0.236 | 0.001525 | 0.017533 | 0.210392 |
| 12 | Salmonella infection | 5132 | 80 | 25 | 0.003979 | 0.574771 | 0.06 | 0.00223 | 0.023671 | 0.30772 |
| 13 | Lysosome | 4142 | 117 | 35 | 0.001748 | -0.0684 | 0.179 | 0.002837 | 0.027969 | 0.391564 |
| 14 | Transcriptional misregulation in cancer | 5202 | 158 | 42 | 0.007253 | -0.13432 | 0.06 | 0.003804 | 0.034993 | 0.524891 |
| 15 | Circadian rhythm | 4710 | 21 | 9 | 0.008468 | -0.37042 | 0.065 | 0.004681 | 0.040377 | 0.646027 |
| 16 | Prion diseases | 5020 | 34 | 12 | 0.014995 | 0.262284 | 0.059 | 0.007104 | 0.056938 | 0.980408 |
| 17 | Influenza A | 5164 | 160 | 38 | 0.054748 | -1.05119 | 0.017 | 0.007427 | 0.056938 | 1 |
| 18 | ErbB signaling pathway | 4012 | 87 | 25 | 0.012657 | -0.34453 | 0.084 | 0.008343 | 0.060094 | 1 |
| 19 | Regulation of actin cytoskeleton | 4810 | 212 | 57 | 0.001524 | 0.186627 | 0.733 | 0.008709 | 0.060094 | 1 |
| 20 | Pertussis | 5133 | 69 | 22 | 0.005103 | 2.197626 | 0.347 | 0.012991 | 0.085372 | 1 |
| 21 | Renal cell carcinoma | 5211 | 69 | 22 | 0.005103 | -0.14983 | 0.379 | 0.014019 | 0.087937 | 1 |
| 22 | Gap junction | 4540 | 85 | 24 | 0.017829 | 0.40207 | 0.148 | 0.018306 | 0.103534 | 1 |
| 23 | Insulin signaling pathway | 4910 | 136 | 37 | 0.007609 | -0.4089 | 0.366 | 0.01917 | 0.103534 | 1 |
| 24 | Bile secretion | 4976 | 71 | 13 | 0.560952 | -1.02139 | 0.005 | 0.019287 | 0.103534 | 1 |
| 25 | Bacterial invasion of epithelial cells | 5100 | 70 | 18 | 0.082565 | 0.583938 | 0.035 | 0.019785 | 0.103534 | 1 |
| 26 | Notch signaling pathway | 4330 | 47 | 17 | 0.003118 | -0.00906 | 0.977 | 0.020693 | 0.103534 | 1 |
| 27 | Small cell lung cancer | 5222 | 83 | 20 | 0.119724 | 0.863637 | 0.028 | 0.022454 | 0.103534 | 1 |
| 28 | Apoptosis | 4210 | 86 | 25 | 0.010883 | -0.37553 | 0.32 | 0.023194 | 0.103534 | 1 |
| 29 | Dopaminergic synapse | 4728 | 128 | 35 | 0.008516 | 0.324649 | 0.41 | 0.023244 | 0.103534 | 1 |
| 30 | Glutamatergic synapse | 4724 | 119 | 32 | 0.01472 | -0.3932 | 0.243 | 0.023727 | 0.103534 | 1 |
| 31 | Colorectal caner | 5210 | 62 | 15 | 0.157691 | 0.70447 | 0.023 | 0.024008 | 0.103534 | 1 |
| 32 | Amphetamine addiction | 5031 | 69 | 16 | 0.192496 | -1.06199 | 0.02 | 0.025254 | 0.105609 | 1 |
| 33 | Melanoma | 5218 | 71 | 19 | 0.053795 | 0.561123 | 0.089 | 0.030363 | 0.123236 | 1 |
| 34 | Basal cell carcinoma | 5217 | 54 | 12 | 0.286224 | 0.586909 | 0.018 | 0.032295 | 0.124887 | 1 |
| 35 | Tight junction | 4530 | 131 | 36 | 0.007086 | -0.03874 | 0.757 | 0.033409 | 0.124887 | 1 |
| 36 | Legionellosis | 5134 | 51 | 16 | 0.018257 | -0.26961 | 0.3 | 0.033997 | 0.124887 | 1 |
| 37 | Alcoholism | 5034 | 128 | 34 | 0.014728 | 0.357546 | 0.377 | 0.034389 | 0.124887 | 1 |
| 38 | Epithelial cell signaling in Helicobacter pylori infection | 5120 | 67 | 20 | 0.015892 | 0.202969 | 0.368 | 0.035918 | 0.127093 | 1 |
| 39 | Non-small cell lung cancer | 5223 | 54 | 10 | 0.550538 | -0.59343 | 0.011 | 0.036982 | 0.127587 | 1 |
| 40 | Serotonergic synapse | 4726 | 114 | 29 | 0.039319 | -0.37626 | 0.162 | 0.038576 | 0.129841 | 1 |
| 41 | Amyotrophic lateral sclerosis (ALS) | 5014 | 52 | 17 | 0.0099 | -0.10402 | 0.708 | 0.04178 | 0.137278 | 1 |
| 42 | Systemic lupus erythematosus | 5322 | 81 | 18 | 0.227846 | 3.573058 | 0.034 | 0.0454 | 0.145701 | 1 |
| 43 | Melanogenesis | 4916 | 99 | 23 | 0.136908 | 0.563948 | 0.059 | 0.047001 | 0.146464 | 1 |
| 44 | HTLV-I infection | 5166 | 256 | 57 | 0.069206 | 0.744172 | 0.119 | 0.04776 | 0.146464 | 1 |
| 45 | Osteoclast differentiation | 4380 | 129 | 31 | 0.067185 | -0.58227 | 0.138 | 0.05267 | 0.158009 | 1 |
| 46 | Chemokine signaling pathway | 4062 | 179 | 39 | 0.145526 | 1.708552 | 0.077 | 0.061533 | 0.18057 | 1 |
| 47 | Vascular smooth muscle contraction | 4270 | 110 | 30 | 0.0146 | -0.10736 | 0.787 | 0.062807 | 0.18057 | 1 |
| 48 | Mineral absorption | 4978 | 49 | 14 | 0.055637 | -0.05001 | 0.213 | 0.064413 | 0.181129 | 1 |
| 49 | Morphine addiction | 5032 | 89 | 24 | 0.030502 | 0.216903 | 0.401 | 0.066095 | 0.181129 | 1 |
| 50 | Thyroid cancer | 5216 | 29 | 8 | 0.151503 | 0.331452 | 0.082 | 0.066939 | 0.181129 | 1 |
| 51 | Staphylococcus aureus infection | 5150 | 48 | 11 | 0.262556 | 6.697988 | 0.05 | 0.070011 | 0.185798 | 1 |
| 52 | Protein processing in endoplasmic reticulum | 4141 | 162 | 31 | 0.443162 | -0.4144 | 0.032 | 0.074534 | 0.19407 | 1 |
| 53 | NOD-like receptor signaling pathway | 4621 | 57 | 13 | 0.243119 | -0.18068 | 0.067 | 0.083355 | 0.210704 | 1 |
| 54 | Calcium signaling pathway | 4020 | 180 | 42 | 0.0581 | -0.3228 | 0.285 | 0.084462 | 0.210704 | 1 |
| 55 | Cytokine-cytokine receptor interaction | 4060 | 251 | 43 | 0.730989 | 1.438247 | 0.023 | 0.085503 | 0.210704 | 1 |
| 56 | Malaria | 5144 | 47 | 15 | 0.018692 | 0 | 1 | 0.093079 | 0.221638 | 1 |
| 57 | Herpes simplex infection | 5168 | 171 | 36 | 0.215317 | -0.48826 | 0.088 | 0.094096 | 0.221638 | 1 |
| 58 | Long-term depression | 4720 | 69 | 20 | 0.021896 | -0.0378 | 0.873 | 0.094758 | 0.221638 | 1 |
| 59 | T cell receptor signaling pathway | 4660 | 108 | 26 | 0.086141 | -0.36713 | 0.236 | 0.099526 | 0.228909 | 1 |
| 60 | Tuberculos | 5152 | 172 | 41 | 0.045201 | -0.40776 | 0.538 | 0.114698 | 0.25948 | 1 |
| 61 | Retrograde endocannabinoid signaling | 4723 | 100 | 23 | 0.14827 | 0.101727 | 0.17 | 0.117981 | 0.262603 | 1 |
| 62 | Dorso-ventral axis formation | 4320 | 23 | 8 | 0.047469 | 0.037706 | 0.574 | 0.125413 | 0.272406 | 1 |
| 63 | Jak-STAT signaling pathway | 4630 | 153 | 23 | 0.887188 | -0.31315 | 0.031 | 0.126333 | 0.272406 | 1 |
| 64 | Olfactory transduction | 4740 | 112 | 8 | 0.999824 | -2.06496 | 0.029 | 0.131655 | 0.279297 | 1 |
| 65 | Cholinergic synapse | 4725 | 109 | 26 | 0.094073 | 0.257348 | 0.314 | 0.133577 | 0.279297 | 1 |
| 66 | Pathogenic Escherichia coli infection | 5130 | 51 | 13 | 0.133495 | 0.239575 | 0.244 | 0.144111 | 0.296826 | 1 |
| 67 | VEGF signaling pathway | 4370 | 71 | 17 | 0.149175 | -0.4763 | 0.233 | 0.151521 | 0.307498 | 1 |
| 68 | GnRH signaling pathway | 4912 | 94 | 22 | 0.135245 | 0.956699 | 0.262 | 0.153787 | 0.307574 | 1 |
| 69 | Prostate cancer | 5215 | 89 | 22 | 0.085692 | 0.293539 | 0.466 | 0.168537 | 0.332259 | 1 |
| 70 | B cell receptor signaling pathway | 4662 | 75 | 18 | 0.138501 | -0.41906 | 0.315 | 0.180273 | 0.344891 | 1 |
| 71 | Amoebiasis | 5146 | 106 | 23 | 0.226884 | 0.229591 | 0.195 | 0.182193 | 0.344891 | 1 |
| 72 | Arrhythmogenic right ventricular cardiomyopathy (ARVC) | 5412 | 74 | 20 | 0.044411 | 0 | 1 | 0.182718 | 0.344891 | 1 |
| 73 | Progesterone-mediated oocyte maturation | 4914 | 84 | 20 | 0.131182 | 0.155134 | 0.344 | 0.184941 | 0.344891 | 1 |
| 74 | Acute myeloid leukemia | 5221 | 56 | 11 | 0.462932 | 0.256722 | 0.103 | 0.192788 | 0.35241 | 1 |
| 75 | Endometrial cancer | 5213 | 52 | 12 | 0.241615 | 0.240017 | 0.205 | 0.19838 | 0.35241 | 1 |
| 76 | Toxoplasmosis | 5145 | 120 | 29 | 0.070305 | 0.122007 | 0.708 | 0.199115 | 0.35241 | 1 |
| 77 | Glioma | 5214 | 64 | 17 | 0.069651 | 0.095589 | 0.715 | 0.199188 | 0.35241 | 1 |
| 78 | Neurotrophin signaling pathway | 4722 | 119 | 29 | 0.064224 | -0.07298 | 0.814 | 0.20656 | 0.360827 | 1 |
| 79 | Sulfur relay system | 4122 | 9 | 3 | 0.221846 | -0.08263 | 0.245 | 0.21264 | 0.363087 | 1 |
| 80 | Phosphatidylinositol signaling system | 4070 | 79 | 16 | 0.383913 | 0.232671 | 0.142 | 0.213116 | 0.363087 | 1 |
| 81 | Toll-like receptor signaling pathway | 4620 | 98 | 17 | 0.652097 | 0.801899 | 0.09 | 0.225101 | 0.378829 | 1 |
| 82 | Leukocyte transendothelial migration | 4670 | 113 | 26 | 0.130467 | 0.39886 | 0.483 | 0.237214 | 0.390201 | 1 |
| 83 | Pancreatic secretion | 4972 | 91 | 22 | 0.103822 | -0.01258 | 0.608 | 0.237513 | 0.390201 | 1 |
| 84 | Fc epsilon RI signaling pathway | 4664 | 73 | 16 | 0.263634 | -0.72347 | 0.252 | 0.246578 | 0.400326 | 1 |
| 85 | Chronic myeloid leukemia | 5220 | 72 | 17 | 0.163504 | -0.17355 | 0.439 | 0.260854 | 0.41551 | 1 |
| 86 | Hedgehog signaling pathway | 4340 | 54 | 14 | 0.110019 | 0.079904 | 0.663 | 0.263913 | 0.41551 | 1 |
| 87 | Complement and coagulation cascades | 4610 | 67 | 16 | 0.160843 | 2.398263 | 0.456 | 0.264963 | 0.41551 | 1 |
| 88 | Oocyte meiosis | 4114 | 109 | 26 | 0.094073 | 0.021732 | 0.941 | 0.303147 | 0.469057 | 1 |
| 89 | Fc gamma R-mediated phagocytosis | 4666 | 91 | 21 | 0.157583 | -0.28624 | 0.569 | 0.305907 | 0.469057 | 1 |
| 90 | Viral carcinogenesis | 5203 | 182 | 35 | 0.423158 | -0.02358 | 0.217 | 0.311092 | 0.471766 | 1 |
| 91 | Adipocytokine signaling pathway | 4920 | 68 | 14 | 0.371834 | -0.25158 | 0.26 | 0.322551 | 0.482027 | 1 |
| 92 | Viral myocarditis | 5416 | 66 | 14 | 0.32772 | 0.272046 | 0.298 | 0.324844 | 0.482027 | 1 |
| 93 | Vibrio cholerae infection | 5110 | 53 | 8 | 0.786927 | -0.33724 | 0.135 | 0.344425 | 0.505396 | 1 |
| 94 | Pancreatic cancer | 5212 | 69 | 16 | 0.192496 | -0.15125 | 0.56 | 0.347918 | 0.505396 | 1 |
| 95 | Salivary secretion | 4970 | 82 | 15 | 0.560183 | 0.065942 | 0.199 | 0.356049 | 0.508581 | 1 |
| 96 | mTOR signaling pathway | 4150 | 62 | 14 | 0.244116 | -0.16998 | 0.463 | 0.359438 | 0.508581 | 1 |
| 97 | Regulation of autophagy | 4140 | 33 | 6 | 0.5867 | -0.36772 | 0.194 | 0.361166 | 0.508581 | 1 |
| 98 | GABAergic synapse | 4727 | 87 | 20 | 0.169228 | 0.059844 | 0.714 | 0.376187 | 0.520253 | 1 |
| 99 | Endocrine and other factor-regulated calcium reabsorption | 4961 | 49 | 12 | 0.180375 | -0.06171 | 0.672 | 0.376995 | 0.520253 | 1 |
| 100 | Type II diabetes mellitus | 4930 | 47 | 6 | 0.888412 | -0.08908 | 0.144 | 0.390992 | 0.534226 | 1 |
| 101 | SNARE interactions in vesicular transport | 4130 | 35 | 9 | 0.183805 | 0.024618 | 0.718 | 0.399237 | 0.540144 | 1 |
| 102 | Hepatitis C | 5160 | 128 | 24 | 0.501754 | -0.26898 | 0.317 | 0.45148 | 0.604896 | 1 |
| 103 | Long-term depression | 4730 | 65 | 14 | 0.306129 | 0.364313 | 0.536 | 0.460648 | 0.611244 | 1 |
| 104 | Chagas disease (American trypanosomiasis) | 5142 | 101 | 21 | 0.309065 | 0.189807 | 0.54 | 0.465703 | 0.612066 | 1 |
| 105 | Taste transduction | 4742 | 45 | 5 | 0.936645 | 0.148006 | 0.184 | 0.475368 | 0.618875 | 1 |
| 106 | p53 signaling pathway | 4115 | 67 | 13 | 0.469882 | -0.08135 | 0.407 | 0.507598 | 0.650634 | 1 |
| 107 | Gastric acid secretion | 4971 | 73 | 15 | 0.36742 | -0.12864 | 0.524 | 0.509721 | 0.650634 | 1 |
| 108 | Cocaine addiction | 5030 | 50 | 10 | 0.445386 | -0.13086 | 0.438 | 0.513906 | 0.650634 | 1 |
| 109 | Shigellosis | 5131 | 60 | 14 | 0.205772 | -0.00402 | 0.966 | 0.519914 | 0.652255 | 1 |
| 110 | Bladder cancer | 5219 | 41 | 8 | 0.493594 | -0.10696 | 0.423 | 0.535845 | 0.666185 | 1 |
| 111 | Aldosterone-regulated sodium reabsorption | 4960 | 39 | 6 | 0.751089 | -0.08962 | 0.29 | 0.54979 | 0.67742 | 1 |
| 112 | Cytosolic DNA-sensing pathway | 4623 | 59 | 9 | 0.785274 | 0.06111 | 0.33 | 0.60908 | 0.743832 | 1 |
| 113 | Intestinal immune network for IgA production | 4672 | 44 | 9 | 0.425609 | 0.009659 | 0.687 | 0.651937 | 0.789187 | 1 |
| 114 | Cell cycle | 4110 | 122 | 16 | 0.954741 | -0.29862 | 0.327 | 0.675636 | 0.810763 | 1 |
| 115 | Antigen processing and presentation | 4612 | 63 | 4 | 0.998512 | -0.15233 | 0.344 | 0.710541 | 0.845298 | 1 |
| 116 | Vasopressin-regulated water reabsorption | 4962 | 44 | 9 | 0.425609 | -0.01558 | 0.826 | 0.719065 | 0.848128 | 1 |
| 117 | Carbohydrate digestion and absorption | 4973 | 38 | 4 | 0.938582 | 0.002921 | 0.382 | 0.726298 | 0.8494 | 1 |
| 118 | Natural killer cell mediated cytotoxicity | 4650 | 127 | 23 | 0.576281 | 0.474984 | 0.688 | 0.763276 | 0.882104 | 1 |
| 119 | African trypanosomiasis | 5143 | 32 | 4 | 0.867054 | -0.01258 | 0.462 | 0.767047 | 0.882104 | 1 |
| 120 | Measles | 5162 | 128 | 17 | 0.953097 | -0.14868 | 0.471 | 0.808456 | 0.915541 | 1 |
| 121 | Epstein-Barr virus infection | 5169 | 189 | 35 | 0.520924 | -0.06358 | 0.864 | 0.809391 | 0.915541 | 1 |
| 122 | Leishmaniasis | 5140 | 66 | 12 | 0.572951 | -0.00291 | 0.981 | 0.885892 | 0.993927 | 1 |
| 123 | Neuroactive ligand-receptor interaction | 4080 | 264 | 32 | 0.998022 | -0.02278 | 0.587 | 0.899093 | 1 | 1 |
| 124 | RIG-I-like receptor signaling pathway | 4622 | 70 | 10 | 0.855657 | -0.02048 | 0.762 | 0.930872 | 1 | 1 |
| 125 | NF-kappa B signaling pathway | 4064 | 88 | 13 | 0.849312 | 0.056866 | 0.803 | 0.943018 | 1 | 1 |
| 126 | Rheumatoid arthritis | 5323 | 83 | 14 | 0.689 | 0 | 1 | 0.945662 | 1 | 1 |
| 127 | TGF-beta signaling pathway | 4350 | 81 | 13 | 0.754038 | -0.00279 | 0.987 | 0.964081 | 1 | 1 |
| 128 | Phototransduction | 4744 | 28 | 3 | 0.911946 | 0.004957 | 0.864 | 0.975728 | 1 | 1 |
| 129 | Maturity onset diabetes of the young | 4950 | 23 | 3 | 0.82455 | 0 | 1 | 0.98362 | 1 | 1 |
| 130 | Type I diabetes mellitus | 4940 | 39 | 5 | 0.870825 | 0 | 1 | 0.991273 | 1 | 1 |
| 131 | Allograft rejection | 5330 | 33 | 3 | 0.957817 | 0 | 1 | 0.999098 | 1 | 1 |
| 132 | Graft-versus-host disease | 5332 | 34 | 3 | 0.963755 | 0 | 1 | 0.999335 | 1 | 1 |
| 133 | Asthma | 5310 | 26 | 2 | 0.965877 | 0 | 1 | 0.999411 | 1 | 1 |
| 134 | RNA degradation | 3018 | 69 | 7 | 0.980309 | 0 | 1 | 0.999805 | 1 | 1 |
| 135 | RNA transport | 3013 | 145 | 16 | 0.994735 | 0 | 1 | 0.999986 | 1 | 1 |
| 136 | Autoimmune thyroid disease | 5320 | 47 | 3 | 0.995446 | 0 | 1 | 0.99999 | 1 | 1 |
| 137 | Fanconi anemia pathway | 3460 | 48 | 1 | 0.999945 | 0 | 1 | 1 | 1 | 1 |

Result obtained by the SPIA methods in the colon cancer dataset

| No | Name | ID | pSize | NDE | pNDE | tA | pPERT | pG | pGFdr | pGFWER |
| --- | --- | --- | --- | --- | --- | --- | --- | --- | --- | --- |
| 1 | Focal adhesion | 4510 | 199 | 68 | 8.54E-08 | 104.012 | 5.00E-06 | 1.26E-11 | 1.72E-09 | 1.72E-09 |
| 2 | Parkinson's disease | 5012 | 116 | 53 | 1.53E-11 | -10.1325 | 0.102 | 4.41E-11 | 3.02E-09 | 6.04E-09 |
| 3 | Alzheimer's disease | 5010 | 159 | 66 | 1.15E-11 | -6.29843 | 0.247 | 7.83E-11 | 3.57E-09 | 1.07E-08 |
| 4 | ECM-receptor interaction | 4512 | 83 | 27 | 0.00148 | 21.96804 | 5.00E-06 | 1.46E-07 | 5.00E-06 | 2.00E-05 |
| 5 | Huntington's disease | 5016 | 171 | 60 | 1.69E-07 | -3.11527 | 0.221 | 6.76E-07 | 1.85E-05 | 9.26E-05 |
| 6 | PPAR signaling pathway | 3320 | 69 | 30 | 1.41E-06 | -3.11107 | 0.057 | 1.39E-06 | 3.18E-05 | 0.000191 |
| 7 | Axon guidance | 4360 | 128 | 46 | 2.10E-06 | 9.203733 | 0.225 | 7.35E-06 | 0.000144 | 0.001007 |
| 8 | Pathways in cancer | 5200 | 321 | 84 | 0.000366 | 66.66049 | 0.002 | 1.11E-05 | 0.00019 | 0.001516 |
| 9 | MAPK signaling pathway | 4010 | 259 | 76 | 1.23E-05 | 8.855113 | 0.282 | 4.69E-05 | 0.000714 | 0.006428 |
| 10 | Glutamatergic synapse | 4724 | 119 | 32 | 0.01472 | -11.1203 | 0.006 | 0.000913 | 0.012505 | 0.125048 |
| 11 | Wnt signaling pathway | 4310 | 149 | 44 | 0.000662 | -8.19114 | 0.215 | 0.001402 | 0.017463 | 0.192098 |
| 12 | Small cell lung cancer | 5222 | 83 | 20 | 0.119724 | 25.86056 | 0.002 | 0.002236 | 0.025525 | 0.3063 |
| 13 | Lysosome | 4142 | 117 | 35 | 0.001748 | -0.75311 | 0.179 | 0.002837 | 0.029902 | 0.388727 |
| 14 | Regulation of actin cytoskeleton | 4810 | 212 | 57 | 0.001524 | 14.26457 | 0.26 | 0.0035 | 0.034249 | 0.479487 |
| 15 | Renal cell carcinoma | 5211 | 69 | 22 | 0.005103 | -7.02221 | 0.162 | 0.006695 | 0.061147 | 0.917204 |
| 16 | Transcriptional misregulation in cancer | 5202 | 158 | 42 | 0.007253 | -1.1845 | 0.131 | 0.007562 | 0.062583 | 1 |
| 17 | Apoptosis | 4210 | 86 | 25 | 0.010883 | -14.6193 | 0.09 | 0.007766 | 0.062583 | 1 |
| 18 | Melanoma | 5218 | 71 | 19 | 0.053795 | 29.46815 | 0.04 | 0.015367 | 0.110142 | 1 |
| 19 | Circadian rhythm | 4710 | 21 | 9 | 0.008468 | -4.2398 | 0.257 | 0.015518 | 0.110142 | 1 |
| 20 | Morphine addiction | 5032 | 89 | 24 | 0.030502 | 6.119395 | 0.081 | 0.017303 | 0.110142 | 1 |
| 21 | Salmonella infection | 5132 | 80 | 25 | 0.003979 | -2.5529 | 0.634 | 0.017614 | 0.110142 | 1 |
| 22 | Prion diseases | 5020 | 34 | 12 | 0.014995 | 6.273047 | 0.175 | 0.018219 | 0.110142 | 1 |
| 23 | Pathogenic Escherichia coli infection | 5130 | 51 | 13 | 0.133495 | 17.10412 | 0.02 | 0.018491 | 0.110142 | 1 |
| 24 | Pertussis | 5133 | 69 | 22 | 0.005103 | -3.58828 | 0.597 | 0.020698 | 0.114091 | 1 |
| 25 | Notch signaling pathway | 4330 | 47 | 17 | 0.003118 | 0.196202 | 0.984 | 0.02082 | 0.114091 | 1 |
| 26 | Insulin signaling pathway | 4910 | 136 | 37 | 0.007609 | -12.8355 | 0.466 | 0.023551 | 0.124096 | 1 |
| 27 | Colorectal cancer | 5210 | 62 | 15 | 0.157691 | 7.089884 | 0.029 | 0.029211 | 0.145179 | 1 |
| 28 | Sulfur relay system | 4122 | 9 | 3 | 0.221846 | -3.43081 | 0.021 | 0.029672 | 0.145179 | 1 |
| 29 | Amyotrophic lateral sclerosis (ALS) | 5014 | 52 | 17 | 0.0099 | -2.94064 | 0.518 | 0.03217 | 0.151978 | 1 |
| 30 | Gap junction | 4540 | 85 | 24 | 0.017829 | 9.006783 | 0.305 | 0.033793 | 0.154323 | 1 |
| 31 | ErbB signaling pathway | 4012 | 87 | 25 | 0.012657 | -7.75691 | 0.51 | 0.039008 | 0.16953 | 1 |
| 32 | Legionellosis | 5134 | 51 | 16 | 0.018257 | -3.84168 | 0.36 | 0.039598 | 0.16953 | 1 |
| 33 | Tight junction | 4530 | 131 | 36 | 0.007086 | -0.10101 | 0.986 | 0.041668 | 0.172987 | 1 |
| 34 | Dopaminergic synapse | 4728 | 128 | 35 | 0.008516 | -0.12707 | 0.972 | 0.04796 | 0.187776 | 1 |
| 35 | Epithelial cell signaling in Helicobacter pylori infection | 5120 | 67 | 20 | 0.015892 | -2.83839 | 0.521 | 0.047972 | 0.187776 | 1 |
| 36 | Alcoholism | 5034 | 128 | 34 | 0.014728 | -5.56229 | 0.606 | 0.051042 | 0.194242 | 1 |
| 37 | Long-term potentiation | 4720 | 69 | 20 | 0.021896 | 9.265449 | 0.423 | 0.052625 | 0.194853 | 1 |
| 38 | Dilated cardiomyopathy | 5414 | 90 | 22 | 0.094476 | -2.57333 | 0.118 | 0.061276 | 0.217104 | 1 |
| 39 | Serotonergic synapse | 4726 | 114 | 29 | 0.039319 | -4.41664 | 0.29 | 0.062416 | 0.217104 | 1 |
| 40 | Systemic lupus erythematosus | 5322 | 81 | 18 | 0.227846 | 5.433914 | 0.051 | 0.063388 | 0.217104 | 1 |
| 41 | Mineral absorption | 4978 | 49 | 14 | 0.055637 | -0.65974 | 0.217 | 0.065398 | 0.218525 | 1 |
| 42 | Vascular smooth muscle contraction | 4270 | 110 | 30 | 0.0146 | 1.560797 | 0.87 | 0.068157 | 0.222323 | 1 |
| 43 | Hepatitis C | 5160 | 128 | 24 | 0.501754 | -10.6179 | 0.027 | 0.071822 | 0.225991 | 1 |
| 44 | VEGF signaling pathway | 4370 | 71 | 17 | 0.149175 | -10.7883 | 0.092 | 0.072581 | 0.225991 | 1 |
| 45 | Chemokine signaling pathway | 4062 | 179 | 39 | 0.145526 | 21.77002 | 0.1 | 0.07611 | 0.231713 | 1 |
| 46 | HTLV-I infection | 5166 | 256 | 57 | 0.069206 | 10.60509 | 0.229 | 0.081534 | 0.24283 | 1 |
| 47 | Cholinergic synapse | 4725 | 109 | 26 | 0.094073 | 11.41878 | 0.183 | 0.087144 | 0.254014 | 1 |
| 48 | GABAergic synapse | 4727 | 87 | 20 | 0.169228 | -3.07989 | 0.108 | 0.091422 | 0.260241 | 1 |
| 49 | Malaria | 5144 | 47 | 15 | 0.018692 | 0 | 1 | 0.093079 | 0.260241 | 1 |
| 50 | B cell receptor signaling pathway | 4662 | 75 | 18 | 0.138501 | -10.1226 | 0.144 | 0.098022 | 0.264038 | 1 |
| 51 | Fc gamma R-mediated phagocytosis | 4666 | 91 | 21 | 0.157583 | -11.0723 | 0.127 | 0.098291 | 0.264038 | 1 |
| 52 | Staphylococcus aureus infection | 5150 | 48 | 11 | 0.262556 | 10.91476 | 0.079 | 0.101129 | 0.264896 | 1 |
| 53 | Fc epsilon RI signaling pathway | 4664 | 73 | 16 | 0.263634 | -15.1384 | 0.08 | 0.102478 | 0.264896 | 1 |
| 54 | Dorso-ventral axis formation | 4320 | 23 | 8 | 0.047469 | 0.65995 | 0.477 | 0.108411 | 0.275044 | 1 |
| 55 | T cell receptor signaling pathway | 4660 | 108 | 26 | 0.086141 | -7.99405 | 0.292 | 0.117786 | 0.293395 | 1 |
| 56 | Glioma | 5214 | 64 | 17 | 0.069651 | 8.321869 | 0.383 | 0.123351 | 0.299971 | 1 |
| 57 | Prostate cancer | 5215 | 89 | 22 | 0.085692 | 8.906677 | 0.316 | 0.124805 | 0.299971 | 1 |
| 58 | Influenza A | 5164 | 160 | 38 | 0.054748 | -3.42505 | 0.516 | 0.129008 | 0.304727 | 1 |
| 59 | Herpes simplex infection | 5168 | 171 | 36 | 0.215317 | -9.10011 | 0.146 | 0.140199 | 0.325547 | 1 |
| 60 | Tuberculosis | 5152 | 172 | 41 | 0.045201 | -4.14445 | 0.713 | 0.14293 | 0.326357 | 1 |
| 61 | Type II diabetes mellitus | 4930 | 47 | 6 | 0.888412 | -7.12584 | 0.043 | 0.162926 | 0.365229 | 1 |
| 62 | Calcium signaling pathway | 4020 | 180 | 42 | 0.0581 | 3.228448 | 0.67 | 0.165286 | 0.365229 | 1 |
| 63 | Toxoplasmosis | 5145 | 120 | 29 | 0.070305 | -3.47992 | 0.575 | 0.170122 | 0.367627 | 1 |
| 64 | Basal cell carcinoma | 5217 | 54 | 12 | 0.286224 | 11.52851 | 0.143 | 0.171738 | 0.367627 | 1 |
| 65 | Arrhythmogenic right ventricular cardiomyopathy (ARVC) | 5412 | 74 | 20 | 0.044411 | 0 | 1 | 0.182718 | 0.385112 | 1 |
| 66 | Phosphatidylinositol signaling system | 4070 | 79 | 16 | 0.383913 | 1.317313 | 0.13 | 0.199513 | 0.410277 | 1 |
| 67 | Neurotrophin signaling pathway | 4722 | 119 | 29 | 0.064224 | -2.70159 | 0.783 | 0.200646 | 0.410277 | 1 |
| 68 | Osteoclast differentiation | 4380 | 129 | 31 | 0.067185 | 1.602033 | 0.815 | 0.213814 | 0.430773 | 1 |
| 69 | Non-small cell lung cancer | 5223 | 54 | 10 | 0.550538 | -11.7019 | 0.11 | 0.230375 | 0.457411 | 1 |
| 70 | Leukocyte transendothelial migration | 4670 | 113 | 26 | 0.130467 | 6.640185 | 0.488 | 0.239014 | 0.46552 | 1 |
| 71 | Bacterial invasion of epithelial cells | 5100 | 70 | 18 | 0.082565 | 2.776205 | 0.781 | 0.241255 | 0.46552 | 1 |
| 72 | Hedgehog signaling pathway | 4340 | 54 | 14 | 0.110019 | -2.21908 | 0.626 | 0.253139 | 0.481668 | 1 |
| 73 | Bile secretion | 4976 | 71 | 13 | 0.560952 | -2.26391 | 0.129 | 0.262392 | 0.48968 | 1 |
| 74 | Pancreatic secretion | 4972 | 91 | 22 | 0.103822 | -0.53684 | 0.715 | 0.267279 | 0.48968 | 1 |
| 75 | Amoebiasis | 5146 | 106 | 23 | 0.226884 | 2.899809 | 0.332 | 0.270112 | 0.48968 | 1 |
| 76 | Amphetamine addiction | 5031 | 69 | 16 | 0.192496 | -3.2277 | 0.396 | 0.272442 | 0.48968 | 1 |
| 77 | Cytokine-cytokine receptor interaction | 4060 | 251 | 43 | 0.730989 | 8.150393 | 0.107 | 0.277532 | 0.48968 | 1 |
| 78 | GnRH signaling pathway | 4912 | 94 | 22 | 0.135245 | 5.843162 | 0.582 | 0.278796 | 0.48968 | 1 |
| 79 | Oocyte meiosis | 4114 | 109 | 26 | 0.094073 | 1.142553 | 0.889 | 0.291149 | 0.504903 | 1 |
| 80 | Cocaine addiction | 5030 | 50 | 10 | 0.445386 | 4.043554 | 0.192 | 0.295799 | 0.506557 | 1 |
| 81 | Complement and coagulation cascades | 4610 | 67 | 16 | 0.160843 | 5.648817 | 0.555 | 0.304949 | 0.509505 | 1 |
| 82 | Melanogenesis | 4916 | 99 | 23 | 0.136908 | 7.090199 | 0.66 | 0.307579 | 0.509505 | 1 |
| 83 | Protein processing in endoplasmic reticulum | 4141 | 162 | 31 | 0.443162 | -3.98583 | 0.207 | 0.310875 | 0.509505 | 1 |
| 84 | Retrograde endocannabinoid signaling | 4723 | 100 | 23 | 0.14827 | 0.271852 | 0.623 | 0.312397 | 0.509505 | 1 |
| 85 | Endocrine and other factor-regulated calcium reabsorption | 4961 | 49 | 12 | 0.180375 | 2.032094 | 0.547 | 0.327176 | 0.521623 | 1 |
| 86 | Thyroid cancer | 5216 | 29 | 8 | 0.151503 | -1.35006 | 0.652 | 0.327442 | 0.521623 | 1 |
| 87 | Progesterone-mediated oocyte maturation | 4914 | 84 | 20 | 0.131182 | 0.896444 | 0.811 | 0.344768 | 0.540676 | 1 |
| 88 | Endometrial cancer | 5213 | 52 | 12 | 0.241615 | -4.8972 | 0.445 | 0.347295 | 0.540676 | 1 |
| 89 | Chronic myeloid leukemia | 5220 | 72 | 17 | 0.163504 | -1.70727 | 0.736 | 0.375149 | 0.577477 | 1 |
| 90 | p53 signaling pathway | 4115 | 67 | 13 | 0.469882 | -2.71118 | 0.267 | 0.385883 | 0.5874 | 1 |
| 91 | Jak-STAT signaling pathway | 4630 | 153 | 23 | 0.887188 | -3.17874 | 0.144 | 0.390629 | 0.58809 | 1 |
| 92 | SNARE interactions in vesicular transport | 4130 | 35 | 9 | 0.183805 | 0.298922 | 0.769 | 0.417896 | 0.622301 | 1 |
| 93 | Carbohydrate digestion and absorption | 4973 | 38 | 4 | 0.938582 | 1.140092 | 0.167 | 0.447211 | 0.657643 | 1 |
| 94 | mTOR signaling pathway | 4150 | 62 | 14 | 0.244116 | 0.925759 | 0.651 | 0.45123 | 0.657643 | 1 |
| 95 | Vibrio cholerae infection | 5110 | 53 | 8 | 0.786927 | -1.57543 | 0.206 | 0.457061 | 0.659129 | 1 |
| 96 | Pancreatic cancer | 5212 | 69 | 16 | 0.192496 | 0.851459 | 0.862 | 0.463975 | 0.662131 | 1 |
| 97 | Bladder cancer | 5219 | 41 | 8 | 0.493594 | -3.58218 | 0.35 | 0.476098 | 0.672426 | 1 |
| 98 | Shigellosis | 5131 | 60 | 14 | 0.205772 | 0.276836 | 0.948 | 0.513895 | 0.718404 | 1 |
| 99 | Epstein-Barr virus infection | 5169 | 189 | 35 | 0.520924 | -3.66811 | 0.382 | 0.520265 | 0.719028 | 1 |
| 100 | Acute myeloid leukemia | 5221 | 56 | 11 | 0.462932 | 2.64973 | 0.436 | 0.524838 | 0.719028 | 1 |
| 101 | Long-term depression | 4730 | 65 | 14 | 0.306129 | 2.330176 | 0.693 | 0.541077 | 0.733936 | 1 |
| 102 | NOD-like receptor signaling pathway | 4621 | 57 | 13 | 0.243119 | 0.25899 | 0.94 | 0.565863 | 0.760032 | 1 |
| 103 | Viral carcinogenesis | 5203 | 182 | 35 | 0.423158 | 0.337071 | 0.593 | 0.597865 | 0.795219 | 1 |
| 104 | Intestinal immune network for IgA production | 4672 | 44 | 9 | 0.425609 | 0.322199 | 0.62 | 0.615434 | 0.810716 | 1 |
| 105 | Chagas disease (American trypanosomiasis) | 5142 | 101 | 21 | 0.309065 | -0.70767 | 0.928 | 0.64502 | 0.834564 | 1 |
| 106 | Salivary secretion | 4970 | 82 | 15 | 0.560183 | 1.642035 | 0.513 | 0.645721 | 0.834564 | 1 |
| 107 | Gastric acid secretion | 4971 | 73 | 15 | 0.36742 | -0.58264 | 0.814 | 0.660083 | 0.838841 | 1 |
| 108 | Adipocytokine signaling pathway | 4920 | 68 | 14 | 0.371834 | 0.769394 | 0.807 | 0.661276 | 0.838841 | 1 |
| 109 | Viral myocarditis | 5416 | 66 | 14 | 0.32772 | -0.08511 | 0.97 | 0.682206 | 0.857452 | 1 |
| 110 | NF-kappa B signaling pathway | 4064 | 88 | 13 | 0.849312 | 3.824123 | 0.406 | 0.711962 | 0.886716 | 1 |
| 111 | Regulation of autophagy | 4140 | 33 | 6 | 0.5867 | -0.67767 | 0.688 | 0.769844 | 0.945484 | 1 |
| 112 | Aldosterone-regulated sodium reabsorption | 4960 | 39 | 6 | 0.751089 | 0.605945 | 0.542 | 0.77295 | 0.945484 | 1 |
| 113 | Vasopressin-regulated water reabsorption | 4962 | 44 | 9 | 0.425609 | -0.0587 | 0.982 | 0.782566 | 0.948774 | 1 |
| 114 | RIG-I-like receptor signaling pathway | 4622 | 70 | 10 | 0.855657 | -1.8864 | 0.51 | 0.798249 | 0.959299 | 1 |
| 115 | African trypanosomiasis | 5143 | 32 | 4 | 0.867054 | -0.53684 | 0.522 | 0.811399 | 0.966623 | 1 |
| 116 | Phototransduction | 4744 | 28 | 3 | 0.911946 | 1.362703 | 0.507 | 0.819027 | 0.967299 | 1 |
| 117 | Natural killer cell mediated cytotoxicity | 4650 | 127 | 23 | 0.576281 | 2.879914 | 0.865 | 0.84552 | 0.984873 | 1 |
| 118 | Leishmaniasis | 5140 | 66 | 12 | 0.572951 | 0.499531 | 0.877 | 0.848285 | 0.984873 | 1 |
| 119 | Antigen processing and presentation | 4612 | 63 | 4 | 0.998512 | 0.133266 | 0.559 | 0.883633 | 1 | 1 |
| 120 | Toll-like receptor signaling pathway | 4620 | 98 | 17 | 0.652097 | -0.89797 | 0.88 | 0.892556 | 1 | 1 |
| 121 | Neuroactive ligand-receptor interaction | 4080 | 264 | 32 | 0.998022 | -0.79104 | 0.58 | 0.895316 | 1 | 1 |
| 122 | TGF-beta signaling pathway | 4350 | 81 | 13 | 0.754038 | 1.340467 | 0.771 | 0.896683 | 1 | 1 |
| 123 | Cytosolic DNA-sensing pathway | 4623 | 59 | 9 | 0.785274 | -0.3098 | 0.757 | 0.903636 | 1 | 1 |
| 124 | Measles | 5162 | 128 | 17 | 0.953097 | -1.78072 | 0.662 | 0.921521 | 1 | 1 |
| 125 | Taste transduction | 4742 | 45 | 5 | 0.936645 | 1.131916 | 0.698 | 0.931625 | 1 | 1 |
| 126 | Rheumatoid arthritis | 5323 | 83 | 14 | 0.689 | 0 | 1 | 0.945662 | 1 | 1 |
| 127 | Maturity onset diabetes of the young | 4950 | 23 | 3 | 0.82455 | 0 | 1 | 0.98362 | 1 | 1 |
| 128 | Cell cycle | 4110 | 122 | 16 | 0.954741 | 0.851828 | 0.889 | 0.98794 | 1 | 1 |
| 129 | Type I diabetes mellitus | 4940 | 39 | 5 | 0.870825 | 0 | 1 | 0.991273 | 1 | 1 |
| 130 | Allograft rejection | 5330 | 33 | 3 | 0.957817 | 0 | 1 | 0.999098 | 1 | 1 |
| 131 | Graft-versus-host disease | 5332 | 34 | 3 | 0.963755 | 0 | 1 | 0.999335 | 1 | 1 |
| 132 | Olfactory transduction | 4740 | 112 | 8 | 0.999824 | -0.25652 | 0.964 | 0.999338 | 1 | 1 |
| 133 | Asthma | 5310 | 26 | 2 | 0.965877 | 0 | 1 | 0.999411 | 1 | 1 |
| 134 | RNA degradation | 3018 | 69 | 7 | 0.980309 | 0 | 1 | 0.999805 | 1 | 1 |
| 135 | RNA transport | 3013 | 145 | 16 | 0.994735 | 0 | 1 | 0.999986 | 1 | 1 |
| 136 | Autoimmune thyroid disease | 5320 | 47 | 3 | 0.995446 | 0 | 1 | 0.99999 | 1 | 1 |
| 137 | Fanconi anemia pathway | 3460 | 48 | 1 | 0.999945 | 0 | 1 | 1 | 1 | 1 |

Result obtained by the BPA methods in the colon cancer dataset

| No | ID and Name | Score | p-value | FDR | FWER |
| --- | --- | --- | --- | --- | --- |
| 1 | hsa00071 Fatty acid metabolism - Homo sapiens (human) | -1232.56 | 0.001 | 0.04775 | 0.104712 |
| 2 | hsa00280 Valine, leucine and isoleucine degradation - Homo sapiens (human) | -1828.51 | 0.001 | 0.04775 | 0.052356 |
| 3 | hsa00590 Arachidonic acid metabolism - Homo sapiens (human) | -1986.77 | 0.001 | 0.04775 | 0.314136 |
| 4 | hsa04360 Axon guidance - Homo sapiens (human) | -4554.07 | 0.001 | 0.04775 | 0.455497 |
| 5 | hsa04662 B cell receptor signaling pathway - Homo sapiens (human) | -2628.38 | 0.002 | 0.0764 | 0.52356 |
| 6 | hsa00251 Glutamate metabolism - Homo sapiens (human) | -1322.97 | 0.003 | 0.081857 | 0.235602 |
| 7 | hsa00561 Glycerolipid metabolism - Homo sapiens (human) | -1357.8 | 0.003 | 0.081857 | 0.502618 |
| 8 | hsa00290 Valine, leucine and isoleucine biosynthesis - Homo sapiens (human) | -351.362 | 0.004 | 0.0955 | 0.115183 |
| 9 | hsa00601 Glycosphingolipid biosynthesis - lacto and neolacto series - Homo sapiens (human) | -1367.92 | 0.005 | 0.0955 | 0.455497 |
| 10 | hsa03320 PPAR signaling pathway - Homo sapiens (human) | -3375.29 | 0.005 | 0.0955 | 0.183246 |
| 11 | hsa04010 MAPK signaling pathway - Homo sapiens (human) | -9276.19 | 0.006 | 0.102846 | 0.78534 |
| 12 | hsa04520 Adherens junction - Homo sapiens (human) | -3838.28 | 0.007 | 0.102846 | 0.816754 |
| 13 | hsa04510 Focal adhesion - Homo sapiens (human) | -4728.15 | 0.007 | 0.102846 | 0.649215 |
| 14 | hsa04670 Leukocyte transendothelial migration - Homo sapiens (human) | -1645.1 | 0.01 | 0.136429 | 0.539267 |
| 15 | hsa00480 Glutathione metabolism - Homo sapiens (human) | -1283.75 | 0.012 | 0.1528 | 0.371728 |
| 16 | hsa04650 Natural killer cell mediated cytotoxicity - Homo sapiens (human) | -5144.1 | 0.013 | 0.155188 | 0.795812 |
| 17 | hsa04910 Insulin signaling pathway - Homo sapiens (human) | -4990.41 | 0.014 | 0.157294 | 0.963351 |
| 18 | hsa05120 Epithelial cell signaling in Helicobacter pylori infection - Homo sapiens (human) | -2611.02 | 0.017 | 0.170895 | 0.874346 |
| 19 | hsa05212 Pancreatic cancer - Homo sapiens (human) | -3135.86 | 0.017 | 0.170895 | 0.926702 |
| 20 | hsa00531 Glycosaminoglycan degradation - Homo sapiens (human) | -1118.29 | 0.019 | 0.17281 | 0.685864 |
| 21 | hsa05131 Pathogenic Escherichia coli infection - EPEC - Homo sapiens (human) | -1814.01 | 0.019 | 0.17281 | 0.979058 |
| 22 | hsa00252 Alanine and aspartate metabolism - Homo sapiens (human) | -1470.22 | 0.025 | 0.215913 | 0.554974 |
| 23 | hsa05014 Amyotrophic lateral sclerosis (ALS) - Homo sapiens (human) | -2388.23 | 0.026 | 0.215913 | 0.39267 |
| 24 | hsa04610 Complement and coagulation cascades - Homo sapiens (human) | -4619.18 | 0.028 | 0.216132 | 0.994764 |
| 25 | hsa04530 Tight junction - Homo sapiens (human) | -4154.86 | 0.03 | 0.216132 | 0.979058 |
| 26 | hsa04070 Phosphatidylinositol signaling system - Homo sapiens (human) | -1601.16 | 0.031 | 0.216132 | 0.549738 |
| 27 | hsa00970 Aminoacyl-tRNA biosynthesis - Homo sapiens (human) | -1940.56 | 0.031 | 0.216132 | 0.994764 |
| 28 | hsa04912 GnRH signaling pathway - Homo sapiens (human) | -3252.54 | 0.032 | 0.216132 | 0.91623 |
| 29 | hsa04630 Jak-STAT signaling pathway - Homo sapiens (human) | -2152.6 | 0.034 | 0.216132 | 0.979058 |
| 30 | hsa04330 Notch signaling pathway - Homo sapiens (human) | -1751.84 | 0.036 | 0.216132 | 0.979058 |
| 31 | hsa04540 Gap junction - Homo sapiens (human) | -2842.88 | 0.038 | 0.216132 | 0.994764 |
| 32 | hsa05210 Colorectal cancer - Homo sapiens (human) | -3316.19 | 0.039 | 0.216132 | 0.984293 |
| 33 | hsa00020 Citrate cycle (TCA cycle) - Homo sapiens (human) | -1130.76 | 0.041 | 0.216132 | 0.439791 |
| 34 | hsa00340 Histidine metabolism - Homo sapiens (human) | -1173.32 | 0.041 | 0.216132 | 0.994764 |
| 35 | hsa00260 Glycine, serine and threonine metabolism - Homo sapiens (human) | -2366.97 | 0.041 | 0.216132 | 1 |
| 36 | hsa04310 Wnt signaling pathway - Homo sapiens (human) | -5061.69 | 0.041 | 0.216132 | 0.994764 |
| 37 | hsa00072 Synthesis and degradation of ketone bodies - Homo sapiens (human) | -345.952 | 0.042 | 0.216132 | 0.240838 |
| 38 | hsa04512 ECM-receptor interaction - Homo sapiens (human) | -3553.29 | 0.043 | 0.216132 | 0.95288 |
| 39 | hsa05040 Huntington's disease - Homo sapiens (human) | -1783.55 | 0.049 | 0.230977 | 1 |
| 40 | hsa00100 Biosynthesis of steroids - Homo sapiens (human) | -1722.84 | 0.05 | 0.230977 | 0.753927 |
| 41 | hsa00051 Fructose and mannose metabolism - Homo sapiens (human) | -317.521 | 0.051 | 0.230977 | 0.539267 |
| 42 | hsa00272 Cysteine metabolism - Homo sapiens (human) | -497.435 | 0.051 | 0.230977 | 0.356021 |
| 43 | hsa05330 Allograft rejection - Homo sapiens (human) | -1427.61 | 0.052 | 0.230977 | 0.994764 |
| 44 | hsa04930 Type II diabetes mellitus - Homo sapiens (human) | -1442.69 | 0.054 | 0.233444 | 0.696335 |
| 45 | hsa03022 Basal transcription factors - Homo sapiens (human) | -1951.12 | 0.055 | 0.233444 | 1 |
| 46 | hsa05221 Acute myeloid leukemia - Homo sapiens (human) | -2668.33 | 0.057 | 0.236674 | 0.979058 |
| 47 | hsa00512 O-Glycan biosynthesis - Homo sapiens (human) | -585.678 | 0.059 | 0.237776 | 0.460733 |
| 48 | hsa00760 Nicotinate and nicotinamide metabolism - Homo sapiens (human) | -1017.38 | 0.06 | 0.237776 | 0.387435 |
| 49 | hsa05010 Alzheimer's disease - Homo sapiens (human) | -4261.32 | 0.061 | 0.237776 | 0.973822 |
| 50 | hsa00643 Styrene degradation - Homo sapiens (human) | -239.459 | 0.063 | 0.24066 | 0.649215 |
| 51 | hsa00830 Retinol metabolism - Homo sapiens (human) | -1375.23 | 0.068 | 0.241263 | 0.581152 |
| 52 | hsa00790 Folate biosynthesis - Homo sapiens (human) | -338.903 | 0.069 | 0.241263 | 0.994764 |
| 53 | hsa00062 Fatty acid elongation in mitochondria - Homo sapiens (human) | -358.288 | 0.069 | 0.241263 | 0.141361 |
| 54 | hsa00603 Glycosphingolipid biosynthesis - globo series - Homo sapiens (human) | -851.935 | 0.07 | 0.241263 | 0.790576 |
| 55 | hsa04720 Long-term potentiation - Homo sapiens (human) | -1986.91 | 0.071 | 0.241263 | 0.706806 |
| 56 | hsa00600 Sphingolipid metabolism - Homo sapiens (human) | -1783.66 | 0.072 | 0.241263 | 0.837696 |
| 57 | hsa04664 Fc epsilon RI signaling pathway - Homo sapiens (human) | -2853.78 | 0.072 | 0.241263 | 0.989529 |
| 58 | hsa05223 Non-small cell lung cancer - Homo sapiens (human) | -2577.15 | 0.076 | 0.249271 | 0.984293 |
| 59 | hsa00534 Heparan sulfate biosynthesis - Homo sapiens (human) | -1193.13 | 0.077 | 0.249271 | 1 |
| 60 | hsa00150 Androgen and estrogen metabolism - Homo sapiens (human) | -168.342 | 0.084 | 0.2674 | 0.95288 |
| 61 | hsa00982 Drug metabolism - cytochrome P450 - Homo sapiens (human) | -1106.31 | 0.092 | 0.2865 | 0.769634 |
| 62 | hsa00430 Taurine and hypotaurine metabolism - Homo sapiens (human) | -479.972 | 0.093 | 0.2865 | 0.685864 |
| 63 | hsa01032 Glycan structures - degradation - Homo sapiens (human) | -1615.68 | 0.095 | 0.288016 | 1 |
| 64 | hsa05222 Small cell lung cancer - Homo sapiens (human) | -2731.75 | 0.103 | 0.307391 | 0.994764 |
| 65 | hsa05012 Parkinson's disease - Homo sapiens (human) | -2039.19 | 0.11 | 0.323231 | 0.994764 |
| 66 | hsa00770 Pantothenate and CoA biosynthesis - Homo sapiens (human) | -716.573 | 0.122 | 0.350642 | 0.727749 |
| 67 | hsa00410 beta-Alanine metabolism - Homo sapiens (human) | -986.41 | 0.123 | 0.350642 | 0.879581 |
| 68 | hsa04130 SNARE interactions in vesicular transport - Homo sapiens (human) | -1895.57 | 0.127 | 0.35144 | 0.874346 |
| 69 | hsa04060 Cytokine-cytokine receptor interaction - Homo sapiens (human) | -8685.57 | 0.127 | 0.35144 | 1 |
| 70 | hsa04620 Toll-like receptor signaling pathway - Homo sapiens (human) | -4919.23 | 0.132 | 0.35144 | 0.994764 |
| 71 | hsa00640 Propanoate metabolism - Homo sapiens (human) | -1221.44 | 0.135 | 0.35144 | 0.874346 |
| 72 | hsa00130 Ubiquinone and menaquinone biosynthesis - Homo sapiens (human) | -420.004 | 0.135 | 0.35144 | 0.994764 |
| 73 | hsa00230 Purine metabolism - Homo sapiens (human) | -3387.48 | 0.136 | 0.35144 | 0.979058 |
| 74 | hsa05211 Renal cell carcinoma - Homo sapiens (human) | -2951.18 | 0.137 | 0.35144 | 0.994764 |
| 75 | hsa04110 Cell cycle - Homo sapiens (human) | -1565.96 | 0.138 | 0.35144 | 0.900524 |
| 76 | hsa00780 Biotin metabolism - Homo sapiens (human) | -254.659 | 0.14 | 0.351842 | 1 |
| 77 | hsa05215 Prostate cancer - Homo sapiens (human) | -3352.26 | 0.142 | 0.352234 | 1 |
| 78 | hsa03450 Non-homologous end-joining - Homo sapiens (human) | -1106.1 | 0.156 | 0.382 | 1 |
| 79 | hsa00950 Alkaloid biosynthesis I - Homo sapiens (human) | -301.357 | 0.164 | 0.39155 | 0.884817 |
| 80 | hsa00630 Glyoxylate and dicarboxylate metabolism - Homo sapiens (human) | -788.663 | 0.164 | 0.39155 | 0.994764 |
| 81 | hsa04210 Apoptosis - Homo sapiens (human) | -3707.32 | 0.174 | 0.408965 | 0.994764 |
| 82 | hsa04350 TGF-beta signaling pathway - Homo sapiens (human) | -3390.76 | 0.178 | 0.408965 | 1 |
| 83 | hsa04020 Calcium signaling pathway - Homo sapiens (human) | -3301.92 | 0.179 | 0.408965 | 1 |
| 84 | hsa00140 C21-Steroid hormone metabolism - Homo sapiens (human) | -672.273 | 0.181 | 0.408965 | 0.95288 |
| 85 | hsa00440 Aminophosphonate metabolism - Homo sapiens (human) | -341.362 | 0.182 | 0.408965 | 1 |
| 86 | hsa04370 VEGF signaling pathway - Homo sapiens (human) | -2302.57 | 0.191 | 0.424198 | 0.994764 |
| 87 | hsa05216 Thyroid cancer - Homo sapiens (human) | -1147.72 | 0.204 | 0.444236 | 0.994764 |
| 88 | hsa04916 Melanogenesis - Homo sapiens (human) | -2632.12 | 0.205 | 0.444236 | 1 |
| 89 | hsa00010 Glycolysis / Gluconeogenesis - Homo sapiens (human) | -1751.68 | 0.207 | 0.444236 | 0.979058 |
| 90 | hsa00520 Nucleotide sugars metabolism - Homo sapiens (human) | -389.219 | 0.217 | 0.460522 | 0.979058 |
| 91 | hsa00785 Lipoic acid metabolism - Homo sapiens (human) | -89.7018 | 0.221 | 0.462967 | 1 |
| 92 | hsa00240 Pyrimidine metabolism - Homo sapiens (human) | -2429.27 | 0.223 | 0.462967 | 0.979058 |
| 93 | hsa00061 Fatty acid biosynthesis - Homo sapiens (human) | -386.104 | 0.227 | 0.463573 | 0.947644 |
| 94 | hsa02010 ABC transporters - General - Homo sapiens (human) | -3665.56 | 0.231 | 0.463573 | 1 |
| 95 | hsa00550 Peptidoglycan biosynthesis - Homo sapiens (human) | -170.053 | 0.232 | 0.463573 | 1 |
| 96 | hsa04920 Adipocytokine signaling pathway - Homo sapiens (human) | -2932.67 | 0.233 | 0.463573 | 1 |
| 97 | hsa04742 Taste transduction - Homo sapiens (human) | -1440.83 | 0.236 | 0.464701 | 1 |
| 98 | hsa05130 Pathogenic Escherichia coli infection - EHEC - Homo sapiens (human) | -1830.46 | 0.239 | 0.465806 | 1 |
| 99 | hsa00562 Inositol phosphate metabolism - Homo sapiens (human) | -1500.78 | 0.249 | 0.480394 | 0.994764 |
| 100 | hsa04810 Regulation of actin cytoskeleton - Homo sapiens (human) | -3069.18 | 0.258 | 0.49278 | 1 |
| 101 | hsa04012 ErbB signaling pathway - Homo sapiens (human) | -3942.99 | 0.262 | 0.495465 | 1 |
| 102 | hsa00750 Vitamin B6 metabolism - Homo sapiens (human) | -397.87 | 0.273 | 0.511206 | 0.994764 |
| 103 | hsa00040 Pentose and glucuronate interconversions - Homo sapiens (human) | -660.745 | 0.282 | 0.522932 | 1 |
| 104 | hsa00220 Urea cycle and metabolism of amino groups - Homo sapiens (human) | -1535.7 | 0.285 | 0.523413 | 1 |
| 105 | hsa00271 Methionine metabolism - Homo sapiens (human) | -1050.29 | 0.288 | 0.523886 | 0.994764 |
| 106 | hsa01040 Biosynthesis of unsaturated fatty acids - Homo sapiens (human) | -170.489 | 0.297 | 0.53516 | 1 |
| 107 | hsa00910 Nitrogen metabolism - Homo sapiens (human) | -814.946 | 0.307 | 0.548009 | 1 |
| 108 | hsa00604 Glycosphingolipid biosynthesis - ganglio series - Homo sapiens (human) | -1003.32 | 0.344 | 0.60837 | 0.994764 |
| 109 | hsa04660 T cell receptor signaling pathway - Homo sapiens (human) | -4231.7 | 0.354 | 0.620312 | 1 |
| 110 | hsa00350 Tyrosine metabolism - Homo sapiens (human) | -1450.73 | 0.36 | 0.622901 | 1 |
| 111 | hsa00120 Bile acid biosynthesis - Homo sapiens (human) | -341.292 | 0.362 | 0.622901 | 1 |
| 112 | hsa03060 Protein export - Homo sapiens (human) | -597.257 | 0.38 | 0.648036 | 1 |
| 113 | hsa03420 Nucleotide excision repair - Homo sapiens (human) | -2303.95 | 0.385 | 0.650752 | 1 |
| 114 | hsa00030 Pentose phosphate pathway - Homo sapiens (human) | -1229.36 | 0.397 | 0.658621 | 1 |
| 115 | hsa00530 Aminosugars metabolism - Homo sapiens (human) | -768.139 | 0.399 | 0.658621 | 1 |
| 116 | hsa00380 Tryptophan metabolism - Homo sapiens (human) | -341.46 | 0.4 | 0.658621 | 1 |
| 117 | hsa00300 Lysine biosynthesis - Homo sapiens (human) | -335.404 | 0.414 | 0.667697 | 1 |
| 118 | hsa04740 Olfactory transduction - Homo sapiens (human) | -1155.04 | 0.416 | 0.667697 | 1 |
| 119 | hsa05219 Bladder cancer - Homo sapiens (human) | -2171.45 | 0.416 | 0.667697 | 1 |
| 120 | hsa00670 One carbon pool by folate - Homo sapiens (human) | -1167 | 0.425 | 0.670868 | 1 |
| 121 | hsa05110 Vibrio cholerae infection - Homo sapiens (human) | -1513.07 | 0.425 | 0.670868 | 1 |
| 122 | hsa00500 Starch and sucrose metabolism - Homo sapiens (human) | -1546.93 | 0.438 | 0.685721 | 1 |
| 123 | hsa00510 N-Glycan biosynthesis - Homo sapiens (human) | -2464.84 | 0.46 | 0.706397 | 1 |
| 124 | hsa05320 Autoimmune thyroid disease - Homo sapiens (human) | -247.847 | 0.461 | 0.706397 | 1 |
| 125 | hsa05340 Primary immunodeficiency - Homo sapiens (human) | -2902.4 | 0.464 | 0.706397 | 1 |
| 126 | hsa00680 Methane metabolism - Homo sapiens (human) | -428.767 | 0.466 | 0.706397 | 1 |
| 127 | hsa04710 Circadian rhythm - Homo sapiens (human) | -532.216 | 0.476 | 0.715874 | 1 |
| 128 | hsa00564 Glycerophospholipid metabolism - Homo sapiens (human) | -2218.96 | 0.484 | 0.721392 | 1 |
| 129 | hsa03430 Mismatch repair - Homo sapiens (human) | -1024.75 | 0.491 | 0.721392 | 1 |
| 130 | hsa00980 Metabolism of xenobiotics by cytochrome P450 - Homo sapiens (human) | -632.537 | 0.491 | 0.721392 | 1 |
| 131 | hsa04140 Regulation of autophagy - Homo sapiens (human) | -1017.24 | 0.497 | 0.724634 | 1 |
| 132 | hsa05060 Prion disease - Homo sapiens (human) | -853.991 | 0.503 | 0.727826 | 1 |
| 133 | hsa00620 Pyruvate metabolism - Homo sapiens (human) | -1496.72 | 0.512 | 0.735278 | 1 |
| 134 | hsa00052 Galactose metabolism - Homo sapiens (human) | -1105.82 | 0.516 | 0.735493 | 1 |
| 135 | hsa04150 mTOR signaling pathway - Homo sapiens (human) | -2280.65 | 0.524 | 0.741363 | 1 |
| 136 | hsa00720 Reductive carboxylate cycle (CO2 fixation) - Homo sapiens (human) | -445.672 | 0.543 | 0.762596 | 1 |
| 137 | hsa01031 Glycan structures - biosynthesis 2 - Homo sapiens (human) | -2563.01 | 0.557 | 0.776547 | 1 |
| 138 | hsa05220 Chronic myeloid leukemia - Homo sapiens (human) | -3159.14 | 0.565 | 0.781993 | 1 |
| 139 | hsa04115 p53 signaling pathway - Homo sapiens (human) | -4235.06 | 0.577 | 0.792856 | 1 |
| 140 | hsa00511 N-Glycan degradation - Homo sapiens (human) | -683.741 | 0.586 | 0.799471 | 1 |
| 141 | hsa03440 Homologous recombination - Homo sapiens (human) | -1537.75 | 0.602 | 0.815475 | 1 |
| 142 | hsa00310 Lysine degradation - Homo sapiens (human) | -427.723 | 0.612 | 0.823183 | 1 |
| 143 | hsa03010 Ribosome - Homo sapiens (human) | -6068.45 | 0.619 | 0.82634 | 1 |
| 144 | hsa00563 Glycosylphosphatidylinositol(GPI)-anchor biosynthesis - Homo sapiens (human) | -1967.06 | 0.623 | 0.82634 | 1 |
| 145 | hsa05050 Dentatorubropallidoluysian atrophy (DRPLA) - Homo sapiens (human) | -684.173 | 0.637 | 0.82851 | 1 |
| 146 | hsa00860 Porphyrin and chlorophyll metabolism - Homo sapiens (human) | -1370.72 | 0.64 | 0.82851 | 1 |
| 147 | hsa04612 Antigen processing and presentation - Homo sapiens (human) | -2027.46 | 0.643 | 0.82851 | 1 |
| 148 | hsa00641 3-Chloroacrylic acid degradation - Homo sapiens (human) | -90.3753 | 0.647 | 0.82851 | 1 |
| 149 | hsa03410 Base excision repair - Homo sapiens (human) | -2050.9 | 0.648 | 0.82851 | 1 |
| 150 | hsa00592 alpha-Linolenic acid metabolism - Homo sapiens (human) | -235.208 | 0.652 | 0.82851 | 1 |
| 151 | hsa00190 Oxidative phosphorylation - Homo sapiens (human) | -7264.47 | 0.655 | 0.82851 | 1 |
| 152 | hsa00920 Sulfur metabolism - Homo sapiens (human) | -582.414 | 0.662 | 0.831855 | 1 |
| 153 | hsa04950 Maturity onset diabetes of the young - Homo sapiens (human) | -1860.64 | 0.682 | 0.851386 | 1 |
| 154 | hsa00533 Keratan sulfate biosynthesis - Homo sapiens (human) | -855.258 | 0.696 | 0.855242 | 1 |
| 155 | hsa05217 Basal cell carcinoma - Homo sapiens (human) | -1426.27 | 0.697 | 0.855242 | 1 |
| 156 | hsa04514 Cell adhesion molecules (CAMs) - Homo sapiens (human) | -6470.9 | 0.701 | 0.855242 | 1 |
| 157 | hsa05213 Endometrial cancer - Homo sapiens (human) | -2470.36 | 0.703 | 0.855242 | 1 |
| 158 | hsa00330 Arginine and proline metabolism - Homo sapiens (human) | -1750.27 | 0.713 | 0.861918 | 1 |
| 159 | hsa00983 Drug metabolism - other enzymes - Homo sapiens (human) | -1658.48 | 0.721 | 0.866107 | 1 |
| 160 | hsa00532 Chondroitin sulfate biosynthesis - Homo sapiens (human) | -1017.55 | 0.732 | 0.873825 | 1 |
| 161 | hsa04730 Long-term depression - Homo sapiens (human) | -2111.89 | 0.748 | 0.885865 | 1 |
| 162 | hsa00450 Selenoamino acid metabolism - Homo sapiens (human) | -513.568 | 0.752 | 0.885865 | 1 |
| 163 | hsa04614 Renin-angiotensin system - Homo sapiens (human) | -1368.31 | 0.756 | 0.885865 | 1 |
| 164 | hsa00900 Terpenoid biosynthesis - Homo sapiens (human) | -342.42 | 0.765 | 0.890945 | 1 |
| 165 | hsa00360 Phenylalanine metabolism - Homo sapiens (human) | -171.557 | 0.773 | 0.894806 | 1 |
| 166 | hsa00281 Geraniol degradation - Homo sapiens (human) | -171.557 | 0.786 | 0.904373 | 1 |
| 167 | hsa00930 Caprolactam degradation - Homo sapiens (human) | -252.903 | 0.792 | 0.90582 | 1 |
| 168 | hsa00232 Caffeine metabolism - Homo sapiens (human) | -211.356 | 0.803 | 0.908935 | 1 |
| 169 | hsa00363 Bisphenol A degradation - Homo sapiens (human) | -171.557 | 0.808 | 0.908935 | 1 |
| 170 | hsa03050 Proteasome - Homo sapiens (human) | -3592.79 | 0.809 | 0.908935 | 1 |
| 171 | hsa05214 Glioma - Homo sapiens (human) | -2639.51 | 0.818 | 0.913673 | 1 |
| 172 | hsa04120 Ubiquitin mediated proteolysis - Homo sapiens (human) | -8037.9 | 0.841 | 0.933901 | 1 |
| 173 | hsa05218 Melanoma - Homo sapiens (human) | -1875.42 | 0.858 | 0.943062 | 1 |
| 174 | hsa03030 DNA replication - Homo sapiens (human) | -2736.59 | 0.867 | 0.943062 | 1 |
| 175 | hsa05332 Graft-versus-host disease - Homo sapiens (human) | -1194.57 | 0.869 | 0.943062 | 1 |
| 176 | hsa05310 Asthma - Homo sapiens (human) | -250.78 | 0.869 | 0.943062 | 1 |
| 177 | hsa00650 Butanoate metabolism - Homo sapiens (human) | -1112.9 | 0.88 | 0.949605 | 1 |
| 178 | hsa00361 gamma-Hexachlorocyclohexane degradation - Homo sapiens (human) | -420.477 | 0.886 | 0.950708 | 1 |
| 179 | hsa01030 Glycan structures - biosynthesis 1 - Homo sapiens (human) | -4789.84 | 0.892 | 0.951799 | 1 |
| 180 | hsa00460 Cyanoamino acid metabolism - Homo sapiens (human) | -342.881 | 0.9 | 0.951852 | 1 |
| 181 | hsa04080 Neuroactive ligand-receptor interaction - Homo sapiens (human) | -7431.95 | 0.904 | 0.951852 | 1 |
| 182 | hsa00053 Ascorbate and aldarate metabolism - Homo sapiens (human) | -257.302 | 0.907 | 0.951852 | 1 |
| 183 | hsa00471 D-Glutamine and D-glutamate metabolism - Homo sapiens (human) | -90.5097 | 0.922 | 0.961228 | 1 |
| 184 | hsa04640 Hematopoietic cell lineage - Homo sapiens (human) | -5474.49 | 0.926 | 0.961228 | 1 |
| 185 | hsa00730 Thiamine metabolism - Homo sapiens (human) | -257.369 | 0.938 | 0.968422 | 1 |
| 186 | hsa00565 Ether lipid metabolism - Homo sapiens (human) | -751.85 | 0.947 | 0.972457 | 1 |
| 187 | hsa00400 Phenylalanine, tyrosine and tryptophan biosynthesis - Homo sapiens (human) | -396.526 | 0.963 | 0.983447 | 1 |
| 188 | hsa03020 RNA polymerase - Homo sapiens (human) | -2055.81 | 0.968 | 0.983447 | 1 |
| 189 | hsa00960 Alkaloid biosynthesis II - Homo sapiens (human) | -258.973 | 0.982 | 0.992392 | 1 |
| 190 | hsa00031 Inositol metabolism - Homo sapiens (human) | -171.691 | 1 | 1 | 1 |
| 191 | hsa00591 Linoleic acid metabolism - Homo sapiens (human) | -171.691 | 1 | 1 | 1 |
| 192 | hsa00364 Fluorobenzoate degradation - Homo sapiens (human) | -1 | -1 | -1 | -1 |
| 193 | hsa00472 D-Arginine and D-ornithine metabolism - Homo sapiens (human) | -1 | -1 | -1 | -1 |
| 194 | hsa00624 1- and 2-Methylnaphthalene degradation - Homo sapiens (human) | -1 | -1 | -1 | -1 |
| 195 | hsa00625 Tetrachloroethene degradation - Homo sapiens (human) | -1 | -1 | -1 | -1 |
| 196 | hsa00627 1,4-Dichlorobenzene degradation - Homo sapiens (human) | -1 | -1 | -1 | -1 |
| 197 | hsa00632 Benzoate degradation via CoA ligation - Homo sapiens (human) | -1 | -1 | -1 | -1 |
| 198 | hsa00740 Riboflavin metabolism - Homo sapiens (human) | -1 | -1 | -1 | -1 |
| 199 | hsa00791 Atrazine degradation - Homo sapiens (human) | -1 | -1 | -1 | -1 |
| 200 | hsa00902 Monoterpenoid biosynthesis - Homo sapiens (human) | -1 | -1 | -1 | -1 |
| 201 | hsa00903 Limonene and pinene degradation - Homo sapiens (human) | -1 | -1 | -1 | -1 |
| 202 | hsa01430 Cell Communication - Homo sapiens (human) | -1 | -1 | -1 | -1 |
| 203 | hsa04340 Hedgehog signaling pathway - Homo sapiens (human) | -1 | -1 | -1 | -1 |
| 204 | hsa04940 Type I diabetes mellitus - Homo sapiens (human) | -1 | -1 | -1 | -1 |
| 205 | hsa05016 - Homo sapiens (human) | -1 | -1 | -1 | -1 |

Result obtained by the GSEA methods in the colon cancer dataset

| No | geneset | size | ES | NES | NOM p-value | FDR q-value | FWER p-value | Rank at max |
| --- | --- | --- | --- | --- | --- | --- | --- | --- |
| 1 | KEGG_PEROXISOME | 72 | -0.647 | -1.978 | 0 | 0.039 | 0.026 | 2,905 |
| 2 | KEGG_PYRUVATE_METABOLISM | 40 | -0.671 | -1.972 | 0 | 0.023 | 0.032 | 1,809 |
| 3 | KEGG_VALINE_LEUCINE_AND_ISOLEUCINE_DEGRADATION | 43 | -0.801 | -1.941 | 0 | 0.023 | 0.047 | 677 |
| 4 | KEGG_HUNTINGTONS_DISEASE | 164 | -0.599 | -1.938 | 0.004 | 0.014 | 0.049 | 1,981 |
| 5 | KEGG_PROPANOATE_METABOLISM | 31 | -0.799 | -1.939 | 0 | 0.018 | 0.049 | 1,724 |
| 6 | KEGG_BUTANOATE_METABOLISM | 31 | -0.809 | -1.921 | 0 | 0.016 | 0.06 | 1,152 |
| 7 | KEGG_TRYPTOPHAN_METABOLISM | 38 | -0.53 | -1.89 | 0.004 | 0.018 | 0.076 | 1,138 |
| 8 | KEGG_LYSINE_DEGRADATION | 43 | -0.516 | -1.89 | 0 | 0.02 | 0.076 | 1,138 |
| 9 | KEGG_FATTY_ACID_METABOLISM | 39 | -0.671 | -1.873 | 0 | 0.019 | 0.091 | 2,524 |
| 10 | KEGG_HISTIDINE_METABOLISM | 27 | -0.589 | -1.87 | 0.002 | 0.018 | 0.097 | 1,138 |
| 11 | KEGG_ALZHEIMERS_DISEASE | 149 | -0.588 | -1.868 | 0.016 | 0.017 | 0.098 | 1,981 |
| 12 | KEGG_OXIDATIVE_PHOSPHORYLATION | 110 | -0.711 | -1.851 | 0.01 | 0.02 | 0.127 | 3,341 |
| 13 | KEGG_CITRATE_CYCLE_TCA_CYCLE | 29 | -0.855 | -1.835 | 0 | 0.022 | 0.142 | 1,785 |
| 14 | KEGG_PARKINSONS_DISEASE | 106 | -0.676 | -1.805 | 0.018 | 0.028 | 0.182 | 1,981 |
| 15 | KEGG_STARCH_AND_SUCROSE_METABOLISM | 39 | -0.594 | -1.792 | 0 | 0.031 | 0.2 | 2,971 |
| 16 | KEGG_GLYCOSYLPHOSPHATIDYLINOSITOL_GPI_ANCHOR_BIOSYNTHESIS | 24 | -0.613 | -1.769 | 0.002 | 0.037 | 0.228 | 5,085 |
| 17 | KEGG_ASCORBATE_AND_ALDARATE_METABOLISM | 16 | -0.746 | -1.768 | 0.01 | 0.035 | 0.229 | 2,971 |
| 18 | KEGG_BETA_ALANINE_METABOLISM | 22 | -0.621 | -1.766 | 0.009 | 0.033 | 0.23 | 1,138 |
| 19 | KEGG_PENTOSE_AND_GLUCURONATE_INTERCONVERSIONS | 18 | -0.666 | -1.758 | 0.004 | 0.034 | 0.239 | 2,971 |
| 20 | KEGG_GLYOXYLATE_AND_DICARBOXYLATE_METABOLISM | 15 | -0.615 | -1.73 | 0.013 | 0.041 | 0.291 | 2,173 |
| 21 | KEGG_ECM_RECEPTOR_INTERACTION | 81 | 0.515 | 1.746 | 0.024 | 0.547 | 0.298 | 4,063 |
| 22 | KEGG_GLYCOLYSIS_GLUCONEOGENESIS | 60 | -0.512 | -1.71 | 0.012 | 0.048 | 0.328 | 2,886 |
| 23 | KEGG_TERPENOID_BACKBONE_BIOSYNTHESIS | 15 | -0.745 | -1.689 | 0.004 | 0.056 | 0.382 | 2,744 |
| 24 | KEGG_PORPHYRIN_AND_CHLOROPHYLL_METABOLISM | 31 | -0.53 | -1.662 | 0.013 | 0.067 | 0.443 | 2,971 |
| 25 | KEGG_RETINOL_METABOLISM | 49 | -0.528 | -1.661 | 0.013 | 0.066 | 0.449 | 3,084 |
| 26 | KEGG_RIBOFLAVIN_METABOLISM | 16 | -0.629 | -1.627 | 0.058 | 0.081 | 0.53 | 1,275 |
| 27 | KEGG_STEROID_BIOSYNTHESIS | 16 | -0.692 | -1.626 | 0.029 | 0.079 | 0.531 | 3,076 |
| 28 | KEGG_METABOLISM_OF_XENOBIOTICS_BY_CYTOCHROME_P450 | 60 | -0.496 | -1.618 | 0.035 | 0.081 | 0.553 | 3,084 |
| 29 | KEGG_PENTOSE_PHOSPHATE_PATHWAY | 26 | -0.546 | -1.607 | 0.036 | 0.084 | 0.573 | 2,825 |
| 30 | KEGG_FOCAL_ADHESION | 188 | 0.431 | 1.621 | 0.026 | 0.871 | 0.575 | 4,396 |
| 31 | KEGG_CELL_ADHESION_MOLECULES_CAMS | 125 | 0.473 | 1.615 | 0.043 | 0.609 | 0.59 | 5,995 |
| 32 | KEGG_BASAL_CELL_CARCINOMA | 53 | 0.465 | 1.608 | 0.018 | 0.486 | 0.603 | 7,312 |
| 33 | KEGG_PROXIMAL_TUBULE_BICARBONATE_RECLAMATION | 23 | -0.574 | -1.598 | 0.027 | 0.087 | 0.608 | 3,337 |
| 34 | KEGG_SELENOAMINO_ACID_METABOLISM | 21 | -0.533 | -1.594 | 0.009 | 0.087 | 0.613 | 3,016 |
| 35 | KEGG_DRUG_METABOLISM_OTHER_ENZYMES | 39 | -0.512 | -1.587 | 0.028 | 0.088 | 0.634 | 3,602 |
| 36 | KEGG_ARGININE_AND_PROLINE_METABOLISM | 52 | -0.458 | -1.579 | 0.031 | 0.09 | 0.654 | 2,730 |
| 37 | KEGG_PPAR_SIGNALING_PATHWAY | 66 | -0.421 | -1.572 | 0.038 | 0.089 | 0.668 | 1,972 |
| 38 | KEGG_DRUG_METABOLISM_CYTOCHROME_P450 | 61 | -0.453 | -1.572 | 0.024 | 0.092 | 0.668 | 3,084 |
| 39 | KEGG_PRION_DISEASES | 35 | 0.451 | 1.577 | 0.01 | 0.495 | 0.678 | 2,482 |
| 40 | KEGG_LEUKOCYTE_TRANSENDOTHELIAL_MIGRATION | 107 | 0.393 | 1.554 | 0.032 | 0.483 | 0.726 | 4,138 |
| 41 | KEGG_THYROID_CANCER | 29 | -0.448 | -1.539 | 0.024 | 0.111 | 0.755 | 2,909 |
| 42 | KEGG_VIBRIO_CHOLERAE_INFECTION | 50 | -0.451 | -1.528 | 0.069 | 0.115 | 0.772 | 3,976 |
| 43 | KEGG_STEROID_HORMONE_BIOSYNTHESIS | 45 | -0.46 | -1.525 | 0.043 | 0.114 | 0.773 | 3,181 |
| 44 | KEGG_NICOTINATE_AND_NICOTINAMIDE_METABOLISM | 21 | -0.504 | -1.522 | 0.025 | 0.113 | 0.775 | 2,242 |
| 45 | KEGG_MTOR_SIGNALING_PATHWAY | 46 | 0.389 | 1.531 | 0.02 | 0.488 | 0.785 | 3,751 |
| 46 | KEGG_COMPLEMENT_AND_COAGULATION_CASCADES | 66 | 0.445 | 1.523 | 0.044 | 0.45 | 0.801 | 6,686 |
| 47 | KEGG_NITROGEN_METABOLISM | 23 | -0.499 | -1.494 | 0.061 | 0.131 | 0.831 | 3,318 |
| 48 | KEGG_CARDIAC_MUSCLE_CONTRACTION | 70 | -0.396 | -1.485 | 0.034 | 0.132 | 0.845 | 2,216 |
| 49 | KEGG_BIOSYNTHESIS_OF_UNSATURATED_FATTY_ACIDS | 19 | -0.513 | -1.486 | 0.074 | 0.135 | 0.845 | 1,900 |
| 50 | KEGG_GLYCEROPHOSPHOLIPID_METABOLISM | 64 | -0.382 | -1.463 | 0.056 | 0.146 | 0.875 | 2,396 |
| 51 | KEGG_INTESTINAL_IMMUNE_NETWORK_FOR_IGA_PRODUCTION | 45 | 0.578 | 1.468 | 0.11 | 0.563 | 0.888 | 5,665 |
| 52 | KEGG_GLYCEROLIPID_METABOLISM | 43 | -0.425 | -1.45 | 0.055 | 0.155 | 0.892 | 3,233 |
| 53 | KEGG_PRIMARY_IMMUNODEFICIENCY | 35 | 0.598 | 1.441 | 0.148 | 0.596 | 0.914 | 5,832 |
| 54 | KEGG_PATHWAYS_IN_CANCER | 315 | 0.292 | 1.421 | 0.014 | 0.602 | 0.926 | 3,867 |
| 55 | KEGG_CYSTEINE_AND_METHIONINE_METABOLISM | 31 | -0.426 | -1.408 | 0.077 | 0.19 | 0.928 | 3,662 |
| 56 | KEGG_PROTEASOME | 42 | -0.553 | -1.401 | 0.179 | 0.193 | 0.936 | 4,651 |
| 57 | KEGG_MAPK_SIGNALING_PATHWAY | 256 | 0.314 | 1.39 | 0.029 | 0.655 | 0.957 | 2,666 |
| 58 | KEGG_SPHINGOLIPID_METABOLISM | 30 | -0.507 | -1.383 | 0.142 | 0.207 | 0.96 | 4,199 |
| 59 | KEGG_AMINO_SUGAR_AND_NUCLEOTIDE_SUGAR_METABOLISM | 44 | -0.435 | -1.375 | 0.15 | 0.212 | 0.963 | 3,247 |
| 60 | KEGG_ASTHMA | 27 | 0.548 | 1.375 | 0.172 | 0.652 | 0.965 | 5,297 |
| 61 | KEGG_FRUCTOSE_AND_MANNOSE_METABOLISM | 34 | -0.46 | -1.365 | 0.153 | 0.218 | 0.973 | 3,747 |
| 62 | KEGG_ALANINE_ASPARTATE_AND_GLUTAMATE_METABOLISM | 32 | -0.387 | -1.339 | 0.093 | 0.238 | 0.978 | 3,296 |
| 63 | KEGG_ENDOCYTOSIS | 157 | -0.316 | -1.34 | 0.104 | 0.241 | 0.978 | 3,372 |
| 64 | KEGG_LEISHMANIA_INFECTION | 62 | 0.45 | 1.346 | 0.191 | 0.699 | 0.979 | 6,138 |
| 65 | KEGG_NOTCH_SIGNALING_PATHWAY | 41 | 0.372 | 1.345 | 0.125 | 0.655 | 0.979 | 4,412 |
| 66 | KEGG_AXON_GUIDANCE | 127 | 0.291 | 1.335 | 0.048 | 0.647 | 0.982 | 3,463 |
| 67 | KEGG_COLORECTAL_CANCER | 61 | 0.34 | 1.331 | 0.094 | 0.619 | 0.984 | 3,751 |
| 68 | KEGG_PANTOTHENATE_AND_COA_BIOSYNTHESIS | 16 | -0.461 | -1.301 | 0.145 | 0.277 | 0.985 | 736 |
| 69 | KEGG_AMYOTROPHIC_LATERAL_SCLEROSIS_ALS | 49 | -0.33 | -1.291 | 0.105 | 0.286 | 0.986 | 762 |
| 70 | KEGG_B_CELL_RECEPTOR_SIGNALING_PATHWAY | 71 | 0.393 | 1.316 | 0.193 | 0.627 | 0.987 | 6,740 |
| 71 | KEGG_INOSITOL_PHOSPHATE_METABOLISM | 46 | -0.334 | -1.278 | 0.115 | 0.286 | 0.988 | 2,560 |
| 72 | KEGG_N_GLYCAN_BIOSYNTHESIS | 42 | -0.419 | -1.279 | 0.196 | 0.29 | 0.988 | 4,364 |
| 73 | KEGG_GLUTATHIONE_METABOLISM | 45 | -0.424 | -1.287 | 0.198 | 0.285 | 0.988 | 2,920 |
| 74 | KEGG_SMALL_CELL_LUNG_CANCER | 82 | 0.305 | 1.31 | 0.088 | 0.613 | 0.99 | 3,974 |
| 75 | KEGG_DILATED_CARDIOMYOPATHY | 89 | 0.367 | 1.282 | 0.141 | 0.663 | 0.994 | 5,563 |
| 76 | KEGG_TYPE_II_DIABETES_MELLITUS | 44 | 0.346 | 1.271 | 0.136 | 0.662 | 0.994 | 6,771 |
| 77 | KEGG_CHEMOKINE_SIGNALING_PATHWAY | 173 | 0.324 | 1.269 | 0.191 | 0.636 | 0.994 | 4,261 |
| 78 | KEGG_WNT_SIGNALING_PATHWAY | 144 | 0.3 | 1.268 | 0.096 | 0.611 | 0.994 | 4,831 |
| 79 | KEGG_HYPERTROPHIC_CARDIOMYOPATHY_HCM | 82 | 0.367 | 1.264 | 0.154 | 0.595 | 0.994 | 5,563 |
| 80 | KEGG_ADHERENS_JUNCTION | 73 | -0.298 | -1.251 | 0.154 | 0.309 | 0.995 | 1,964 |
| 81 | KEGG_LINOLEIC_ACID_METABOLISM | 27 | -0.446 | -1.252 | 0.212 | 0.313 | 0.995 | 4,764 |
| 82 | KEGG_MELANOMA | 71 | 0.316 | 1.253 | 0.147 | 0.598 | 0.996 | 4,678 |
| 83 | KEGG_O_GLYCAN_BIOSYNTHESIS | 29 | -0.423 | -1.245 | 0.226 | 0.311 | 0.996 | 1,764 |
| 84 | KEGG_HEMATOPOIETIC_CELL_LINEAGE | 83 | 0.399 | 1.235 | 0.28 | 0.618 | 0.998 | 5,524 |
| 85 | KEGG_T_CELL_RECEPTOR_SIGNALING_PATHWAY | 107 | 0.323 | 1.23 | 0.25 | 0.605 | 0.998 | 6,138 |
| 86 | KEGG_JAK_STAT_SIGNALING_PATHWAY | 151 | 0.316 | 1.227 | 0.221 | 0.593 | 0.998 | 8,376 |
| 87 | KEGG_CHRONIC_MYELOID_LEUKEMIA | 72 | 0.299 | 1.225 | 0.187 | 0.575 | 0.998 | 3,751 |
| 88 | KEGG_CYTOKINE_CYTOKINE_RECEPTOR_INTERACTION | 245 | 0.334 | 1.225 | 0.231 | 0.557 | 0.998 | 5,342 |
| 89 | KEGG_VIRAL_MYOCARDITIS | 67 | 0.363 | 1.215 | 0.271 | 0.561 | 0.998 | 5,297 |
| 90 | KEGG_AMINOACYL_TRNA_BIOSYNTHESIS | 32 | -0.386 | -1.197 | 0.268 | 0.351 | 0.998 | 2,681 |
| 91 | KEGG_FC_GAMMA_R_MEDIATED_PHAGOCYTOSIS | 87 | -0.288 | -1.208 | 0.192 | 0.342 | 0.998 | 2,897 |
| 92 | KEGG_PRIMARY_BILE_ACID_BIOSYNTHESIS | 16 | -0.394 | -1.211 | 0.199 | 0.344 | 0.998 | 2,609 |
| 93 | KEGG_GALACTOSE_METABOLISM | 25 | -0.38 | -1.22 | 0.191 | 0.337 | 0.998 | 2,602 |
| 94 | KEGG_ALPHA_LINOLENIC_ACID_METABOLISM | 17 | -0.434 | -1.188 | 0.262 | 0.352 | 0.999 | 2,396 |
| 95 | KEGG_RENIN_ANGIOTENSIN_SYSTEM | 16 | -0.42 | -1.19 | 0.266 | 0.354 | 0.999 | 2,455 |
| 96 | KEGG_VASCULAR_SMOOTH_MUSCLE_CONTRACTION | 110 | 0.316 | 1.19 | 0.293 | 0.599 | 1 | 5,107 |
| 97 | KEGG_GLYCOSPHINGOLIPID_BIOSYNTHESIS_GANGLIO_SERIES | 15 | 0.407 | 1.184 | 0.239 | 0.592 | 1 | 5,244 |
| 98 | KEGG_GAP_JUNCTION | 75 | 0.294 | 1.157 | 0.226 | 0.635 | 1 | 3,836 |
| 99 | KEGG_REGULATION_OF_ACTIN_CYTOSKELETON | 196 | 0.259 | 1.155 | 0.235 | 0.622 | 1 | 4,686 |
| 100 | KEGG_MELANOGENESIS | 98 | 0.29 | 1.154 | 0.228 | 0.607 | 1 | 3,081 |
| 101 | KEGG_TGF_BETA_SIGNALING_PATHWAY | 82 | 0.294 | 1.141 | 0.259 | 0.617 | 1 | 7,570 |
| 102 | KEGG_HEDGEHOG_SIGNALING_PATHWAY | 53 | 0.299 | 1.134 | 0.266 | 0.617 | 1 | 5,498 |
| 103 | KEGG_TOLL_LIKE_RECEPTOR_SIGNALING_PATHWAY | 98 | 0.32 | 1.13 | 0.33 | 0.61 | 1 | 6,661 |
| 104 | KEGG_GLIOMA | 63 | 0.268 | 1.113 | 0.267 | 0.628 | 1 | 3,751 |
| 105 | KEGG_AUTOIMMUNE_THYROID_DISEASE | 49 | 0.418 | 1.101 | 0.389 | 0.638 | 1 | 5,297 |
| 106 | KEGG_ERBB_SIGNALING_PATHWAY | 85 | 0.251 | 1.09 | 0.297 | 0.646 | 1 | 3,751 |
| 107 | KEGG_GLYCOSAMINOGLYCAN_BIOSYNTHESIS_HEPARAN_SULFATE | 25 | 0.341 | 1.084 | 0.333 | 0.643 | 1 | 8,564 |
| 108 | KEGG_PANCREATIC_CANCER | 69 | 0.249 | 1.082 | 0.31 | 0.632 | 1 | 3,751 |
| 109 | KEGG_PROGESTERONE_MEDIATED_OOCYTE_MATURATION | 82 | 0.249 | 1.082 | 0.334 | 0.619 | 1 | 3,825 |
| 110 | KEGG_CYTOSOLIC_DNA_SENSING_PATHWAY | 51 | 0.329 | 1.073 | 0.378 | 0.623 | 1 | 6,639 |
| 111 | KEGG_SYSTEMIC_LUPUS_ERYTHEMATOSUS | 107 | 0.296 | 1.071 | 0.362 | 0.614 | 1 | 3,982 |
| 112 | KEGG_ACUTE_MYELOID_LEUKEMIA | 56 | 0.275 | 1.056 | 0.374 | 0.63 | 1 | 6,661 |
| 113 | KEGG_SNARE_INTERACTIONS_IN_VESICULAR_TRANSPORT | 34 | 0.279 | 1.056 | 0.366 | 0.617 | 1 | 2,771 |
| 114 | KEGG_PURINE_METABOLISM | 149 | 0.228 | 1.05 | 0.344 | 0.617 | 1 | 4,309 |
| 115 | KEGG_GLYCINE_SERINE_AND_THREONINE_METABOLISM | 31 | 0.297 | 1.049 | 0.387 | 0.606 | 1 | 2,083 |
| 116 | KEGG_DORSO_VENTRAL_AXIS_FORMATION | 23 | 0.31 | 1.047 | 0.388 | 0.598 | 1 | 2,803 |
| 117 | KEGG_GRAFT_VERSUS_HOST_DISEASE | 37 | 0.423 | 1.04 | 0.459 | 0.599 | 1 | 5,297 |
| 118 | KEGG_PROSTATE_CANCER | 87 | 0.237 | 1.017 | 0.393 | 0.629 | 1 | 3,825 |
| 119 | KEGG_NEUROACTIVE_LIGAND_RECEPTOR_INTERACTION | 252 | 0.262 | 1.015 | 0.418 | 0.622 | 1 | 8,368 |
| 120 | KEGG_RIBOSOME | 79 | 0.511 | 0.994 | 0.571 | 0.649 | 1 | 7,902 |
| 121 | KEGG_RENAL_CELL_CARCINOMA | 69 | 0.243 | 0.994 | 0.438 | 0.638 | 1 | 3,751 |
| 122 | KEGG_ANTIGEN_PROCESSING_AND_PRESENTATION | 81 | 0.303 | 0.992 | 0.478 | 0.631 | 1 | 6,066 |
| 123 | KEGG_NEUROTROPHIN_SIGNALING_PATHWAY | 122 | 0.233 | 0.99 | 0.44 | 0.623 | 1 | 3,751 |
| 124 | KEGG_EPITHELIAL_CELL_SIGNALING_IN_HELICOBACTER_PYLORI_INFECTION | 62 | 0.241 | 0.965 | 0.509 | 0.657 | 1 | 3,697 |
| 125 | KEGG_TYPE_I_DIABETES_MELLITUS | 40 | 0.373 | 0.959 | 0.533 | 0.658 | 1 | 5,700 |
| 126 | KEGG_PHOSPHATIDYLINOSITOL_SIGNALING_SYSTEM | 67 | 0.226 | 0.955 | 0.56 | 0.652 | 1 | 4,811 |
| 127 | KEGG_ARRHYTHMOGENIC_RIGHT_VENTRICULAR_CARDIOMYOPATHY_ARVC | 73 | 0.252 | 0.952 | 0.557 | 0.647 | 1 | 5,995 |
| 128 | KEGG_ALLOGRAFT_REJECTION | 34 | 0.405 | 0.949 | 0.533 | 0.642 | 1 | 5,700 |
| 129 | KEGG_CALCIUM_SIGNALING_PATHWAY | 171 | 0.231 | 0.944 | 0.557 | 0.641 | 1 | 4,439 |
| 130 | KEGG_GLYCOSAMINOGLYCAN_BIOSYNTHESIS_KERATAN_SULFATE | 15 | 0.335 | 0.9 | 0.573 | 0.706 | 1 | 8,806 |
| 131 | KEGG_ADIPOCYTOKINE_SIGNALING_PATHWAY | 66 | 0.206 | 0.881 | 0.725 | 0.728 | 1 | 6,319 |
| 132 | KEGG_NATURAL_KILLER_CELL_MEDIATED_CYTOTOXICITY | 129 | 0.247 | 0.871 | 0.593 | 0.734 | 1 | 5,235 |
| 133 | KEGG_VEGF_SIGNALING_PATHWAY | 71 | 0.213 | 0.862 | 0.705 | 0.738 | 1 | 6,740 |
| 134 | KEGG_GLYCOSAMINOGLYCAN_DEGRADATION | 21 | 0.291 | 0.828 | 0.661 | 0.782 | 1 | 6,478 |
| 135 | KEGG_OLFACTORY_TRANSDUCTION | 114 | 0.209 | 0.808 | 0.766 | 0.803 | 1 | 9,392 |
| 136 | KEGG_NOD_LIKE_RECEPTOR_SIGNALING_PATHWAY | 51 | 0.203 | 0.767 | 0.813 | 0.854 | 1 | 6,138 |
| 137 | KEGG_RNA_POLYMERASE | 28 | 0.255 | 0.763 | 0.719 | 0.848 | 1 | 9,776 |
| 138 | KEGG_ONE_CARBON_POOL_BY_FOLATE | 16 | 0.264 | 0.729 | 0.748 | 0.882 | 1 | 5,245 |
| 139 | KEGG_CELL_CYCLE | 113 | 0.187 | 0.697 | 0.801 | 0.908 | 1 | 5,455 |
| 140 | KEGG_TASTE_TRANSDUCTION | 44 | 0.189 | 0.66 | 0.945 | 0.933 | 1 | 6,104 |
| 141 | KEGG_DNA_REPLICATION | 34 | 0.227 | 0.59 | 0.843 | 0.974 | 1 | 5,626 |
| 142 | KEGG_RNA_DEGRADATION | 51 | 0.17 | 0.585 | 0.937 | 0.965 | 1 | 6,081 |
| 143 | KEGG_HOMOLOGOUS_RECOMBINATION | 26 | 0.146 | 0.468 | 0.998 | 0.995 | 1 | 3,971 |
| 144 | KEGG_MISMATCH_REPAIR | 22 | 0.155 | 0.385 | 0.995 | 0.995 | 1 | 8,925 |
| 145 | KEGG_NUCLEOTIDE_EXCISION_REPAIR | 44 | -0.151 | -0.492 | 0.976 | 0.98 | 1 | 4,520 |
| 146 | KEGG_RIG_I_LIKE_RECEPTOR_SIGNALING_PATHWAY | 65 | -0.162 | -0.587 | 0.959 | 0.951 | 1 | 1,177 |
| 147 | KEGG_BASE_EXCISION_REPAIR | 33 | -0.168 | -0.591 | 0.962 | 0.959 | 1 | 2,998 |
| 148 | KEGG_BASAL_TRANSCRIPTION_FACTORS | 33 | -0.165 | -0.614 | 0.964 | 0.955 | 1 | 4,853 |
| 149 | KEGG_SPLICEOSOME | 96 | -0.172 | -0.625 | 0.861 | 0.957 | 1 | 4,829 |
| 150 | KEGG_UBIQUITIN_MEDIATED_PROTEOLYSIS | 122 | -0.175 | -0.763 | 0.846 | 0.818 | 1 | 3,495 |
| 151 | KEGG_BLADDER_CANCER | 40 | -0.209 | -0.765 | 0.822 | 0.825 | 1 | 3,858 |
| 152 | KEGG_NON_SMALL_CELL_LUNG_CANCER | 52 | -0.201 | -0.769 | 0.807 | 0.829 | 1 | 2,912 |
| 153 | KEGG_REGULATION_OF_AUTOPHAGY | 33 | -0.235 | -0.8 | 0.755 | 0.792 | 1 | 2,995 |
| 154 | KEGG_P53_SIGNALING_PATHWAY | 64 | -0.226 | -0.821 | 0.698 | 0.768 | 1 | 3,488 |
| 155 | KEGG_VASOPRESSIN_REGULATED_WATER_REABSORPTION | 44 | -0.223 | -0.844 | 0.744 | 0.741 | 1 | 3,726 |
| 156 | KEGG_PYRIMIDINE_METABOLISM | 88 | -0.203 | -0.856 | 0.667 | 0.73 | 1 | 2,998 |
| 157 | KEGG_LONG_TERM_DEPRESSION | 66 | -0.227 | -0.888 | 0.635 | 0.687 | 1 | 2,552 |
| 158 | KEGG_ALDOSTERONE_REGULATED_SODIUM_REABSORPTION | 40 | -0.254 | -0.89 | 0.596 | 0.691 | 1 | 2,613 |
| 159 | KEGG_ENDOMETRIAL_CANCER | 51 | -0.224 | -0.892 | 0.637 | 0.696 | 1 | 3,923 |
| 160 | KEGG_GLYCOSPHINGOLIPID_BIOSYNTHESIS_LACTO_AND_NEOLACTO_SERIES | 26 | -0.286 | -0.894 | 0.618 | 0.702 | 1 | 2,363 |
| 161 | KEGG_LYSOSOME | 115 | -0.269 | -0.911 | 0.557 | 0.681 | 1 | 3,841 |
| 162 | KEGG_APOPTOSIS | 82 | -0.24 | -0.945 | 0.563 | 0.631 | 1 | 3,180 |
| 163 | KEGG_MATURITY_ONSET_DIABETES_OF_THE_YOUNG | 19 | -0.379 | -0.971 | 0.496 | 0.595 | 1 | 4,123 |
| 164 | KEGG_INSULIN_SIGNALING_PATHWAY | 131 | -0.224 | -0.972 | 0.513 | 0.6 | 1 | 2,932 |
| 165 | KEGG_LONG_TERM_POTENTIATION | 68 | -0.241 | -0.978 | 0.504 | 0.599 | 1 | 4,360 |
| 166 | KEGG_FC_EPSILON_RI_SIGNALING_PATHWAY | 75 | -0.241 | -0.986 | 0.467 | 0.592 | 1 | 2,552 |
| 167 | KEGG_TYROSINE_METABOLISM | 39 | -0.262 | -1.012 | 0.43 | 0.555 | 1 | 800 |
| 168 | KEGG_TIGHT_JUNCTION | 125 | -0.242 | -1.019 | 0.416 | 0.551 | 1 | 2,860 |
| 169 | KEGG_PROTEIN_EXPORT | 20 | -0.408 | -1.023 | 0.457 | 0.552 | 1 | 4,736 |
| 170 | KEGG_ABC_TRANSPORTERS | 44 | -0.294 | -1.024 | 0.412 | 0.559 | 1 | 2,966 |
| 171 | KEGG_GNRH_SIGNALING_PATHWAY | 97 | -0.248 | -1.062 | 0.348 | 0.502 | 1 | 3,934 |
| 172 | KEGG_OOCYTE_MEIOSIS | 106 | -0.232 | -1.087 | 0.286 | 0.47 | 1 | 2,660 |
| 173 | KEGG_PATHOGENIC_ESCHERICHIA_COLI_INFECTION | 43 | -0.309 | -1.099 | 0.316 | 0.458 | 1 | 3,423 |
| 174 | KEGG_ARACHIDONIC_ACID_METABOLISM | 51 | -0.297 | -1.127 | 0.29 | 0.421 | 1 | 2,920 |
| 175 | KEGG_PHENYLALANINE_METABOLISM | 18 | -0.373 | -1.146 | 0.282 | 0.4 | 1 | 1,525 |
| 176 | KEGG_ETHER_LIPID_METABOLISM | 28 | -0.399 | -1.156 | 0.298 | 0.391 | 1 | 3,859 |
