# Supplementary material for: Signalling pathway impact analysis based on the strength of interaction between genes
Source: IET Syst Biol. 2016 Aug 1;10(4):147–52. doi: 10.1049/iet-syb.2015.0089 (PMC8687233; doi:10.1049/iet-syb.2015.0089)
Supplement: Supplementary file 2 — Supplementary Data [file SYB2-10-147-s002.docx]

Result obtained by the PSPIA methods in the lung cancer dataset

| No | Name | ID | pSize | NDE | pNDE | tA | pPERT | pG | pGFdr | pGFWER |
| --- | --- | --- | --- | --- | --- | --- | --- | --- | --- | --- |
| 1 | Pathways in cancer | 5200 | 321 | 224 | 7.76E-10 | -11.1286 | 5.00E-06 | 1.33E-13 | 1.82E-11 | 1.82E-11 |
| 2 | Protein processing in endoplasmic reticulum | 4141 | 162 | 119 | 8.84E-08 | 2.971762 | 0.008 | 1.56E-08 | 1.07E-06 | 2.14E-06 |
| 3 | Fanconi anemia pathway | 3460 | 48 | 43 | 7.03E-08 | 2.193532 | 0.09 | 1.26E-07 | 5.75E-06 | 1.72E-05 |
| 4 | Focal adhesion | 4510 | 199 | 141 | 2.45E-07 | -4.60429 | 0.082 | 3.77E-07 | 1.29E-05 | 5.16E-05 |
| 5 | Chemokine signaling pathway | 4062 | 179 | 111 | 0.010536 | -12.4931 | 5.00E-06 | 9.36E-07 | 2.56E-05 | 0.000128 |
| 6 | Cell cycle | 4110 | 122 | 90 | 2.36E-06 | -4.55216 | 0.028 | 1.16E-06 | 2.65E-05 | 0.000159 |
| 7 | Small cell lung cancer | 5222 | 83 | 66 | 5.49E-07 | -1.87643 | 0.191 | 1.79E-06 | 3.50E-05 | 0.000245 |
| 8 | HTLV-I infection | 5166 | 256 | 168 | 3.41E-05 | -5.38983 | 0.008 | 4.40E-06 | 6.92E-05 | 0.000603 |
| 9 | Melanogenesis | 4916 | 99 | 61 | 0.056518 | -4.85816 | 5.00E-06 | 4.54E-06 | 6.92E-05 | 0.000623 |
| 10 | Wnt signaling pathway | 4310 | 149 | 103 | 5.19E-05 | 3.180999 | 0.011 | 8.78E-06 | 0.00012 | 0.001203 |
| 11 | Natural killer cell mediated cytotoxicity | 4650 | 127 | 71 | 0.301567 | -11.1188 | 5.00E-06 | 2.17E-05 | 0.000271 | 0.002976 |
| 12 | Osteoclast differentiation | 4380 | 129 | 90 | 8.88E-05 | -4.6184 | 0.026 | 3.23E-05 | 0.000368 | 0.00442 |
| 13 | Vascular smooth muscle contraction | 4270 | 110 | 74 | 0.001868 | -5.86645 | 0.002 | 5.04E-05 | 0.000531 | 0.006907 |
| 14 | ECM-receptor interaction | 4512 | 83 | 59 | 0.000658 | 2.81305 | 0.008 | 6.92E-05 | 0.000677 | 0.00948 |
| 15 | Bacterial invasion of epithelial cells | 5100 | 70 | 52 | 0.000235 | -4.10623 | 0.025 | 7.67E-05 | 0.000701 | 0.01051 |
| 16 | Transcriptional misregulation in cancer | 5202 | 158 | 110 | 1.79E-05 | -0.27182 | 0.439 | 0.0001 | 0.000859 | 0.013742 |
| 17 | MAPK signaling pathway | 4010 | 259 | 162 | 0.00141 | -6.00163 | 0.006 | 0.000107 | 0.000865 | 0.014698 |
| 18 | Amoebiasis | 5146 | 106 | 68 | 0.014623 | -5.66701 | 0.001 | 0.000177 | 0.00135 | 0.024307 |
| 19 | RNA transport | 3013 | 145 | 101 | 3.76E-05 | -0.12885 | 0.77 | 0.000331 | 0.00239 | 0.045411 |
| 20 | Melanoma | 5218 | 71 | 50 | 0.002295 | -3.44163 | 0.017 | 0.000435 | 0.002873 | 0.059613 |
| 21 | Pancreatic cancer | 5212 | 69 | 52 | 0.000125 | -1.71597 | 0.316 | 0.00044 | 0.002873 | 0.060325 |
| 22 | TGF-beta signaling pathway | 4350 | 81 | 59 | 0.000229 | -2.06686 | 0.251 | 0.000618 | 0.003846 | 0.084613 |
| 23 | Tight junction | 4530 | 131 | 87 | 0.001441 | -2.24223 | 0.064 | 0.000949 | 0.005652 | 0.130003 |
| 24 | Fc gamma R-mediated phagocytosis | 4666 | 91 | 59 | 0.016287 | -5.14545 | 0.006 | 0.001 | 0.005708 | 0.137003 |
| 25 | Chagas disease (American trypanosomiasis) | 5142 | 101 | 70 | 0.0007 | -2.04232 | 0.257 | 0.001732 | 0.009165 | 0.237289 |
| 26 | Colorectal cancer | 5210 | 62 | 46 | 0.00056 | -0.93103 | 0.323 | 0.001739 | 0.009165 | 0.238288 |
| 27 | Salmonella infection | 5132 | 80 | 55 | 0.00334 | -3.59097 | 0.061 | 0.001935 | 0.009821 | 0.265159 |
| 28 | Parkinson's disease | 5012 | 116 | 80 | 0.000385 | 0.42711 | 0.574 | 0.002084 | 0.010194 | 0.285445 |
| 29 | Pertussis | 5133 | 69 | 47 | 0.008404 | -8.06404 | 0.028 | 0.002201 | 0.010399 | 0.30158 |
| 30 | Cytokine-cytokine receptor interaction | 4060 | 251 | 139 | 0.264907 | -8.18633 | 0.001 | 0.002447 | 0.011173 | 0.3352 |
| 31 | Chronic myeloid leukemia | 5220 | 72 | 50 | 0.003652 | -2.67155 | 0.079 | 0.00264 | 0.011669 | 0.361729 |
| 32 | Salivary secretion | 4970 | 82 | 51 | 0.063218 | -3.13266 | 0.005 | 0.002864 | 0.01226 | 0.392314 |
| 33 | Calcium signaling pathway | 4020 | 180 | 96 | 0.517254 | -4.91388 | 0.001 | 0.004431 | 0.018397 | 0.607088 |
| 34 | Glioma | 5214 | 64 | 45 | 0.003923 | -1.91219 | 0.172 | 0.005601 | 0.022569 | 0.767344 |
| 35 | Leukocyte transendothelial migration | 4670 | 113 | 72 | 0.015254 | -3.48857 | 0.058 | 0.007104 | 0.027094 | 0.973311 |
| 36 | Non-small cell lung cancer | 5223 | 54 | 39 | 0.003333 | 1.062095 | 0.269 | 0.007188 | 0.027094 | 0.984757 |
| 37 | Prostate cancer | 5215 | 89 | 61 | 0.002305 | -1.39305 | 0.397 | 0.007317 | 0.027094 | 1 |
| 38 | Mineral absorption | 4978 | 49 | 34 | 0.015631 | 0.09556 | 0.091 | 0.010747 | 0.038746 | 1 |
| 39 | Epstein-Barr virus infection | 5169 | 189 | 120 | 0.00262 | 0.622868 | 0.599 | 0.011703 | 0.041111 | 1 |
| 40 | Epithelial cell signaling in Helicobacter pylori infection | 5120 | 67 | 47 | 0.00348 | -0.58441 | 0.467 | 0.012062 | 0.041313 | 1 |
| 41 | Phosphatid | 4070 | 79 | 54 | 0.004389 | -0.27935 | 0.385 | 0.012475 | 0.041684 | 1 |
| 42 | Prion diseases | 5020 | 34 | 25 | 0.012477 | -0.74027 | 0.166 | 0.014871 | 0.047805 | 1 |
| 43 | Complement and coagulation cascades | 4610 | 67 | 37 | 0.418577 | -14.1077 | 0.005 | 0.015004 | 0.047805 | 1 |
| 44 | Malaria | 5144 | 47 | 30 | 0.093669 | -1.67911 | 0.023 | 0.015383 | 0.047897 | 1 |
| 45 | mTOR signaling pathway | 4150 | 62 | 44 | 0.003259 | 0.326539 | 0.719 | 0.016536 | 0.049873 | 1 |
| 46 | Axon guidance | 4360 | 128 | 82 | 0.008286 | -1.75729 | 0.287 | 0.016746 | 0.049873 | 1 |
| 47 | Hedgehog signaling pathway | 4340 | 54 | 35 | 0.056722 | 1.320181 | 0.044 | 0.017453 | 0.050874 | 1 |
| 48 | Viral carcinogenesis | 5203 | 182 | 115 | 0.004002 | -0.08894 | 0.722 | 0.019784 | 0.056468 | 1 |
| 49 | Progesterone-mediated oocyte maturation | 4914 | 84 | 57 | 0.004455 | 0.557388 | 0.711 | 0.021398 | 0.059826 | 1 |
| 50 | Cholinergic synapse | 4725 | 109 | 63 | 0.193326 | -3.21742 | 0.02 | 0.025347 | 0.06945 | 1 |
| 51 | NF-kappa B signaling pathway | 4064 | 88 | 55 | 0.049476 | -3.35799 | 0.084 | 0.026944 | 0.07238 | 1 |
| 52 | Alzheimer's disease | 5010 | 159 | 100 | 0.008406 | -0.49061 | 0.561 | 0.029977 | 0.077925 | 1 |
| 53 | Hepatitis C | 5160 | 128 | 79 | 0.031861 | 1.991095 | 0.149 | 0.030146 | 0.077925 | 1 |
| 54 | Regulation of actin cytoskeleton | 4810 | 212 | 121 | 0.143433 | -4.50687 | 0.034 | 0.030837 | 0.078234 | 1 |
| 55 | Aldosterone-regulated sodium reabsorption | 4960 | 39 | 29 | 0.005573 | 0.024136 | 0.917 | 0.032077 | 0.079901 | 1 |
| 56 | Toxoplasmosis | 5145 | 120 | 72 | 0.079824 | -2.29482 | 0.067 | 0.033325 | 0.081527 | 1 |
| 57 | Endocrine and other factor-regulated calcium reabsorption | 4961 | 49 | 28 | 0.342402 | -1.97152 | 0.017 | 0.035777 | 0.085989 | 1 |
| 58 | Vibrio cholerae infection | 5110 | 53 | 36 | 0.021116 | 0.448434 | 0.284 | 0.036681 | 0.086642 | 1 |
| 59 | Staphylococcus aureus infection | 5150 | 48 | 24 | 0.723204 | -11.0743 | 0.009 | 0.039278 | 0.091205 | 1 |
| 60 | Huntington's disease | 5016 | 171 | 105 | 0.018382 | -0.25661 | 0.371 | 0.040836 | 0.093241 | 1 |
| 61 | VEGF signa | 4370 | 71 | 42 | 0.187867 | -3.09722 | 0.041 | 0.045185 | 0.100528 | 1 |
| 62 | SNARE interactions in vesicular transport | 4130 | 35 | 26 | 0.008764 | 0.057916 | 0.894 | 0.04583 | 0.100528 | 1 |
| 63 | Sulfur relay system | 4122 | 9 | 8 | 0.030452 | 0.360763 | 0.26 | 0.046228 | 0.100528 | 1 |
| 64 | Dilated cardiomyopathy | 5414 | 90 | 53 | 0.164502 | -0.62256 | 0.056 | 0.052391 | 0.11215 | 1 |
| 65 | Adipocytokine signaling pathway | 4920 | 68 | 43 | 0.061279 | -1.67446 | 0.163 | 0.055999 | 0.117431 | 1 |
| 66 | Insulin signaling pathway | 4910 | 136 | 82 | 0.057137 | -2.47571 | 0.177 | 0.056573 | 0.117431 | 1 |
| 67 | Legionellosis | 5134 | 51 | 29 | 0.352034 | -2.7534 | 0.032 | 0.061801 | 0.126369 | 1 |
| 68 | Acute myeloid leukemia | 5221 | 56 | 35 | 0.103127 | -1.35021 | 0.121 | 0.06718 | 0.135349 | 1 |
| 69 | Measles | 5162 | 128 | 79 | 0.031861 | -1.08505 | 0.449 | 0.075063 | 0.148602 | 1 |
| 70 | Pathogenic Escherichia coli infection | 5130 | 51 | 34 | 0.035826 | -1.35102 | 0.405 | 0.075928 | 0.148602 | 1 |
| 71 | Gap junction | 4540 | 85 | 54 | 0.035054 | -0.99445 | 0.431 | 0.078451 | 0.151377 | 1 |
| 72 | Notch signaling pathway | 4330 | 47 | 30 | 0.093669 | -1.81067 | 0.17 | 0.081847 | 0.155737 | 1 |
| 73 | Basal cell carcinoma | 5217 | 54 | 35 | 0.056722 | -0.85822 | 0.32 | 0.090919 | 0.16734 | 1 |
| 74 | Amphetamine addiction | 5031 | 69 | 35 | 0.704254 | 2.420851 | 0.026 | 0.091558 | 0.16734 | 1 |
| 75 | p53 signaling pathway | 4115 | 67 | 44 | 0.026251 | 0.28301 | 0.698 | 0.09161 | 0.16734 | 1 |
| 76 | Herpes simplex infection | 5168 | 171 | 96 | 0.244066 | 2.278966 | 0.08 | 0.096378 | 0.173733 | 1 |
| 77 | Renal cell carcinoma | 5211 | 69 | 43 | 0.080415 | -1.37584 | 0.259 | 0.101461 | 0.180522 | 1 |
| 78 | Leishmaniasis | 5140 | 66 | 38 | 0.278869 | -2.03678 | 0.076 | 0.102876 | 0.180693 | 1 |
| 79 | Viral myocarditis | 5416 | 66 | 27 | 0.983405 | -1.93147 | 0.025 | 0.115688 | 0.200624 | 1 |
| 80 | Fc epsilon RI signaling pathway | 4664 | 73 | 41 | 0.349453 | -3.01132 | 0.082 | 0.13045 | 0.223396 | 1 |
| 81 | Antigen processing and presentation | 4612 | 63 | 39 | 0.103584 | -1.1164 | 0.288 | 0.134608 | 0.225203 | 1 |
| 82 | Carbohydrate digestion and absorption | 4973 | 38 | 22 | 0.339837 | -0.76811 | 0.088 | 0.134866 | 0.225203 | 1 |
| 83 | ErbB signaling pathway | 4012 | 87 | 55 | 0.037875 | -0.30921 | 0.812 | 0.137833 | 0.225203 | 1 |
| 84 | Long-term depression | 4730 | 65 | 37 | 0.31779 | -2.19023 | 0.097 | 0.138081 | 0.225203 | 1 |
| 85 | PPAR signaling pathway | 3320 | 69 | 38 | 0.425817 | -0.85191 | 0.075 | 0.141925 | 0.227586 | 1 |
| 86 | GABAergic synapse | 4727 | 87 | 47 | 0.483126 | 0.995061 | 0.067 | 0.143414 | 0.227586 | 1 |
| 87 | Alcoholism | 5034 | 128 | 78 | 0.047041 | 0.464618 | 0.695 | 0.144525 | 0.227586 | 1 |
| 88 | African trypanosomiasis | 5143 | 32 | 21 | 0.108429 | 0.357258 | 0.371 | 0.169485 | 0.263858 | 1 |
| 89 | Dorso-ventral axis formation | 4320 | 23 | 13 | 0.458381 | 0.571725 | 0.09 | 0.172773 | 0.265954 | 1 |
| 90 | Type II diabetes mellitus | 4930 | 47 | 27 | 0.332411 | 0.729739 | 0.14 | 0.189291 | 0.288143 | 1 |
| 91 | B cell receptor signaling pathway | 4662 | 75 | 47 | 0.0625 | 0.269955 | 0.785 | 0.19697 | 0.294259 | 1 |
| 92 | Shigellosis | 5131 | 60 | 37 | 0.117615 | -0.80741 | 0.421 | 0.198334 | 0.294259 | 1 |
| 93 | Bladder cancer | 5219 | 41 | 27 | 0.07011 | 0.177987 | 0.713 | 0.199753 | 0.294259 | 1 |
| 94 | Morphine addiction | 5032 | 89 | 49 | 0.404766 | -0.94667 | 0.127 | 0.203977 | 0.295415 | 1 |
| 95 | Tuberculosis | 5152 | 172 | 92 | 0.501939 | -3.78529 | 0.103 | 0.20485 | 0.295415 | 1 |
| 96 | Arrhythmogenic right ventricular cardiomyopathy (ARVC) | 5412 | 74 | 44 | 0.167918 | 0.154744 | 0.346 | 0.223427 | 0.31885 | 1 |
| 97 | Circadian rhythm | 4710 | 21 | 12 | 0.445383 | 0.845535 | 0.14 | 0.235381 | 0.331898 | 1 |
| 98 | RNA degradation | 3018 | 69 | 39 | 0.334 | 0.347782 | 0.189 | 0.23752 | 0.331898 | 1 |
| 99 | Apoptosis | 4210 | 86 | 51 | 0.152304 | -1.17182 | 0.42 | 0.239839 | 0.331898 | 1 |
| 100 | Systemic lupus erythematosus | 5322 | 81 | 39 | 0.847635 | -4.13842 | 0.082 | 0.254833 | 0.349121 | 1 |
| 101 | Jak-STAT signaling pathway | 4630 | 153 | 80 | 0.622575 | 1.132023 | 0.125 | 0.276527 | 0.375091 | 1 |
| 102 | Dopaminergic synapse | 4728 | 128 | 76 | 0.094176 | -0.1801 | 0.864 | 0.285501 | 0.383468 | 1 |
| 103 | Influenza A | 5164 | 160 | 91 | 0.196913 | -1.0008 | 0.47 | 0.312818 | 0.416078 | 1 |
| 104 | Oocyte meiosis | 4114 | 109 | 59 | 0.462411 | 3.046961 | 0.224 | 0.338439 | 0.445828 | 1 |
| 105 | Neurotrophin signaling pathway | 4722 | 119 | 66 | 0.344637 | -1.44231 | 0.315 | 0.349613 | 0.456162 | 1 |
| 106 | GnRH signaling pathway | 4912 | 94 | 50 | 0.54351 | -2.10978 | 0.217 | 0.37005 | 0.478272 | 1 |
| 107 | Vasopressin-regulated water reabsorption | 4962 | 44 | 27 | 0.175361 | 0.125842 | 0.837 | 0.428418 | 0.548536 | 1 |
| 108 | Rheumatoid arthritis | 5323 | 83 | 47 | 0.304059 | -0.33991 | 0.529 | 0.454764 | 0.573083 | 1 |
| 109 | Toll-like receptor signaling pathway | 4620 | 98 | 53 | 0.472225 | 1.812009 | 0.342 | 0.455956 | 0.573083 | 1 |
| 110 | Regulation of autophagy | 4140 | 33 | 12 | 0.983052 | -1.61885 | 0.172 | 0.469609 | 0.584876 | 1 |
| 111 | T cell receptor signaling pathway | 4660 | 108 | 60 | 0.347839 | -0.76491 | 0.5 | 0.478133 | 0.590128 | 1 |
| 112 | NOD-like receptor signaling pathway | 4621 | 57 | 34 | 0.200021 | -0.11338 | 0.899 | 0.488353 | 0.59736 | 1 |
| 113 | Endometrial cancer | 5213 | 52 | 31 | 0.215901 | -0.05702 | 0.94 | 0.526609 | 0.638455 | 1 |
| 114 | Phototransduction | 4744 | 28 | 12 | 0.90111 | 0.458062 | 0.271 | 0.588466 | 0.707192 | 1 |
| 115 | Long-term potentiation | 4720 | 69 | 31 | 0.933329 | -0.82675 | 0.292 | 0.626824 | 0.746738 | 1 |
| 116 | Retrograde endocannabinoid signaling | 4723 | 100 | 47 | 0.911055 | -0.20096 | 0.311 | 0.64066 | 0.756642 | 1 |
| 117 | Cocaine addiction | 5030 | 50 | 26 | 0.623937 | -0.57353 | 0.504 | 0.678263 | 0.794206 | 1 |
| 118 | Pancreatic secretion | 4972 | 91 | 46 | 0.731093 | -0.18119 | 0.481 | 0.719172 | 0.834971 | 1 |
| 119 | Olfactory transduction | 4740 | 112 | 21 | 1 | -0.88064 | 0.395 | 0.761903 | 0.872188 | 1 |
| 120 | Amyotrophic lateral sclerosis (ALS) | 5014 | 52 | 29 | 0.409819 | -0.01052 | 0.992 | 0.772456 | 0.872188 | 1 |
| 121 | Lysosome | 4142 | 117 | 64 | 0.409356 | 0 | 1 | 0.77498 | 0.872188 | 1 |
| 122 | Thyroid cancer | 5216 | 29 | 16 | 0.491372 | -0.06919 | 0.837 | 0.776693 | 0.872188 | 1 |
| 123 | Graft-versus-host disease | 5332 | 34 | 18 | 0.5818 | -0.02048 | 0.78 | 0.81235 | 0.904813 | 1 |
| 124 | Autoimmune thyroid disease | 5320 | 47 | 11 | 0.999993 | -0.02048 | 0.53 | 0.866483 | 0.957324 | 1 |
| 125 | Gastric acid secretion | 4971 | 73 | 36 | 0.784141 | -0.26285 | 0.702 | 0.87909 | 0.960001 | 1 |
| 126 | Type I diabetes mellitus | 4940 | 39 | 19 | 0.765545 | -0.02048 | 0.729 | 0.883583 | 0.960001 | 1 |
| 127 | Bile secretion | 4976 | 71 | 35 | 0.782871 | 0.063279 | 0.727 | 0.889928 | 0.960001 | 1 |
| 128 | Neuroactive ligand-receptor interaction | 4080 | 264 | 103 | 0.999999 | -0.1957 | 0.593 | 0.902878 | 0.965252 | 1 |
| 129 | Glutamatergic synapse | 4724 | 119 | 62 | 0.631889 | -0.04314 | 0.957 | 0.908887 | 0.965252 | 1 |
| 130 | Intestinal immune network for IgA production | 4672 | 44 | 15 | 0.996614 | -0.09674 | 0.665 | 0.935376 | 0.970658 | 1 |
| 131 | Maturity onset diabetes of the young | 4950 | 23 | 11 | 0.766578 | 0.046252 | 0.882 | 0.940744 | 0.970658 | 1 |
| 132 | Allograft rejection | 5330 | 33 | 12 | 0.983052 | -0.02048 | 0.699 | 0.944972 | 0.970658 | 1 |
| 133 | RIG-I-like receptor signaling pathway | 4622 | 70 | 33 | 0.872587 | 0.216187 | 0.793 | 0.946759 | 0.970658 | 1 |
| 134 | Cytosolic DNA-sensing pathway | 4623 | 59 | 28 | 0.84552 | 0.058434 | 0.827 | 0.949403 | 0.970658 | 1 |
| 135 | Serotonergic synapse | 4726 | 114 | 56 | 0.83429 | -0.13162 | 0.873 | 0.959213 | 0.973424 | 1 |
| 136 | Taste transduction | 4742 | 45 | 12 | 0.99992 | 0.11729 | 0.822 | 0.983111 | 0.99034 | 1 |
| 137 | Asthma | 5310 | 26 | 5 | 0.999923 | 0 | 1 | 1 | 1 | 1 |

Result obtained by the MSPIA methods in the lung cancer dataset

| No | Name | ID | pSize | NDE | pNDE | tA | pPERT | pG | pGFdr | pGFWER |
| --- | --- | --- | --- | --- | --- | --- | --- | --- | --- | --- |
| 1 | Pathways in cancer | 5200 | 321 | 224 | 7.76E-10 | -4.29922 | 0.003 | 6.47E-11 | 8.93E-09 | 8.93E-09 |
| 2 | Focal adhesion | 4510 | 199 | 141 | 2.45E-07 | -3.36883 | 0.002 | 1.10E-08 | 7.60E-07 | 1.52E-06 |
| 3 | Protein processing in endoplasmic reticulum | 4141 | 162 | 119 | 8.84E-08 | 1.036406 | 0.012 | 2.30E-08 | 1.06E-06 | 3.17E-06 |
| 4 | Fanconi anemia pathway | 3460 | 48 | 43 | 7.03E-08 | 1.338611 | 0.05 | 7.20E-08 | 2.48E-06 | 9.93E-06 |
| 5 | Small cell lung cancer | 5222 | 83 | 66 | 5.49E-07 | -0.78272 | 0.02 | 2.12E-07 | 5.86E-06 | 2.93E-05 |
| 6 | Cell cycle | 4110 | 122 | 90 | 2.36E-06 | -2.34636 | 0.013 | 5.62E-07 | 1.29E-05 | 7.76E-05 |
| 7 | Wnt signaling pathway | 4310 | 149 | 103 | 5.19E-05 | 1.242498 | 0.003 | 2.60E-06 | 5.12E-05 | 0.000358 |
| 8 | Melanogenesis | 4916 | 99 | 61 | 0.056518 | -1.43508 | 5.00E-06 | 4.54E-06 | 7.84E-05 | 0.000627 |
| 9 | HTLV-I infection | 5166 | 256 | 168 | 3.41E-05 | -1.38193 | 0.017 | 8.91E-06 | 0.000137 | 0.00123 |
| 10 | Cytokine-cytokine receptor interaction | 4060 | 251 | 139 | 0.264907 | -3.69338 | 5.00E-06 | 1.93E-05 | 0.000266 | 0.002657 |
| 11 | Vascular smooth muscle contraction | 4270 | 110 | 74 | 0.001868 | -2.45388 | 0.001 | 2.65E-05 | 0.000332 | 0.003657 |
| 12 | Calcium signaling pathway | 4020 | 180 | 96 | 0.517254 | -1.79033 | 5.00E-06 | 3.59E-05 | 0.000412 | 0.004949 |
| 13 | MAPK signaling pathway | 4010 | 259 | 162 | 0.00141 | -1.65711 | 0.004 | 7.38E-05 | 0.000761 | 0.010186 |
| 14 | ECM-receptor interaction | 4512 | 83 | 59 | 0.000658 | 1.007981 | 0.009 | 7.72E-05 | 0.000761 | 0.010647 |
| 15 | Pancreatic cancer | 5212 | 69 | 52 | 0.000125 | -1.12383 | 0.066 | 0.000105 | 0.000965 | 0.014476 |
| 16 | Bacterial invasion of epithelial cells | 5100 | 70 | 52 | 0.000235 | -1.02345 | 0.038 | 0.000113 | 0.000973 | 0.015575 |
| 17 | Chemokine signaling pathway | 4062 | 179 | 111 | 0.010536 | -5.57898 | 0.001 | 0.000131 | 0.00102 | 0.018118 |
| 18 | Transcriptional misregulation in cancer | 5202 | 158 | 110 | 1.79E-05 | -0.03135 | 0.627 | 0.000139 | 0.00102 | 0.019217 |
| 19 | Tight junction | 4530 | 131 | 87 | 0.001441 | -0.87407 | 0.008 | 0.000143 | 0.00102 | 0.019676 |
| 20 | Osteoclast differentiation | 4380 | 129 | 90 | 8.88E-05 | -0.82223 | 0.135 | 0.000148 | 0.00102 | 0.020393 |
| 21 | Amoebiasis | 5146 | 106 | 68 | 0.014623 | -1.07817 | 0.001 | 0.000177 | 0.001166 | 0.024484 |
| 22 | Melanoma | 5218 | 71 | 50 | 0.002295 | -0.81306 | 0.007 | 0.000193 | 0.001213 | 0.026693 |
| 23 | RNA transport | 3013 | 145 | 101 | 3.76E-05 | -0.20232 | 0.479 | 0.000215 | 0.001288 | 0.029635 |
| 24 | Chagas disease (American trypanosomiasis) | 5142 | 101 | 70 | 0.0007 | -0.90938 | 0.053 | 0.000416 | 0.002295 | 0.05738 |
| 25 | Chronic myeloid leukemia | 5220 | 72 | 50 | 0.003652 | -1.28172 | 0.023 | 0.000872 | 0.00463 | 0.120387 |
| 26 | Salmonella infection | 5132 | 80 | 55 | 0.00334 | -0.62914 | 0.028 | 0.000961 | 0.004913 | 0.132651 |
| 27 | TGF-beta signaling pathway | 4350 | 81 | 59 | 0.000229 | -0.38253 | 0.599 | 0.001355 | 0.006677 | 0.186966 |
| 28 | Glioma | 5214 | 64 | 45 | 0.003923 | -0.7302 | 0.037 | 0.001428 | 0.006795 | 0.197051 |
| 29 | Colorectal cancer | 5210 | 62 | 46 | 0.00056 | -0.27323 | 0.375 | 0.001988 | 0.009145 | 0.274344 |
| 30 | Parkinson's disease | 5012 | 116 | 80 | 0.000385 | 0.199264 | 0.605 | 0.002184 | 0.009721 | 0.301365 |
| 31 | Salivary secretion | 4970 | 82 | 51 | 0.063218 | -1.02909 | 0.005 | 0.002864 | 0.012349 | 0.395178 |
| 32 | Pertussis | 5133 | 69 | 47 | 0.008404 | -6.76009 | 0.049 | 0.003622 | 0.015146 | 0.499815 |
| 33 | Prostate cancer | 5215 | 89 | 61 | 0.002305 | -1.05288 | 0.191 | 0.003843 | 0.015596 | 0.530277 |
| 34 | Progesterone-mediated oocyte maturation | 4914 | 84 | 57 | 0.004455 | 1.356192 | 0.118 | 0.004495 | 0.017586 | 0.620375 |
| 35 | Alzheimer's disease | 5010 | 159 | 100 | 0.008406 | -0.37664 | 0.064 | 0.004588 | 0.017586 | 0.633108 |
| 36 | Cholinergic synapse | 4725 | 109 | 63 | 0.193326 | -1.03611 | 0.004 | 0.006314 | 0.023549 | 0.871317 |
| 37 | Vibrio cholerae infection | 5110 | 53 | 36 | 0.021116 | 0.309999 | 0.043 | 0.007268 | 0.026394 | 1 |
| 38 | Endocrine and other factor-regulated calcium reabsorption | 4961 | 49 | 28 | 0.342402 | -0.49553 | 0.003 | 0.008095 | 0.028645 | 1 |
| 39 | Complement and coagulation cascades | 4610 | 67 | 37 | 0.418577 | -13.8861 | 0.003 | 0.009644 | 0.033272 | 1 |
| 40 | Fc gamma R-mediated phagocytosis | 4666 | 91 | 59 | 0.016287 | -1.65043 | 0.091 | 0.011137 | 0.035488 | 1 |
| 41 | Non-small cell lung cancer | 5223 | 54 | 39 | 0.003333 | 0.092799 | 0.461 | 0.011491 | 0.035488 | 1 |
| 42 | Epstein-Barr virus infection | 5169 | 189 | 120 | 0.00262 | 0.25074 | 0.592 | 0.011585 | 0.035488 | 1 |
| 43 | Mineral absorption | 4978 | 49 | 34 | 0.015631 | 0.027181 | 0.101 | 0.011764 | 0.035488 | 1 |
| 44 | Malaria | 5144 | 47 | 30 | 0.093669 | -0.57596 | 0.017 | 0.011851 | 0.035488 | 1 |
| 45 | mTOR signaling pathway | 4150 | 62 | 44 | 0.003259 | 0.257175 | 0.494 | 0.011966 | 0.035488 | 1 |
| 46 | Viral carcinogenesis | 5203 | 182 | 115 | 0.004002 | -0.05081 | 0.407 | 0.012086 | 0.035488 | 1 |
| 47 | Phosphatid | 4070 | 79 | 54 | 0.004389 | -0.19833 | 0.393 | 0.012699 | 0.036508 | 1 |
| 48 | Acute myeloid leukemia | 5221 | 56 | 35 | 0.103127 | -0.42946 | 0.018 | 0.013531 | 0.038107 | 1 |
| 49 | Epithelial cell signaling in Helicobacter pylori infection | 5120 | 67 | 47 | 0.00348 | -0.05653 | 0.563 | 0.014175 | 0.039124 | 1 |
| 50 | Prion diseases | 5020 | 34 | 25 | 0.012477 | -0.2482 | 0.186 | 0.016398 | 0.044372 | 1 |
| 51 | Aldosterone-regulated sodium reabsorption | 4960 | 39 | 29 | 0.005573 | 0.032602 | 0.434 | 0.016991 | 0.045091 | 1 |
| 52 | Leukocyte transendothelial migration | 4670 | 113 | 72 | 0.015254 | -0.68067 | 0.177 | 0.018669 | 0.048609 | 1 |
| 53 | NF-kappa B signaling pathway | 4064 | 88 | 55 | 0.049476 | -1.08778 | 0.059 | 0.019956 | 0.050663 | 1 |
| 54 | Huntington's disease | 5016 | 171 | 105 | 0.018382 | -0.12459 | 0.161 | 0.020192 | 0.050663 | 1 |
| 55 | GABAergic synapse | 4727 | 87 | 47 | 0.483126 | 0.430284 | 0.008 | 0.025338 | 0.06244 | 1 |
| 56 | Regulation of actin cytoskeleton | 4810 | 212 | 121 | 0.143433 | -1.13175 | 0.028 | 0.026175 | 0.063371 | 1 |
| 57 | Sulfur relay system | 4122 | 9 | 8 | 0.030452 | 0.063904 | 0.154 | 0.029837 | 0.070993 | 1 |
| 58 | Notch signaling pathway | 4330 | 47 | 30 | 0.093669 | -0.71399 | 0.053 | 0.031303 | 0.073217 | 1 |
| 59 | Insulin signaling pathway | 4910 | 136 | 82 | 0.057137 | -1.07997 | 0.099 | 0.034929 | 0.080337 | 1 |
| 60 | Axon guidance | 4360 | 128 | 82 | 0.008286 | -0.20045 | 0.712 | 0.036183 | 0.081857 | 1 |
| 61 | Leishmaniasis | 5140 | 66 | 38 | 0.278869 | -1.01646 | 0.023 | 0.0388 | 0.086361 | 1 |
| 62 | African trypanosomiasis | 5143 | 32 | 21 | 0.108429 | 0.319998 | 0.061 | 0.039808 | 0.087198 | 1 |
| 63 | Herpes simplex infection | 5168 | 171 | 96 | 0.244066 | 0.674523 | 0.029 | 0.042119 | 0.090819 | 1 |
| 64 | SNARE interactions in vesicular transport | 4130 | 35 | 26 | 0.008764 | 0.014505 | 0.869 | 0.044764 | 0.095038 | 1 |
| 65 | Carbohydrate digestion and absorption | 4973 | 38 | 22 | 0.339837 | -0.36346 | 0.031 | 0.058501 | 0.121326 | 1 |
| 66 | Bladder cancer | 5219 | 41 | 27 | 0.07011 | 0.07695 | 0.153 | 0.059373 | 0.121326 | 1 |
| 67 | Viral myocarditis | 5416 | 66 | 27 | 0.983405 | -1.36543 | 0.011 | 0.059784 | 0.121326 | 1 |
| 68 | Pathogenic Escherichia coli infection | 5130 | 51 | 34 | 0.035826 | -0.22559 | 0.333 | 0.064765 | 0.128344 | 1 |
| 69 | ErbB signaling pathway | 4012 | 87 | 55 | 0.037875 | -0.17533 | 0.317 | 0.065102 | 0.128344 | 1 |
| 70 | B cell receptor signaling pathway | 4662 | 75 | 47 | 0.0625 | 0.246914 | 0.217 | 0.071888 | 0.138607 | 1 |
| 71 | VEGF signa | 4370 | 71 | 42 | 0.187867 | -0.9429 | 0.073 | 0.072539 | 0.138607 | 1 |
| 72 | Basal cell carcinoma | 5217 | 54 | 35 | 0.056722 | -0.33413 | 0.245 | 0.073321 | 0.138607 | 1 |
| 73 | Renal cell carcinoma | 5211 | 69 | 43 | 0.080415 | -0.68631 | 0.177 | 0.074757 | 0.139411 | 1 |
| 74 | p53 signaling pathway | 4115 | 67 | 44 | 0.026251 | -0.11162 | 0.565 | 0.077289 | 0.142212 | 1 |
| 75 | Staphylococcus aureus infection | 5150 | 48 | 24 | 0.723204 | -10.0315 | 0.021 | 0.078781 | 0.14305 | 1 |
| 76 | Dilated cardiomyopathy | 5414 | 90 | 53 | 0.164502 | -0.09745 | 0.094 | 0.079934 | 0.143258 | 1 |
| 77 | Natural killer cell mediated cytotoxicity | 4650 | 127 | 71 | 0.301567 | -3.89043 | 0.053 | 0.082093 | 0.143942 | 1 |
| 78 | Hepatitis C | 5160 | 128 | 79 | 0.031861 | 0.24521 | 0.504 | 0.082402 | 0.143942 | 1 |
| 79 | Measles | 5162 | 128 | 79 | 0.031861 | -0.22959 | 0.519 | 0.084369 | 0.14451 | 1 |
| 80 | Oocyte meiosis | 4114 | 109 | 59 | 0.462411 | 3.416317 | 0.036 | 0.084824 | 0.14451 | 1 |
| 81 | Amphetamine addiction | 5031 | 69 | 35 | 0.704254 | 0.766491 | 0.024 | 0.085868 | 0.14451 | 1 |
| 82 | Toxoplasmosis | 5145 | 120 | 72 | 0.079824 | -0.55999 | 0.231 | 0.092073 | 0.153085 | 1 |
| 83 | Gap junction | 4540 | 85 | 54 | 0.035054 | -0.19962 | 0.551 | 0.095549 | 0.156973 | 1 |
| 84 | Fc epsilon RI signaling pathway | 4664 | 73 | 41 | 0.349453 | -1.66061 | 0.059 | 0.100647 | 0.163404 | 1 |
| 85 | Cocaine addiction | 5030 | 50 | 26 | 0.623937 | 0.450123 | 0.034 | 0.102953 | 0.165204 | 1 |
| 86 | PPAR signaling pathway | 3320 | 69 | 38 | 0.425817 | -0.4379 | 0.054 | 0.10974 | 0.173093 | 1 |
| 87 | Adipocytokine signaling pathway | 4920 | 68 | 43 | 0.061279 | -0.36231 | 0.378 | 0.110378 | 0.173093 | 1 |
| 88 | Antigen processing and presentation | 4612 | 63 | 39 | 0.103584 | -0.73195 | 0.256 | 0.122775 | 0.19037 | 1 |
| 89 | Type II diabetes mellitus | 4930 | 47 | 27 | 0.332411 | 0.184101 | 0.083 | 0.126647 | 0.194192 | 1 |
| 90 | Hedgehog signaling pathway | 4340 | 54 | 35 | 0.056722 | 0.060975 | 0.515 | 0.132422 | 0.200816 | 1 |
| 91 | Shigellosis | 5131 | 60 | 37 | 0.117615 | -0.21972 | 0.262 | 0.138044 | 0.207066 | 1 |
| 92 | Legionellosis | 5134 | 51 | 29 | 0.352034 | -0.72984 | 0.09 | 0.141052 | 0.209303 | 1 |
| 93 | Jak-STAT signaling pathway | 4630 | 153 | 80 | 0.622575 | 0.310816 | 0.056 | 0.151879 | 0.222971 | 1 |
| 94 | Alcoholism | 5034 | 128 | 78 | 0.047041 | 0.062165 | 0.818 | 0.163833 | 0.237989 | 1 |
| 95 | Morphine addiction | 5032 | 89 | 49 | 0.404766 | -0.31402 | 0.115 | 0.189324 | 0.272153 | 1 |
| 96 | Long-term depression | 4730 | 65 | 37 | 0.31779 | -1.04862 | 0.168 | 0.209826 | 0.298516 | 1 |
| 97 | Apoptosis | 4210 | 86 | 51 | 0.152304 | -0.21209 | 0.37 | 0.218429 | 0.307584 | 1 |
| 98 | Systemic lupus erythematosus | 5322 | 81 | 39 | 0.847635 | -3.80136 | 0.068 | 0.222116 | 0.309616 | 1 |
| 99 | Arrhythmogenic right ventricular cardiomyopathy (ARVC) | 5412 | 74 | 44 | 0.167918 | 0.013824 | 0.355 | 0.227708 | 0.314238 | 1 |
| 100 | Toll-like receptor signaling pathway | 4620 | 98 | 53 | 0.472225 | -1.14942 | 0.128 | 0.230054 | 0.314332 | 1 |
| 101 | Dopaminergic synapse | 4728 | 128 | 76 | 0.094176 | -0.09807 | 0.778 | 0.264765 | 0.358212 | 1 |
| 102 | Endometrial cancer | 5213 | 52 | 31 | 0.215901 | -0.20284 | 0.365 | 0.279028 | 0.373844 | 1 |
| 103 | Circadian rhythm | 4710 | 21 | 12 | 0.445383 | 0.189002 | 0.205 | 0.309844 | 0.408951 | 1 |
| 104 | GnRH signaling pathway | 4912 | 94 | 50 | 0.54351 | -1.75078 | 0.169 | 0.311159 | 0.408951 | 1 |
| 105 | Retrograde endocannabinoid signaling | 4723 | 100 | 47 | 0.911055 | -0.14686 | 0.106 | 0.322305 | 0.419605 | 1 |
| 106 | Dorso-ventral axis formation | 4320 | 23 | 13 | 0.458381 | 0.054638 | 0.219 | 0.331145 | 0.427084 | 1 |
| 107 | Vasopressin-regulated water reabsorption | 4962 | 44 | 27 | 0.175361 | -0.02515 | 0.628 | 0.353079 | 0.451157 | 1 |
| 108 | Pancreatic secretion | 4972 | 91 | 46 | 0.731093 | -0.15688 | 0.164 | 0.374218 | 0.47378 | 1 |
| 109 | NOD-like receptor signaling pathway | 4621 | 57 | 34 | 0.200021 | -0.07955 | 0.624 | 0.384541 | 0.482424 | 1 |
| 110 | Long-term potentiation | 4720 | 69 | 31 | 0.933329 | -0.21543 | 0.142 | 0.400372 | 0.495248 | 1 |
| 111 | Influenza A | 5164 | 160 | 91 | 0.196913 | 0.200315 | 0.677 | 0.401941 | 0.495248 | 1 |
| 112 | RNA degradation | 3018 | 69 | 39 | 0.334 | 0.028405 | 0.409 | 0.408542 | 0.498927 | 1 |
| 113 | Phototransduction | 4744 | 28 | 12 | 0.90111 | 0.162819 | 0.155 | 0.414611 | 0.501897 | 1 |
| 114 | Neurotrophin signaling pathway | 4722 | 119 | 66 | 0.344637 | -0.22924 | 0.588 | 0.526129 | 0.631355 | 1 |
| 115 | Tuberculosis | 5152 | 172 | 92 | 0.501939 | -0.57704 | 0.415 | 0.535083 | 0.636565 | 1 |
| 116 | Amyotrophic lateral sclerosis (ALS) | 5014 | 52 | 29 | 0.409819 | 0.275321 | 0.516 | 0.540019 | 0.636946 | 1 |
| 117 | Rheumatoid arthritis | 5323 | 83 | 47 | 0.304059 | -0.06992 | 0.712 | 0.547766 | 0.640607 | 1 |
| 118 | T cell receptor signaling pathway | 4660 | 108 | 60 | 0.347839 | -0.12405 | 0.661 | 0.56791 | 0.658584 | 1 |
| 119 | Bile secretion | 4976 | 71 | 35 | 0.782871 | -0.04543 | 0.345 | 0.623638 | 0.713697 | 1 |
| 120 | Thyroid cancer | 5216 | 29 | 16 | 0.491372 | -0.03523 | 0.553 | 0.625778 | 0.713697 | 1 |
| 121 | RIG-I-like receptor signaling pathway | 4622 | 70 | 33 | 0.872587 | 0.090819 | 0.416 | 0.730843 | 0.826691 | 1 |
| 122 | Autoimmune thyroid disease | 5320 | 47 | 11 | 0.999993 | -0.00416 | 0.388 | 0.755336 | 0.842729 | 1 |
| 123 | Graft-versus-host disease | 5332 | 34 | 18 | 0.5818 | -0.00416 | 0.673 | 0.758685 | 0.842729 | 1 |
| 124 | Gastric acid secretion | 4971 | 73 | 36 | 0.784141 | -0.0968 | 0.519 | 0.772842 | 0.842729 | 1 |
| 125 | Lysosome | 4142 | 117 | 64 | 0.409356 | 0 | 1 | 0.77498 | 0.842729 | 1 |
| 126 | Neuroactive ligand-receptor interaction | 4080 | 264 | 103 | 0.999999 | -0.04726 | 0.41 | 0.775555 | 0.842729 | 1 |
| 127 | Glutamatergic synapse | 4724 | 119 | 62 | 0.631889 | -0.07688 | 0.79 | 0.846013 | 0.887517 | 1 |
| 128 | Olfactory transduction | 4740 | 112 | 21 | 1 | -0.22827 | 0.502 | 0.847956 | 0.887517 | 1 |
| 129 | Regulation of autophagy | 4140 | 33 | 12 | 0.983052 | -0.18586 | 0.518 | 0.852881 | 0.887517 | 1 |
| 130 | Serotonergic synapse | 4726 | 114 | 56 | 0.83429 | -0.1298 | 0.612 | 0.8538 | 0.887517 | 1 |
| 131 | Cytosolic DNA-sensing pathway | 4623 | 59 | 28 | 0.84552 | 0.0218 | 0.604 | 0.853873 | 0.887517 | 1 |
| 132 | Type I diabetes mellitus | 4940 | 39 | 19 | 0.765545 | -0.00416 | 0.67 | 0.85536 | 0.887517 | 1 |
| 133 | Allograft rejection | 5330 | 33 | 12 | 0.983052 | -0.00416 | 0.539 | 0.8664 | 0.892262 | 1 |
| 134 | Maturity onset diabetes of the young | 4950 | 23 | 11 | 0.766578 | 0.002192 | 0.964 | 0.96251 | 0.978923 | 1 |
| 135 | Intestinal immune network for IgA production | 4672 | 44 | 15 | 0.996614 | -0.00245 | 0.749 | 0.964736 | 0.978923 | 1 |
| 136 | Taste transduction | 4742 | 45 | 12 | 0.99992 | 0.013171 | 0.826 | 0.983886 | 0.991068 | 1 |
| 137 | Asthma | 5310 | 26 | 5 | 0.999923 | 0 | 1 | 1 | 1 | 1 |

Result obtained by the SPIA methods in the lung cancer dataset

|  | Name | ID | pSize | NDE | pNDE | tA | pPERT | pG | pGFdr | pGFWER |
| --- | --- | --- | --- | --- | --- | --- | --- | --- | --- | --- |
| 1 | Pathways in cancer | 5200 | 321 | 224 | 7.76E-10 | -96.6053 | 0.003 | 6.47E-11 | 8.86E-09 | 8.86E-09 |
| 2 | Focal adhesion | 4510 | 199 | 141 | 2.45E-07 | -65.2 | 0.002 | 1.10E-08 | 7.54E-07 | 1.51E-06 |
| 3 | Fanconi anemia pathway | 3460 | 48 | 43 | 7.03E-08 | 5.920218 | 0.048 | 6.92E-08 | 3.16E-06 | 9.49E-06 |
| 4 | Small cell lung cancer | 5222 | 83 | 66 | 5.49E-07 | -32.7872 | 0.009 | 9.95E-08 | 3.19E-06 | 1.36E-05 |
| 5 | MAPK signaling pathway | 4010 | 259 | 162 | 0.00141 | -42.1789 | 5.00E-06 | 1.39E-07 | 3.19E-06 | 1.91E-05 |
| 6 | Protein processing in endoplasmic reticulum | 4141 | 162 | 119 | 8.84E-08 | 10.23397 | 0.08 | 1.40E-07 | 3.19E-06 | 1.92E-05 |
| 7 | Vascular smooth muscle contraction | 4270 | 110 | 74 | 0.001868 | -53.0945 | 5.00E-06 | 1.82E-07 | 3.56E-06 | 2.49E-05 |
| 8 | Chemokine signaling pathway | 4062 | 179 | 111 | 0.010536 | -78.1946 | 5.00E-06 | 9.36E-07 | 1.60E-05 | 0.000128 |
| 9 | Salivary secretion | 4970 | 82 | 51 | 0.063218 | -17.7315 | 5.00E-06 | 5.05E-06 | 7.36E-05 | 0.000691 |
| 10 | Cell cycle | 4110 | 122 | 90 | 2.36E-06 | -17.8527 | 0.143 | 5.38E-06 | 7.36E-05 | 0.000736 |
| 11 | Wnt signaling pathway | 4310 | 149 | 103 | 5.19E-05 | 19.70119 | 0.037 | 2.72E-05 | 0.000339 | 0.003728 |
| 12 | HTLV-I infection | 5166 | 256 | 168 | 3.41E-05 | -23.4154 | 0.07 | 3.33E-05 | 0.000378 | 0.004566 |
| 13 | Calcium signaling pathway | 4020 | 180 | 96 | 0.517254 | -32.5844 | 5.00E-06 | 3.59E-05 | 0.000378 | 0.004913 |
| 14 | Osteoclast differentiation | 4380 | 129 | 90 | 8.88E-05 | -24.7194 | 0.032 | 3.91E-05 | 0.000383 | 0.005359 |
| 15 | Bacterial invasion of epithelial cells | 5100 | 70 | 52 | 0.000235 | -40.3999 | 0.016 | 5.08E-05 | 0.000464 | 0.006956 |
| 16 | Transcriptional misregulation in cancer | 5202 | 158 | 110 | 1.79E-05 | -0.90839 | 0.337 | 7.86E-05 | 0.000673 | 0.010768 |
| 17 | TGF-beta signaling pathway | 4350 | 81 | 59 | 0.000229 | 17.57903 | 0.085 | 0.00023 | 0.001855 | 0.031536 |
| 18 | RNA transport | 3013 | 145 | 101 | 3.76E-05 | 0.62923 | 0.705 | 0.000306 | 0.002328 | 0.041898 |
| 19 | ECM-receptor interaction | 4512 | 83 | 59 | 0.000658 | 8.371353 | 0.079 | 0.000564 | 0.00407 | 0.077322 |
| 20 | Pancreatic cancer | 5212 | 69 | 52 | 0.000125 | 6.234982 | 0.484 | 0.000649 | 0.004443 | 0.08886 |
| 21 | Colorectal cancer | 5210 | 62 | 46 | 0.00056 | -6.61721 | 0.175 | 0.001002 | 0.00654 | 0.13733 |
| 22 | Melanoma | 5218 | 71 | 50 | 0.002295 | -33.4063 | 0.085 | 0.001862 | 0.011593 | 0.255046 |
| 23 | Parkinson's disease | 5012 | 116 | 80 | 0.000385 | 3.676272 | 0.582 | 0.002109 | 0.012565 | 0.288998 |
| 24 | Pertussis | 5133 | 69 | 47 | 0.008404 | -17.3402 | 0.036 | 0.002754 | 0.015722 | 0.377329 |
| 25 | Fc gamma R-mediated phagocytosis | 4666 | 91 | 59 | 0.016287 | -26.9151 | 0.021 | 0.003072 | 0.016832 | 0.42081 |
| 26 | Amoebiasis | 5146 | 106 | 68 | 0.014623 | -10.8011 | 0.026 | 0.003374 | 0.01778 | 0.462271 |
| 27 | Chagas disease (American trypanosomiasis) | 5142 | 101 | 70 | 0.0007 | -3.88293 | 0.734 | 0.004407 | 0.022363 | 0.603795 |
| 28 | Epstein-Barr virus infection | 5169 | 189 | 120 | 0.00262 | 7.196447 | 0.275 | 0.005934 | 0.028371 | 0.812941 |
| 29 | Tight junction | 4530 | 131 | 87 | 0.001441 | -3.93374 | 0.507 | 0.006006 | 0.028371 | 0.822759 |
| 30 | Cytokine-cytokine receptor interaction | 4060 | 251 | 139 | 0.264907 | -24.2878 | 0.003 | 0.006467 | 0.029533 | 0.885988 |
| 31 | Leukocyte transendothelial migration | 4670 | 113 | 72 | 0.015254 | -28.5019 | 0.058 | 0.007104 | 0.031397 | 0.973311 |
| 32 | Salmonella infection | 5132 | 80 | 55 | 0.00334 | -7.40482 | 0.28 | 0.007459 | 0.031933 | 1 |
| 33 | Dilated cardiomyopathy | 5414 | 90 | 53 | 0.164502 | -6.87634 | 0.006 | 0.007818 | 0.032456 | 1 |
| 34 | mTOR signaling pathway | 4150 | 62 | 44 | 0.003259 | -3.14365 | 0.353 | 0.008937 | 0.036012 | 1 |
| 35 | Prostate cancer | 5215 | 89 | 61 | 0.002305 | -8.24648 | 0.573 | 0.010077 | 0.039443 | 1 |
| 36 | Axon guidance | 4360 | 128 | 82 | 0.008286 | -13.1104 | 0.168 | 0.010548 | 0.039794 | 1 |
| 37 | Mineral absorption | 4978 | 49 | 34 | 0.015631 | 1.033856 | 0.091 | 0.010747 | 0.039794 | 1 |
| 38 | Phosphatid | 4070 | 79 | 54 | 0.004389 | -1.18541 | 0.357 | 0.011686 | 0.041537 | 1 |
| 39 | Hedgehog signaling pathway | 4340 | 54 | 35 | 0.056722 | 14.14377 | 0.028 | 0.011824 | 0.041537 | 1 |
| 40 | Glioma | 5214 | 64 | 45 | 0.003923 | -10.525 | 0.469 | 0.013427 | 0.045988 | 1 |
| 41 | Chronic myeloid leukemia | 5220 | 72 | 50 | 0.003652 | -5.23787 | 0.561 | 0.014733 | 0.049231 | 1 |
| 42 | Epithelial cell signaling in Helicobacter pylori infection | 5120 | 67 | 47 | 0.00348 | -3.19731 | 0.615 | 0.015296 | 0.049893 | 1 |
| 43 | Non-small cell lung cancer | 5223 | 54 | 39 | 0.003333 | 4.665458 | 0.732 | 0.017117 | 0.054537 | 1 |
| 44 | Staphylococcus aureus infection | 5150 | 48 | 24 | 0.723204 | -20.9961 | 0.004 | 0.019803 | 0.05998 | 1 |
| 45 | Viral carcinogenesis | 5203 | 182 | 115 | 0.004002 | -0.42643 | 0.73 | 0.019971 | 0.05998 | 1 |
| 46 | Alzheimer's disease | 5010 | 159 | 100 | 0.008406 | -5.76804 | 0.351 | 0.020139 | 0.05998 | 1 |
| 47 | Regulation of actin cytoskeleton | 4810 | 212 | 121 | 0.143433 | -37.7454 | 0.022 | 0.021327 | 0.062166 | 1 |
| 48 | Aldosterone-regulated sodium reabsorption | 4960 | 39 | 29 | 0.005573 | -1.99584 | 0.617 | 0.022945 | 0.06549 | 1 |
| 49 | Progesterone-mediated oocyte maturation | 4914 | 84 | 57 | 0.004455 | -1.1919 | 0.858 | 0.025103 | 0.069851 | 1 |
| 50 | Prion diseases | 5020 | 34 | 25 | 0.012477 | -5.55486 | 0.312 | 0.025493 | 0.069851 | 1 |
| 51 | Melanogenesis | 4916 | 99 | 61 | 0.056518 | -42.959 | 0.073 | 0.026779 | 0.071934 | 1 |
| 52 | NF-kappa B signaling pathway | 4064 | 88 | 55 | 0.049476 | -16.1526 | 0.099 | 0.030951 | 0.081544 | 1 |
| 53 | Malaria | 5144 | 47 | 30 | 0.093669 | -3.05462 | 0.056 | 0.032786 | 0.084749 | 1 |
| 54 | Cholinergic synapse | 4725 | 109 | 63 | 0.193326 | -23.4781 | 0.033 | 0.038627 | 0.097998 | 1 |
| 55 | SNARE interactions in vesicular transport | 4130 | 35 | 26 | 0.008764 | 0.677509 | 0.745 | 0.039382 | 0.098097 | 1 |
| 56 | p53 signaling pathway | 4115 | 67 | 44 | 0.026251 | 4.001227 | 0.336 | 0.050547 | 0.12366 | 1 |
| 57 | Natural killer cell mediated cytotoxicity | 4650 | 127 | 71 | 0.301567 | -58.7003 | 0.031 | 0.05303 | 0.127458 | 1 |
| 58 | Hepatitis C | 5160 | 128 | 79 | 0.031861 | 6.792011 | 0.339 | 0.059708 | 0.139508 | 1 |
| 59 | Complement and coagulation cascades | 4610 | 67 | 37 | 0.418577 | -30.4145 | 0.026 | 0.06008 | 0.139508 | 1 |
| 60 | Adipocytokine signaling pathway | 4920 | 68 | 43 | 0.061279 | -7.08971 | 0.205 | 0.067548 | 0.154234 | 1 |
| 61 | Type II diabetes mellitus | 4930 | 47 | 27 | 0.332411 | 13.59099 | 0.039 | 0.0693 | 0.155641 | 1 |
| 62 | Sulfur relay system | 4122 | 9 | 8 | 0.030452 | 1.255652 | 0.501 | 0.079071 | 0.171179 | 1 |
| 63 | African trypanosomiasis | 5143 | 32 | 21 | 0.108429 | 2.864984 | 0.143 | 0.080109 | 0.171179 | 1 |
| 64 | Endocrine and other factor-regulated calcium reabsorption | 4961 | 49 | 28 | 0.342402 | -10.3236 | 0.046 | 0.081129 | 0.171179 | 1 |
| 65 | Huntington's disease | 5016 | 171 | 105 | 0.018382 | -0.56154 | 0.858 | 0.081216 | 0.171179 | 1 |
| 66 | Gap junction | 4540 | 85 | 54 | 0.035054 | -7.54034 | 0.489 | 0.086844 | 0.178964 | 1 |
| 67 | ErbB signaling pathway | 4012 | 87 | 55 | 0.037875 | -11.2556 | 0.457 | 0.087523 | 0.178964 | 1 |
| 68 | Vibrio cholerae infection | 5110 | 53 | 36 | 0.021116 | 0.415521 | 0.859 | 0.09087 | 0.183076 | 1 |
| 69 | Measles | 5162 | 128 | 79 | 0.031861 | -4.73145 | 0.608 | 0.095771 | 0.190155 | 1 |
| 70 | Acute myeloid leukemia | 5221 | 56 | 35 | 0.103127 | -7.25909 | 0.224 | 0.110141 | 0.215561 | 1 |
| 71 | Toxoplasmosis | 5145 | 120 | 72 | 0.079824 | -8.92325 | 0.302 | 0.113912 | 0.219801 | 1 |
| 72 | Long-term depression | 4730 | 65 | 37 | 0.31779 | -16.379 | 0.081 | 0.119945 | 0.228228 | 1 |
| 73 | Pathogenic Escherichia coli infection | 5130 | 51 | 34 | 0.035826 | -2.08177 | 0.793 | 0.129579 | 0.243182 | 1 |
| 74 | Basal cell carcinoma | 5217 | 54 | 35 | 0.056722 | -6.844 | 0.522 | 0.133822 | 0.247752 | 1 |
| 75 | Renal cell carcinoma | 5211 | 69 | 43 | 0.080415 | -4.72004 | 0.452 | 0.156827 | 0.28647 | 1 |
| 76 | Notch signaling pathway | 4330 | 47 | 30 | 0.093669 | -7.0774 | 0.404 | 0.16175 | 0.291576 | 1 |
| 77 | Shigellosis | 5131 | 60 | 37 | 0.117615 | -6.53317 | 0.364 | 0.17771 | 0.312647 | 1 |
| 78 | Morphine addiction | 5032 | 89 | 49 | 0.404766 | -7.36864 | 0.106 | 0.178004 | 0.312647 | 1 |
| 79 | Systemic lupus erythematosus | 5322 | 81 | 39 | 0.847635 | -6.68362 | 0.052 | 0.181677 | 0.313961 | 1 |
| 80 | VEGF signa | 4370 | 71 | 42 | 0.187867 | -11.3676 | 0.241 | 0.185404 | 0.313961 | 1 |
| 81 | Alcoholism | 5034 | 128 | 78 | 0.047041 | 0.632585 | 0.964 | 0.185627 | 0.313961 | 1 |
| 82 | Leishmaniasis | 5140 | 66 | 38 | 0.278869 | -8.65548 | 0.171 | 0.192802 | 0.32212 | 1 |
| 83 | B cell receptor signaling pathway | 4662 | 75 | 47 | 0.0625 | -2.02604 | 0.814 | 0.202401 | 0.333246 | 1 |
| 84 | Phototransduction | 4744 | 28 | 12 | 0.90111 | 8.482256 | 0.058 | 0.20652 | 0.333246 | 1 |
| 85 | Toll-like receptor signaling pathway | 4620 | 98 | 53 | 0.472225 | 15.41287 | 0.112 | 0.20836 | 0.333246 | 1 |
| 86 | Dorso-ventral axis formation | 4320 | 23 | 13 | 0.458381 | 1.851116 | 0.116 | 0.209191 | 0.333246 | 1 |
| 87 | Apoptosis | 4210 | 86 | 51 | 0.152304 | -10.9198 | 0.36 | 0.214028 | 0.333704 | 1 |
| 88 | Insulin signaling pathway | 4910 | 136 | 82 | 0.057137 | 0.740435 | 0.97 | 0.215749 | 0.333704 | 1 |
| 89 | PPAR signaling pathway | 3320 | 69 | 38 | 0.425817 | -2.7231 | 0.131 | 0.216786 | 0.333704 | 1 |
| 90 | Bladder cancer | 5219 | 41 | 27 | 0.07011 | 0.212294 | 0.972 | 0.251197 | 0.382377 | 1 |
| 91 | RNA degradation | 3018 | 69 | 39 | 0.334 | 1.74739 | 0.209 | 0.255632 | 0.384853 | 1 |
| 92 | Legionellosis | 5134 | 51 | 29 | 0.352034 | -6.4573 | 0.204 | 0.260951 | 0.38859 | 1 |
| 93 | Arrhythmogenic right ventricular cardiomyopathy (ARVC) | 5412 | 74 | 44 | 0.167918 | 0.568183 | 0.46 | 0.275045 | 0.401761 | 1 |
| 94 | Carbohydrate digestion and absorption | 4973 | 38 | 22 | 0.339837 | -2.61119 | 0.228 | 0.275661 | 0.401761 | 1 |
| 95 | Antigen processing and presentation | 4612 | 63 | 39 | 0.103584 | -0.84535 | 0.768 | 0.280927 | 0.405127 | 1 |
| 96 | GABAergic synapse | 4727 | 87 | 47 | 0.483126 | 3.607279 | 0.172 | 0.289823 | 0.413601 | 1 |
| 97 | Jak-STAT signaling pathway | 4630 | 153 | 80 | 0.622575 | 4.918986 | 0.143 | 0.30437 | 0.426343 | 1 |
| 98 | Dopaminergic synapse | 4728 | 128 | 76 | 0.094176 | 0.326731 | 0.948 | 0.304975 | 0.426343 | 1 |
| 99 | Amphetamine addiction | 5031 | 69 | 35 | 0.704254 | 7.285163 | 0.135 | 0.318793 | 0.441159 | 1 |
| 100 | Neurotrophin signaling pathway | 4722 | 119 | 66 | 0.344637 | -13.6245 | 0.282 | 0.323743 | 0.443527 | 1 |
| 101 | Influenza A | 5164 | 160 | 91 | 0.196913 | -4.80105 | 0.538 | 0.343761 | 0.46629 | 1 |
| 102 | Herpes simplex infection | 5168 | 171 | 96 | 0.244066 | 6.795486 | 0.45 | 0.352424 | 0.473354 | 1 |
| 103 | Circadian rhythm | 4710 | 21 | 12 | 0.445383 | 3.882872 | 0.262 | 0.367368 | 0.487301 | 1 |
| 104 | Endometrial cancer | 5213 | 52 | 31 | 0.215901 | 5.15542 | 0.546 | 0.369922 | 0.487301 | 1 |
| 105 | NOD-like receptor signaling pathway | 4621 | 57 | 34 | 0.200021 | -3.15183 | 0.639 | 0.390749 | 0.509834 | 1 |
| 106 | Fc epsilon RI signaling pathway | 4664 | 73 | 41 | 0.349453 | -9.99512 | 0.398 | 0.413448 | 0.534362 | 1 |
| 107 | GnRH signaling pathway | 4912 | 94 | 50 | 0.54351 | -17.4273 | 0.272 | 0.430445 | 0.55113 | 1 |
| 108 | Vasopressin-regulated water reabsorption | 4962 | 44 | 27 | 0.175361 | 0.564519 | 0.874 | 0.440727 | 0.55907 | 1 |
| 109 | Rheumatoid arthritis | 5323 | 83 | 47 | 0.304059 | -1.12038 | 0.552 | 0.467393 | 0.587457 | 1 |
| 110 | Amyotrophic lateral sclerosis (ALS) | 5014 | 52 | 29 | 0.409819 | -4.90785 | 0.421 | 0.475703 | 0.592467 | 1 |
| 111 | Cocaine addiction | 5030 | 50 | 26 | 0.623937 | -4.4523 | 0.298 | 0.498741 | 0.615564 | 1 |
| 112 | Tuberculosis | 5152 | 172 | 92 | 0.501939 | -12.7134 | 0.396 | 0.519901 | 0.63595 | 1 |
| 113 | T cell receptor signaling pathway | 4660 | 108 | 60 | 0.347839 | -4.42038 | 0.646 | 0.56018 | 0.679157 | 1 |
| 114 | Regulation of autophagy | 4140 | 33 | 12 | 0.983052 | -3.99091 | 0.233 | 0.566629 | 0.680949 | 1 |
| 115 | Viral myocarditis | 5416 | 66 | 27 | 0.983405 | -3.57288 | 0.312 | 0.669329 | 0.797374 | 1 |
| 116 | Thyroid cancer | 5216 | 29 | 16 | 0.491372 | 1.291357 | 0.705 | 0.713658 | 0.842122 | 1 |
| 117 | Retrograde endocannabinoid signaling | 4723 | 100 | 47 | 0.911055 | -1.4782 | 0.386 | 0.719184 | 0.842122 | 1 |
| 118 | Gastric acid secretion | 4971 | 73 | 36 | 0.784141 | -2.83251 | 0.475 | 0.740318 | 0.859522 | 1 |
| 119 | Long-term potentiation | 4720 | 69 | 31 | 0.933329 | -10.0494 | 0.407 | 0.747551 | 0.860626 | 1 |
| 120 | Graft-versus-host disease | 5332 | 34 | 18 | 0.5818 | -0.27911 | 0.699 | 0.77258 | 0.877457 | 1 |
| 121 | Lysosome | 4142 | 117 | 64 | 0.409356 | 0 | 1 | 0.77498 | 0.877457 | 1 |
| 122 | Oocyte meiosis | 4114 | 109 | 59 | 0.462411 | 1.324433 | 0.92 | 0.789016 | 0.886026 | 1 |
| 123 | Pancreatic secretion | 4972 | 91 | 46 | 0.731093 | -0.79493 | 0.647 | 0.827129 | 0.916868 | 1 |
| 124 | RIG-I-like receptor signaling pathway | 4622 | 70 | 33 | 0.872587 | 2.850211 | 0.55 | 0.832248 | 0.916868 | 1 |
| 125 | Autoimmune thyroid disease | 5320 | 47 | 11 | 0.999993 | -0.27911 | 0.499 | 0.845877 | 0.916868 | 1 |
| 126 | Glutamatergic synapse | 4724 | 119 | 62 | 0.631889 | -1.15911 | 0.799 | 0.849932 | 0.916868 | 1 |
| 127 | Type I diabetes mellitus | 4940 | 39 | 19 | 0.765545 | -0.27911 | 0.661 | 0.850714 | 0.916868 | 1 |
| 128 | Intestinal immune network for IgA production | 4672 | 44 | 15 | 0.996614 | -0.67362 | 0.536 | 0.869126 | 0.916868 | 1 |
| 129 | Bile secretion | 4976 | 71 | 35 | 0.782871 | 0.750133 | 0.684 | 0.869939 | 0.916868 | 1 |
| 130 | Serotonergic synapse | 4726 | 114 | 56 | 0.83429 | 2.472818 | 0.642 | 0.87002 | 0.916868 | 1 |
| 131 | Allograft rejection | 5330 | 33 | 12 | 0.983052 | -0.27911 | 0.685 | 0.939669 | 0.982708 | 1 |
| 132 | Maturity onset diabetes of the young | 4950 | 23 | 11 | 0.766578 | -0.13802 | 0.963 | 0.962278 | 0.993362 | 1 |
| 133 | Neuroactive ligand-receptor interaction | 4080 | 264 | 103 | 0.999999 | -0.71865 | 0.749 | 0.965473 | 0.993362 | 1 |
| 134 | Taste transduction | 4742 | 45 | 12 | 0.99992 | 1.021429 | 0.786 | 0.975252 | 0.993362 | 1 |
| 135 | Cytosolic DNA-sensing pathway | 4623 | 59 | 28 | 0.84552 | 0.125288 | 0.948 | 0.97886 | 0.993362 | 1 |
| 136 | Olfactory transduction | 4740 | 112 | 21 | 1 | -0.02475 | 0.993 | 0.999975 | 1 | 1 |
| 137 | Asthma | 5310 | 26 | 5 | 0.999923 | 0 | 1 | 1 | 1 | 1 |

Result obtained by the BPA methods in the lung cancer dataset

| No | ID and Name | Score | p-value | FDR | FWER |
| --- | --- | --- | --- | --- | --- |
| 1 | hsa03320 PPAR signaling pathway - Homo sapiens (human) | -8162.68 | 0.001 | 0.004659 | 0.015707 |
| 2 | hsa04110 Cell cycle - Homo sapiens (human) | -6914.19 | 0.001 | 0.004659 | 0.151832 |
| 3 | hsa04210 Apoptosis - Homo sapiens (human) | -18271.4 | 0.001 | 0.004659 | 0.528796 |
| 4 | hsa04360 Axon guidance - Homo sapiens (human) | -19281 | 0.001 | 0.004659 | 0.120419 |
| 5 | hsa04510 Focal adhesion - Homo sapiens (human) | -21203.6 | 0.001 | 0.004659 | 0.198953 |
| 6 | hsa04514 Cell adhesion molecules (CAMs) - Homo sapiens (human) | -28476.8 | 0.001 | 0.004659 | 0.361257 |
| 7 | hsa04530 Tight junction - Homo sapiens (human) | -19793.7 | 0.001 | 0.004659 | 0.418848 |
| 8 | hsa04610 Complement and coagulation cascades - Homo sapiens (human) | -20311.1 | 0.001 | 0.004659 | 0.324607 |
| 9 | hsa04612 Antigen processing and presentation - Homo sapiens (human) | -10045.3 | 0.001 | 0.004659 | 0.65445 |
| 10 | hsa04660 T cell receptor signaling pathway - Homo sapiens (human) | -20913 | 0.001 | 0.004659 | 0.52356 |
| 11 | hsa04664 Fc epsilon RI signaling pathway - Homo sapiens (human) | -13153.6 | 0.001 | 0.004659 | 0.424084 |
| 12 | hsa04670 Leukocyte transendothelial migration - Homo sapiens (human) | -7990.84 | 0.001 | 0.004659 | 0.303665 |
| 13 | hsa04710 Circadian rhythm - Homo sapiens (human) | -2623 | 0.001 | 0.004659 | 0.251309 |
| 14 | hsa04720 Long-term potentiation - Homo sapiens (human) | -9060.58 | 0.001 | 0.004659 | 0.162304 |
| 15 | hsa04910 Insulin signaling pathway - Homo sapiens (human) | -23512.5 | 0.001 | 0.004659 | 0.329843 |
| 16 | hsa04916 Melanogenesis - Homo sapiens (human) | -7365.85 | 0.001 | 0.004659 | 0.031414 |
| 17 | hsa04920 Adipocytokine signaling pathway - Homo sapiens (human) | -9364.92 | 0.001 | 0.004659 | 0.041885 |
| 18 | hsa05110 Vibrio cholerae infection - Homo sapiens (human) | -6405.74 | 0.001 | 0.004659 | 0.198953 |
| 19 | hsa05211 Renal cell carcinoma - Homo sapiens (human) | -14052.3 | 0.001 | 0.004659 | 0.371728 |
| 20 | hsa05215 Prostate cancer - Homo sapiens (human) | -15208.9 | 0.001 | 0.004659 | 0.376963 |
| 21 | hsa05217 Basal cell carcinoma - Homo sapiens (human) | -5450.68 | 0.001 | 0.004659 | 0.109948 |
| 22 | hsa03430 Mismatch repair - Homo sapiens (human) | -5217.81 | 0.002 | 0.00764 | 0.963351 |
| 23 | hsa04512 ECM-receptor interaction - Homo sapiens (human) | -16423.8 | 0.002 | 0.00764 | 0.34555 |
| 24 | hsa05330 Allograft rejection - Homo sapiens (human) | -6973.01 | 0.002 | 0.00764 | 0.497382 |
| 25 | hsa04350 TGF-beta signaling pathway - Homo sapiens (human) | -15184.5 | 0.003 | 0.009242 | 0.319372 |
| 26 | hsa04620 Toll-like receptor signaling pathway - Homo sapiens (human) | -23898.4 | 0.003 | 0.009242 | 0.617801 |
| 27 | hsa04662 B cell receptor signaling pathway - Homo sapiens (human) | -11735.2 | 0.003 | 0.009242 | 0.17801 |
| 28 | hsa05210 Colorectal cancer - Homo sapiens (human) | -16302 | 0.003 | 0.009242 | 0.65445 |
| 29 | hsa05212 Pancreatic cancer - Homo sapiens (human) | -14648.4 | 0.003 | 0.009242 | 0.413613 |
| 30 | hsa04010 MAPK signaling pathway - Homo sapiens (human) | -44999.9 | 0.004 | 0.011576 | 0.52356 |
| 31 | hsa04650 Natural killer cell mediated cytotoxicity - Homo sapiens (human) | -19883.2 | 0.004 | 0.011576 | 0.099476 |
| 32 | hsa05012 Parkinson's disease - Homo sapiens (human) | -10574 | 0.004 | 0.011576 | 0.780105 |
| 33 | hsa05223 Non-small cell lung cancer - Homo sapiens (human) | -10871.1 | 0.004 | 0.011576 | 0.188482 |
| 34 | hsa04115 p53 signaling pathway - Homo sapiens (human) | -20721.9 | 0.005 | 0.012089 | 0.602094 |
| 35 | hsa04310 Wnt signaling pathway - Homo sapiens (human) | -21485.2 | 0.005 | 0.012089 | 0.319372 |
| 36 | hsa04540 Gap junction - Homo sapiens (human) | -12409 | 0.005 | 0.012089 | 0.293194 |
| 37 | hsa04912 GnRH signaling pathway - Homo sapiens (human) | -16023.9 | 0.005 | 0.012089 | 0.549738 |
| 38 | hsa05010 Alzheimer's disease - Homo sapiens (human) | -19447.9 | 0.005 | 0.012089 | 0.319372 |
| 39 | hsa05214 Glioma - Homo sapiens (human) | -11119.1 | 0.005 | 0.012089 | 0.308901 |
| 40 | hsa05218 Melanoma - Homo sapiens (human) | -8006.6 | 0.005 | 0.012089 | 0.34555 |
| 41 | hsa05220 Chronic myeloid leukemia - Homo sapiens (human) | -14671.3 | 0.005 | 0.012089 | 0.497382 |
| 42 | hsa05332 Graft-versus-host disease - Homo sapiens (human) | -5554.1 | 0.005 | 0.012089 | 0.492147 |
| 43 | hsa04070 Phosphatidylinositol signaling system - Homo sapiens (human) | -7681.91 | 0.006 | 0.013326 | 0.303665 |
| 44 | hsa04130 SNARE interactions in vesicular transport - Homo sapiens (human) | -9876.13 | 0.006 | 0.013326 | 0.560209 |
| 45 | hsa04140 Regulation of autophagy - Homo sapiens (human) | -5090.6 | 0.006 | 0.013326 | 0.748691 |
| 46 | hsa05014 Amyotrophic lateral sclerosis (ALS) - Homo sapiens (human) | -13866.8 | 0.006 | 0.013326 | 0.790576 |
| 47 | hsa04080 Neuroactive ligand-receptor interaction - Homo sapiens (human) | -35726.9 | 0.007 | 0.015368 | 0.664921 |
| 48 | hsa00052 Galactose metabolism - Homo sapiens (human) | -4833.92 | 0.01 | 0.020538 | 0.308901 |
| 49 | hsa00230 Purine metabolism - Homo sapiens (human) | -15108.3 | 0.01 | 0.020538 | 0.282723 |
| 50 | hsa00340 Histidine metabolism - Homo sapiens (human) | -5916.39 | 0.01 | 0.020538 | 0.790576 |
| 51 | hsa00140 C21-Steroid hormone metabolism - Homo sapiens (human) | -2758.73 | 0.011 | 0.021885 | 0.246073 |
| 52 | hsa04150 mTOR signaling pathway - Homo sapiens (human) | -11005.2 | 0.011 | 0.021885 | 0.65445 |
| 53 | hsa05222 Small cell lung cancer - Homo sapiens (human) | -13217.3 | 0.011 | 0.021885 | 0.612565 |
| 54 | hsa04020 Calcium signaling pathway - Homo sapiens (human) | -15337.5 | 0.013 | 0.025081 | 0.560209 |
| 55 | hsa04370 VEGF signaling pathway - Homo sapiens (human) | -8965.3 | 0.013 | 0.025081 | 0.282723 |
| 56 | hsa04740 Olfactory transduction - Homo sapiens (human) | -4753.33 | 0.013 | 0.025081 | 0.162304 |
| 57 | hsa04950 Maturity onset diabetes of the young - Homo sapiens (human) | -6777.28 | 0.015 | 0.02865 | 0.308901 |
| 58 | hsa00860 Porphyrin and chlorophyll metabolism - Homo sapiens (human) | -5685.15 | 0.018 | 0.03404 | 0.157068 |
| 59 | hsa00563 Glycosylphosphatidylinositol(GPI)-anchor biosynthesis - Homo sapiens (human) | -9164.43 | 0.019 | 0.035578 | 0.497382 |
| 60 | hsa00760 Nicotinate and nicotinamide metabolism - Homo sapiens (human) | -4336.37 | 0.021 | 0.0382 | 0.376963 |
| 61 | hsa04520 Adherens junction - Homo sapiens (human) | -17426.5 | 0.021 | 0.0382 | 0.356021 |
| 62 | hsa05130 Pathogenic Escherichia coli infection - EHEC - Homo sapiens (human) | -9074.95 | 0.021 | 0.0382 | 0.717277 |
| 63 | hsa00785 Lipoic acid metabolism - Homo sapiens (human) | -440.332 | 0.022 | 0.039271 | 0.900524 |
| 64 | hsa04330 Notch signaling pathway - Homo sapiens (human) | -8826.26 | 0.022 | 0.039271 | 0.827225 |
| 65 | hsa05213 Endometrial cancer - Homo sapiens (human) | -11066.3 | 0.023 | 0.040676 | 0.481675 |
| 66 | hsa04930 Type II diabetes mellitus - Homo sapiens (human) | -7697.08 | 0.024 | 0.042055 | 0.643979 |
| 67 | hsa00350 Tyrosine metabolism - Homo sapiens (human) | -7399.31 | 0.028 | 0.048618 | 0.994764 |
| 68 | hsa00072 Synthesis and degradation of ketone bodies - Homo sapiens (human) | -2038.16 | 0.033 | 0.056277 | 0.612565 |
| 69 | hsa05219 Bladder cancer - Homo sapiens (human) | -10319.9 | 0.033 | 0.056277 | 0.769634 |
| 70 | hsa04012 ErbB signaling pathway - Homo sapiens (human) | -18459.6 | 0.036 | 0.06085 | 0.727749 |
| 71 | hsa00643 Styrene degradation - Homo sapiens (human) | -1177.34 | 0.041 | 0.068693 | 0.612565 |
| 72 | hsa05221 Acute myeloid leukemia - Homo sapiens (human) | -12631.5 | 0.044 | 0.073078 | 0.649215 |
| 73 | hsa00300 Lysine biosynthesis - Homo sapiens (human) | -1687.28 | 0.052 | 0.084169 | 0.73822 |
| 74 | hsa00400 Phenylalanine, tyrosine and tryptophan biosynthesis - Homo sapiens (human) | -1779.57 | 0.052 | 0.084169 | 0.455497 |
| 75 | hsa00900 Terpenoid biosynthesis - Homo sapiens (human) | -1706.11 | 0.052 | 0.084169 | 0.790576 |
| 76 | hsa00363 Bisphenol A degradation - Homo sapiens (human) | -869.199 | 0.053 | 0.085067 | 0.95288 |
| 77 | hsa00910 Nitrogen metabolism - Homo sapiens (human) | -4052.63 | 0.054 | 0.08595 | 0.643979 |
| 78 | hsa00950 Alkaloid biosynthesis I - Homo sapiens (human) | -1572.25 | 0.062 | 0.097868 | 0.659686 |
| 79 | hsa01040 Biosynthesis of unsaturated fatty acids - Homo sapiens (human) | -870.123 | 0.065 | 0.100935 | 0.979058 |
| 80 | hsa05131 Pathogenic Escherichia coli infection - EPEC - Homo sapiens (human) | -9104.31 | 0.065 | 0.100935 | 0.769634 |
| 81 | hsa02010 ABC transporters - General - Homo sapiens (human) | -18736.2 | 0.075 | 0.115524 | 1 |
| 82 | hsa04742 Taste transduction - Homo sapiens (human) | -7013.35 | 0.077 | 0.117656 | 0.801047 |
| 83 | hsa00564 Glycerophospholipid metabolism - Homo sapiens (human) | -11284.6 | 0.08 | 0.12127 | 0.968586 |
| 84 | hsa00980 Metabolism of xenobiotics by cytochrome P450 - Homo sapiens (human) | -2431.12 | 0.096 | 0.144378 | 0.439791 |
| 85 | hsa00680 Methane metabolism - Homo sapiens (human) | -2136.74 | 0.097 | 0.144742 | 0.827225 |
| 86 | hsa04614 Renin-angiotensin system - Homo sapiens (human) | -6972.47 | 0.109 | 0.161388 | 1 |
| 87 | hsa04730 Long-term depression - Homo sapiens (human) | -9338.34 | 0.111 | 0.163085 | 0.649215 |
| 88 | hsa00650 Butanoate metabolism - Homo sapiens (human) | -5666.32 | 0.113 | 0.164756 | 1 |
| 89 | hsa00361 gamma-Hexachlorocyclohexane degradation - Homo sapiens (human) | -2121.95 | 0.117 | 0.168023 | 0.795812 |
| 90 | hsa00770 Pantothenate and CoA biosynthesis - Homo sapiens (human) | -3944.97 | 0.117 | 0.168023 | 0.780105 |
| 91 | hsa00271 Methionine metabolism - Homo sapiens (human) | -5228.77 | 0.121 | 0.17247 | 0.790576 |
| 92 | hsa00532 Chondroitin sulfate biosynthesis - Homo sapiens (human) | -5012.2 | 0.126 | 0.178267 | 0.890052 |
| 93 | hsa00592 alpha-Linolenic acid metabolism - Homo sapiens (human) | -1252.18 | 0.136 | 0.191 | 0.858639 |
| 94 | hsa00471 D-Glutamine and D-glutamate metabolism - Homo sapiens (human) | -441.841 | 0.142 | 0.197971 | 1 |
| 95 | hsa03060 Protein export - Homo sapiens (human) | -3051.3 | 0.152 | 0.210377 | 1 |
| 96 | hsa03010 Ribosome - Homo sapiens (human) | -30958.3 | 0.173 | 0.237719 | 1 |
| 97 | hsa05216 Thyroid cancer - Homo sapiens (human) | -5648.2 | 0.194 | 0.264671 | 0.900524 |
| 98 | hsa03050 Proteasome - Homo sapiens (human) | -18314.6 | 0.202 | 0.273631 | 1 |
| 99 | hsa00440 Aminophosphonate metabolism - Homo sapiens (human) | -1682.73 | 0.206 | 0.277085 | 0.874346 |
| 100 | hsa00511 N-Glycan degradation - Homo sapiens (human) | -3488.04 | 0.212 | 0.283161 | 1 |
| 101 | hsa01032 Glycan structures - degradation - Homo sapiens (human) | -8285 | 0.216 | 0.2865 | 1 |
| 102 | hsa00790 Folate biosynthesis - Homo sapiens (human) | -1743.92 | 0.219 | 0.287808 | 1 |
| 103 | hsa04630 Jak-STAT signaling pathway - Homo sapiens (human) | -10504 | 0.22 | 0.287808 | 0.947644 |
| 104 | hsa04120 Ubiquitin mediated proteolysis - Homo sapiens (human) | -40993.2 | 0.241 | 0.313136 | 1 |
| 105 | hsa00190 Oxidative phosphorylation - Homo sapiens (human) | -37030.8 | 0.258 | 0.332959 | 1 |
| 106 | hsa00310 Lysine degradation - Homo sapiens (human) | -2180.32 | 0.268 | 0.343544 | 1 |
| 107 | hsa04810 Regulation of actin cytoskeleton - Homo sapiens (human) | -15702 | 0.34 | 0.432933 | 1 |
| 108 | hsa05310 Asthma - Homo sapiens (human) | -1249.4 | 0.349 | 0.44145 | 1 |
| 109 | hsa05050 Dentatorubropallidoluysian atrophy (DRPLA) - Homo sapiens (human) | -3489.27 | 0.371 | 0.466191 | 1 |
| 110 | hsa05060 Prion disease - Homo sapiens (human) | -4361.85 | 0.381 | 0.475627 | 1 |
| 111 | hsa03440 Homologous recombination - Homo sapiens (human) | -7851.02 | 0.399 | 0.489744 | 1 |
| 112 | hsa03030 DNA replication - Homo sapiens (human) | -13958.3 | 0.4 | 0.489744 | 1 |
| 113 | hsa03420 Nucleotide excision repair - Homo sapiens (human) | -11776.8 | 0.4 | 0.489744 | 1 |
| 114 | hsa00591 Linoleic acid metabolism - Homo sapiens (human) | -872.519 | 0.403 | 0.490274 | 1 |
| 115 | hsa00970 Aminoacyl-tRNA biosynthesis - Homo sapiens (human) | -10024.4 | 0.422 | 0.510139 | 1 |
| 116 | hsa05320 Autoimmune thyroid disease - Homo sapiens (human) | -1250.12 | 0.45 | 0.540566 | 1 |
| 117 | hsa00040 Pentose and glucuronate interconversions - Homo sapiens (human) | -3457.55 | 0.453 | 0.540769 | 1 |
| 118 | hsa00130 Ubiquinone and menaquinone biosynthesis - Homo sapiens (human) | -2187.43 | 0.458 | 0.543342 | 1 |
| 119 | hsa00460 Cyanoamino acid metabolism - Homo sapiens (human) | -1745.19 | 0.461 | 0.543525 | 1 |
| 120 | hsa00641 3-Chloroacrylic acid degradation - Homo sapiens (human) | -442.707 | 0.48 | 0.562454 | 1 |
| 121 | hsa00630 Glyoxylate and dicarboxylate metabolism - Homo sapiens (human) | -4090.8 | 0.53 | 0.617256 | 1 |
| 122 | hsa00031 Inositol metabolism - Homo sapiens (human) | -873.095 | 0.583 | 0.674867 | 1 |
| 123 | hsa04060 Cytokine-cytokine receptor interaction - Homo sapiens (human) | -44505.9 | 0.613 | 0.705319 | 1 |
| 124 | hsa01031 Glycan structures - biosynthesis 2 - Homo sapiens (human) | -13089.4 | 0.64 | 0.731976 | 1 |
| 125 | hsa00530 Aminosugars metabolism - Homo sapiens (human) | -3926.82 | 0.651 | 0.740125 | 1 |
| 126 | hsa00281 Geraniol degradation - Homo sapiens (human) | -873.172 | 0.674 | 0.76174 | 1 |
| 127 | hsa00534 Heparan sulfate biosynthesis - Homo sapiens (human) | -5962.28 | 0.687 | 0.768442 | 1 |
| 128 | hsa00780 Biotin metabolism - Homo sapiens (human) | -1309.43 | 0.688 | 0.768442 | 1 |
| 129 | hsa00150 Androgen and estrogen metabolism - Homo sapiens (human) | -873.172 | 0.692 | 0.768442 | 1 |
| 130 | hsa03410 Base excision repair - Homo sapiens (human) | -10471.7 | 0.71 | 0.783873 | 1 |
| 131 | hsa05340 Primary immunodeficiency - Homo sapiens (human) | -14836.1 | 0.72 | 0.789103 | 1 |
| 132 | hsa01030 Glycan structures - biosynthesis 1 - Homo sapiens (human) | -24435.2 | 0.723 | 0.789103 | 1 |
| 133 | hsa00533 Keratan sulfate biosynthesis - Homo sapiens (human) | -4364.28 | 0.764 | 0.827667 | 1 |
| 134 | hsa00450 Selenoamino acid metabolism - Homo sapiens (human) | -2618.92 | 0.767 | 0.827667 | 1 |
| 135 | hsa00120 Bile acid biosynthesis - Homo sapiens (human) | -1746.16 | 0.786 | 0.843404 | 1 |
| 136 | hsa00930 Caprolactam degradation - Homo sapiens (human) | -1299.29 | 0.793 | 0.846162 | 1 |
| 137 | hsa03450 Non-homologous end-joining - Homo sapiens (human) | -5673.19 | 0.804 | 0.846907 | 1 |
| 138 | hsa00730 Thiamine metabolism - Homo sapiens (human) | -1309.67 | 0.805 | 0.846907 | 1 |
| 139 | hsa03020 RNA polymerase - Homo sapiens (human) | -10472.3 | 0.807 | 0.846907 | 1 |
| 140 | hsa00550 Peptidoglycan biosynthesis - Homo sapiens (human) | -873.326 | 0.837 | 0.87359 | 1 |
| 141 | hsa00053 Ascorbate and aldarate metabolism - Homo sapiens (human) | -1309.87 | 0.936 | 0.971609 | 1 |
| 142 | hsa00380 Tryptophan metabolism - Homo sapiens (human) | -1746.41 | 0.951 | 0.981843 | 1 |
| 143 | hsa05120 Epithelial cell signaling in Helicobacter pylori infection - Homo sapiens (human) | -13277.4 | 0.975 | 1 | 1 |
| 144 | hsa05040 Huntington's disease - Homo sapiens (human) | -9167.4 | 0.993 | 1 | 1 |
| 145 | hsa00290 Valine, leucine and isoleucine biosynthesis - Homo sapiens (human) | -2133.22 | 0.994 | 1 | 1 |
| 146 | hsa03022 Basal transcription factors - Homo sapiens (human) | -10039.4 | 0.994 | 1 | 1 |
| 147 | hsa04640 Hematopoietic cell lineage - Homo sapiens (human) | -27940.5 | 0.998 | 1 | 1 |
| 148 | hsa00360 Phenylalanine metabolism - Homo sapiens (human) | -873.364 | 1 | 1 | 1 |
| 149 | hsa00364 Fluorobenzoate degradation - Homo sapiens (human) | -1 | -1 | -1 | -1 |
| 150 | hsa00472 D-Arginine and D-ornithine metabolism - Homo sapiens (human) | -1 | -1 | -1 | -1 |
| 151 | hsa00624 1- and 2-Methylnaphthalene degradation - Homo sapiens (human) | -1 | -1 | -1 | -1 |
| 152 | hsa00625 Tetrachloroethene degradation - Homo sapiens (human) | -1 | -1 | -1 | -1 |
| 153 | hsa00627 1,4-Dichlorobenzene degradation - Homo sapiens (human) | -1 | -1 | -1 | -1 |
| 154 | hsa00632 Benzoate degradation via CoA ligation - Homo sapiens (human) | -1 | -1 | -1 | -1 |
| 155 | hsa00740 Riboflavin metabolism - Homo sapiens (human) | -1 | -1 | -1 | -1 |
| 156 | hsa00791 Atrazine degradation - Homo sapiens (human) | -1 | -1 | -1 | -1 |
| 157 | hsa00902 Monoterpenoid biosynthesis - Homo sapiens (human) | -1 | -1 | -1 | -1 |
| 158 | hsa00903 Limonene and pinene degradation - Homo sapiens (human) | -1 | -1 | -1 | -1 |
| 159 | hsa01430 Cell Communication - Homo sapiens (human) | -1 | -1 | -1 | -1 |
| 160 | hsa04340 Hedgehog signaling pathway - Homo sapiens (human) | -1 | -1 | -1 | -1 |
| 161 | hsa04940 Type I diabetes mellitus - Homo sapiens (human) | -1 | -1 | -1 | -1 |
| 162 | hsa05016 - Homo sapiens (human) | -1 | -1 | -1 | -1 |
| 163 | hsa05322 Systemic lupus erythematosus - Homo sapiens (human) | -1 | -1 | -1 | -1 |

Result obtained by the GSEA methods in the lung cancer dataset

| No | geneset | size | ES | NES | NOM p-value | FDR q-value | FWER p-value | Rank at max |
| --- | --- | --- | --- | --- | --- | --- | --- | --- |
| 1 | KEGG_LONG_TERM_POTENTIATION | 68 | -0.535 | -2.035 | 0 | 0.006 | 0.009 | 2,840 |
| 2 | KEGG_VASCULAR_SMOOTH_MUSCLE_CONTRACTION | 108 | -0.637 | -2.085 | 0 | 0.008 | 0.005 | 2,840 |
| 3 | KEGG_DILATED_CARDIOMYOPATHY | 90 | -0.575 | -1.957 | 0 | 0.011 | 0.036 | 3,373 |
| 4 | KEGG_CALCIUM_SIGNALING_PATHWAY | 173 | -0.485 | -1.968 | 0 | 0.013 | 0.032 | 2,653 |
| 5 | KEGG_GAP_JUNCTION | 86 | -0.532 | -1.897 | 0 | 0.015 | 0.072 | 2,258 |
| 6 | KEGG_NEUROACTIVE_LIGAND_RECEPTOR_INTERACTION | 263 | -0.436 | -1.873 | 0 | 0.018 | 0.095 | 1,707 |
| 7 | KEGG_HYPERTROPHIC_CARDIOMYOPATHY_HCM | 83 | -0.518 | -1.898 | 0 | 0.018 | 0.071 | 3,373 |
| 8 | KEGG_TIGHT_JUNCTION | 130 | -0.451 | -1.838 | 0 | 0.024 | 0.135 | 1,010 |
| 9 | KEGG_PRION_DISEASES | 34 | -0.6 | -1.78 | 0.004 | 0.025 | 0.218 | 2,801 |
| 10 | KEGG_DORSO_VENTRAL_AXIS_FORMATION | 23 | -0.566 | -1.77 | 0.004 | 0.026 | 0.245 | 2,072 |
| 11 | KEGG_LONG_TERM_DEPRESSION | 65 | -0.503 | -1.78 | 0 | 0.026 | 0.217 | 2,162 |
| 12 | KEGG_AXON_GUIDANCE | 127 | -0.454 | -1.753 | 0.002 | 0.028 | 0.282 | 2,583 |
| 13 | KEGG_LEUKOCYTE_TRANSENDOTHELIAL_MIGRATION | 113 | -0.516 | -1.781 | 0.006 | 0.028 | 0.217 | 3,417 |
| 14 | KEGG_NATURAL_KILLER_CELL_MEDIATED_CYTOTOXICITY | 126 | -0.541 | -1.796 | 0.014 | 0.028 | 0.191 | 3,783 |
| 15 | KEGG_GNRH_SIGNALING_PATHWAY | 94 | -0.444 | -1.757 | 0 | 0.028 | 0.272 | 2,840 |
| 16 | KEGG_TGF_BETA_SIGNALING_PATHWAY | 82 | -0.525 | -1.785 | 0 | 0.029 | 0.209 | 2,709 |
| 17 | KEGG_VIRAL_MYOCARDITIS | 66 | -0.547 | -1.809 | 0.006 | 0.031 | 0.174 | 4,301 |
| 18 | KEGG_PPAR_SIGNALING_PATHWAY | 67 | -0.495 | -1.797 | 0.002 | 0.031 | 0.191 | 1,469 |
| 19 | KEGG_MELANOGENESIS | 99 | -0.434 | -1.736 | 0 | 0.032 | 0.32 | 2,128 |
| 20 | KEGG_REGULATION_OF_ACTIN_CYTOSKELETON | 211 | -0.416 | -1.698 | 0.004 | 0.046 | 0.414 | 3,161 |
| 21 | KEGG_MAPK_SIGNALING_PATHWAY | 257 | -0.416 | -1.683 | 0.008 | 0.05 | 0.445 | 3,165 |
| 22 | KEGG_ALDOSTERONE_REGULATED_SODIUM_REABSORPTION | 42 | -0.521 | -1.671 | 0.01 | 0.054 | 0.467 | 2,903 |
| 23 | KEGG_PHOSPHATIDYLINOSITOL_SIGNALING_SYSTEM | 75 | -0.427 | -1.664 | 0.002 | 0.054 | 0.479 | 3,963 |
| 24 | KEGG_ENDOCYTOSIS | 173 | -0.412 | -1.637 | 0.004 | 0.057 | 0.556 | 2,635 |
| 25 | KEGG_ARRHYTHMOGENIC_RIGHT_VENTRICULAR_CARDIOMYOPATHY_ARVC | 74 | -0.472 | -1.639 | 0.014 | 0.058 | 0.55 | 3,373 |
| 26 | KEGG_CELL_ADHESION_MOLECULES_CAMS | 128 | -0.454 | -1.645 | 0.016 | 0.059 | 0.527 | 3,225 |
| 27 | KEGG_TYPE_II_DIABETES_MELLITUS | 45 | -0.491 | -1.639 | 0.022 | 0.06 | 0.547 | 3,919 |
| 28 | KEGG_CHEMOKINE_SIGNALING_PATHWAY | 178 | -0.482 | -1.647 | 0.008 | 0.06 | 0.525 | 3,417 |
| 29 | KEGG_COMPLEMENT_AND_COAGULATION_CASCADES | 67 | -0.499 | -1.65 | 0.028 | 0.06 | 0.517 | 2,224 |
| 30 | KEGG_O_GLYCAN_BIOSYNTHESIS | 26 | 0.682 | 1.835 | 0.002 | 0.068 | 0.132 | 2,345 |
| 31 | KEGG_FC_GAMMA_R_MEDIATED_PHAGOCYTOSIS | 92 | -0.465 | -1.612 | 0.018 | 0.068 | 0.609 | 3,552 |
| 32 | KEGG_FOCAL_ADHESION | 197 | -0.436 | -1.596 | 0.024 | 0.071 | 0.636 | 2,962 |
| 33 | KEGG_WNT_SIGNALING_PATHWAY | 147 | -0.384 | -1.597 | 0.004 | 0.073 | 0.633 | 2,932 |
| 34 | KEGG_HEMATOPOIETIC_CELL_LINEAGE | 83 | -0.529 | -1.572 | 0.066 | 0.084 | 0.676 | 2,945 |
| 35 | KEGG_PYRIMIDINE_METABOLISM | 89 | 0.544 | 1.752 | 0.002 | 0.087 | 0.265 | 2,910 |
| 36 | KEGG_RENIN_ANGIOTENSIN_SYSTEM | 17 | -0.596 | -1.559 | 0.031 | 0.087 | 0.692 | 2,275 |
| 37 | KEGG_GRAFT_VERSUS_HOST_DISEASE | 34 | -0.674 | -1.56 | 0.068 | 0.089 | 0.692 | 4,242 |
| 38 | KEGG_ADHERENS_JUNCTION | 72 | -0.451 | -1.547 | 0.052 | 0.092 | 0.712 | 3,067 |
| 39 | KEGG_JAK_STAT_SIGNALING_PATHWAY | 150 | -0.4 | -1.543 | 0.04 | 0.092 | 0.718 | 2,902 |
| 40 | KEGG_PATHWAYS_IN_CANCER | 321 | -0.361 | -1.524 | 0.016 | 0.098 | 0.745 | 2,939 |
| 41 | KEGG_GLYCOSPHINGOLIPID_BIOSYNTHESIS_GANGLIO_SERIES | 15 | -0.535 | -1.525 | 0.04 | 0.099 | 0.744 | 347 |
| 42 | KEGG_CARDIAC_MUSCLE_CONTRACTION | 73 | -0.332 | -1.527 | 0.02 | 0.101 | 0.743 | 2,478 |
| 43 | KEGG_ADIPOCYTOKINE_SIGNALING_PATHWAY | 66 | -0.403 | -1.513 | 0.036 | 0.102 | 0.761 | 2,145 |
| 44 | KEGG_GLYOXYLATE_AND_DICARBOXYLATE_METABOLISM | 16 | 0.605 | 1.759 | 0.008 | 0.109 | 0.251 | 2,516 |
| 45 | KEGG_HEDGEHOG_SIGNALING_PATHWAY | 54 | -0.377 | -1.498 | 0.026 | 0.111 | 0.791 | 2,049 |
| 46 | KEGG_N_GLYCAN_BIOSYNTHESIS | 45 | 0.625 | 1.844 | 0.008 | 0.123 | 0.119 | 4,004 |
| 47 | KEGG_PROTEIN_EXPORT | 23 | 0.671 | 1.697 | 0.024 | 0.127 | 0.387 | 4,565 |
| 48 | KEGG_CYTOKINE_CYTOKINE_RECEPTOR_INTERACTION | 247 | -0.403 | -1.472 | 0.058 | 0.128 | 0.829 | 2,760 |
| 49 | KEGG_AUTOIMMUNE_THYROID_DISEASE | 47 | -0.468 | -1.463 | 0.098 | 0.132 | 0.837 | 5,216 |
| 50 | KEGG_RENAL_CELL_CARCINOMA | 69 | -0.399 | -1.459 | 0.04 | 0.132 | 0.843 | 2,868 |
| 51 | KEGG_LEISHMANIA_INFECTION | 66 | -0.484 | -1.449 | 0.108 | 0.139 | 0.861 | 3,745 |
| 52 | KEGG_BASE_EXCISION_REPAIR | 32 | 0.646 | 1.569 | 0.057 | 0.139 | 0.667 | 5,142 |
| 53 | KEGG_FC_EPSILON_RI_SIGNALING_PATHWAY | 73 | -0.398 | -1.445 | 0.07 | 0.14 | 0.868 | 3,686 |
| 54 | KEGG_ALANINE_ASPARTATE_AND_GLUTAMATE_METABOLISM | 32 | 0.539 | 1.652 | 0.01 | 0.141 | 0.486 | 3,962 |
| 55 | KEGG_VEGF_SIGNALING_PATHWAY | 70 | -0.382 | -1.439 | 0.044 | 0.142 | 0.873 | 3,426 |
| 56 | KEGG_ALLOGRAFT_REJECTION | 33 | -0.58 | -1.43 | 0.145 | 0.146 | 0.881 | 5,216 |
| 57 | KEGG_HOMOLOGOUS_RECOMBINATION | 28 | 0.687 | 1.571 | 0.037 | 0.147 | 0.666 | 2,089 |
| 58 | KEGG_PROTEASOME | 42 | 0.637 | 1.63 | 0.037 | 0.148 | 0.533 | 4,885 |
| 59 | KEGG_CELL_CYCLE | 122 | 0.543 | 1.662 | 0.042 | 0.15 | 0.457 | 3,205 |
| 60 | KEGG_PYRUVATE_METABOLISM | 39 | 0.488 | 1.518 | 0.056 | 0.15 | 0.769 | 6,257 |
| 61 | KEGG_RNA_POLYMERASE | 27 | 0.508 | 1.508 | 0.045 | 0.152 | 0.785 | 2,910 |
| 62 | KEGG_DNA_REPLICATION | 36 | 0.678 | 1.493 | 0.11 | 0.153 | 0.816 | 4,369 |
| 63 | KEGG_AMINO_SUGAR_AND_NUCLEOTIDE_SUGAR_METABOLISM | 42 | 0.481 | 1.485 | 0.048 | 0.155 | 0.832 | 3,449 |
| 64 | KEGG_CITRATE_CYCLE_TCA_CYCLE | 30 | 0.553 | 1.519 | 0.065 | 0.156 | 0.764 | 4,966 |
| 65 | KEGG_OXIDATIVE_PHOSPHORYLATION | 116 | 0.448 | 1.496 | 0.066 | 0.158 | 0.813 | 6,437 |
| 66 | KEGG_P53_SIGNALING_PATHWAY | 66 | 0.46 | 1.572 | 0.036 | 0.159 | 0.665 | 2,591 |
| 67 | KEGG_BUTANOATE_METABOLISM | 33 | 0.515 | 1.53 | 0.038 | 0.162 | 0.747 | 4,514 |
| 68 | KEGG_COLORECTAL_CANCER | 62 | -0.397 | -1.405 | 0.059 | 0.163 | 0.908 | 2,868 |
| 69 | KEGG_GLYCOSAMINOGLYCAN_BIOSYNTHESIS_HEPARAN_SULFATE | 26 | 0.495 | 1.521 | 0.051 | 0.164 | 0.76 | 1,619 |
| 70 | KEGG_LYSINE_DEGRADATION | 44 | 0.509 | 1.577 | 0.025 | 0.164 | 0.649 | 4,910 |
| 71 | KEGG_ONE_CARBON_POOL_BY_FOLATE | 16 | 0.61 | 1.586 | 0.029 | 0.167 | 0.625 | 3,090 |
| 72 | KEGG_PURINE_METABOLISM | 147 | 0.353 | 1.466 | 0.035 | 0.169 | 0.857 | 3,090 |
| 73 | KEGG_PORPHYRIN_AND_CHLOROPHYLL_METABOLISM | 29 | 0.491 | 1.459 | 0.06 | 0.17 | 0.864 | 2,935 |
| 74 | KEGG_FRUCTOSE_AND_MANNOSE_METABOLISM | 33 | 0.486 | 1.531 | 0.055 | 0.173 | 0.747 | 2,821 |
| 75 | KEGG_AMINOACYL_TRNA_BIOSYNTHESIS | 41 | 0.563 | 1.594 | 0.027 | 0.174 | 0.607 | 3,779 |
| 76 | KEGG_INSULIN_SIGNALING_PATHWAY | 135 | -0.336 | -1.385 | 0.057 | 0.175 | 0.922 | 2,868 |
| 77 | KEGG_GLIOMA | 64 | -0.351 | -1.386 | 0.046 | 0.177 | 0.921 | 2,868 |
| 78 | KEGG_NUCLEOTIDE_EXCISION_REPAIR | 44 | 0.518 | 1.446 | 0.109 | 0.178 | 0.882 | 4,384 |
| 79 | KEGG_MISMATCH_REPAIR | 22 | 0.654 | 1.43 | 0.12 | 0.189 | 0.903 | 2,509 |
| 80 | KEGG_CHRONIC_MYELOID_LEUKEMIA | 72 | -0.395 | -1.355 | 0.138 | 0.193 | 0.941 | 2,868 |
| 81 | KEGG_DRUG_METABOLISM_CYTOCHROME_P450 | 59 | -0.394 | -1.35 | 0.074 | 0.195 | 0.943 | 2,813 |
| 82 | KEGG_MELANOMA | 71 | -0.333 | -1.359 | 0.055 | 0.196 | 0.939 | 2,939 |
| 83 | KEGG_PANCREATIC_CANCER | 69 | -0.387 | -1.355 | 0.107 | 0.197 | 0.941 | 2,868 |
| 84 | KEGG_PROXIMAL_TUBULE_BICARBONATE_RECLAMATION | 23 | -0.432 | -1.337 | 0.128 | 0.198 | 0.952 | 1,235 |
| 85 | KEGG_T_CELL_RECEPTOR_SIGNALING_PATHWAY | 107 | -0.387 | -1.338 | 0.156 | 0.2 | 0.951 | 3,783 |
| 86 | KEGG_NOD_LIKE_RECEPTOR_SIGNALING_PATHWAY | 61 | -0.43 | -1.34 | 0.138 | 0.202 | 0.951 | 3,460 |
| 87 | KEGG_ARGININE_AND_PROLINE_METABOLISM | 50 | 0.412 | 1.399 | 0.064 | 0.203 | 0.927 | 3,962 |
| 88 | KEGG_PARKINSONS_DISEASE | 112 | 0.417 | 1.4 | 0.115 | 0.21 | 0.927 | 5,158 |
| 89 | KEGG_ABC_TRANSPORTERS | 43 | -0.34 | -1.32 | 0.098 | 0.213 | 0.959 | 1,043 |
| 90 | KEGG_HUNTINGTONS_DISEASE | 166 | 0.384 | 1.4 | 0.111 | 0.217 | 0.926 | 3,617 |
| 91 | KEGG_B_CELL_RECEPTOR_SIGNALING_PATHWAY | 73 | -0.392 | -1.296 | 0.134 | 0.219 | 0.967 | 3,125 |
| 92 | KEGG_GLUTATHIONE_METABOLISM | 47 | 0.439 | 1.378 | 0.124 | 0.221 | 0.943 | 2,866 |
| 93 | KEGG_TYPE_I_DIABETES_MELLITUS | 39 | -0.468 | -1.296 | 0.204 | 0.223 | 0.967 | 4,242 |
| 94 | KEGG_NON_SMALL_CELL_LUNG_CANCER | 54 | -0.361 | -1.306 | 0.117 | 0.223 | 0.963 | 3,417 |
| 95 | KEGG_SMALL_CELL_LUNG_CANCER | 84 | -0.363 | -1.302 | 0.136 | 0.224 | 0.966 | 3,462 |
| 96 | KEGG_AMYOTROPHIC_LATERAL_SCLEROSIS_ALS | 51 | -0.336 | -1.297 | 0.1 | 0.226 | 0.967 | 2,315 |
| 97 | KEGG_BASAL_CELL_CARCINOMA | 54 | -0.342 | -1.274 | 0.121 | 0.232 | 0.975 | 1,887 |
| 98 | KEGG_NITROGEN_METABOLISM | 22 | -0.423 | -1.28 | 0.178 | 0.233 | 0.975 | 2,077 |
| 99 | KEGG_APOPTOSIS | 86 | -0.371 | -1.276 | 0.175 | 0.233 | 0.975 | 3,462 |
| 100 | KEGG_SPLICEOSOME | 113 | 0.417 | 1.36 | 0.142 | 0.235 | 0.954 | 5,756 |
| 101 | KEGG_DRUG_METABOLISM_OTHER_ENZYMES | 38 | 0.414 | 1.348 | 0.091 | 0.236 | 0.959 | 2,604 |
| 102 | KEGG_TYROSINE_METABOLISM | 42 | -0.34 | -1.261 | 0.096 | 0.241 | 0.98 | 3,198 |
| 103 | KEGG_PEROXISOME | 77 | 0.387 | 1.348 | 0.133 | 0.243 | 0.959 | 5,001 |
| 104 | KEGG_RNA_DEGRADATION | 57 | 0.403 | 1.337 | 0.114 | 0.244 | 0.966 | 4,556 |
| 105 | KEGG_ASTHMA | 26 | -0.472 | -1.254 | 0.241 | 0.246 | 0.982 | 5,697 |
| 106 | KEGG_NEUROTROPHIN_SIGNALING_PATHWAY | 125 | -0.324 | -1.233 | 0.208 | 0.262 | 0.988 | 3,460 |
| 107 | KEGG_GLYCOSYLPHOSPHATIDYLINOSITOL_GPI_ANCHOR_BIOSYNTHESIS | 23 | 0.48 | 1.305 | 0.169 | 0.262 | 0.974 | 5,256 |
| 108 | KEGG_PENTOSE_PHOSPHATE_PATHWAY | 26 | 0.432 | 1.31 | 0.172 | 0.263 | 0.973 | 4,190 |
| 109 | KEGG_INOSITOL_PHOSPHATE_METABOLISM | 54 | -0.33 | -1.234 | 0.132 | 0.263 | 0.988 | 3,963 |
| 110 | KEGG_ERBB_SIGNALING_PATHWAY | 87 | -0.317 | -1.227 | 0.186 | 0.264 | 0.988 | 2,868 |
| 111 | KEGG_VALINE_LEUCINE_AND_ISOLEUCINE_DEGRADATION | 44 | 0.438 | 1.314 | 0.172 | 0.264 | 0.972 | 4,398 |
| 112 | KEGG_TASTE_TRANSDUCTION | 44 | -0.316 | -1.203 | 0.24 | 0.288 | 0.991 | 2,903 |
| 113 | KEGG_RIBOFLAVIN_METABOLISM | 16 | 0.426 | 1.278 | 0.206 | 0.289 | 0.978 | 3,238 |
| 114 | KEGG_PHENYLALANINE_METABOLISM | 18 | -0.38 | -1.191 | 0.22 | 0.298 | 0.991 | 1,461 |
| 115 | KEGG_GLYCOSPHINGOLIPID_BIOSYNTHESIS_LACTO_AND_NEOLACTO_SERIES | 26 | 0.383 | 1.247 | 0.164 | 0.308 | 0.985 | 1,110 |
| 116 | KEGG_VIBRIO_CHOLERAE_INFECTION | 53 | 0.347 | 1.251 | 0.159 | 0.311 | 0.984 | 2,872 |
| 117 | KEGG_CYSTEINE_AND_METHIONINE_METABOLISM | 34 | 0.403 | 1.254 | 0.193 | 0.313 | 0.983 | 2,530 |
| 118 | KEGG_ANTIGEN_PROCESSING_AND_PRESENTATION | 73 | -0.345 | -1.164 | 0.301 | 0.323 | 0.993 | 4,427 |
| 119 | KEGG_ACUTE_MYELOID_LEUKEMIA | 56 | -0.349 | -1.165 | 0.269 | 0.326 | 0.993 | 3,125 |
| 120 | KEGG_GLYCOSAMINOGLYCAN_BIOSYNTHESIS_CHONDROITIN_SULFATE | 22 | 0.424 | 1.204 | 0.228 | 0.337 | 0.99 | 1,619 |
| 121 | KEGG_TERPENOID_BACKBONE_BIOSYNTHESIS | 15 | 0.463 | 1.209 | 0.248 | 0.338 | 0.99 | 5,333 |
| 122 | KEGG_VASOPRESSIN_REGULATED_WATER_REABSORPTION | 44 | -0.359 | -1.148 | 0.289 | 0.338 | 0.995 | 3,788 |
| 123 | KEGG_SELENOAMINO_ACID_METABOLISM | 26 | 0.388 | 1.219 | 0.193 | 0.339 | 0.989 | 2,601 |
| 124 | KEGG_BASAL_TRANSCRIPTION_FACTORS | 33 | 0.377 | 1.21 | 0.242 | 0.344 | 0.99 | 3,425 |
| 125 | KEGG_THYROID_CANCER | 29 | -0.381 | -1.131 | 0.309 | 0.352 | 0.998 | 2,072 |
| 126 | KEGG_MTOR_SIGNALING_PATHWAY | 50 | -0.309 | -1.126 | 0.276 | 0.355 | 0.998 | 2,909 |
| 127 | KEGG_BLADDER_CANCER | 40 | -0.336 | -1.132 | 0.251 | 0.355 | 0.998 | 1,468 |
| 128 | KEGG_GLYCOSAMINOGLYCAN_BIOSYNTHESIS_KERATAN_SULFATE | 15 | 0.411 | 1.182 | 0.219 | 0.358 | 0.99 | 3,613 |
| 129 | KEGG_PANTOTHENATE_AND_COA_BIOSYNTHESIS | 16 | 0.427 | 1.155 | 0.274 | 0.359 | 0.995 | 6,643 |
| 130 | KEGG_OTHER_GLYCAN_DEGRADATION | 15 | 0.443 | 1.176 | 0.29 | 0.36 | 0.994 | 3,362 |
| 131 | KEGG_STEROID_BIOSYNTHESIS | 15 | 0.462 | 1.158 | 0.279 | 0.362 | 0.995 | 2,889 |
| 132 | KEGG_PROGESTERONE_MEDIATED_OOCYTE_MATURATION | 83 | 0.3 | 1.162 | 0.225 | 0.363 | 0.995 | 1,561 |
| 133 | KEGG_RIG_I_LIKE_RECEPTOR_SIGNALING_PATHWAY | 69 | 0.297 | 1.164 | 0.269 | 0.368 | 0.995 | 2,758 |
| 134 | KEGG_PROSTATE_CANCER | 89 | -0.289 | -1.105 | 0.283 | 0.372 | 0.998 | 2,868 |
| 135 | KEGG_ENDOMETRIAL_CANCER | 52 | -0.313 | -1.106 | 0.31 | 0.376 | 0.998 | 3,417 |
| 136 | KEGG_FATTY_ACID_METABOLISM | 41 | -0.32 | -1.089 | 0.339 | 0.379 | 0.998 | 3,087 |
| 137 | KEGG_NOTCH_SIGNALING_PATHWAY | 47 | -0.298 | -1.09 | 0.3 | 0.383 | 0.998 | 2,094 |
| 138 | KEGG_GLYCOSAMINOGLYCAN_DEGRADATION | 21 | -0.349 | -1.091 | 0.308 | 0.385 | 0.998 | 311 |
| 139 | KEGG_MATURITY_ONSET_DIABETES_OF_THE_YOUNG | 23 | 0.353 | 1.13 | 0.316 | 0.386 | 0.997 | 8,585 |
| 140 | KEGG_SNARE_INTERACTIONS_IN_VESICULAR_TRANSPORT | 37 | -0.317 | -1.073 | 0.345 | 0.397 | 0.998 | 2,510 |
| 141 | KEGG_EPITHELIAL_CELL_SIGNALING_IN_HELICOBACTER_PYLORI_INFECTION | 66 | -0.297 | -1.064 | 0.373 | 0.404 | 0.998 | 2,518 |
| 142 | KEGG_ALZHEIMERS_DISEASE | 154 | 0.274 | 1.082 | 0.343 | 0.404 | 0.999 | 3,683 |
| 143 | KEGG_GLYCEROPHOSPHOLIPID_METABOLISM | 71 | 0.289 | 1.069 | 0.332 | 0.408 | 1 | 3,540 |
| 144 | KEGG_PROPANOATE_METABOLISM | 32 | 0.363 | 1.109 | 0.331 | 0.409 | 0.999 | 4,398 |
| 145 | KEGG_OOCYTE_MEIOSIS | 108 | 0.269 | 1.083 | 0.318 | 0.409 | 0.999 | 1,016 |
| 146 | KEGG_GLYCOLYSIS_GLUCONEOGENESIS | 60 | 0.317 | 1.093 | 0.333 | 0.409 | 0.999 | 6,876 |
| 147 | KEGG_PRIMARY_BILE_ACID_BIOSYNTHESIS | 16 | 0.373 | 1.085 | 0.343 | 0.412 | 0.999 | 3,433 |
| 148 | KEGG_SPHINGOLIPID_METABOLISM | 35 | 0.348 | 1.07 | 0.386 | 0.413 | 1 | 2,860 |
| 149 | KEGG_GALACTOSE_METABOLISM | 26 | 0.348 | 1.094 | 0.333 | 0.415 | 0.999 | 2,821 |
| 150 | KEGG_ARACHIDONIC_ACID_METABOLISM | 52 | -0.304 | -1.052 | 0.386 | 0.417 | 0.998 | 2,677 |
| 151 | KEGG_PENTOSE_AND_GLUCURONATE_INTERCONVERSIONS | 16 | 0.39 | 1.097 | 0.315 | 0.419 | 0.999 | 3,064 |
| 152 | KEGG_ECM_RECEPTOR_INTERACTION | 82 | -0.311 | -1.03 | 0.41 | 0.442 | 0.999 | 3,382 |
| 153 | KEGG_REGULATION_OF_AUTOPHAGY | 33 | -0.279 | -0.988 | 0.464 | 0.495 | 1 | 1,537 |
| 154 | KEGG_UBIQUITIN_MEDIATED_PROTEOLYSIS | 131 | 0.256 | 0.995 | 0.444 | 0.5 | 1 | 5,375 |
| 155 | KEGG_TOLL_LIKE_RECEPTOR_SIGNALING_PATHWAY | 97 | -0.273 | -0.98 | 0.48 | 0.502 | 1 | 2,145 |
| 156 | KEGG_RIBOSOME | 73 | 0.29 | 0.998 | 0.452 | 0.503 | 1 | 8,110 |
| 157 | KEGG_CYTOSOLIC_DNA_SENSING_PATHWAY | 53 | -0.27 | -0.963 | 0.508 | 0.523 | 1 | 2,451 |
| 158 | KEGG_GLYCINE_SERINE_AND_THREONINE_METABOLISM | 31 | -0.299 | -0.955 | 0.507 | 0.528 | 1 | 2,077 |
| 159 | KEGG_PATHOGENIC_ESCHERICHIA_COLI_INFECTION | 52 | -0.308 | -0.94 | 0.501 | 0.545 | 1 | 2,426 |
| 160 | KEGG_METABOLISM_OF_XENOBIOTICS_BY_CYTOCHROME_P450 | 57 | -0.281 | -0.929 | 0.566 | 0.557 | 1 | 2,813 |
| 161 | KEGG_LYSOSOME | 115 | 0.293 | 0.932 | 0.537 | 0.591 | 1 | 6,518 |
| 162 | KEGG_HISTIDINE_METABOLISM | 28 | -0.282 | -0.897 | 0.576 | 0.6 | 1 | 2,291 |
| 163 | KEGG_TRYPTOPHAN_METABOLISM | 39 | 0.258 | 0.903 | 0.603 | 0.63 | 1 | 4,155 |
| 164 | KEGG_BETA_ALANINE_METABOLISM | 22 | -0.284 | -0.868 | 0.661 | 0.64 | 1 | 2,291 |
| 165 | KEGG_GLYCEROLIPID_METABOLISM | 48 | 0.235 | 0.87 | 0.702 | 0.664 | 1 | 6,451 |
| 166 | KEGG_NICOTINATE_AND_NICOTINAMIDE_METABOLISM | 21 | 0.278 | 0.871 | 0.679 | 0.674 | 1 | 4,130 |
| 167 | KEGG_BIOSYNTHESIS_OF_UNSATURATED_FATTY_ACIDS | 19 | 0.281 | 0.838 | 0.714 | 0.696 | 1 | 4,377 |
| 168 | KEGG_STARCH_AND_SUCROSE_METABOLISM | 36 | 0.245 | 0.827 | 0.73 | 0.705 | 1 | 5,292 |
| 169 | KEGG_STEROID_HORMONE_BIOSYNTHESIS | 44 | 0.241 | 0.839 | 0.747 | 0.705 | 1 | 4,746 |
| 170 | KEGG_SYSTEMIC_LUPUS_ERYTHEMATOSUS | 81 | 0.227 | 0.815 | 0.711 | 0.713 | 1 | 4,936 |
| 171 | KEGG_ETHER_LIPID_METABOLISM | 28 | 0.254 | 0.794 | 0.755 | 0.735 | 1 | 1,022 |
| 172 | KEGG_INTESTINAL_IMMUNE_NETWORK_FOR_IGA_PRODUCTION | 44 | -0.282 | -0.779 | 0.69 | 0.772 | 1 | 5,216 |
