# Supplementary material for: Signalling pathway impact analysis based on the strength of interaction between genes
Source: IET Syst Biol. 2016 Aug 1;10(4):147–52. doi: 10.1049/iet-syb.2015.0089 (PMC8687233; doi:10.1049/iet-syb.2015.0089)
Supplement: Supplementary file 3 — Supplementary Data [file SYB2-10-147-s001.docx]

Result obtained by the PSPIA methods in the pancreatic cancer dataset

| No | Name | ID | pSize | NDE | pNDE | tA | pPERT | pG | pGFdr | pGFWER |
| --- | --- | --- | --- | --- | --- | --- | --- | --- | --- | --- |
| 1 | Focal adhesion | 4510 | 199 | 140 | 4.23E-07 | 10.70376 | 5.00E-06 | 5.89E-11 | 8.07E-09 | 8.07E-09 |
| 2 | ECM-receptor interaction | 4512 | 83 | 64 | 5.01E-06 | 8.146983 | 5.00E-06 | 6.36E-10 | 4.36E-08 | 8.71E-08 |
| 3 | Pathways in cancer | 5200 | 321 | 221 | 4.65E-09 | 8.671855 | 0.014 | 1.59E-09 | 7.27E-08 | 2.18E-07 |
| 4 | Small cell lung cancer | 5222 | 83 | 61 | 0.000105 | 7.511786 | 5.00E-06 | 1.18E-08 | 4.03E-07 | 1.61E-06 |
| 5 | Bacterial invasion of epithelial cells | 5100 | 70 | 51 | 0.000541 | 6.91377 | 5.00E-06 | 5.61E-08 | 1.54E-06 | 7.68E-06 |
| 6 | Gastric acid secretion | 4971 | 73 | 46 | 0.055053 | -2.65293 | 5.00E-06 | 4.43E-06 | 8.80E-05 | 0.000607 |
| 7 | Salmonella infection | 5132 | 80 | 50 | 0.055856 | 6.115971 | 5.00E-06 | 4.49E-06 | 8.80E-05 | 0.000616 |
| 8 | Regulation of actin cytoskeleton | 4810 | 212 | 144 | 6.86E-06 | 4.118324 | 0.05 | 5.45E-06 | 9.33E-05 | 0.000746 |
| 9 | Arrhythmogenic right ventricular cardiomyopathy (ARVC) | 5412 | 74 | 57 | 1.74E-05 | 0.359507 | 0.042 | 1.10E-05 | 0.000168 | 0.001513 |
| 10 | Cell cycle | 4110 | 122 | 84 | 0.000267 | -3.06781 | 0.034 | 0.000114 | 0.001568 | 0.015675 |
| 11 | Endocrine and other factor-regulated calcium reabsorption | 4961 | 49 | 38 | 0.000348 | -1.80975 | 0.043 | 0.000181 | 0.002259 | 0.024845 |
| 12 | Pathogenic Escherichia coli infection | 5130 | 51 | 35 | 0.017289 | 6.135667 | 0.002 | 0.00039 | 0.00445 | 0.053398 |
| 13 | Pancreatic secretion | 4972 | 91 | 63 | 0.001203 | -0.6682 | 0.036 | 0.000478 | 0.005041 | 0.065528 |
| 14 | Mineral absorption | 4978 | 49 | 37 | 0.00103 | 0.202069 | 0.112 | 0.001161 | 0.011363 | 0.159087 |
| 15 | Wnt signaling pathway | 4310 | 149 | 99 | 0.000601 | 1.104267 | 0.216 | 0.001293 | 0.011805 | 0.177075 |
| 16 | Calcium signaling pathway | 4020 | 180 | 115 | 0.002039 | -1.98777 | 0.082 | 0.001621 | 0.013882 | 0.222109 |
| 17 | Tuberculosis | 5152 | 172 | 98 | 0.168746 | 7.865083 | 0.002 | 0.003035 | 0.024462 | 0.415849 |
| 18 | Amyotrophic lateral sclerosis (ALS) | 5014 | 52 | 39 | 0.000951 | 1.015802 | 0.401 | 0.003382 | 0.02574 | 0.463316 |
| 19 | Salivary secretion | 4970 | 82 | 53 | 0.022315 | -1.59066 | 0.021 | 0.004061 | 0.029281 | 0.55634 |
| 20 | Notch signaling pathway | 4330 | 47 | 33 | 0.012474 | -2.71589 | 0.042 | 0.004482 | 0.030699 | 0.613988 |
| 21 | Axon guidance | 4360 | 128 | 81 | 0.012196 | 2.916274 | 0.052 | 0.005304 | 0.0346 | 0.726604 |
| 22 | Thyroid cancer | 5216 | 29 | 21 | 0.02678 | 1.12333 | 0.025 | 0.005563 | 0.034642 | 0.76212 |
| 23 | Dilated cardiomyopathy | 5414 | 90 | 62 | 0.001592 | -0.22393 | 0.499 | 0.006467 | 0.038518 | 0.885917 |
| 24 | p53 signaling pathway | 4115 | 67 | 48 | 0.001436 | 0.351418 | 0.679 | 0.007736 | 0.042123 | 1 |
| 25 | Maturity onset diabetes of the young | 4950 | 23 | 16 | 0.082684 | -2.07236 | 0.012 | 0.007854 | 0.042123 | 1 |
| 26 | Apoptosis | 4210 | 86 | 52 | 0.101252 | 3.587878 | 0.01 | 0.007994 | 0.042123 | 1 |
| 27 | Viral myocarditis | 5416 | 66 | 43 | 0.03134 | 1.748725 | 0.042 | 0.010047 | 0.05098 | 1 |
| 28 | Neuroactive ligand-receptor interaction | 4080 | 264 | 152 | 0.07745 | -1.39016 | 0.02 | 0.011571 | 0.056616 | 1 |
| 29 | Colorectal cancer | 5210 | 62 | 40 | 0.045198 | 1.477059 | 0.039 | 0.01294 | 0.0583 | 1 |
| 30 | Pancreatic cancer | 5212 | 69 | 46 | 0.015075 | 1.995687 | 0.119 | 0.013137 | 0.0583 | 1 |
| 31 | Leukocyte transendothelial migration | 4670 | 113 | 75 | 0.00274 | 0.861113 | 0.658 | 0.013194 | 0.0583 | 1 |
| 32 | Tight junction | 4530 | 131 | 84 | 0.006632 | 0.85937 | 0.282 | 0.013618 | 0.0583 | 1 |
| 33 | Herpes simplex infection | 5168 | 171 | 94 | 0.334784 | 3.920664 | 0.006 | 0.014483 | 0.060128 | 1 |
| 34 | Antigen processing and presentation | 4612 | 63 | 35 | 0.393667 | 2.410008 | 0.01 | 0.025736 | 0.102946 | 1 |
| 35 | Amoebiasis | 5146 | 106 | 64 | 0.07767 | -1.37226 | 0.052 | 0.0263 | 0.102946 | 1 |
| 36 | TGF-beta signaling pathway | 4350 | 81 | 53 | 0.016107 | -1.2424 | 0.265 | 0.027559 | 0.103095 | 1 |
| 37 | MAPK signaling pathway | 4010 | 259 | 157 | 0.008137 | -0.94602 | 0.531 | 0.027843 | 0.103095 | 1 |
| 38 | Protein processing in endoplasmic reticulum | 4141 | 162 | 101 | 0.010361 | -0.57097 | 0.433 | 0.028743 | 0.103626 | 1 |
| 39 | Basal cell carcinoma | 5217 | 54 | 37 | 0.015126 | 0.598375 | 0.436 | 0.039711 | 0.139496 | 1 |
| 40 | Shigellosis | 5131 | 60 | 40 | 0.022576 | 0.836627 | 0.304 | 0.041052 | 0.140603 | 1 |
| 41 | Vibrio cholerae infection | 5110 | 53 | 34 | 0.068144 | 0.851169 | 0.125 | 0.049111 | 0.164104 | 1 |
| 42 | Melanoma | 5218 | 71 | 43 | 0.124519 | -2.13935 | 0.076 | 0.053566 | 0.174728 | 1 |
| 43 | Transcriptional misregulation in cancer | 5202 | 158 | 98 | 0.013912 | -0.01082 | 0.766 | 0.059054 | 0.181197 | 1 |
| 44 | Influenza A | 5164 | 160 | 92 | 0.146221 | 2.034893 | 0.073 | 0.059134 | 0.181197 | 1 |
| 45 | Renal cell carcinoma | 5211 | 69 | 39 | 0.324401 | 1.847606 | 0.034 | 0.060742 | 0.181197 | 1 |
| 46 | Insulin signaling pathway | 4910 | 136 | 78 | 0.178248 | -3.08235 | 0.062 | 0.06084 | 0.181197 | 1 |
| 47 | Fc gamma R-mediated phagocytosis | 4666 | 91 | 56 | 0.063583 | 1.758777 | 0.183 | 0.063458 | 0.184972 | 1 |
| 48 | Viral carcinogenesis | 5203 | 182 | 109 | 0.036925 | 0.181855 | 0.383 | 0.074368 | 0.212259 | 1 |
| 49 | Chronic myeloid leukemia | 5220 | 72 | 41 | 0.293671 | 2.267297 | 0.062 | 0.091146 | 0.254195 | 1 |
| 50 | Non-small cell lung cancer | 5223 | 54 | 35 | 0.054113 | 0.852623 | 0.344 | 0.092772 | 0.254195 | 1 |
| 51 | Melanogenesis | 4916 | 99 | 60 | 0.078949 | 1.81764 | 0.242 | 0.094721 | 0.254448 | 1 |
| 52 | Epithelial cell signaling in Helicobacter pylori infection | 5120 | 67 | 40 | 0.166097 | 1.280302 | 0.136 | 0.108209 | 0.281786 | 1 |
| 53 | Morphine addiction | 5032 | 89 | 49 | 0.393128 | -1.38972 | 0.058 | 0.109012 | 0.281786 | 1 |
| 54 | Alzheimer's disease | 5010 | 159 | 85 | 0.491187 | 2.239235 | 0.052 | 0.119215 | 0.296526 | 1 |
| 55 | Natural killer cell mediated cytotoxicity | 4650 | 127 | 68 | 0.49183 | 5.558154 | 0.053 | 0.121136 | 0.296526 | 1 |
| 56 | HTLV-I infection | 5166 | 256 | 149 | 0.054575 | -1.15574 | 0.478 | 0.121208 | 0.296526 | 1 |
| 57 | RNA degradation | 3018 | 69 | 44 | 0.04706 | -0.1293 | 0.574 | 0.124566 | 0.299395 | 1 |
| 58 | Circadian rhythm | 4710 | 21 | 14 | 0.15094 | -0.86994 | 0.187 | 0.128922 | 0.304522 | 1 |
| 59 | Endometrial cancer | 5213 | 52 | 31 | 0.209224 | 1.290355 | 0.145 | 0.136378 | 0.316675 | 1 |
| 60 | Oocyte meiosis | 4114 | 109 | 61 | 0.304008 | 2.561706 | 0.105 | 0.141872 | 0.323941 | 1 |
| 61 | Glutamatergic synapse | 4724 | 119 | 72 | 0.061067 | -0.6211 | 0.552 | 0.147982 | 0.332352 | 1 |
| 62 | Aldosterone-regulated sodium reabsorption | 4960 | 39 | 26 | 0.060077 | -0.14962 | 0.639 | 0.163537 | 0.351133 | 1 |
| 63 | Long-term depression | 4730 | 65 | 42 | 0.039486 | 0.035064 | 0.984 | 0.16505 | 0.351133 | 1 |
| 64 | Rheumatoid arthritis | 5323 | 83 | 46 | 0.374057 | 0.779787 | 0.105 | 0.166418 | 0.351133 | 1 |
| 65 | Lysosome | 4142 | 117 | 72 | 0.039331 | 0 | 1 | 0.166596 | 0.351133 | 1 |
| 66 | NOD-like receptor signaling pathway | 4621 | 57 | 30 | 0.578783 | 1.348407 | 0.07 | 0.170409 | 0.353727 | 1 |
| 67 | Hedgehog signaling pathway | 4340 | 54 | 34 | 0.091999 | -0.56358 | 0.486 | 0.183653 | 0.368009 | 1 |
| 68 | Intestinal immune network for IgA production | 4672 | 44 | 21 | 0.805092 | 0.718715 | 0.056 | 0.184813 | 0.368009 | 1 |
| 69 | RNA transport | 3013 | 145 | 85 | 0.102645 | -0.25215 | 0.442 | 0.185693 | 0.368009 | 1 |
| 70 | Serotonergic synapse | 4726 | 114 | 66 | 0.172384 | -0.78066 | 0.271 | 0.189839 | 0.368009 | 1 |
| 71 | Carbohydrate digestion and absorption | 4973 | 38 | 23 | 0.223829 | -0.22455 | 0.21 | 0.19072 | 0.368009 | 1 |
| 72 | RIG-I-like receptor signaling pathway | 4622 | 70 | 36 | 0.653252 | 1.466642 | 0.077 | 0.200686 | 0.379632 | 1 |
| 73 | Adipocytokine signaling pathway | 4920 | 68 | 40 | 0.202536 | 0.872235 | 0.251 | 0.202286 | 0.379632 | 1 |
| 74 | VEGF signa | 4370 | 71 | 44 | 0.081736 | 0.253899 | 0.818 | 0.247727 | 0.458071 | 1 |
| 75 | Prostate cancer | 5215 | 89 | 54 | 0.09005 | -0.4552 | 0.755 | 0.250769 | 0.458071 | 1 |
| 76 | ErbB signaling pathway | 4012 | 87 | 51 | 0.174862 | 1.126385 | 0.399 | 0.255536 | 0.460637 | 1 |
| 77 | Cytosolic DNA-sensing pathway | 4623 | 59 | 26 | 0.935062 | 1.050271 | 0.076 | 0.258972 | 0.460768 | 1 |
| 78 | Phototransduction | 4744 | 28 | 18 | 0.158072 | 0.467052 | 0.46 | 0.263311 | 0.462483 | 1 |
| 79 | Gap junction | 4540 | 85 | 51 | 0.119239 | 0.620967 | 0.627 | 0.268655 | 0.464689 | 1 |
| 80 | Vascular smooth muscle contraction | 4270 | 110 | 65 | 0.11939 | -0.61673 | 0.637 | 0.271985 | 0.464689 | 1 |
| 81 | Toxoplasmosis | 5145 | 120 | 68 | 0.241014 | 1.027215 | 0.32 | 0.274743 | 0.464689 | 1 |
| 82 | Chagas disease (American trypanosomiasis) | 5142 | 101 | 51 | 0.731354 | 1.931046 | 0.109 | 0.281344 | 0.470051 | 1 |
| 83 | Toll-like receptor signaling pathway | 4620 | 98 | 36 | 0.999602 | 2.514388 | 0.089 | 0.304216 | 0.502139 | 1 |
| 84 | Bladder cancer | 5219 | 41 | 26 | 0.119707 | 0.178341 | 0.804 | 0.321539 | 0.524415 | 1 |
| 85 | Long-term potentiation | 4720 | 69 | 39 | 0.324401 | -1.20803 | 0.314 | 0.334529 | 0.539182 | 1 |
| 86 | Dopaminergic synapse | 4728 | 128 | 70 | 0.389174 | -0.87315 | 0.273 | 0.344446 | 0.54871 | 1 |
| 87 | Fanconi anemia pathway | 3460 | 48 | 25 | 0.610864 | 0.867697 | 0.182 | 0.355392 | 0.549636 | 1 |
| 88 | Dorso-ventral axis formation | 4320 | 23 | 15 | 0.168546 | 0.052634 | 0.667 | 0.358115 | 0.549636 | 1 |
| 89 | PPAR signaling pathway | 3320 | 69 | 40 | 0.242668 | 0.213964 | 0.473 | 0.363253 | 0.549636 | 1 |
| 90 | Epstein-Barr virus infection | 5169 | 189 | 108 | 0.144696 | -0.32012 | 0.797 | 0.364422 | 0.549636 | 1 |
| 91 | Type I diabetes mellitus | 4940 | 39 | 20 | 0.64961 | 0.45178 | 0.178 | 0.365087 | 0.549636 | 1 |
| 92 | Amphetamine addiction | 5031 | 69 | 38 | 0.415434 | -0.72957 | 0.285 | 0.371026 | 0.550856 | 1 |
| 93 | NF-kappa B signaling pathway | 4064 | 88 | 45 | 0.680499 | 1.842664 | 0.176 | 0.373939 | 0.550856 | 1 |
| 94 | Hepatitis C | 5160 | 128 | 74 | 0.160125 | 0.413063 | 0.776 | 0.383383 | 0.557941 | 1 |
| 95 | Asthma | 5310 | 26 | 6 | 0.999569 | -0.02011 | 0.126 | 0.386893 | 0.557941 | 1 |
| 96 | Graft-versus-host disease | 5332 | 34 | 16 | 0.808659 | 0.45178 | 0.161 | 0.395625 | 0.562187 | 1 |
| 97 | Complement and coagulation cascades | 4610 | 67 | 36 | 0.505323 | -4.18274 | 0.26 | 0.398045 | 0.562187 | 1 |
| 98 | Bile secretion | 4976 | 71 | 40 | 0.33199 | -0.1758 | 0.434 | 0.423226 | 0.585887 | 1 |
| 99 | Allograft rejection | 5330 | 33 | 15 | 0.853032 | 0.431672 | 0.169 | 0.423378 | 0.585887 | 1 |
| 100 | Autoimmune thyroid disease | 5320 | 47 | 20 | 0.944061 | 0.431672 | 0.171 | 0.455835 | 0.624494 | 1 |
| 101 | Fc epsilon RI signaling pathway | 4664 | 73 | 35 | 0.839697 | 1.537644 | 0.199 | 0.466069 | 0.632192 | 1 |
| 102 | Chemokine signaling pathway | 4062 | 179 | 88 | 0.86912 | 2.27689 | 0.204 | 0.484014 | 0.650098 | 1 |
| 103 | Osteoclast differentiation | 4380 | 129 | 69 | 0.496199 | 0.966284 | 0.387 | 0.508898 | 0.676883 | 1 |
| 104 | Neurotrophin signaling pathway | 4722 | 119 | 66 | 0.331894 | 0.803324 | 0.603 | 0.5221 | 0.687766 | 1 |
| 105 | Retrograde endocannabinoid signaling | 4723 | 100 | 52 | 0.622933 | -0.26847 | 0.328 | 0.528797 | 0.689954 | 1 |
| 106 | Taste transduction | 4742 | 45 | 18 | 0.971739 | -0.85642 | 0.221 | 0.545103 | 0.704519 | 1 |
| 107 | GABAergic synapse | 4727 | 87 | 49 | 0.307499 | -0.17089 | 0.721 | 0.555686 | 0.711485 | 1 |
| 108 | Legionellosis | 5134 | 51 | 26 | 0.669631 | 0.842927 | 0.356 | 0.580203 | 0.726192 | 1 |
| 109 | SNARE interactions in vesicular transport | 4130 | 35 | 16 | 0.850796 | 0.482295 | 0.283 | 0.583613 | 0.726192 | 1 |
| 110 | Cytokine-cytokine receptor interaction | 4060 | 251 | 125 | 0.86472 | 1.969111 | 0.284 | 0.590407 | 0.726192 | 1 |
| 111 | Type II diabetes mellitus | 4930 | 47 | 27 | 0.32449 | -0.13308 | 0.76 | 0.591854 | 0.726192 | 1 |
| 112 | Glioma | 5214 | 64 | 37 | 0.262067 | 0.080419 | 0.946 | 0.593675 | 0.726192 | 1 |
| 113 | B cell receptor signaling pathway | 4662 | 75 | 41 | 0.435464 | 0.350656 | 0.581 | 0.600721 | 0.728308 | 1 |
| 114 | Olfactory transduction | 4740 | 112 | 52 | 0.933477 | -1.47457 | 0.289 | 0.623225 | 0.746786 | 1 |
| 115 | Acute myeloid leukemia | 5221 | 56 | 26 | 0.870378 | 0.788018 | 0.321 | 0.635655 | 0.746786 | 1 |
| 116 | GnRH signaling pathway | 4912 | 94 | 50 | 0.531196 | 0.878776 | 0.539 | 0.644398 | 0.746786 | 1 |
| 117 | Parkinson's disease | 5012 | 116 | 53 | 0.954036 | 0.922927 | 0.301 | 0.64546 | 0.746786 | 1 |
| 118 | Huntington's disease | 5016 | 171 | 89 | 0.633998 | 0.489128 | 0.456 | 0.647872 | 0.746786 | 1 |
| 119 | Systemic lupus erythematosus | 5322 | 81 | 38 | 0.888788 | 1.386547 | 0.326 | 0.648668 | 0.746786 | 1 |
| 120 | mTOR signaling pathway | 4150 | 62 | 28 | 0.915201 | 0.419433 | 0.36 | 0.695273 | 0.79377 | 1 |
| 121 | Sulfur relay system | 4122 | 9 | 5 | 0.574731 | 0.09504 | 0.594 | 0.708292 | 0.801951 | 1 |
| 122 | Cocaine addiction | 5030 | 50 | 25 | 0.71769 | -0.32326 | 0.515 | 0.737486 | 0.82816 | 1 |
| 123 | T cell receptor signaling pathway | 4660 | 108 | 59 | 0.408703 | -0.06961 | 0.93 | 0.747772 | 0.8299 | 1 |
| 124 | Measles | 5162 | 128 | 69 | 0.458672 | 0.234444 | 0.847 | 0.755807 | 0.8299 | 1 |
| 125 | Jak-STAT signaling pathway | 4630 | 153 | 80 | 0.607445 | -0.28278 | 0.642 | 0.757208 | 0.8299 | 1 |
| 126 | Regulation of autophagy | 4140 | 33 | 11 | 0.993046 | 0.482746 | 0.466 | 0.819338 | 0.887219 | 1 |
| 127 | Phosphatid | 4070 | 79 | 42 | 0.537798 | 0.04929 | 0.874 | 0.824887 | 0.887219 | 1 |
| 128 | African trypanosomiasis | 5143 | 32 | 16 | 0.700203 | 0.104532 | 0.679 | 0.828935 | 0.887219 | 1 |
| 129 | Prion diseases | 5020 | 34 | 15 | 0.888063 | 0.143661 | 0.598 | 0.867158 | 0.916105 | 1 |
| 130 | Pertussis | 5133 | 69 | 35 | 0.695003 | -0.58389 | 0.769 | 0.869297 | 0.916105 | 1 |
| 131 | Alcoholism | 5034 | 128 | 59 | 0.952523 | -0.50593 | 0.6 | 0.891257 | 0.932078 | 1 |
| 132 | Vasopressin-regulated water reabsorption | 4962 | 44 | 23 | 0.601493 | 0.001941 | 0.994 | 0.905411 | 0.939707 | 1 |
| 133 | Progesterone-mediated oocyte maturation | 4914 | 84 | 42 | 0.749178 | -0.16063 | 0.871 | 0.931095 | 0.959098 | 1 |
| 134 | Cholinergic synapse | 4725 | 109 | 54 | 0.797543 | 0.144345 | 0.904 | 0.956844 | 0.978266 | 1 |
| 135 | Leishmaniasis | 5140 | 66 | 32 | 0.807417 | -0.00773 | 0.987 | 0.977822 | 0.992308 | 1 |
| 136 | Malaria | 5144 | 47 | 22 | 0.84251 | 0 | 1 | 0.986891 | 0.994147 | 1 |
| 137 | Staphylococcus aureus infection | 5150 | 48 | 16 | 0.998141 | -0.17434 | 0.935 | 0.997722 | 0.997722 | 1 |

Result obtained by the MSPIA methods in the pancreatic cancer dataset

| No | Name | ID | pSize | NDE | pNDE | tA | pPERT | pG | pGFdr | pGFWER |
| --- | --- | --- | --- | --- | --- | --- | --- | --- | --- | --- |
| 1 | ECM-receptor interaction | 4512 | 83 | 64 | 5.01E-06 | 2.115915 | 5.00E-06 | 6.36E-10 | 8.84E-08 | 8.84E-08 |
| 2 | Small cell lung cancer | 5222 | 83 | 61 | 0.000105 | 1.993039 | 5.00E-06 | 1.18E-08 | 6.74E-07 | 1.63E-06 |
| 3 | Focal adhesion | 4510 | 199 | 140 | 4.23E-07 | 2.635503 | 0.002 | 1.85E-08 | 6.74E-07 | 2.57E-06 |
| 4 | Pathways in cancer | 5200 | 321 | 221 | 4.65E-09 | 1.356148 | 0.191 | 1.94E-08 | 6.74E-07 | 2.70E-06 |
| 5 | Bacterial invasion of epithelial cells | 5100 | 70 | 51 | 0.000541 | 1.883855 | 5.00E-06 | 5.61E-08 | 1.56E-06 | 7.79E-06 |
| 6 | Regulation of actin cytoskeleton | 4810 | 212 | 144 | 6.86E-06 | 1.038289 | 0.063 | 6.76E-06 | 0.000157 | 0.00094 |
| 7 | Arrhythmogenic right ventricular cardiomyopathy (ARVC) | 5412 | 74 | 57 | 1.74E-05 | 0.08917 | 0.054 | 1.40E-05 | 0.000277 | 0.001941 |
| 8 | Cell cycle | 4110 | 122 | 84 | 0.000267 | -1.1539 | 0.024 | 8.30E-05 | 0.001442 | 0.011537 |
| 9 | p53 signaling pathway | 4115 | 67 | 48 | 0.001436 | -0.35967 | 0.033 | 0.000519 | 0.007618 | 0.072181 |
| 10 | Pancreatic secretion | 4972 | 91 | 63 | 0.001203 | -0.10285 | 0.044 | 0.000574 | 0.007618 | 0.079783 |
| 11 | Salmonella infection | 5132 | 80 | 50 | 0.055856 | 1.855747 | 0.001 | 0.000603 | 0.007618 | 0.083794 |
| 12 | Pathogenic Escherichia coli infection | 5130 | 51 | 35 | 0.017289 | 1.128827 | 0.004 | 0.000732 | 0.007778 | 0.101692 |
| 13 | Endocrine and other factor-regulated calcium reabsorption | 4961 | 49 | 38 | 0.000348 | -0.36236 | 0.212 | 0.000776 | 0.007778 | 0.107906 |
| 14 | Wnt signaling pathway | 4310 | 149 | 99 | 0.000601 | 0.39648 | 0.124 | 0.000783 | 0.007778 | 0.108892 |
| 15 | Mineral absorption | 4978 | 49 | 37 | 0.00103 | 0.041408 | 0.088 | 0.000934 | 0.008657 | 0.129859 |
| 16 | Gastric acid secretion | 4971 | 73 | 46 | 0.055053 | -0.54623 | 0.002 | 0.001114 | 0.009675 | 0.154792 |
| 17 | Notch signaling pathway | 4330 | 47 | 33 | 0.012474 | -0.82084 | 0.016 | 0.0019 | 0.015535 | 0.264088 |
| 18 | Apoptosis | 4210 | 86 | 52 | 0.101252 | 0.779513 | 0.004 | 0.003569 | 0.025875 | 0.496057 |
| 19 | Maturity onset diabetes of the young | 4950 | 23 | 16 | 0.082684 | -0.75962 | 0.005 | 0.003634 | 0.025875 | 0.50518 |
| 20 | Protein processing in endoplasmic reticulum | 4141 | 162 | 101 | 0.010361 | -0.42313 | 0.041 | 0.003723 | 0.025875 | 0.517494 |
| 21 | Axon guidance | 4360 | 128 | 81 | 0.012196 | 0.794924 | 0.044 | 0.004577 | 0.028921 | 0.636254 |
| 22 | Calcium signaling pathway | 4020 | 180 | 115 | 0.002039 | -0.27845 | 0.287 | 0.004941 | 0.029862 | 0.686827 |
| 23 | Amyotrophic lateral sclerosis (ALS) | 5014 | 52 | 39 | 0.000951 | 0.294884 | 0.66 | 0.005254 | 0.030427 | 0.730246 |
| 24 | Colorectal cancer | 5210 | 62 | 40 | 0.045198 | 0.2769 | 0.018 | 0.006601 | 0.036101 | 0.917584 |
| 25 | Amoebiasis | 5146 | 106 | 64 | 0.07767 | -0.45592 | 0.011 | 0.006891 | 0.036101 | 0.957791 |
| 26 | Melanoma | 5218 | 71 | 43 | 0.124519 | -0.88692 | 0.007 | 0.007012 | 0.036101 | 0.974728 |
| 27 | Tight junction | 4530 | 131 | 84 | 0.006632 | 0.39115 | 0.157 | 0.008191 | 0.040663 | 1 |
| 28 | Leukocyte transendothelial migration | 4670 | 113 | 75 | 0.00274 | 0.359106 | 0.437 | 0.009253 | 0.04435 | 1 |
| 29 | Dilated cardiomyopathy | 5414 | 90 | 62 | 0.001592 | -0.01624 | 0.841 | 0.010199 | 0.047257 | 1 |
| 30 | MAPK signaling pathway | 4010 | 259 | 157 | 0.008137 | -0.66586 | 0.185 | 0.011288 | 0.050612 | 1 |
| 31 | Salivary secretion | 4970 | 82 | 53 | 0.022315 | -0.36863 | 0.082 | 0.013364 | 0.058051 | 1 |
| 32 | Pancreatic cancer | 5212 | 69 | 46 | 0.015075 | 0.594518 | 0.133 | 0.01446 | 0.059207 | 1 |
| 33 | Thyroid cancer | 5216 | 29 | 21 | 0.02678 | 0.19143 | 0.075 | 0.014482 | 0.059207 | 1 |
| 34 | Viral myocarditis | 5416 | 66 | 43 | 0.03134 | 0.43931 | 0.094 | 0.020113 | 0.079878 | 1 |
| 35 | Tuberculosis | 5152 | 172 | 98 | 0.168746 | 1.679751 | 0.027 | 0.02912 | 0.112434 | 1 |
| 36 | Antigen processing and presentation | 4612 | 63 | 35 | 0.393667 | 1.563977 | 0.012 | 0.030022 | 0.112784 | 1 |
| 37 | Insulin signaling pathway | 4910 | 136 | 78 | 0.178248 | -1.26206 | 0.041 | 0.043255 | 0.155859 | 1 |
| 38 | Shigellosis | 5131 | 60 | 40 | 0.022576 | 0.091685 | 0.328 | 0.04373 | 0.155859 | 1 |
| 39 | Neuroactive ligand-receptor interaction | 4080 | 264 | 152 | 0.07745 | -0.46786 | 0.101 | 0.045767 | 0.159041 | 1 |
| 40 | Basal cell carcinoma | 5217 | 54 | 37 | 0.015126 | 0.134047 | 0.555 | 0.048523 | 0.164505 | 1 |
| 41 | Melanogenesis | 4916 | 99 | 60 | 0.078949 | 0.673921 | 0.117 | 0.052508 | 0.168398 | 1 |
| 42 | Natural killer cell mediated cytotoxicity | 4650 | 127 | 68 | 0.49183 | 4.470076 | 0.019 | 0.053012 | 0.168398 | 1 |
| 43 | Renal cell carcinoma | 5211 | 69 | 39 | 0.324401 | 0.466878 | 0.029 | 0.053306 | 0.168398 | 1 |
| 44 | Transcriptional misregulation in cancer | 5202 | 158 | 98 | 0.013912 | ######## | 0.716 | 0.055872 | 0.172582 | 1 |
| 45 | TGF-beta signaling pathway | 4350 | 81 | 53 | 0.016107 | -0.17492 | 0.705 | 0.062207 | 0.183973 | 1 |
| 46 | Prostate cancer | 5215 | 89 | 54 | 0.09005 | -0.56801 | 0.166 | 0.077779 | 0.225234 | 1 |
| 47 | Viral carcinogenesis | 5203 | 182 | 109 | 0.036925 | 0.018083 | 0.439 | 0.08303 | 0.235534 | 1 |
| 48 | RNA transport | 3013 | 145 | 85 | 0.102645 | -0.21446 | 0.186 | 0.094668 | 0.261519 | 1 |
| 49 | Herpes simplex infection | 5168 | 171 | 94 | 0.334784 | 0.607546 | 0.058 | 0.095953 | 0.261519 | 1 |
| 50 | RNA degradation | 3018 | 69 | 44 | 0.04706 | -0.02469 | 0.432 | 0.099528 | 0.266045 | 1 |
| 51 | Vibrio cholerae infection | 5110 | 53 | 34 | 0.068144 | 0.075424 | 0.326 | 0.106788 | 0.280066 | 1 |
| 52 | Aldosterone-regulated sodium reabsorption | 4960 | 39 | 26 | 0.060077 | -0.04166 | 0.418 | 0.117635 | 0.302802 | 1 |
| 53 | HTLV-I infection | 5166 | 256 | 149 | 0.054575 | -0.23367 | 0.499 | 0.125362 | 0.316823 | 1 |
| 54 | Chronic myeloid leukemia | 5220 | 72 | 41 | 0.293671 | 0.395938 | 0.096 | 0.128803 | 0.319707 | 1 |
| 55 | Serotonergic synapse | 4726 | 114 | 66 | 0.172384 | -0.35452 | 0.171 | 0.133361 | 0.321337 | 1 |
| 56 | Influenza A | 5164 | 160 | 92 | 0.146221 | 0.605202 | 0.203 | 0.134083 | 0.321337 | 1 |
| 57 | Fc gamma R-mediated phagocytosis | 4666 | 91 | 56 | 0.063583 | 0.480634 | 0.486 | 0.138345 | 0.325499 | 1 |
| 58 | Bladder cancer | 5219 | 41 | 26 | 0.119707 | -0.09978 | 0.264 | 0.140774 | 0.325499 | 1 |
| 59 | Intestinal immune network for IgA production | 4672 | 44 | 21 | 0.805092 | 0.3453 | 0.04 | 0.142845 | 0.325499 | 1 |
| 60 | Non-small cell lung cancer | 5223 | 54 | 35 | 0.054113 | 0.072969 | 0.685 | 0.159204 | 0.3542 | 1 |
| 61 | Long-term depression | 4730 | 65 | 42 | 0.039486 | 0.051899 | 0.949 | 0.160537 | 0.3542 | 1 |
| 62 | Lysosome | 4142 | 117 | 72 | 0.039331 | 0 | 1 | 0.166596 | 0.361825 | 1 |
| 63 | PPAR signaling pathway | 3320 | 69 | 40 | 0.242668 | 0.137825 | 0.176 | 0.177387 | 0.37454 | 1 |
| 64 | Epithelial cell signaling in Helicobacter pylori infection | 5120 | 67 | 40 | 0.166097 | 0.139951 | 0.258 | 0.177839 | 0.37454 | 1 |
| 65 | VEGF signa | 4370 | 71 | 44 | 0.081736 | -0.21398 | 0.552 | 0.184916 | 0.383632 | 1 |
| 66 | Glutamatergic synapse | 4724 | 119 | 72 | 0.061067 | -0.10436 | 0.795 | 0.195416 | 0.399452 | 1 |
| 67 | NOD-like receptor signaling pathway | 4621 | 57 | 30 | 0.578783 | 0.251141 | 0.096 | 0.216154 | 0.423656 | 1 |
| 68 | Carbohydrate digestion and absorption | 4973 | 38 | 23 | 0.223829 | -0.09032 | 0.25 | 0.217291 | 0.423656 | 1 |
| 69 | Chagas disease (American trypanosomiasis) | 5142 | 101 | 51 | 0.731354 | 0.568496 | 0.08 | 0.224589 | 0.423656 | 1 |
| 70 | Gap junction | 4540 | 85 | 51 | 0.119239 | 0.192144 | 0.493 | 0.225373 | 0.423656 | 1 |
| 71 | Long-term potentiation | 4720 | 69 | 39 | 0.324401 | -0.30387 | 0.185 | 0.228845 | 0.423656 | 1 |
| 72 | Amphetamine addiction | 5031 | 69 | 38 | 0.415434 | -0.28127 | 0.149 | 0.23412 | 0.423656 | 1 |
| 73 | Allograft rejection | 5330 | 33 | 15 | 0.853032 | 0.283543 | 0.074 | 0.237515 | 0.423656 | 1 |
| 74 | Autoimmune thyroid disease | 5320 | 47 | 20 | 0.944061 | 0.283543 | 0.067 | 0.237868 | 0.423656 | 1 |
| 75 | Phototransduction | 4744 | 28 | 18 | 0.158072 | 0.258774 | 0.401 | 0.23824 | 0.423656 | 1 |
| 76 | Rheumatoid arthritis | 5323 | 83 | 46 | 0.374057 | 0.21782 | 0.171 | 0.239828 | 0.423656 | 1 |
| 77 | Type I diabetes mellitus | 4940 | 39 | 20 | 0.64961 | 0.283688 | 0.099 | 0.240783 | 0.423656 | 1 |
| 78 | RIG-I-like receptor signaling pathway | 4622 | 70 | 36 | 0.653252 | 0.334533 | 0.103 | 0.248875 | 0.428202 | 1 |
| 79 | Hedgehog signaling pathway | 4340 | 54 | 34 | 0.091999 | -0.07672 | 0.734 | 0.249528 | 0.428202 | 1 |
| 80 | Circadian rhythm | 4710 | 21 | 14 | 0.15094 | -0.10241 | 0.486 | 0.264996 | 0.448325 | 1 |
| 81 | Graft-versus-host disease | 5332 | 34 | 16 | 0.808659 | 0.283688 | 0.092 | 0.267705 | 0.448325 | 1 |
| 82 | ErbB signaling pathway | 4012 | 87 | 51 | 0.174862 | 0.255854 | 0.449 | 0.278288 | 0.456325 | 1 |
| 83 | Toxoplasmosis | 5145 | 120 | 68 | 0.241014 | 0.197559 | 0.327 | 0.279048 | 0.456325 | 1 |
| 84 | Cytosolic DNA-sensing pathway | 4623 | 59 | 26 | 0.935062 | 0.236718 | 0.087 | 0.285458 | 0.45933 | 1 |
| 85 | Morphine addiction | 5032 | 89 | 49 | 0.393128 | -0.24826 | 0.209 | 0.287494 | 0.45933 | 1 |
| 86 | Alzheimer's disease | 5010 | 159 | 85 | 0.491187 | 0.356347 | 0.173 | 0.294473 | 0.465134 | 1 |
| 87 | Vascular smooth muscle contraction | 4270 | 110 | 65 | 0.11939 | -0.11811 | 0.752 | 0.306187 | 0.478203 | 1 |
| 88 | Adipocytokine signaling pathway | 4920 | 68 | 40 | 0.202536 | 0.144128 | 0.526 | 0.345095 | 0.53298 | 1 |
| 89 | Fc epsilon RI signaling pathway | 4664 | 73 | 35 | 0.839697 | 0.970945 | 0.131 | 0.352801 | 0.538894 | 1 |
| 90 | Epstein-Barr virus infection | 5169 | 189 | 108 | 0.144696 | -0.09469 | 0.786 | 0.360973 | 0.545383 | 1 |
| 91 | Complement and coagulation cascades | 4610 | 67 | 36 | 0.505323 | -3.38514 | 0.255 | 0.392892 | 0.574593 | 1 |
| 92 | Asthma | 5310 | 26 | 6 | 0.999569 | -0.00014 | 0.129 | 0.393071 | 0.574593 | 1 |
| 93 | Huntington's disease | 5016 | 171 | 89 | 0.633998 | 0.101861 | 0.206 | 0.396459 | 0.574593 | 1 |
| 94 | Dorso-ventral axis formation | 4320 | 23 | 15 | 0.168546 | 0.012697 | 0.776 | 0.396841 | 0.574593 | 1 |
| 95 | Osteoclast differentiation | 4380 | 129 | 69 | 0.496199 | 0.282943 | 0.279 | 0.412179 | 0.585395 | 1 |
| 96 | Endometrial cancer | 5213 | 52 | 31 | 0.209224 | 0.082709 | 0.663 | 0.412724 | 0.585395 | 1 |
| 97 | SNARE interactions in vesicular transport | 4130 | 35 | 16 | 0.850796 | 0.101705 | 0.167 | 0.419336 | 0.588764 | 1 |
| 98 | Hepatitis C | 5160 | 128 | 74 | 0.160125 | 0.008073 | 0.975 | 0.446059 | 0.620022 | 1 |
| 99 | Dopaminergic synapse | 4728 | 128 | 70 | 0.389174 | -0.17052 | 0.46 | 0.48698 | 0.665391 | 1 |
| 100 | Taste transduction | 4742 | 45 | 18 | 0.971739 | -0.72056 | 0.185 | 0.488272 | 0.665391 | 1 |
| 101 | Toll-like receptor signaling pathway | 4620 | 98 | 36 | 0.999602 | 0.738501 | 0.184 | 0.495355 | 0.666062 | 1 |
| 102 | Regulation of autophagy | 4140 | 33 | 11 | 0.993046 | 0.407663 | 0.187 | 0.498348 | 0.666062 | 1 |
| 103 | Jak-STAT signaling pathway | 4630 | 153 | 80 | 0.607445 | -0.12463 | 0.321 | 0.513761 | 0.680122 | 1 |
| 104 | Type II diabetes mellitus | 4930 | 47 | 27 | 0.32449 | 0.048776 | 0.66 | 0.544192 | 0.713611 | 1 |
| 105 | Glioma | 5214 | 64 | 37 | 0.262067 | -0.05361 | 0.832 | 0.550132 | 0.714657 | 1 |
| 106 | Chemokine signaling pathway | 4062 | 179 | 88 | 0.86912 | 0.872693 | 0.258 | 0.559477 | 0.719467 | 1 |
| 107 | Oocyte meiosis | 4114 | 109 | 61 | 0.304008 | 0.120682 | 0.748 | 0.564186 | 0.719467 | 1 |
| 108 | Fanconi anemia pathway | 3460 | 48 | 25 | 0.610864 | 0.321248 | 0.398 | 0.586946 | 0.735195 | 1 |
| 109 | GABAergic synapse | 4727 | 87 | 49 | 0.307499 | -0.03428 | 0.791 | 0.587098 | 0.735195 | 1 |
| 110 | Progesterone-mediated oocyte maturation | 4914 | 84 | 42 | 0.749178 | 0.202226 | 0.344 | 0.607154 | 0.753521 | 1 |
| 111 | Bile secretion | 4976 | 71 | 40 | 0.33199 | -0.01114 | 0.812 | 0.622965 | 0.766302 | 1 |
| 112 | Pertussis | 5133 | 69 | 35 | 0.695003 | -1.53249 | 0.398 | 0.632096 | 0.770713 | 1 |
| 113 | Alcoholism | 5034 | 128 | 59 | 0.952523 | -0.26274 | 0.303 | 0.647265 | 0.782347 | 1 |
| 114 | B cell receptor signaling pathway | 4662 | 75 | 41 | 0.435464 | 0.053754 | 0.703 | 0.668512 | 0.801061 | 1 |
| 115 | GnRH signaling pathway | 4912 | 94 | 50 | 0.531196 | 0.478466 | 0.596 | 0.680718 | 0.808717 | 1 |
| 116 | Neurotrophin signaling pathway | 4722 | 119 | 66 | 0.331894 | 0.016719 | 0.971 | 0.687196 | 0.809493 | 1 |
| 117 | Parkinson's disease | 5012 | 116 | 53 | 0.954036 | 0.152065 | 0.348 | 0.698075 | 0.81368 | 1 |
| 118 | NF-kappa B signaling pathway | 4064 | 88 | 45 | 0.680499 | 0.275344 | 0.507 | 0.712166 | 0.81368 | 1 |
| 119 | Cholinergic synapse | 4725 | 109 | 54 | 0.797543 | 0.172357 | 0.437 | 0.715888 | 0.81368 | 1 |
| 120 | Systemic lupus erythematosus | 5322 | 81 | 38 | 0.888788 | 1.22946 | 0.394 | 0.717629 | 0.81368 | 1 |
| 121 | Sulfur relay system | 4122 | 9 | 5 | 0.574731 | 0.004836 | 0.629 | 0.72933 | 0.81368 | 1 |
| 122 | mTOR signaling pathway | 4150 | 62 | 28 | 0.915201 | 0.141495 | 0.396 | 0.730258 | 0.81368 | 1 |
| 123 | Cocaine addiction | 5030 | 50 | 25 | 0.71769 | -0.08036 | 0.507 | 0.731726 | 0.81368 | 1 |
| 124 | Measles | 5162 | 128 | 69 | 0.458672 | -0.05465 | 0.818 | 0.743001 | 0.819659 | 1 |
| 125 | T cell receptor signaling pathway | 4660 | 108 | 59 | 0.408703 | -0.0059 | 0.974 | 0.764749 | 0.831783 | 1 |
| 126 | Vasopressin-regulated water reabsorption | 4962 | 44 | 23 | 0.601493 | 0.028017 | 0.664 | 0.765958 | 0.831783 | 1 |
| 127 | Retrograde endocannabinoid signaling | 4723 | 100 | 52 | 0.622933 | -0.01862 | 0.663 | 0.778223 | 0.83855 | 1 |
| 128 | Phosphatid | 4070 | 79 | 42 | 0.537798 | 0.03153 | 0.872 | 0.824074 | 0.881125 | 1 |
| 129 | Prion diseases | 5020 | 34 | 15 | 0.888063 | 0.034393 | 0.55 | 0.838422 | 0.889624 | 1 |
| 130 | Legionellosis | 5134 | 51 | 26 | 0.669631 | 0.074949 | 0.798 | 0.869239 | 0.915335 | 1 |
| 131 | African trypanosomiasis | 5143 | 32 | 16 | 0.700203 | 0.007342 | 0.821 | 0.893123 | 0.933414 | 1 |
| 132 | Olfactory transduction | 4740 | 112 | 52 | 0.933477 | -0.49737 | 0.656 | 0.912683 | 0.946739 | 1 |
| 133 | Acute myeloid leukemia | 5221 | 56 | 26 | 0.870378 | 0.051333 | 0.762 | 0.935574 | 0.963294 | 1 |
| 134 | Leishmaniasis | 5140 | 66 | 32 | 0.807417 | -0.03279 | 0.898 | 0.958167 | 0.979303 | 1 |
| 135 | Cytokine-cytokine receptor interaction | 4060 | 251 | 125 | 0.86472 | 0.074378 | 0.9 | 0.973363 | 0.987572 | 1 |
| 136 | Malaria | 5144 | 47 | 22 | 0.84251 | 0.000367 | 0.977 | 0.983345 | 0.990471 | 1 |
| 137 | Staphyloco | 5150 | 48 | 16 | 0.998141 | 0.321577 | 0.888 | 0.993282 | 0.993282 | 1 |

Result obtained by the SPIA methods in the pancreatic cancer dataset

| No | Name | ID | pSize | NDE | pNDE | tA | pPERT | pG | pGFdr | pGFWER |
| --- | --- | --- | --- | --- | --- | --- | --- | --- | --- | --- |
| 1 | ECM-receptor interaction | 4512 | 83 | 64 | 5.01E-06 | 27.07024 | 5.00E-06 | 6.36E-10 | 8.71E-08 | 8.71E-08 |
| 2 | Pathways in cancer | 5200 | 321 | 221 | 4.65E-09 | 41.64233 | 0.103 | 1.08E-08 | 5.37E-07 | 1.47E-06 |
| 3 | Small cell lung cancer | 5222 | 83 | 61 | 0.000105 | 47.08389 | 5.00E-06 | 1.18E-08 | 5.37E-07 | 1.61E-06 |
| 4 | Focal adhesion | 4510 | 199 | 140 | 4.23E-07 | 51.30014 | 0.004 | 3.58E-08 | 1.01E-06 | 4.91E-06 |
| 5 | Endocrine and other factor-regulated calcium reabsorption | 4961 | 49 | 38 | 0.000348 | -13.7678 | 5.00E-06 | 3.69E-08 | 1.01E-06 | 5.05E-06 |
| 6 | Regulation of actin cytoskeleton | 4810 | 212 | 144 | 6.86E-06 | 36.33971 | 0.01 | 1.20E-06 | 2.74E-05 | 0.000164 |
| 7 | Arrhythmogenic right ventricular cardiomyopathy (ARVC) | 5412 | 74 | 57 | 1.74E-05 | 2.12172 | 0.049 | 1.28E-05 | 0.00025 | 0.001747 |
| 8 | Pancreatic secretion | 4972 | 91 | 63 | 0.001203 | -3.41603 | 0.039 | 0.000514 | 0.008809 | 0.070474 |
| 9 | Calcium signaling pathway | 4020 | 180 | 115 | 0.002039 | -16.7045 | 0.037 | 0.000792 | 0.012049 | 0.108445 |
| 10 | Wnt signaling pathway | 4310 | 149 | 99 | 0.000601 | 9.061288 | 0.163 | 0.001003 | 0.013741 | 0.137408 |
| 11 | Mineral absorption | 4978 | 49 | 37 | 0.00103 | 1.187319 | 0.112 | 0.001161 | 0.014462 | 0.159087 |
| 12 | Bacterial invasion of epithelial cells | 5100 | 70 | 51 | 0.000541 | 12.56376 | 0.337 | 0.001752 | 0.019962 | 0.239968 |
| 13 | Gastric acid secretion | 4971 | 73 | 46 | 0.055053 | -9.50319 | 0.004 | 0.002075 | 0.019962 | 0.284219 |
| 14 | Cell cycle | 4110 | 122 | 84 | 0.000267 | 1.922495 | 0.834 | 0.002094 | 0.019962 | 0.286937 |
| 15 | Viral myocarditis | 5416 | 66 | 43 | 0.03134 | 12.69012 | 0.008 | 0.00233 | 0.019962 | 0.319143 |
| 16 | Amyotrophic lateral sclerosis (ALS) | 5014 | 52 | 39 | 0.000951 | 5.939738 | 0.264 | 0.002331 | 0.019962 | 0.319397 |
| 17 | Pathogenic Escherichia coli infection | 5130 | 51 | 35 | 0.017289 | 18.89958 | 0.023 | 0.003511 | 0.028295 | 0.481023 |
| 18 | Tuberculosis | 5152 | 172 | 98 | 0.168746 | 38.56684 | 0.003 | 0.004348 | 0.033092 | 0.595653 |
| 19 | Tight junction | 4530 | 131 | 84 | 0.006632 | 7.307726 | 0.11 | 0.005999 | 0.042029 | 0.821802 |
| 20 | Dilated cardiomyopathy | 5414 | 90 | 62 | 0.001592 | -1.09018 | 0.47 | 0.006136 | 0.042029 | 0.84057 |
| 21 | Salivary secretion | 4970 | 82 | 53 | 0.022315 | -6.04513 | 0.041 | 0.007316 | 0.04556 | 1 |
| 22 | Olfactory transduction | 4740 | 112 | 52 | 0.933477 | -30.6225 | 0.001 | 0.007446 | 0.04556 | 1 |
| 23 | Neuroactive ligand-receptor interaction | 4080 | 264 | 152 | 0.07745 | -5.08958 | 0.013 | 0.007955 | 0.04556 | 1 |
| 24 | Complement and coagulation cascades | 4610 | 67 | 36 | 0.505323 | -37.6481 | 0.002 | 0.007981 | 0.04556 | 1 |
| 25 | Thyroid cancer | 5216 | 29 | 21 | 0.02678 | 8.703454 | 0.044 | 0.009125 | 0.050003 | 1 |
| 26 | p53 signaling pathway | 4115 | 67 | 48 | 0.001436 | -0.07649 | 0.983 | 0.010677 | 0.056259 | 1 |
| 27 | Salmonella infection | 5132 | 80 | 50 | 0.055856 | 12.03435 | 0.027 | 0.011306 | 0.057368 | 1 |
| 28 | Colorectal cancer | 5210 | 62 | 40 | 0.045198 | 8.181636 | 0.037 | 0.012364 | 0.058459 | 1 |
| 29 | Herpes simplex infection | 5168 | 171 | 94 | 0.334784 | 23.52553 | 0.005 | 0.012375 | 0.058459 | 1 |
| 30 | Leukocyte transendothelial migration | 4670 | 113 | 75 | 0.00274 | 3.857155 | 0.711 | 0.014106 | 0.062897 | 1 |
| 31 | Antigen processing and presentation | 4612 | 63 | 35 | 0.393667 | 7.182463 | 0.005 | 0.014232 | 0.062897 | 1 |
| 32 | Axon guidance | 4360 | 128 | 81 | 0.012196 | 9.798559 | 0.181 | 0.015708 | 0.06391 | 1 |
| 33 | Apoptosis | 4210 | 86 | 52 | 0.101252 | 20.45827 | 0.022 | 0.015831 | 0.06391 | 1 |
| 34 | Maturity onset diabetes of the young | 4950 | 23 | 16 | 0.082684 | -7.52432 | 0.027 | 0.015861 | 0.06391 | 1 |
| 35 | Influenza A | 5164 | 160 | 92 | 0.146221 | 16.81476 | 0.016 | 0.016512 | 0.064633 | 1 |
| 36 | Notch signaling pathway | 4330 | 47 | 33 | 0.012474 | -8.86058 | 0.204 | 0.017746 | 0.067534 | 1 |
| 37 | Shigellosis | 5131 | 60 | 40 | 0.022576 | 8.765468 | 0.168 | 0.024936 | 0.091553 | 1 |
| 38 | Protein processing in endoplasmic reticulum | 4141 | 162 | 101 | 0.010361 | -3.40562 | 0.374 | 0.025394 | 0.091553 | 1 |
| 39 | Transcriptional misregulation in cancer | 5202 | 158 | 98 | 0.013912 | -0.50654 | 0.307 | 0.027573 | 0.096859 | 1 |
| 40 | Melanoma | 5218 | 71 | 43 | 0.124519 | -26.5986 | 0.042 | 0.032704 | 0.112011 | 1 |
| 41 | MAPK signaling pathway | 4010 | 259 | 157 | 0.008137 | 3.21107 | 0.68 | 0.034287 | 0.11457 | 1 |
| 42 | TGF-beta signaling pathway | 4350 | 81 | 53 | 0.016107 | 5.402742 | 0.431 | 0.041446 | 0.135193 | 1 |
| 43 | Pancreatic cancer | 5212 | 69 | 46 | 0.015075 | 4.715968 | 0.485 | 0.043271 | 0.137863 | 1 |
| 44 | Chronic myeloid leukemia | 5220 | 72 | 41 | 0.293671 | 14.3163 | 0.034 | 0.055982 | 0.174307 | 1 |
| 45 | Fc gamma R-mediated phagocytosis | 4666 | 91 | 56 | 0.063583 | 10.9975 | 0.205 | 0.069607 | 0.207356 | 1 |
| 46 | Basal cell carcinoma | 5217 | 54 | 37 | 0.015126 | 1.63362 | 0.862 | 0.069623 | 0.207356 | 1 |
| 47 | HTLV-I infection | 5166 | 256 | 149 | 0.054575 | 9.61187 | 0.303 | 0.084371 | 0.245932 | 1 |
| 48 | Renal cell carcinoma | 5211 | 69 | 39 | 0.324401 | 10.29844 | 0.055 | 0.089678 | 0.253449 | 1 |
| 49 | Morphine addiction | 5032 | 89 | 49 | 0.393128 | -5.95905 | 0.046 | 0.09065 | 0.253449 | 1 |
| 50 | VEGF signa | 4370 | 71 | 44 | 0.081736 | 8.2938 | 0.227 | 0.092531 | 0.253534 | 1 |
| 51 | Gap junction | 4540 | 85 | 51 | 0.119239 | -11.345 | 0.16 | 0.094613 | 0.254156 | 1 |
| 52 | ErbB signaling pathway | 4012 | 87 | 51 | 0.174862 | 20.09637 | 0.121 | 0.102739 | 0.268139 | 1 |
| 53 | Viral carcinogenesis | 5203 | 182 | 109 | 0.036925 | 0.46347 | 0.58 | 0.103733 | 0.268139 | 1 |
| 54 | Amoebiasis | 5146 | 106 | 64 | 0.07767 | -3.53846 | 0.304 | 0.112061 | 0.279863 | 1 |
| 55 | Epithelial cell signaling in Helicobacter pylori infection | 5120 | 67 | 40 | 0.166097 | 7.490237 | 0.143 | 0.112586 | 0.279863 | 1 |
| 56 | Melanogenesis | 4916 | 99 | 60 | 0.078949 | 18.92963 | 0.307 | 0.114397 | 0.279863 | 1 |
| 57 | Cytokine-cytokine receptor interaction | 4060 | 251 | 125 | 0.86472 | 13.01841 | 0.03 | 0.120678 | 0.29005 | 1 |
| 58 | Non-small cell lung cancer | 5223 | 54 | 35 | 0.054113 | 6.576154 | 0.531 | 0.130729 | 0.308792 | 1 |
| 59 | Rheumatoid arthritis | 5323 | 83 | 46 | 0.374057 | 3.111136 | 0.079 | 0.133617 | 0.310263 | 1 |
| 60 | Hedgehog signaling pathway | 4340 | 54 | 34 | 0.091999 | -4.57046 | 0.368 | 0.148479 | 0.331308 | 1 |
| 61 | Aldosterone-regulated sodium reabsorption | 4960 | 39 | 26 | 0.060077 | -1.44331 | 0.578 | 0.151409 | 0.331308 | 1 |
| 62 | Chemokine signaling pathway | 4062 | 179 | 88 | 0.86912 | 26.47737 | 0.041 | 0.154454 | 0.331308 | 1 |
| 63 | RNA degradation | 3018 | 69 | 44 | 0.04706 | -0.41734 | 0.767 | 0.155987 | 0.331308 | 1 |
| 64 | Alzheimer's disease | 5010 | 159 | 85 | 0.491187 | 7.892265 | 0.074 | 0.156827 | 0.331308 | 1 |
| 65 | Vibrio cholerae infection | 5110 | 53 | 34 | 0.068144 | 1.032058 | 0.535 | 0.15719 | 0.331308 | 1 |
| 66 | Long-term depression | 4730 | 65 | 42 | 0.039486 | 0.140842 | 0.982 | 0.164794 | 0.340651 | 1 |
| 67 | Lysosome | 4142 | 117 | 72 | 0.039331 | 0 | 1 | 0.166596 | 0.340651 | 1 |
| 68 | NOD-like receptor signaling pathway | 4621 | 57 | 30 | 0.578783 | 8.632065 | 0.075 | 0.179586 | 0.361813 | 1 |
| 69 | Phototransduction | 4744 | 28 | 18 | 0.158072 | 4.033036 | 0.309 | 0.196311 | 0.389777 | 1 |
| 70 | Amphetamine addiction | 5031 | 69 | 38 | 0.415434 | -6.04319 | 0.121 | 0.200587 | 0.392578 | 1 |
| 71 | Bladder cancer | 5219 | 41 | 26 | 0.119707 | 4.036406 | 0.456 | 0.213322 | 0.41162 | 1 |
| 72 | Circadian rhythm | 4710 | 21 | 14 | 0.15094 | -2.46465 | 0.394 | 0.227312 | 0.427292 | 1 |
| 73 | Glutamatergic synapse | 4724 | 119 | 72 | 0.061067 | 0.091575 | 0.976 | 0.227681 | 0.427292 | 1 |
| 74 | Carbohydrate digestion and absorption | 4973 | 38 | 23 | 0.223829 | -1.4447 | 0.279 | 0.235643 | 0.430886 | 1 |
| 75 | Oocyte meiosis | 4114 | 109 | 61 | 0.304008 | 11.11115 | 0.215 | 0.243657 | 0.430886 | 1 |
| 76 | Prostate cancer | 5215 | 89 | 54 | 0.09005 | 3.38478 | 0.729 | 0.244434 | 0.430886 | 1 |
| 77 | GnRH signaling pathway | 4912 | 94 | 50 | 0.531196 | 20.31963 | 0.124 | 0.245036 | 0.430886 | 1 |
| 78 | Fanconi anemia pathway | 3460 | 48 | 25 | 0.610864 | 2.805947 | 0.108 | 0.245322 | 0.430886 | 1 |
| 79 | Toll-like receptor signaling pathway | 4620 | 98 | 36 | 0.999602 | 12.86373 | 0.072 | 0.261363 | 0.446515 | 1 |
| 80 | RIG-I-like receptor signaling pathway | 4622 | 70 | 36 | 0.653252 | 6.799448 | 0.111 | 0.262781 | 0.446515 | 1 |
| 81 | Pertussis | 5133 | 69 | 35 | 0.695003 | 9.206661 | 0.105 | 0.263998 | 0.446515 | 1 |
| 82 | Vascular smooth muscle contraction | 4270 | 110 | 65 | 0.11939 | -4.15685 | 0.625 | 0.268281 | 0.448225 | 1 |
| 83 | Adipocytokine signaling pathway | 4920 | 68 | 40 | 0.202536 | 3.162989 | 0.393 | 0.281039 | 0.456956 | 1 |
| 84 | Endometrial cancer | 5213 | 52 | 31 | 0.209224 | 6.843705 | 0.381 | 0.281336 | 0.456956 | 1 |
| 85 | RNA transport | 3013 | 145 | 85 | 0.102645 | -0.31044 | 0.785 | 0.283513 | 0.456956 | 1 |
| 86 | Serotonergic synapse | 4726 | 114 | 66 | 0.172384 | -3.15033 | 0.481 | 0.289373 | 0.460977 | 1 |
| 87 | Bile secretion | 4976 | 71 | 40 | 0.33199 | -1.64165 | 0.262 | 0.299396 | 0.471462 | 1 |
| 88 | Cytosolic DNA-sensing pathway | 4623 | 59 | 26 | 0.935062 | 3.373188 | 0.095 | 0.303892 | 0.473105 | 1 |
| 89 | Dopaminergic synapse | 4728 | 128 | 70 | 0.389174 | -3.94217 | 0.258 | 0.331194 | 0.509816 | 1 |
| 90 | Hepatitis C | 5160 | 128 | 74 | 0.160125 | 2.794889 | 0.642 | 0.336667 | 0.512483 | 1 |
| 91 | Toxoplasmosis | 5145 | 120 | 68 | 0.241014 | 5.123155 | 0.434 | 0.340746 | 0.512992 | 1 |
| 92 | Glioma | 5214 | 64 | 37 | 0.262067 | -6.67344 | 0.451 | 0.370585 | 0.55185 | 1 |
| 93 | PPAR signaling pathway | 3320 | 69 | 40 | 0.242668 | 0.80924 | 0.518 | 0.386388 | 0.560782 | 1 |
| 94 | Asthma | 5310 | 26 | 6 | 0.999569 | -0.32207 | 0.126 | 0.386893 | 0.560782 | 1 |
| 95 | Epstein-Barr virus infection | 5169 | 189 | 108 | 0.144696 | 0.935781 | 0.877 | 0.388863 | 0.560782 | 1 |
| 96 | B cell receptor signaling pathway | 4662 | 75 | 41 | 0.435464 | 7.94357 | 0.298 | 0.394756 | 0.56335 | 1 |
| 97 | Dorso-ventral axis formation | 4320 | 23 | 15 | 0.168546 | 0.151153 | 0.812 | 0.409045 | 0.577723 | 1 |
| 98 | NF-kappa B signaling pathway | 4064 | 88 | 45 | 0.680499 | 8.510468 | 0.211 | 0.422259 | 0.590301 | 1 |
| 99 | T cell receptor signaling pathway | 4660 | 108 | 59 | 0.408703 | 7.270372 | 0.374 | 0.439957 | 0.608829 | 1 |
| 100 | Insulin signaling pathway | 4910 | 136 | 78 | 0.178248 | -2.68918 | 0.879 | 0.447094 | 0.612519 | 1 |
| 101 | Type I diabetes mellitus | 4940 | 39 | 20 | 0.64961 | 1.151505 | 0.246 | 0.452854 | 0.614267 | 1 |
| 102 | African trypanosomiasis | 5143 | 32 | 16 | 0.700203 | 1.855489 | 0.238 | 0.46526 | 0.621381 | 1 |
| 103 | Long-term potentiation | 4720 | 69 | 39 | 0.324401 | -7.19709 | 0.517 | 0.467169 | 0.621381 | 1 |
| 104 | Graft-versus-host disease | 5332 | 34 | 16 | 0.808659 | 1.151505 | 0.214 | 0.476615 | 0.627849 | 1 |
| 105 | Osteoclast differentiation | 4380 | 129 | 69 | 0.496199 | -6.92393 | 0.358 | 0.4846 | 0.632287 | 1 |
| 106 | Measles | 5162 | 128 | 69 | 0.458672 | 5.258723 | 0.411 | 0.503065 | 0.650188 | 1 |
| 107 | Natural killer cell mediated cytotoxicity | 4650 | 127 | 68 | 0.49183 | 16.50551 | 0.409 | 0.523749 | 0.670594 | 1 |
| 108 | Intestinal immune network for IgA production | 4672 | 44 | 21 | 0.805092 | 1.518606 | 0.268 | 0.546654 | 0.693441 | 1 |
| 109 | Type II diabetes mellitus | 4930 | 47 | 27 | 0.32449 | -1.61639 | 0.712 | 0.569547 | 0.71061 | 1 |
| 110 | Retrograde endocannabinoid signaling | 4723 | 100 | 52 | 0.622933 | -1.01812 | 0.372 | 0.570563 | 0.71061 | 1 |
| 111 | SNARE interactions in vesicular transport | 4130 | 35 | 16 | 0.850796 | 1.634397 | 0.282 | 0.5824 | 0.716241 | 1 |
| 112 | Systemic lupus erythematosus | 5322 | 81 | 38 | 0.888788 | 2.435842 | 0.273 | 0.58626 | 0.716241 | 1 |
| 113 | Fc epsilon RI signaling pathway | 4664 | 73 | 35 | 0.839697 | 9.331601 | 0.295 | 0.593389 | 0.716241 | 1 |
| 114 | Neurotrophin signaling pathway | 4722 | 119 | 66 | 0.331894 | 2.674077 | 0.752 | 0.595996 | 0.716241 | 1 |
| 115 | Taste transduction | 4742 | 45 | 18 | 0.971739 | -4.20362 | 0.261 | 0.601571 | 0.716655 | 1 |
| 116 | Chagas disease (American trypanosomiasis) | 5142 | 101 | 51 | 0.731354 | 6.6696 | 0.38 | 0.633768 | 0.748502 | 1 |
| 117 | GABAergic synapse | 4727 | 87 | 49 | 0.307499 | -0.09779 | 0.955 | 0.653493 | 0.765201 | 1 |
| 118 | Huntington's disease | 5016 | 171 | 89 | 0.633998 | 1.542757 | 0.495 | 0.677528 | 0.786621 | 1 |
| 119 | Allograft rejection | 5330 | 33 | 15 | 0.853032 | 0.829431 | 0.374 | 0.683517 | 0.786906 | 1 |
| 120 | Jak-STAT signaling pathway | 4630 | 153 | 80 | 0.607445 | -1.67714 | 0.538 | 0.692302 | 0.790378 | 1 |
| 121 | Legionellosis | 5134 | 51 | 26 | 0.669631 | 2.38647 | 0.512 | 0.709859 | 0.803725 | 1 |
| 122 | Vasopressin-regulated water reabsorption | 4962 | 44 | 23 | 0.601493 | -1.20805 | 0.596 | 0.726248 | 0.815541 | 1 |
| 123 | Autoimmune thyroid disease | 5320 | 47 | 20 | 0.944061 | 0.829431 | 0.393 | 0.738882 | 0.822982 | 1 |
| 124 | Sulfur relay system | 4122 | 9 | 5 | 0.574731 | 0.489073 | 0.674 | 0.754741 | 0.833867 | 1 |
| 125 | Cholinergic synapse | 4725 | 109 | 54 | 0.797543 | -4.98192 | 0.52 | 0.779739 | 0.854594 | 1 |
| 126 | Parkinson's disease | 5012 | 116 | 53 | 0.954036 | 2.883577 | 0.514 | 0.839808 | 0.907658 | 1 |
| 127 | Phosphatid | 4070 | 79 | 42 | 0.537798 | 0.081805 | 0.916 | 0.841405 | 0.907658 | 1 |
| 128 | Leishmaniasis | 5140 | 66 | 32 | 0.807417 | 1.998346 | 0.65 | 0.863172 | 0.918531 | 1 |
| 129 | Cocaine addiction | 5030 | 50 | 25 | 0.71769 | -1.04364 | 0.735 | 0.864894 | 0.918531 | 1 |
| 130 | Acute myeloid leukemia | 5221 | 56 | 26 | 0.870378 | 1.595394 | 0.68 | 0.90228 | 0.950865 | 1 |
| 131 | Progesterone-mediated oocyte maturation | 4914 | 84 | 42 | 0.749178 | 0.837008 | 0.84 | 0.920763 | 0.962747 | 1 |
| 132 | Regulation of autophagy | 4140 | 33 | 11 | 0.993046 | 1.318823 | 0.649 | 0.927611 | 0.962747 | 1 |
| 133 | mTOR signaling pathway | 4150 | 62 | 28 | 0.915201 | 0.566332 | 0.758 | 0.947405 | 0.975899 | 1 |
| 134 | Malaria | 5144 | 47 | 22 | 0.84251 | 0 | 1 | 0.986891 | 0.998178 | 1 |
| 135 | Prion diseases | 5020 | 34 | 15 | 0.888063 | -0.14043 | 0.97 | 0.989921 | 0.998178 | 1 |
| 136 | Alcoholism | 5034 | 128 | 59 | 0.952523 | -0.79394 | 0.935 | 0.993786 | 0.998178 | 1 |
| 137 | Staphylococcus aureus infection | 5150 | 48 | 16 | 0.998141 | -0.30594 | 0.942 | 0.998178 | 0.998178 | 1 |

Result obtained by the BPA methods in the pancreatic cancer dataset

| No | ID and Name | Score | p-value | FDR | FWER |
| --- | --- | --- | --- | --- | --- |
| 1 | hsa05222 Small cell lung cancer - Homo sapiens (human) | -12114.1 | 0.001 | 0.078647 | 0.382199 |
| 2 | hsa05211 Renal cell carcinoma - Homo sapiens (human) | -13171.8 | 0.003 | 0.078647 | 0.319372 |
| 3 | hsa04110 Cell cycle - Homo sapiens (human) | -7125.17 | 0.005 | 0.078647 | 0.34555 |
| 4 | hsa04115 p53 signaling pathway - Homo sapiens (human) | -19057.5 | 0.005 | 0.078647 | 0.518325 |
| 5 | hsa04512 ECM-receptor interaction - Homo sapiens (human) | -15255.9 | 0.005 | 0.078647 | 0.293194 |
| 6 | hsa04070 Phosphatidylinositol signaling system - Homo sapiens (human) | -7368.28 | 0.006 | 0.078647 | 0.193717 |
| 7 | hsa05221 Acute myeloid leukemia - Homo sapiens (human) | -11635.3 | 0.006 | 0.078647 | 0.314136 |
| 8 | hsa04370 VEGF signaling pathway - Homo sapiens (human) | -9510.56 | 0.007 | 0.078647 | 0.21466 |
| 9 | hsa04510 Focal adhesion - Homo sapiens (human) | -21675.6 | 0.007 | 0.078647 | 0.397906 |
| 10 | hsa04520 Adherens junction - Homo sapiens (human) | -16897.7 | 0.007 | 0.078647 | 0.387435 |
| 11 | hsa05120 Epithelial cell signaling in Helicobacter pylori infection - Homo sapiens (human) | -12059.4 | 0.008 | 0.084889 | 0.518325 |
| 12 | hsa04330 Notch signaling pathway - Homo sapiens (human) | -8355.02 | 0.009 | 0.08595 | 0.848168 |
| 13 | hsa05220 Chronic myeloid leukemia - Homo sapiens (human) | -14367.2 | 0.009 | 0.08595 | 0.769634 |
| 14 | hsa00983 Drug metabolism - other enzymes - Homo sapiens (human) | -6557.02 | 0.012 | 0.089531 | 0.219895 |
| 15 | hsa04310 Wnt signaling pathway - Homo sapiens (human) | -23040.4 | 0.012 | 0.089531 | 0.575916 |
| 16 | hsa04360 Axon guidance - Homo sapiens (human) | -21926.5 | 0.012 | 0.089531 | 0.60733 |
| 17 | hsa00230 Purine metabolism - Homo sapiens (human) | -14801.3 | 0.013 | 0.089531 | 0.198953 |
| 18 | hsa00410 beta-Alanine metabolism - Homo sapiens (human) | -4109.65 | 0.013 | 0.089531 | 0.26178 |
| 19 | hsa00512 O-Glycan biosynthesis - Homo sapiens (human) | -2401.59 | 0.013 | 0.089531 | 0.020942 |
| 20 | hsa04210 Apoptosis - Homo sapiens (human) | -17428.8 | 0.013 | 0.089531 | 0.591623 |
| 21 | hsa00750 Vitamin B6 metabolism - Homo sapiens (human) | -1337.1 | 0.014 | 0.089531 | 0.020942 |
| 22 | hsa00010 Glycolysis / Gluconeogenesis - Homo sapiens (human) | -7664.7 | 0.015 | 0.089531 | 0.371728 |
| 23 | hsa00232 Caffeine metabolism - Homo sapiens (human) | -930.205 | 0.015 | 0.089531 | 0.193717 |
| 24 | hsa00601 Glycosphingolipid biosynthesis - lacto and neolacto series - Homo sapiens (human) | -5899.56 | 0.015 | 0.089531 | 0.193717 |
| 25 | hsa04610 Complement and coagulation cascades - Homo sapiens (human) | -19430.2 | 0.015 | 0.089531 | 0.518325 |
| 26 | hsa00562 Inositol phosphate metabolism - Homo sapiens (human) | -7153.79 | 0.016 | 0.092606 | 0.424084 |
| 27 | hsa04720 Long-term potentiation - Homo sapiens (human) | -8114.14 | 0.017 | 0.092919 | 0.382199 |
| 28 | hsa00220 Urea cycle and metabolism of amino groups - Homo sapiens (human) | -5913.59 | 0.018 | 0.092919 | 0.099476 |
| 29 | hsa00252 Alanine and aspartate metabolism - Homo sapiens (human) | -6127.54 | 0.018 | 0.092919 | 0.099476 |
| 30 | hsa04530 Tight junction - Homo sapiens (human) | -19127.3 | 0.018 | 0.092919 | 0.612565 |
| 31 | hsa00361 gamma-Hexachlorocyclohexane degradation - Homo sapiens (human) | -1784.52 | 0.02 | 0.097532 | 0.293194 |
| 32 | hsa05210 Colorectal cancer - Homo sapiens (human) | -15349.2 | 0.021 | 0.097532 | 0.65445 |
| 33 | hsa03410 Base excision repair - Homo sapiens (human) | -9641.43 | 0.022 | 0.097532 | 1 |
| 34 | hsa00071 Fatty acid metabolism - Homo sapiens (human) | -6052.06 | 0.023 | 0.097532 | 0.193717 |
| 35 | hsa00480 Glutathione metabolism - Homo sapiens (human) | -5947 | 0.023 | 0.097532 | 0.26178 |
| 36 | hsa00670 One carbon pool by folate - Homo sapiens (human) | -5031.6 | 0.023 | 0.097532 | 0.246073 |
| 37 | hsa04910 Insulin signaling pathway - Homo sapiens (human) | -22928.7 | 0.023 | 0.097532 | 0.612565 |
| 38 | hsa05216 Thyroid cancer - Homo sapiens (human) | -5398.61 | 0.023 | 0.097532 | 0.596859 |
| 39 | hsa00272 Cysteine metabolism - Homo sapiens (human) | -2613.52 | 0.024 | 0.097532 | 0.575916 |
| 40 | hsa05014 Amyotrophic lateral sclerosis (ALS) - Homo sapiens (human) | -12725.4 | 0.024 | 0.097532 | 0.643979 |
| 41 | hsa04514 Cell adhesion molecules (CAMs) - Homo sapiens (human) | -29042.1 | 0.025 | 0.099479 | 0.628272 |
| 42 | hsa00510 N-Glycan biosynthesis - Homo sapiens (human) | -11702.5 | 0.026 | 0.100906 | 0.780105 |
| 43 | hsa00260 Glycine, serine and threonine metabolism - Homo sapiens (human) | -10813.7 | 0.028 | 0.100906 | 0.65445 |
| 44 | hsa00620 Pyruvate metabolism - Homo sapiens (human) | -7424.62 | 0.028 | 0.100906 | 0.575916 |
| 45 | hsa04630 Jak-STAT signaling pathway - Homo sapiens (human) | -10168.5 | 0.028 | 0.100906 | 0.712042 |
| 46 | hsa04650 Natural killer cell mediated cytotoxicity - Homo sapiens (human) | -22334.9 | 0.028 | 0.100906 | 0.539267 |
| 47 | hsa00340 Histidine metabolism - Homo sapiens (human) | -5380.95 | 0.029 | 0.102574 | 0.769634 |
| 48 | hsa04710 Circadian rhythm - Homo sapiens (human) | -2462.87 | 0.031 | 0.107655 | 0.293194 |
| 49 | hsa00330 Arginine and proline metabolism - Homo sapiens (human) | -6885.47 | 0.032 | 0.108233 | 0.366492 |
| 50 | hsa00052 Galactose metabolism - Homo sapiens (human) | -4846.13 | 0.033 | 0.108233 | 0.397906 |
| 51 | hsa00563 Glycosylphosphatidylinositol(GPI)-anchor biosynthesis - Homo sapiens (human) | -8839.16 | 0.034 | 0.108233 | 0.581152 |
| 52 | hsa05010 Alzheimer's disease - Homo sapiens (human) | -20054.1 | 0.034 | 0.108233 | 0.811518 |
| 53 | hsa05332 Graft-versus-host disease - Homo sapiens (human) | -5609.61 | 0.034 | 0.108233 | 0.91623 |
| 54 | hsa00531 Glycosaminoglycan degradation - Homo sapiens (human) | -4854.77 | 0.036 | 0.112721 | 0.319372 |
| 55 | hsa05223 Non-small cell lung cancer - Homo sapiens (human) | -10993.5 | 0.037 | 0.113984 | 0.361257 |
| 56 | hsa00520 Nucleotide sugars metabolism - Homo sapiens (human) | -1898.23 | 0.04 | 0.12127 | 0.481675 |
| 57 | hsa05330 Allograft rejection - Homo sapiens (human) | -6731.57 | 0.041 | 0.122359 | 0.832461 |
| 58 | hsa04010 MAPK signaling pathway - Homo sapiens (human) | -44085.5 | 0.042 | 0.123415 | 0.759162 |
| 59 | hsa00770 Pantothenate and CoA biosynthesis - Homo sapiens (human) | -2999.83 | 0.045 | 0.128284 | 0.246073 |
| 60 | hsa04742 Taste transduction - Homo sapiens (human) | -6409.21 | 0.045 | 0.128284 | 0.764398 |
| 61 | hsa00604 Glycosphingolipid biosynthesis - ganglio series - Homo sapiens (human) | -4321.15 | 0.046 | 0.129206 | 0.251309 |
| 62 | hsa04540 Gap junction - Homo sapiens (human) | -12856.3 | 0.047 | 0.130101 | 0.596859 |
| 63 | hsa00281 Geraniol degradation - Homo sapiens (human) | -801.993 | 0.049 | 0.131817 | 0.958115 |
| 64 | hsa04670 Leukocyte transendothelial migration - Homo sapiens (human) | -7824.41 | 0.049 | 0.131817 | 0.575916 |
| 65 | hsa00830 Retinol metabolism - Homo sapiens (human) | -4757.65 | 0.051 | 0.135292 | 0.277487 |
| 66 | hsa04660 T cell receptor signaling pathway - Homo sapiens (human) | -20075.1 | 0.053 | 0.138671 | 0.748691 |
| 67 | hsa04020 Calcium signaling pathway - Homo sapiens (human) | -14128.1 | 0.054 | 0.139378 | 0.712042 |
| 68 | hsa00130 Ubiquinone and menaquinone biosynthesis - Homo sapiens (human) | -1996.5 | 0.057 | 0.14387 | 0.811518 |
| 69 | hsa00280 Valine, leucine and isoleucine degradation - Homo sapiens (human) | -9131.6 | 0.058 | 0.14387 | 0.21466 |
| 70 | hsa00561 Glycerolipid metabolism - Homo sapiens (human) | -6350.49 | 0.058 | 0.14387 | 0.623037 |
| 71 | hsa05215 Prostate cancer - Homo sapiens (human) | -15522.5 | 0.06 | 0.146198 | 0.795812 |
| 72 | hsa00020 Citrate cycle (TCA cycle) - Homo sapiens (human) | -6068.24 | 0.062 | 0.146198 | 0.612565 |
| 73 | hsa00400 Phenylalanine, tyrosine and tryptophan biosynthesis - Homo sapiens (human) | -1892.36 | 0.062 | 0.146198 | 0.554974 |
| 74 | hsa00920 Sulfur metabolism - Homo sapiens (human) | -2662.6 | 0.062 | 0.146198 | 0.65445 |
| 75 | hsa00251 Glutamate metabolism - Homo sapiens (human) | -5500.83 | 0.066 | 0.153732 | 0.319372 |
| 76 | hsa05060 Prion disease - Homo sapiens (human) | -4016.64 | 0.068 | 0.156482 | 1 |
| 77 | hsa00970 Aminoacyl-tRNA biosynthesis - Homo sapiens (human) | -9201.24 | 0.07 | 0.158443 | 1 |
| 78 | hsa00300 Lysine biosynthesis - Homo sapiens (human) | -1488.59 | 0.072 | 0.158443 | 0.518325 |
| 79 | hsa00072 Synthesis and degradation of ketone bodies - Homo sapiens (human) | -1704.42 | 0.073 | 0.158443 | 0.445026 |
| 80 | hsa00650 Butanoate metabolism - Homo sapiens (human) | -5221.84 | 0.073 | 0.158443 | 1 |
| 81 | hsa04662 B cell receptor signaling pathway - Homo sapiens (human) | -12823.9 | 0.073 | 0.158443 | 0.759162 |
| 82 | hsa05213 Endometrial cancer - Homo sapiens (human) | -10784.2 | 0.075 | 0.160955 | 0.570681 |
| 83 | hsa00640 Propanoate metabolism - Homo sapiens (human) | -6221.74 | 0.078 | 0.163714 | 0.790576 |
| 84 | hsa05217 Basal cell carcinoma - Homo sapiens (human) | -6681.63 | 0.078 | 0.163714 | 0.848168 |
| 85 | hsa00630 Glyoxylate and dicarboxylate metabolism - Homo sapiens (human) | -3989.72 | 0.08 | 0.166087 | 0.884817 |
| 86 | hsa00271 Methionine metabolism - Homo sapiens (human) | -4025.62 | 0.082 | 0.168409 | 0.21466 |
| 87 | hsa04130 SNARE interactions in vesicular transport - Homo sapiens (human) | -9382.12 | 0.084 | 0.170681 | 0.801047 |
| 88 | hsa00450 Selenoamino acid metabolism - Homo sapiens (human) | -2410.43 | 0.086 | 0.171104 | 1 |
| 89 | hsa04810 Regulation of actin cytoskeleton - Homo sapiens (human) | -14466.8 | 0.086 | 0.171104 | 1 |
| 90 | hsa00532 Chondroitin sulfate biosynthesis - Homo sapiens (human) | -4691.36 | 0.092 | 0.181155 | 0.832461 |
| 91 | hsa05212 Pancreatic cancer - Homo sapiens (human) | -14323.8 | 0.095 | 0.185153 | 0.670157 |
| 92 | hsa05320 Autoimmune thyroid disease - Homo sapiens (human) | -1169.93 | 0.097 | 0.187141 | 0.670157 |
| 93 | hsa00980 Metabolism of xenobiotics by cytochrome P450 - Homo sapiens (human) | -2615.39 | 0.099 | 0.18909 | 0.591623 |
| 94 | hsa04620 Toll-like receptor signaling pathway - Homo sapiens (human) | -22783.4 | 0.102 | 0.191 | 0.884817 |
| 95 | hsa04912 GnRH signaling pathway - Homo sapiens (human) | -15172.9 | 0.102 | 0.191 | 0.769634 |
| 96 | hsa04612 Antigen processing and presentation - Homo sapiens (human) | -9092.51 | 0.104 | 0.191 | 0.748691 |
| 97 | hsa05214 Glioma - Homo sapiens (human) | -11282.3 | 0.104 | 0.191 | 0.696335 |
| 98 | hsa00140 C21-Steroid hormone metabolism - Homo sapiens (human) | -1462.12 | 0.109 | 0.198208 | 0.277487 |
| 99 | hsa04080 Neuroactive ligand-receptor interaction - Homo sapiens (human) | -33631.1 | 0.11 | 0.198208 | 1 |
| 100 | hsa00910 Nitrogen metabolism - Homo sapiens (human) | -3449.73 | 0.112 | 0.199925 | 0.47644 |
| 101 | hsa00061 Fatty acid biosynthesis - Homo sapiens (human) | -1912.97 | 0.116 | 0.205148 | 0.743455 |
| 102 | hsa04350 TGF-beta signaling pathway - Homo sapiens (human) | -15634.2 | 0.118 | 0.206771 | 0.811518 |
| 103 | hsa00565 Ether lipid metabolism - Homo sapiens (human) | -3604.19 | 0.121 | 0.2101 | 0.884817 |
| 104 | hsa05310 Asthma - Homo sapiens (human) | -1171.25 | 0.123 | 0.211283 | 0.712042 |
| 105 | hsa00530 Aminosugars metabolism - Homo sapiens (human) | -3616.73 | 0.125 | 0.211283 | 1 |
| 106 | hsa05219 Bladder cancer - Homo sapiens (human) | -9500.12 | 0.125 | 0.211283 | 0.780105 |
| 107 | hsa00641 3-Chloroacrylic acid degradation - Homo sapiens (human) | -407.728 | 0.135 | 0.225878 | 1 |
| 108 | hsa04060 Cytokine-cytokine receptor interaction - Homo sapiens (human) | -40994.8 | 0.136 | 0.225878 | 1 |
| 109 | hsa04740 Olfactory transduction - Homo sapiens (human) | -4396.4 | 0.142 | 0.23381 | 0.643979 |
| 110 | hsa00460 Cyanoamino acid metabolism - Homo sapiens (human) | -1607.23 | 0.155 | 0.250387 | 1 |
| 111 | hsa05110 Vibrio cholerae infection - Homo sapiens (human) | -6888.5 | 0.155 | 0.250387 | 0.86911 |
| 112 | hsa03010 Ribosome - Homo sapiens (human) | -28543.6 | 0.156 | 0.250387 | 1 |
| 113 | hsa03320 PPAR signaling pathway - Homo sapiens (human) | -13671.7 | 0.161 | 0.256258 | 0.486911 |
| 114 | hsa00720 Reductive carboxylate cycle (CO2 fixation) - Homo sapiens (human) | -2336.48 | 0.163 | 0.257298 | 0.780105 |
| 115 | hsa00430 Taurine and hypotaurine metabolism - Homo sapiens (human) | -2273.07 | 0.172 | 0.269279 | 0.759162 |
| 116 | hsa00860 Porphyrin and chlorophyll metabolism - Homo sapiens (human) | -6135.53 | 0.192 | 0.298146 | 0.780105 |
| 117 | hsa00310 Lysine degradation - Homo sapiens (human) | -2009.77 | 0.194 | 0.298823 | 1 |
| 118 | hsa00290 Valine, leucine and isoleucine biosynthesis - Homo sapiens (human) | -1923.73 | 0.201 | 0.30469 | 0.780105 |
| 119 | hsa03420 Nucleotide excision repair - Homo sapiens (human) | -10855.7 | 0.201 | 0.30469 | 1 |
| 120 | hsa04150 mTOR signaling pathway - Homo sapiens (human) | -10309.7 | 0.209 | 0.313359 | 0.837696 |
| 121 | hsa00150 Androgen and estrogen metabolism - Homo sapiens (human) | -803.579 | 0.21 | 0.313359 | 1 |
| 122 | hsa05012 Parkinson's disease - Homo sapiens (human) | -10022.9 | 0.212 | 0.313891 | 1 |
| 123 | hsa04012 ErbB signaling pathway - Homo sapiens (human) | -16897.5 | 0.218 | 0.320292 | 0.801047 |
| 124 | hsa00051 Fructose and mannose metabolism - Homo sapiens (human) | -1533.41 | 0.224 | 0.324556 | 0.848168 |
| 125 | hsa00533 Keratan sulfate biosynthesis - Homo sapiens (human) | -4020.11 | 0.225 | 0.324556 | 1 |
| 126 | hsa00062 Fatty acid elongation in mitochondria - Homo sapiens (human) | -2024.51 | 0.226 | 0.324556 | 0.78534 |
| 127 | hsa04664 Fc epsilon RI signaling pathway - Homo sapiens (human) | -13439.5 | 0.231 | 0.329261 | 0.86911 |
| 128 | hsa00534 Heparan sulfate biosynthesis - Homo sapiens (human) | -5589.71 | 0.239 | 0.338141 | 1 |
| 129 | hsa00591 Linoleic acid metabolism - Homo sapiens (human) | -803.996 | 0.245 | 0.341569 | 1 |
| 130 | hsa02010 ABC transporters - General - Homo sapiens (human) | -17288.5 | 0.245 | 0.341569 | 1 |
| 131 | hsa00190 Oxidative phosphorylation - Homo sapiens (human) | -34134.5 | 0.255 | 0.352935 | 1 |
| 132 | hsa04916 Melanogenesis - Homo sapiens (human) | -12007.8 | 0.259 | 0.355892 | 0.926702 |
| 133 | hsa00960 Alkaloid biosynthesis II - Homo sapiens (human) | -1205.94 | 0.281 | 0.383364 | 1 |
| 134 | hsa00603 Glycosphingolipid biosynthesis - globo series - Homo sapiens (human) | -4230.76 | 0.304 | 0.411801 | 0.973822 |
| 135 | hsa00900 Terpenoid biosynthesis - Homo sapiens (human) | -1598.97 | 0.31 | 0.416972 | 1 |
| 136 | hsa00730 Thiamine metabolism - Homo sapiens (human) | -1206.68 | 0.389 | 0.519573 | 1 |
| 137 | hsa00380 Tryptophan metabolism - Homo sapiens (human) | -1608.94 | 0.432 | 0.573 | 1 |
| 138 | hsa00550 Peptidoglycan biosynthesis - Homo sapiens (human) | -804.795 | 0.443 | 0.582034 | 1 |
| 139 | hsa00680 Methane metabolism - Homo sapiens (human) | -2013.01 | 0.445 | 0.582034 | 1 |
| 140 | hsa05218 Melanoma - Homo sapiens (human) | -8667.09 | 0.45 | 0.582034 | 1 |
| 141 | hsa05050 Dentatorubropallidoluysian atrophy (DRPLA) - Homo sapiens (human) | -2815.45 | 0.451 | 0.582034 | 1 |
| 142 | hsa04614 Renin-angiotensin system - Homo sapiens (human) | -6435.91 | 0.484 | 0.62043 | 1 |
| 143 | hsa05040 Huntington's disease - Homo sapiens (human) | -8045.3 | 0.507 | 0.64558 | 1 |
| 144 | hsa00120 Bile acid biosynthesis - Homo sapiens (human) | -1609.2 | 0.511 | 0.646364 | 1 |
| 145 | hsa00790 Folate biosynthesis - Homo sapiens (human) | -1609.3 | 0.542 | 0.679111 | 1 |
| 146 | hsa00564 Glycerophospholipid metabolism - Homo sapiens (human) | -10459.3 | 0.544 | 0.679111 | 1 |
| 147 | hsa04920 Adipocytokine signaling pathway - Homo sapiens (human) | -12532.4 | 0.561 | 0.695786 | 1 |
| 148 | hsa00030 Pentose phosphate pathway - Homo sapiens (human) | -5623.01 | 0.574 | 0.704006 | 1 |
| 149 | hsa00785 Lipoic acid metabolism - Homo sapiens (human) | -408.685 | 0.575 | 0.704006 | 1 |
| 150 | hsa00950 Alkaloid biosynthesis I - Homo sapiens (human) | -1323.93 | 0.58 | 0.705605 | 1 |
| 151 | hsa00363 Bisphenol A degradation - Homo sapiens (human) | -805.017 | 0.591 | 0.7067 | 1 |
| 152 | hsa04930 Type II diabetes mellitus - Homo sapiens (human) | -6005.56 | 0.591 | 0.7067 | 1 |
| 153 | hsa01031 Glycan structures - biosynthesis 2 - Homo sapiens (human) | -12068.1 | 0.592 | 0.7067 | 1 |
| 154 | hsa04730 Long-term depression - Homo sapiens (human) | -9145.19 | 0.614 | 0.72841 | 1 |
| 155 | hsa00511 N-Glycan degradation - Homo sapiens (human) | -3218.62 | 0.642 | 0.752282 | 1 |
| 156 | hsa03022 Basal transcription factors - Homo sapiens (human) | -9252.51 | 0.642 | 0.752282 | 1 |
| 157 | hsa00350 Tyrosine metabolism - Homo sapiens (human) | -6839.97 | 0.658 | 0.766329 | 1 |
| 158 | hsa00053 Ascorbate and aldarate metabolism - Homo sapiens (human) | -1207.27 | 0.672 | 0.777891 | 1 |
| 159 | hsa03450 Non-homologous end-joining - Homo sapiens (human) | -5230.31 | 0.686 | 0.789313 | 1 |
| 160 | hsa05131 Pathogenic Escherichia coli infection - EPEC - Homo sapiens (human) | -8684.5 | 0.712 | 0.806444 | 1 |
| 161 | hsa00360 Phenylalanine metabolism - Homo sapiens (human) | -805.184 | 0.714 | 0.806444 | 1 |
| 162 | hsa00471 D-Glutamine and D-glutamate metabolism - Homo sapiens (human) | -408.8 | 0.719 | 0.806444 | 1 |
| 163 | hsa01040 Biosynthesis of unsaturated fatty acids - Homo sapiens (human) | -805.184 | 0.721 | 0.806444 | 1 |
| 164 | hsa03020 RNA polymerase - Homo sapiens (human) | -9655.56 | 0.722 | 0.806444 | 1 |
| 165 | hsa04640 Hematopoietic cell lineage - Homo sapiens (human) | -25752.4 | 0.735 | 0.812023 | 1 |
| 166 | hsa00440 Aminophosphonate metabolism - Homo sapiens (human) | -1609.49 | 0.737 | 0.812023 | 1 |
| 167 | hsa00930 Caprolactam degradation - Homo sapiens (human) | -1181.84 | 0.743 | 0.812023 | 1 |
| 168 | hsa01032 Glycan structures - degradation - Homo sapiens (human) | -7644.26 | 0.744 | 0.812023 | 1 |
| 169 | hsa03060 Protein export - Homo sapiens (human) | -2816.66 | 0.76 | 0.824773 | 1 |
| 170 | hsa03030 DNA replication - Homo sapiens (human) | -12873.8 | 0.772 | 0.833062 | 1 |
| 171 | hsa04140 Regulation of autophagy - Homo sapiens (human) | -4712.27 | 0.81 | 0.869157 | 1 |
| 172 | hsa00040 Pentose and glucuronate interconversions - Homo sapiens (human) | -3059.19 | 0.839 | 0.893796 | 1 |
| 173 | hsa05130 Pathogenic Escherichia coli infection - EHEC - Homo sapiens (human) | -8692.79 | 0.843 | 0.893796 | 1 |
| 174 | hsa03440 Homologous recombination - Homo sapiens (human) | -7242.7 | 0.847 | 0.893796 | 1 |
| 175 | hsa01030 Glycan structures - biosynthesis 1 - Homo sapiens (human) | -22533.1 | 0.876 | 0.919319 | 1 |
| 176 | hsa00031 Inositol metabolism - Homo sapiens (human) | -805.33 | 0.911 | 0.947734 | 1 |
| 177 | hsa00500 Starch and sucrose metabolism - Homo sapiens (human) | -6287.75 | 0.913 | 0.947734 | 1 |
| 178 | hsa00592 alpha-Linolenic acid metabolism - Homo sapiens (human) | -1194 | 0.92 | 0.948839 | 1 |
| 179 | hsa05340 Primary immunodeficiency - Homo sapiens (human) | -13683.8 | 0.924 | 0.948839 | 1 |
| 180 | hsa03430 Mismatch repair - Homo sapiens (human) | -4829.63 | 0.939 | 0.959086 | 1 |
| 181 | hsa04950 Maturity onset diabetes of the young - Homo sapiens (human) | -7027.14 | 0.953 | 0.964095 | 1 |
| 182 | hsa00643 Styrene degradation - Homo sapiens (human) | -1192.41 | 0.954 | 0.964095 | 1 |
| 183 | hsa04120 Ubiquitin mediated proteolysis - Homo sapiens (human) | -37827.5 | 0.974 | 0.979126 | 1 |
| 184 | hsa03050 Proteasome - Homo sapiens (human) | -16905.6 | 0.998 | 0.998 | 1 |
| 185 | hsa00364 Fluorobenzoate degradation - Homo sapiens (human) | -1 | -1 | -1 | -1 |
| 186 | hsa00472 D-Arginine and D-ornithine metabolism - Homo sapiens (human) | -1 | -1 | -1 | -1 |
| 187 | hsa00624 1- and 2-Methylnaphthalene degradation - Homo sapiens (human) | -1 | -1 | -1 | -1 |
| 188 | hsa00625 Tetrachloroethene degradation - Homo sapiens (human) | -1 | -1 | -1 | -1 |
| 189 | hsa00627 1,4-Dichlorobenzene degradation - Homo sapiens (human) | -1 | -1 | -1 | -1 |
| 190 | hsa00632 Benzoate degradation via CoA ligation - Homo sapiens (human) | -1 | -1 | -1 | -1 |
| 191 | hsa00740 Riboflavin metabolism - Homo sapiens (human) | -1 | -1 | -1 | -1 |
| 192 | hsa00791 Atrazine degradation - Homo sapiens (human) | -1 | -1 | -1 | -1 |
| 193 | hsa00902 Monoterpenoid biosynthesis - Homo sapiens (human) | -1 | -1 | -1 | -1 |
| 194 | hsa00903 Limonene and pinene degradation - Homo sapiens (human) | -1 | -1 | -1 | -1 |
| 195 | hsa01430 Cell Communication - Homo sapiens (human) | -1 | -1 | -1 | -1 |
| 196 | hsa04340 Hedgehog signaling pathway - Homo sapiens (human) | -1 | -1 | -1 | -1 |
| 197 | hsa04940 Type I diabetes mellitus - Homo sapiens (human) | -1 | -1 | -1 | -1 |
| 198 | hsa05016 - Homo sapiens (human) | -1 | -1 | -1 | -1 |
| 199 | hsa05322 Systemic lupus erythematosus - Homo sapiens (human) | -1 | -1 | -1 | -1 |

Result obtained by the GSEA methods in the pancreatic cancer dataset

| No | geneset | size | ES | NES | NOM p-value | FDR q-value | FWER p-value | Rank at max |
| --- | --- | --- | --- | --- | --- | --- | --- | --- |
| 1 | KEGG_PATHOGENIC_ESCHERICHIA_COLI_INFECTION | 43 | 0.675 | 1.907 | 0 | 0.08 | 0.049 | 2,693 |
| 2 | KEGG_SYSTEMIC_LUPUS_ERYTHEMATOSUS | 107 | 0.413 | 1.48 | 0.076 | 0.251 | 0.837 | 3,626 |
| 3 | KEGG_VASOPRESSIN_REGULATED_WATER_REABSORPTION | 44 | 0.447 | 1.485 | 0.03 | 0.254 | 0.831 | 4,238 |
| 4 | KEGG_PYRIMIDINE_METABOLISM | 88 | 0.475 | 1.486 | 0.088 | 0.262 | 0.831 | 5,994 |
| 5 | KEGG_AXON_GUIDANCE | 127 | 0.415 | 1.522 | 0.031 | 0.266 | 0.779 | 3,405 |
| 6 | KEGG_ECM_RECEPTOR_INTERACTION | 81 | 0.442 | 1.435 | 0.115 | 0.27 | 0.896 | 4,993 |
| 7 | KEGG_NUCLEOTIDE_EXCISION_REPAIR | 44 | 0.553 | 1.513 | 0.058 | 0.27 | 0.79 | 5,661 |
| 8 | KEGG_APOPTOSIS | 82 | 0.477 | 1.486 | 0.06 | 0.273 | 0.831 | 4,836 |
| 9 | KEGG_HOMOLOGOUS_RECOMBINATION | 26 | 0.561 | 1.418 | 0.104 | 0.275 | 0.916 | 6,313 |
| 10 | KEGG_ENDOCYTOSIS | 157 | 0.411 | 1.525 | 0.035 | 0.275 | 0.776 | 5,481 |
| 11 | KEGG_SNARE_INTERACTIONS_IN_VESICULAR_TRANSPORT | 34 | 0.494 | 1.436 | 0.091 | 0.276 | 0.895 | 4,562 |
| 12 | KEGG_PROSTATE_CANCER | 87 | 0.399 | 1.414 | 0.059 | 0.276 | 0.923 | 4,929 |
| 13 | KEGG_AMINO_SUGAR_AND_NUCLEOTIDE_SUGAR_METABOLISM | 44 | 0.495 | 1.421 | 0.113 | 0.278 | 0.915 | 4,833 |
| 14 | KEGG_ENDOMETRIAL_CANCER | 51 | 0.456 | 1.489 | 0.056 | 0.28 | 0.828 | 3,265 |
| 15 | KEGG_RNA_DEGRADATION | 51 | 0.529 | 1.453 | 0.093 | 0.281 | 0.882 | 5,689 |
| 16 | KEGG_DNA_REPLICATION | 34 | 0.63 | 1.424 | 0.14 | 0.281 | 0.909 | 6,259 |
| 17 | KEGG_CHRONIC_MYELOID_LEUKEMIA | 72 | 0.468 | 1.437 | 0.075 | 0.282 | 0.895 | 6,201 |
| 18 | KEGG_REGULATION_OF_ACTIN_CYTOSKELETON | 196 | 0.391 | 1.499 | 0.051 | 0.283 | 0.809 | 2,870 |
| 19 | KEGG_VIBRIO_CHOLERAE_INFECTION | 50 | 0.447 | 1.535 | 0.038 | 0.284 | 0.754 | 2,951 |
| 20 | KEGG_UBIQUITIN_MEDIATED_PROTEOLYSIS | 122 | 0.399 | 1.456 | 0.046 | 0.284 | 0.873 | 5,666 |
| 21 | KEGG_OOCYTE_MEIOSIS | 106 | 0.375 | 1.446 | 0.055 | 0.285 | 0.888 | 4,296 |
| 22 | KEGG_COLORECTAL_CANCER | 61 | 0.451 | 1.44 | 0.054 | 0.286 | 0.891 | 4,402 |
| 23 | KEGG_SMALL_CELL_LUNG_CANCER | 82 | 0.489 | 1.527 | 0.029 | 0.287 | 0.771 | 6,201 |
| 24 | KEGG_O_GLYCAN_BIOSYNTHESIS | 29 | 0.537 | 1.543 | 0.062 | 0.288 | 0.747 | 2,831 |
| 25 | KEGG_PANCREATIC_CANCER | 69 | 0.469 | 1.492 | 0.05 | 0.288 | 0.825 | 5,198 |
| 26 | KEGG_THYROID_CANCER | 29 | 0.534 | 1.614 | 0.016 | 0.292 | 0.573 | 3,422 |
| 27 | KEGG_FRUCTOSE_AND_MANNOSE_METABOLISM | 34 | 0.508 | 1.399 | 0.087 | 0.293 | 0.941 | 2,615 |
| 28 | KEGG_P53_SIGNALING_PATHWAY | 64 | 0.499 | 1.599 | 0.006 | 0.298 | 0.609 | 2,965 |
| 29 | KEGG_CELL_CYCLE | 113 | 0.519 | 1.586 | 0.031 | 0.301 | 0.637 | 4,105 |
| 30 | KEGG_PATHWAYS_IN_CANCER | 315 | 0.412 | 1.544 | 0.023 | 0.305 | 0.744 | 5,570 |
| 31 | KEGG_GLYCOLYSIS_GLUCONEOGENESIS | 60 | 0.45 | 1.36 | 0.125 | 0.308 | 0.963 | 1,933 |
| 32 | KEGG_BASAL_CELL_CARCINOMA | 53 | 0.453 | 1.561 | 0.041 | 0.31 | 0.702 | 4,145 |
| 33 | KEGG_LYSOSOME | 115 | 0.44 | 1.363 | 0.17 | 0.312 | 0.961 | 6,610 |
| 34 | KEGG_LEUKOCYTE_TRANSENDOTHELIAL_MIGRATION | 107 | 0.396 | 1.353 | 0.156 | 0.312 | 0.965 | 5,260 |
| 35 | KEGG_NOD_LIKE_RECEPTOR_SIGNALING_PATHWAY | 51 | 0.529 | 1.549 | 0.026 | 0.315 | 0.731 | 4,982 |
| 36 | KEGG_RENAL_CELL_CARCINOMA | 69 | 0.417 | 1.381 | 0.09 | 0.316 | 0.956 | 5,496 |
| 37 | KEGG_HEDGEHOG_SIGNALING_PATHWAY | 53 | 0.366 | 1.364 | 0.099 | 0.317 | 0.961 | 4,686 |
| 38 | KEGG_TIGHT_JUNCTION | 125 | 0.402 | 1.62 | 0.017 | 0.317 | 0.559 | 2,939 |
| 39 | KEGG_GALACTOSE_METABOLISM | 25 | 0.492 | 1.367 | 0.133 | 0.318 | 0.96 | 3,531 |
| 40 | KEGG_BASE_EXCISION_REPAIR | 33 | 0.601 | 1.568 | 0.042 | 0.32 | 0.688 | 5,147 |
| 41 | KEGG_FC_GAMMA_R_MEDIATED_PHAGOCYTOSIS | 87 | 0.436 | 1.343 | 0.146 | 0.323 | 0.969 | 4,580 |
| 42 | KEGG_B_CELL_RECEPTOR_SIGNALING_PATHWAY | 71 | 0.475 | 1.368 | 0.134 | 0.325 | 0.96 | 5,497 |
| 43 | KEGG_ADHERENS_JUNCTION | 73 | 0.511 | 1.704 | 0.004 | 0.326 | 0.375 | 4,935 |
| 44 | KEGG_FOCAL_ADHESION | 188 | 0.389 | 1.369 | 0.129 | 0.331 | 0.96 | 4,993 |
| 45 | KEGG_VIRAL_MYOCARDITIS | 67 | 0.438 | 1.334 | 0.179 | 0.332 | 0.971 | 4,552 |
| 46 | KEGG_HYPERTROPHIC_CARDIOMYOPATHY_HCM | 82 | 0.336 | 1.33 | 0.101 | 0.332 | 0.972 | 3,110 |
| 47 | KEGG_EPITHELIAL_CELL_SIGNALING_IN_HELICOBACTER_PYLORI_INFECTION | 62 | 0.509 | 1.628 | 0.021 | 0.342 | 0.538 | 4,982 |
| 48 | KEGG_LEISHMANIA_INFECTION | 62 | 0.489 | 1.315 | 0.212 | 0.343 | 0.98 | 6,927 |
| 49 | KEGG_PENTOSE_PHOSPHATE_PATHWAY | 26 | 0.47 | 1.319 | 0.177 | 0.344 | 0.979 | 3,176 |
| 50 | KEGG_ARRHYTHMOGENIC_RIGHT_VENTRICULAR_CARDIOMYOPATHY_ARVC | 73 | 0.458 | 1.734 | 0.007 | 0.353 | 0.297 | 2,774 |
| 51 | KEGG_NON_SMALL_CELL_LUNG_CANCER | 52 | 0.4 | 1.299 | 0.139 | 0.356 | 0.983 | 5,803 |
| 52 | KEGG_VEGF_SIGNALING_PATHWAY | 71 | 0.379 | 1.302 | 0.159 | 0.359 | 0.983 | 4,402 |
| 53 | KEGG_ANTIGEN_PROCESSING_AND_PRESENTATION | 81 | 0.386 | 1.278 | 0.235 | 0.378 | 0.986 | 3,567 |
| 54 | KEGG_WNT_SIGNALING_PATHWAY | 144 | 0.319 | 1.281 | 0.121 | 0.38 | 0.986 | 4,309 |
| 55 | KEGG_MISMATCH_REPAIR | 22 | 0.532 | 1.245 | 0.25 | 0.381 | 0.992 | 5,661 |
| 56 | KEGG_AMYOTROPHIC_LATERAL_SCLEROSIS_ALS | 49 | 0.385 | 1.246 | 0.186 | 0.385 | 0.992 | 5,408 |
| 57 | KEGG_BASAL_TRANSCRIPTION_FACTORS | 33 | 0.428 | 1.267 | 0.181 | 0.389 | 0.987 | 7,079 |
| 58 | KEGG_AMINOACYL_TRNA_BIOSYNTHESIS | 32 | 0.496 | 1.235 | 0.286 | 0.391 | 0.993 | 5,949 |
| 59 | KEGG_ERBB_SIGNALING_PATHWAY | 85 | 0.339 | 1.246 | 0.183 | 0.391 | 0.992 | 3,104 |
| 60 | KEGG_PROTEASOME | 42 | 0.691 | 1.632 | 0.019 | 0.391 | 0.525 | 3,399 |
| 61 | KEGG_RNA_POLYMERASE | 28 | 0.448 | 1.23 | 0.242 | 0.393 | 0.993 | 6,504 |
| 62 | KEGG_NOTCH_SIGNALING_PATHWAY | 41 | 0.408 | 1.247 | 0.22 | 0.395 | 0.992 | 5,120 |
| 63 | KEGG_TERPENOID_BACKBONE_BIOSYNTHESIS | 15 | 0.518 | 1.225 | 0.264 | 0.396 | 0.993 | 4,990 |
| 64 | KEGG_PROGESTERONE_MEDIATED_OOCYTE_MATURATION | 82 | 0.343 | 1.254 | 0.185 | 0.399 | 0.988 | 3,796 |
| 65 | KEGG_CYTOSOLIC_DNA_SENSING_PATHWAY | 51 | 0.368 | 1.249 | 0.236 | 0.4 | 0.991 | 4,836 |
| 66 | KEGG_NEUROTROPHIN_SIGNALING_PATHWAY | 122 | 0.363 | 1.257 | 0.191 | 0.4 | 0.987 | 5,334 |
| 67 | KEGG_SPLICEOSOME | 96 | 0.624 | 1.651 | 0.027 | 0.415 | 0.483 | 5,428 |
| 68 | KEGG_STEROID_BIOSYNTHESIS | 16 | 0.503 | 1.189 | 0.283 | 0.442 | 0.996 | 3,824 |
| 69 | KEGG_TOLL_LIKE_RECEPTOR_SIGNALING_PATHWAY | 98 | 0.352 | 1.19 | 0.269 | 0.446 | 0.996 | 6,841 |
| 70 | KEGG_RIG_I_LIKE_RECEPTOR_SIGNALING_PATHWAY | 65 | 0.318 | 1.18 | 0.262 | 0.45 | 0.997 | 5,114 |
| 71 | KEGG_INSULIN_SIGNALING_PATHWAY | 131 | 0.305 | 1.177 | 0.217 | 0.45 | 0.997 | 3,322 |
| 72 | KEGG_ACUTE_MYELOID_LEUKEMIA | 56 | 0.387 | 1.163 | 0.304 | 0.46 | 0.998 | 5,514 |
| 73 | KEGG_DILATED_CARDIOMYOPATHY | 89 | 0.299 | 1.164 | 0.243 | 0.465 | 0.997 | 2,774 |
| 74 | KEGG_OLFACTORY_TRANSDUCTION | 114 | -0.472 | -1.572 | 0.051 | 0.473 | 0.665 | 8,843 |
| 75 | KEGG_PURINE_METABOLISM | 149 | 0.315 | 1.146 | 0.292 | 0.482 | 0.998 | 5,994 |
| 76 | KEGG_CELL_ADHESION_MOLECULES_CAMS | 125 | 0.344 | 1.14 | 0.324 | 0.485 | 0.999 | 1,930 |
| 77 | KEGG_TGF_BETA_SIGNALING_PATHWAY | 82 | 0.31 | 1.119 | 0.311 | 0.508 | 0.999 | 4,461 |
| 78 | KEGG_STARCH_AND_SUCROSE_METABOLISM | 39 | 0.381 | 1.113 | 0.32 | 0.512 | 0.999 | 1,940 |
| 79 | KEGG_CHEMOKINE_SIGNALING_PATHWAY | 173 | 0.325 | 1.12 | 0.34 | 0.514 | 0.999 | 4,836 |
| 80 | KEGG_RIBOFLAVIN_METABOLISM | 16 | 0.38 | 1.108 | 0.316 | 0.514 | 0.999 | 1,053 |
| 81 | KEGG_BLADDER_CANCER | 40 | 0.342 | 1.095 | 0.326 | 0.522 | 0.999 | 3,571 |
| 82 | KEGG_MELANOGENESIS | 98 | 0.286 | 1.097 | 0.328 | 0.525 | 0.999 | 4,238 |
| 83 | KEGG_NATURAL_KILLER_CELL_MEDIATED_CYTOTOXICITY | 129 | 0.29 | 1.086 | 0.366 | 0.531 | 0.999 | 4,309 |
| 84 | KEGG_T_CELL_RECEPTOR_SIGNALING_PATHWAY | 107 | 0.328 | 1.076 | 0.418 | 0.543 | 0.999 | 6,201 |
| 85 | KEGG_HUNTINGTONS_DISEASE | 164 | 0.34 | 1.061 | 0.404 | 0.561 | 0.999 | 6,448 |
| 86 | KEGG_PRION_DISEASES | 35 | 0.325 | 1.033 | 0.426 | 0.576 | 0.999 | 3,917 |
| 87 | KEGG_SPHINGOLIPID_METABOLISM | 30 | 0.355 | 1.046 | 0.425 | 0.58 | 0.999 | 3,738 |
| 88 | KEGG_PORPHYRIN_AND_CHLOROPHYLL_METABOLISM | 31 | 0.372 | 1.026 | 0.432 | 0.581 | 0.999 | 5,302 |
| 89 | KEGG_ALZHEIMERS_DISEASE | 149 | 0.313 | 1.033 | 0.439 | 0.581 | 0.999 | 6,496 |
| 90 | KEGG_GLIOMA | 63 | 0.28 | 1.037 | 0.41 | 0.582 | 0.999 | 6,135 |
| 91 | KEGG_GLYCOSAMINOGLYCAN_DEGRADATION | 21 | 0.376 | 1.041 | 0.392 | 0.582 | 0.999 | 5,499 |
| 92 | KEGG_DORSO_VENTRAL_AXIS_FORMATION | 23 | 0.344 | 1.017 | 0.426 | 0.591 | 0.999 | 5,792 |
| 93 | KEGG_PRIMARY_BILE_ACID_BIOSYNTHESIS | 16 | -0.558 | -1.586 | 0.034 | 0.643 | 0.647 | 3,502 |
| 94 | KEGG_MAPK_SIGNALING_PATHWAY | 256 | 0.225 | 0.982 | 0.482 | 0.643 | 1 | 4,999 |
| 95 | KEGG_MELANOMA | 71 | 0.254 | 0.977 | 0.511 | 0.646 | 1 | 3,778 |
| 96 | KEGG_DRUG_METABOLISM_OTHER_ENZYMES | 39 | 0.324 | 0.941 | 0.554 | 0.701 | 1 | 5,195 |
| 97 | KEGG_GLYCOSPHINGOLIPID_BIOSYNTHESIS_LACTO_AND_NEOLACTO_SERIES | 26 | 0.314 | 0.902 | 0.578 | 0.76 | 1 | 1,277 |
| 98 | KEGG_ALDOSTERONE_REGULATED_SODIUM_REABSORPTION | 40 | 0.264 | 0.888 | 0.612 | 0.778 | 1 | 2,428 |
| 99 | KEGG_GLYCOSAMINOGLYCAN_BIOSYNTHESIS_KERATAN_SULFATE | 15 | 0.325 | 0.878 | 0.631 | 0.787 | 1 | 1,795 |
| 100 | KEGG_CALCIUM_SIGNALING_PATHWAY | 171 | -0.323 | -1.41 | 0.041 | 0.806 | 0.92 | 4,384 |
| 101 | KEGG_GLYCINE_SERINE_AND_THREONINE_METABOLISM | 31 | -0.518 | -1.445 | 0.07 | 0.824 | 0.879 | 1,105 |
| 102 | KEGG_MATURITY_ONSET_DIABETES_OF_THE_YOUNG | 19 | -0.503 | -1.366 | 0.119 | 0.845 | 0.946 | 5,622 |
| 103 | KEGG_ALLOGRAFT_REJECTION | 34 | 0.339 | 0.819 | 0.667 | 0.856 | 1 | 1,930 |
| 104 | KEGG_HEMATOPOIETIC_CELL_LINEAGE | 83 | 0.26 | 0.813 | 0.686 | 0.857 | 1 | 1,718 |
| 105 | KEGG_TYPE_I_DIABETES_MELLITUS | 40 | 0.305 | 0.822 | 0.645 | 0.86 | 1 | 4,763 |
| 106 | KEGG_OXIDATIVE_PHOSPHORYLATION | 110 | 0.298 | 0.826 | 0.62 | 0.861 | 1 | 6,570 |
| 107 | KEGG_GRAFT_VERSUS_HOST_DISEASE | 37 | 0.323 | 0.829 | 0.628 | 0.865 | 1 | 3,917 |
| 108 | KEGG_CYTOKINE_CYTOKINE_RECEPTOR_INTERACTION | 245 | 0.195 | 0.788 | 0.747 | 0.876 | 1 | 4,075 |
| 109 | KEGG_CYSTEINE_AND_METHIONINE_METABOLISM | 31 | 0.242 | 0.797 | 0.758 | 0.877 | 1 | 1,867 |
| 110 | KEGG_LONG_TERM_POTENTIATION | 68 | 0.196 | 0.791 | 0.812 | 0.878 | 1 | 2,707 |
| 111 | KEGG_PHENYLALANINE_METABOLISM | 18 | 0.271 | 0.747 | 0.825 | 0.915 | 1 | 1,684 |
| 112 | KEGG_COMPLEMENT_AND_COAGULATION_CASCADES | 66 | 0.223 | 0.75 | 0.802 | 0.92 | 1 | 2,560 |
| 113 | KEGG_FC_EPSILON_RI_SIGNALING_PATHWAY | 75 | 0.209 | 0.755 | 0.801 | 0.92 | 1 | 3,652 |
| 114 | KEGG_BIOSYNTHESIS_OF_UNSATURATED_FATTY_ACIDS | 19 | 0.281 | 0.714 | 0.831 | 0.933 | 1 | 6,415 |
| 115 | KEGG_ONE_CARBON_POOL_BY_FOLATE | 16 | 0.278 | 0.715 | 0.8 | 0.939 | 1 | 3,605 |
| 116 | KEGG_PHOSPHATIDYLINOSITOL_SIGNALING_SYSTEM | 67 | 0.202 | 0.694 | 0.869 | 0.943 | 1 | 5,739 |
| 117 | KEGG_ARGININE_AND_PROLINE_METABOLISM | 52 | 0.232 | 0.717 | 0.856 | 0.945 | 1 | 2,527 |
| 118 | KEGG_ADIPOCYTOKINE_SIGNALING_PATHWAY | 66 | 0.205 | 0.698 | 0.928 | 0.946 | 1 | 4,836 |
| 119 | KEGG_GLYCEROPHOSPHOLIPID_METABOLISM | 64 | 0.189 | 0.663 | 0.926 | 0.948 | 1 | 4,702 |
| 120 | KEGG_LONG_TERM_DEPRESSION | 66 | 0.181 | 0.682 | 0.935 | 0.95 | 1 | 2,449 |
| 121 | KEGG_GAP_JUNCTION | 75 | 0.197 | 0.718 | 0.862 | 0.951 | 1 | 4,457 |
| 122 | KEGG_GNRH_SIGNALING_PATHWAY | 97 | 0.161 | 0.666 | 0.974 | 0.953 | 1 | 5,325 |
| 123 | KEGG_TYPE_II_DIABETES_MELLITUS | 44 | 0.2 | 0.667 | 0.892 | 0.96 | 1 | 2,428 |
| 124 | KEGG_PYRUVATE_METABOLISM | 40 | 0.211 | 0.635 | 0.878 | 0.97 | 1 | 1,615 |
| 125 | KEGG_MTOR_SIGNALING_PATHWAY | 46 | 0.162 | 0.557 | 0.982 | 0.983 | 1 | 5,744 |
| 126 | KEGG_AUTOIMMUNE_THYROID_DISEASE | 49 | 0.199 | 0.591 | 0.829 | 0.984 | 1 | 1,930 |
| 127 | KEGG_PRIMARY_IMMUNODEFICIENCY | 35 | 0.2 | 0.538 | 0.919 | 0.985 | 1 | 2,634 |
| 128 | KEGG_GLYCOSYLPHOSPHATIDYLINOSITOL_GPI_ANCHOR_BIOSYNTHESIS | 24 | 0.204 | 0.578 | 0.936 | 0.985 | 1 | 4,927 |
| 129 | KEGG_STEROID_HORMONE_BIOSYNTHESIS | 45 | 0.178 | 0.562 | 0.969 | 0.987 | 1 | 2,956 |
| 130 | KEGG_CITRATE_CYCLE_TCA_CYCLE | 29 | 0.198 | 0.46 | 0.957 | 0.987 | 1 | 6,376 |
| 131 | KEGG_N_GLYCAN_BIOSYNTHESIS | 42 | 0.168 | 0.513 | 0.976 | 0.988 | 1 | 7,389 |
| 132 | KEGG_GLYCOSPHINGOLIPID_BIOSYNTHESIS_GANGLIO_SERIES | 15 | 0.209 | 0.596 | 0.958 | 0.988 | 1 | 3,431 |
| 133 | KEGG_PENTOSE_AND_GLUCURONATE_INTERCONVERSIONS | 18 | 0.191 | 0.479 | 0.991 | 0.99 | 1 | 249 |
| 134 | KEGG_PARKINSONS_DISEASE | 106 | 0.218 | 0.598 | 0.8 | 0.995 | 1 | 6,448 |
| 135 | KEGG_INTESTINAL_IMMUNE_NETWORK_FOR_IGA_PRODUCTION | 45 | -0.174 | -0.49 | 0.968 | 0.997 | 1 | 5,631 |
| 136 | KEGG_RIBOSOME | 79 | -0.136 | -0.362 | 0.949 | 0.997 | 1 | 5,126 |
| 137 | KEGG_HISTIDINE_METABOLISM | 27 | -0.194 | -0.53 | 0.972 | 1 | 1 | 5,685 |
| 138 | KEGG_JAK_STAT_SIGNALING_PATHWAY | 151 | -0.133 | -0.567 | 0.989 | 1 | 1 | 5,822 |
| 139 | KEGG_ASCORBATE_AND_ALDARATE_METABOLISM | 16 | -0.235 | -0.602 | 0.902 | 1 | 1 | 15,774 |
| 140 | KEGG_TYROSINE_METABOLISM | 39 | -0.194 | -0.627 | 0.96 | 1 | 1 | 4,219 |
| 141 | KEGG_VASCULAR_SMOOTH_MUSCLE_CONTRACTION | 110 | -0.165 | -0.639 | 0.969 | 1 | 1 | 4,526 |
| 142 | KEGG_LYSINE_DEGRADATION | 43 | -0.197 | -0.642 | 0.924 | 1 | 1 | 2,962 |
| 143 | KEGG_INOSITOL_PHOSPHATE_METABOLISM | 46 | -0.19 | -0.653 | 0.948 | 1 | 1 | 585 |
| 144 | KEGG_PEROXISOME | 72 | -0.226 | -0.655 | 0.795 | 1 | 1 | 6,276 |
| 145 | KEGG_GLYOXYLATE_AND_DICARBOXYLATE_METABOLISM | 15 | -0.273 | -0.665 | 0.816 | 1 | 1 | 4,111 |
| 146 | KEGG_ALANINE_ASPARTATE_AND_GLUTAMATE_METABOLISM | 32 | -0.215 | -0.666 | 0.932 | 1 | 1 | 2,942 |
| 147 | KEGG_TRYPTOPHAN_METABOLISM | 38 | -0.231 | -0.701 | 0.84 | 1 | 1 | 1,943 |
| 148 | KEGG_ARACHIDONIC_ACID_METABOLISM | 51 | -0.218 | -0.701 | 0.921 | 1 | 1 | 2,999 |
| 149 | KEGG_GLYCEROLIPID_METABOLISM | 43 | -0.209 | -0.712 | 0.913 | 1 | 1 | 2,750 |
| 150 | KEGG_PPAR_SIGNALING_PATHWAY | 66 | -0.272 | -0.716 | 0.841 | 1 | 1 | 6,761 |
| 151 | KEGG_ABC_TRANSPORTERS | 44 | -0.225 | -0.726 | 0.854 | 1 | 1 | 7,097 |
| 152 | KEGG_GLYCOSAMINOGLYCAN_BIOSYNTHESIS_HEPARAN_SULFATE | 25 | -0.217 | -0.734 | 0.845 | 1 | 1 | 1,482 |
| 153 | KEGG_LINOLEIC_ACID_METABOLISM | 27 | -0.31 | -0.746 | 0.735 | 1 | 1 | 7,915 |
| 154 | KEGG_RETINOL_METABOLISM | 49 | -0.303 | -0.753 | 0.757 | 1 | 1 | 3,995 |
| 155 | KEGG_ASTHMA | 27 | -0.303 | -0.764 | 0.655 | 1 | 1 | 14,372 |
| 156 | KEGG_CARDIAC_MUSCLE_CONTRACTION | 70 | -0.212 | -0.766 | 0.737 | 1 | 1 | 5,748 |
| 157 | KEGG_BUTANOATE_METABOLISM | 31 | -0.302 | -0.783 | 0.702 | 1 | 1 | 5,691 |
| 158 | KEGG_NICOTINATE_AND_NICOTINAMIDE_METABOLISM | 21 | -0.256 | -0.786 | 0.755 | 1 | 1 | 2,273 |
| 159 | KEGG_DRUG_METABOLISM_CYTOCHROME_P450 | 61 | -0.325 | -0.821 | 0.738 | 1 | 1 | 802 |
| 160 | KEGG_ETHER_LIPID_METABOLISM | 28 | -0.233 | -0.824 | 0.784 | 1 | 1 | 1,231 |
| 161 | KEGG_PANTOTHENATE_AND_COA_BIOSYNTHESIS | 16 | -0.321 | -0.832 | 0.647 | 1 | 1 | 5,912 |
| 162 | KEGG_FATTY_ACID_METABOLISM | 39 | -0.373 | -0.857 | 0.691 | 1 | 1 | 7,479 |
| 163 | KEGG_GLUTATHIONE_METABOLISM | 45 | -0.28 | -0.866 | 0.62 | 1 | 1 | 802 |
| 164 | KEGG_PROTEIN_EXPORT | 20 | -0.383 | -0.879 | 0.595 | 1 | 1 | 2,383 |
| 165 | KEGG_METABOLISM_OF_XENOBIOTICS_BY_CYTOCHROME_P450 | 60 | -0.339 | -0.889 | 0.683 | 1 | 1 | 802 |
